# Synthesis of ciprofloxacin-linked 1,2,3-triazole conjugates as potent antibacterial agents using click chemistry: exploring their function as DNA gyrase inhibitors *via in silico*- and *in vitro*-based studies†

**DOI:** 10.1039/d4ra01332h

**Published:** 2024-05-30

**Authors:** Upendra Kumar Patel, Punit Tiwari, Ragini Tilak, Gaurav Joshi, Roshan Kumar, Alka Agarwal

**Affiliations:** a Department of Medicinal Chemistry, Institute of Medical Sciences, Banaras Hindu University Varanasi UP-221005 India agarwal.dralka@gmail.com; b Department of Microbiology, Institute of Medical Sciences, Banaras Hindu University Varanasi UP-221005 India; c Department of Pharmaceutical Sciences, Hemvati Nandan Bahuguna Garhwal University (Central University) Dist. Garhwal (Uttarakhand) Srinagar-246174 India; d Department of Microbiology, Central University of Punjab Ghudda Bathinda-151401 India

## Abstract

The antibacterial efficacy of some newly developed C-3 carboxylic group-containing ciprofloxacin-linked 1,2,3-triazole conjugates was studied. Twenty-one compounds from three different series of triazoles were synthesized using click chemistry and evaluated for their antibacterial activity against nine different pathogenic strains, including three Gram-positive strains, *i.e. Enterococcus faecalis* (ATCC29212), *Staphylococcus aureus* (ATCC25923), *Staphylococcus epidermidis* (clinical isolate), and six Gram-negative bacterial strains, *i.e. Escherichia coli* (ATCC25922), *Pseudomonas aeruginosa* (ATCC27853), *Salmonella typhi* (clinical isolate), *Proteus mirabilis* (clinical isolate), *Acinetobacter baumannii* (clinical isolate) and *Klebsiella pneumonia* (clinical isolate). Among the compounds, 10, 10a, 10b, 10c, 10d, 11a, 11f, 12c, 12e and 12f showed excellent activity with MIC values upto 12.5 μg mL^−1^, whereas the control ciprofloxacin showed MIC values of 0.781–25 μg mL^−1^ towards various strains. In addition, the low toxicity profile of the synthesized molecules revealed that they are potent antibiotics. Molecular docking and MD analysis were performed using the protein structure of *E. coli* DNA gyrase B, which was further corroborated with an *in vitro* assay to evaluate the inhibition of DNA gyrase. The analysis revealed that compound 10b was the most potent inhibitor of DNA gyrase compared to ciprofloxacin, which was employed as the positive control. Furthermore, the structure of two title compounds (11a and 12d) was characterized using single-crystal analysis.

## Abstract 2

The antibacterial efficacy of some newly developed C-3 carboxylic group-containing ciprofloxacin-linked 1,2,3-triazole conjugates was studied.

## Graphical abstract

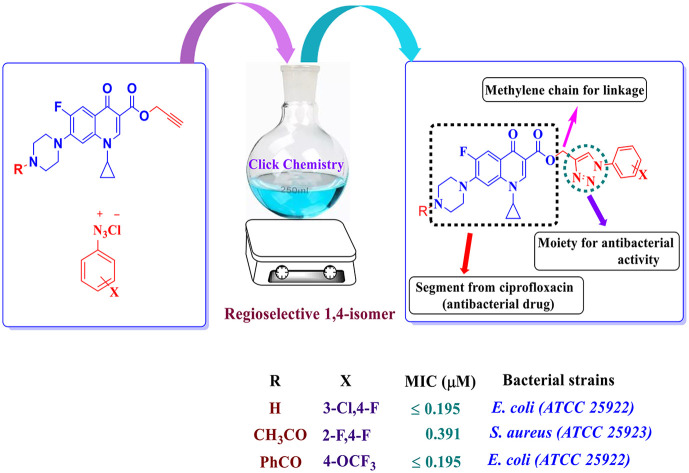

## Introduction

Fluoroquinolones are the most commonly recommended antibiotics for treating bacterial infections. They possess broad antibacterial activity, good efficacy profiles, and efficient pharmacokinetics.^1^ Accordingly, second-generation fluoroquinolones such as ciprofloxacin, ofloxacin, enoxacin, and norfloxacin are used to treat numerous bacterial infections. Besides, they are essential for the treatment of sexually transmitted diseases (STDs), upper and lower respiratory tract infections, and urinary tract infections (UTI) and exhibit antitubercular and antitumor activities. In addition, they can be employed to treat the infections of the skin, soft tissues, gastrointestinal system, bones, joints as well as nosocomial infections and chronic osteomyelitis.^2–4^ Among the second-generation fluoroquinolones, ciprofloxacin is the most popular broad-spectrum, synthetic chemotherapeutic antibiotic that has been approved for the treatment of infections, including chronic bacterial prostatitis, acute uncomplicated cystitis, shigellosis, urinary tract infections and acute sinusitis.^5,6^

**Fig. 1:**
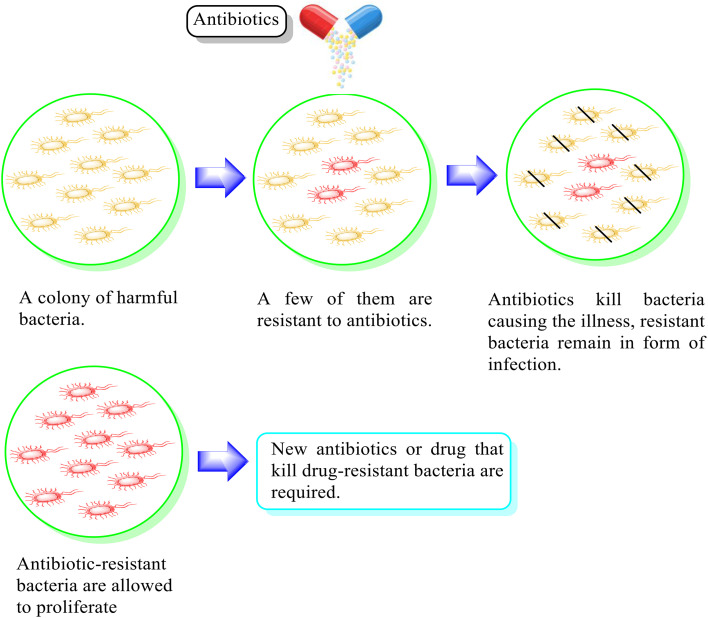
Illustration of the antimicrobial effect mediated by the antibiotic and associated mechanisms of drug resistance.

Ciprofloxacin functions by targeting bacterial DNA gyrase and topoisomerase IV (Topo IV), forming quinolone ternary complexes when it interacts with DNA and DNA gyrase or Topo IV. The antibacterial effectiveness of fluoroquinolone is due to the generation of these complexes, which hinder DNA replication and cell growth.^7^ However, the excessive use of ciprofloxacin has resulted in drug resistance, as discussed in the literature^8,9^ (Fig. 1). This developed drug resistance in bacteria can outlast other methods of prophylaxis or treatment.^10^ Consequently, infectious diseases carried through persistent bacteria, viruses, and fungi tend to pose a significant global problem, and if not addressed, they may result in 10 million fatalities annually by 2050.^11^ In this case, concomitant medication is one of the tactics that has shown some clinical effectiveness in preventing or delaying the emergence of resistance.^12^ It depends on the combination of two or more antibiotics, each having a unique mode of action, which reduces the chance of cells acquiring resistance.^13^ The pharmacokinetic characteristics of drugs in combination are considerably more likely to be changed; therefore, therapeutic results *in vivo* will not always coincide with *in vitro* outcomes.^14,15^ Designing hybrid antibacterial agents is another effective strategy that reduces the limitations of combination treatment, which integrates two or more active antibiotic structures with different bacterial targets in the same molecular framework *via* one or more chemical linkages. This approach creates a potential tool that reduces the anticipated side effects. Hybrid approaches have become popular due to their useful function in inhibiting the emergence of bacterial resistance through increased affinity and efficacy compared to the parent medications.^11,16–18^ They serve as novel leads with alternative actions and several pharmacological targets given that a single hybrid compound targets bacterial cells *via* different mechanisms of action. Novel hybrid molecules improve the ability to enhance the pharmacokinetic characteristics, toxicity profiles, and retention of drugs.^19,20^ A rationally designed linker between two bioactive groups may also improve the chance of both drug targets showing a lower incidence of new resistance mutations and may even be able to reduce existing drug resistance mechanisms.^21^

To control the prevailing drug resistance, drug development to treat bacterial infections is necessary. In this case, click chemistry is a vital synthetic approach that involves a set of chemical reactions and ensures high yield with the comprehensive option of incorporating numerous chemical space parameters into a drug candidate. The versatility and efficiency click chemistry^22^ also ensure the outcome of the reaction, making it a convenient tool in various chemistry domains, which include drug discovery, materials science, and bioconjugation. Click chemistry also enables the fast synthesis of complex molecules and diverse heterocycles, thus paving the way for developing new pharmaceuticals with significantly improved efficacy and reduced side reactions or off-target effects. In general, click chemistry combines two similar or diverse pharmacophoric groups *via* a specific skeleton to yield a new analogue with synergistic biological attributes, thus ensuring that this technique is viable for lead compound discovery.^23–25^ The 1,2,3-triazole ring system, formed from the Huisgen 1,3-dipolar cycloaddition^26^ of azides and alkynes *via* copper-catalyzed click reaction, is a reasonably well-known pharmacophore.^27–29^ This moiety is a preferred linking unit because it is robust under oxidative and reductive conditions, resilient to metabolic degradation, actively binds to biomolecular targets and increases its solubility through hydrogen bonding and dipole interactions.^30,31^ Triazole has attracted particular interest in recent years in the search for novel antibiotics because various pharmacological molecules, *i.e.* cephalosporin, tazobactam, and cefatrizine, used to treat bacterial infections have a 1,2,3-triazole group.

**Fig. 2:**
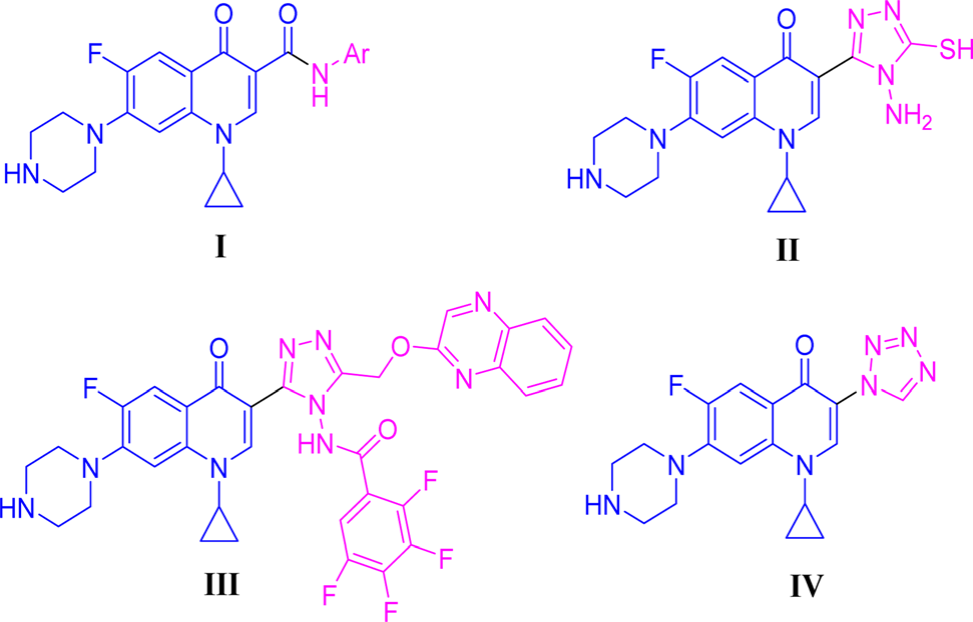
Structures of some C-3-modified ciprofloxacin hybrids.

Positions C-7 (piperazine) and C-3 (carboxylic) of ciprofloxacin, which are the most flexible sites for chemical modification and an area that considerably determines the potency, have been the focus of research on the ciprofloxacin moiety. Unfortunately, due to the insufficient information in the literature regarding ciprofloxacin analogues at the carboxylic position (C-3), they have not attracted as much attention as C-7, which has been prevalent in recent years.^32^ However, it has been observed that the incorporation of carboxamide (I),^33^ triazole (II and III)^34,35^ and tetrazole (IV)^36^ rings at the C-3 position significantly increases the antibacterial activity of synthesized hybrids in comparison to ciprofloxacin. Some C-3-modified ciprofloxacin hybrids are shown in Fig. 2. From the above discussion, there is scope for further investigation at the C-3 position of ciprofloxacin and it has often been observed that chemical transformation enhances the activity compared to a parent molecule.

**Scheme 1:**
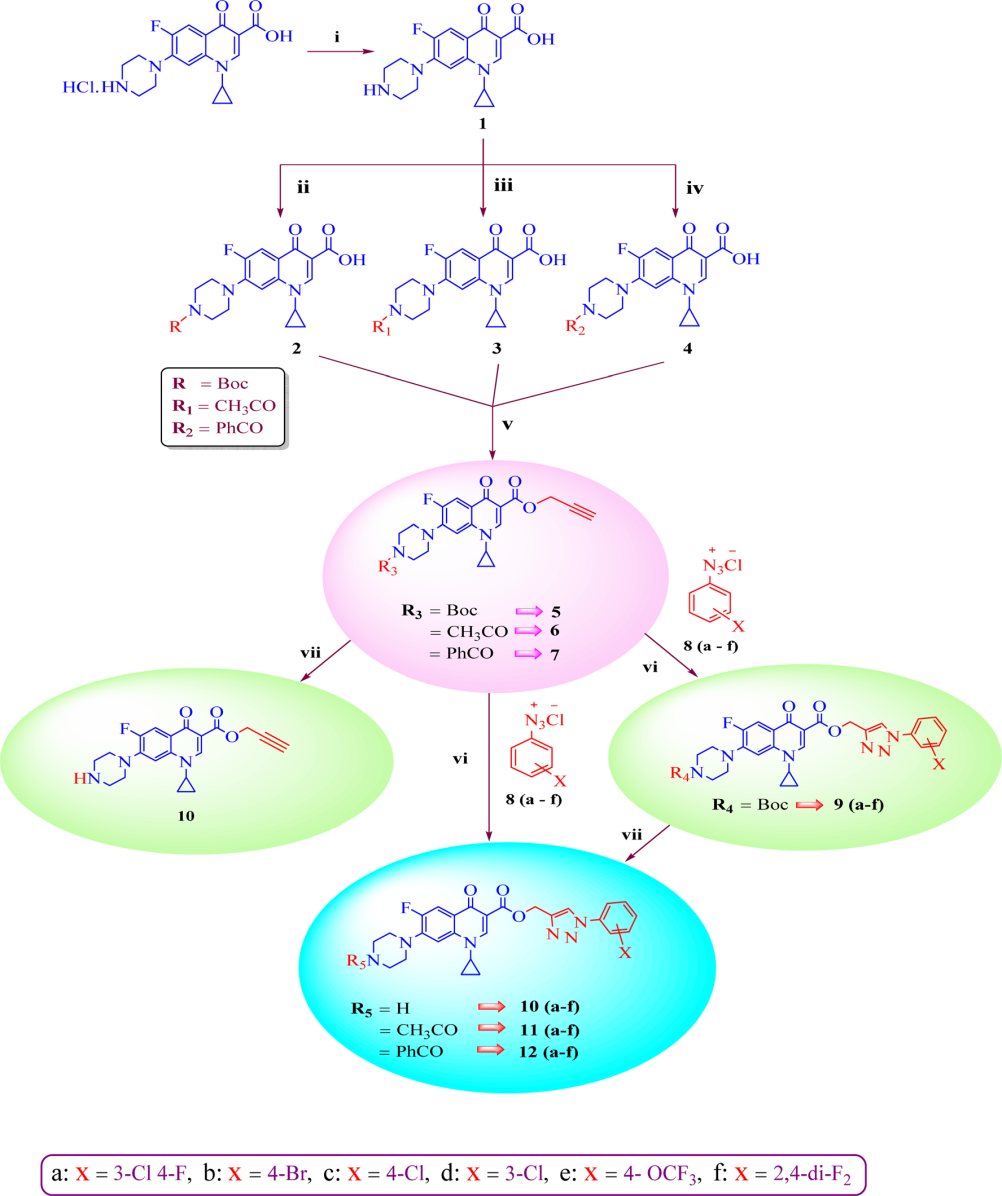
Systematic strategy for the synthesis of ciprofloxacin-triazole hybrids 9–12(a–f). Reagents and conditions (i) = 5% aq. NaHCO_3_, (ii) = Boc_2_O, 1 M NaOH, THF, rt 16 h, (iii) = 2 M NaOH, CH_3_COC1, 1,4-dioxane, stirred, 0 °C to rt 2 h, (iv) = 2 M NaOH, PhCOC1, 1,4-dioxane, stirred, 0 °C to rt 2 h, (v) = NaHCO_3_, propargyl bromide, DMF, stirred 100 °C 48 h, (vi) = CuSO_4_·5H_2_O, sodium ascorbate, DMF/H_2_O (4 : 1), stirred 16–25 h, rt, click chemistry, (vii) = DCM(dry), TFA, rt 24 h.

Our group has been working on the design and synthesis of various small molecules with antimicrobial activity.^37,38^ Encouraged by our earlier research,^39,40^ we aimed to develop a new and more effective series of ciprofloxacin conjugates using click chemistry for antibacterial findings (Scheme 1). Herein, we synthesized a new series of ciprofloxacin analogs with their C-3 carboxylic groups linked to various substituted 1,2,3-triazole rings and variations at the piperazine moiety. Our main goal was to improve the effectiveness of the synthesized compounds against Gram-positive and Gram-negative bacteria, while assessing their toxicity to human red blood cells. Also, they were further tested using the DNA gyrase assay as a possible target and molecular docking studies were conducted to investigate their binding mechanisms.

## Results and discussion

### Chemistry

A systematic approach was used to create novel 1,2,3-triazole analogs of ciprofloxacin, as displayed in Scheme 1. Triazole derivatives 9–12(a–f) were synthesized in several steps. In the first synthetic step, free ciprofloxacin (1) was generated by solubilizing ciprofloxacin hydrochloride in a 5% aqueous solution of NaHCO_3_. In the next step, free ciprofloxacin was treated with Boc-anhydride, acyl chloride, and benzoyl chloride under different reaction conditions to ciprofloxacin products with a nitrogen-protected piperazine moiety (2, 3 and 4). Subsequently, the products (2, 3 and 4) were treated with propargyl bromide at 100 °C in the presence of NaHCO_3_ to obtain the propargylated products (5, 6 and 7, respectively). In the final step (for 11 and 12), the propargylated products (5, 6 and 7) underwent copper-catalyzed [3 + 2] cycloaddition reactions with various substituted aromatic azides 8(a–f) to afford 21 compounds 6, 7, 10 and 10–12(a–f). Compounds 5 and 9(a–f) were dissolved in a mixture of trifluoroacetic acid and dichloromethane (1 : 4 v/v, 5 mL) to obtain the deprotected products 10 and 10(a–f), respectively. The experimental section includes a description of the general synthesis process for each compound. The ^1^H NMR, ^13^C NMR, ^19^F NMR, IR and mass spectroscopy data validated the structures of all the synthesized analogues. Also, the X-ray crystallographic investigation supported the structure of compounds 11a and 12d.

### Mechanism for the formation of 1,4 regioisomer

**Fig. 3:**
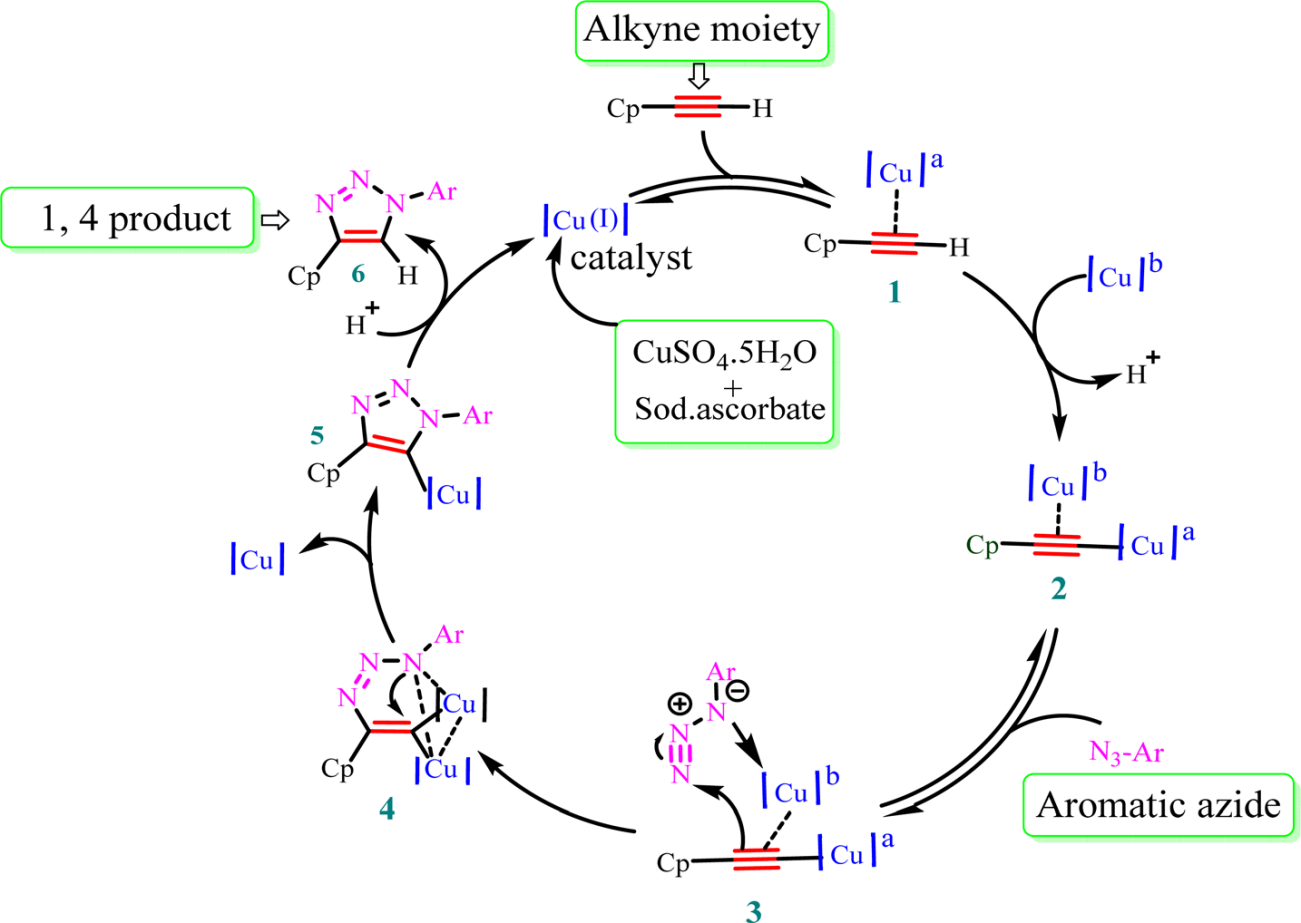
Plausible mechanism for Cu(i)-catalyzed alkyne–azide dipolar cycloaddition. Cp = ciprofloxacin and a,b = chemically equivalent copper atoms.

The stepwise catalytic cycle begins with the formation of a Cu–alkyne π complex (1), followed by deprotonation of the alkyne proton to form copper acetylide (2), as shown in Fig. 3. The coordination of copper increases the acidity of the acetylenic proton and facilitates deprotonation in the aqueous medium. One of the copper ions from species (2) coordinates with azide nitrogen (3) and activates it toward attack of the terminal nitrogen of the azide group on the alkyne carbon, leading to the synthesis of metallacycle (4). This metallacycle undergoes ring contraction *via* the transannular interaction between the lone pair of electrons present on the azide nitrogen and the carbon–copper double bond. Subsequently, Cu triazolide species (5) is formed, which undergoes protonation to generate 1,4-disubstituted triazole (6) and the Cu(i) catalyst.^41^

### Biological activity

#### Antibacterial activity

An analysis of the twenty-one synthesized compounds, *i.e.*, 6, 7, 10, 10(a–f), 11(a–f) and 12(a–f), with the ciprofloxacin C-3 carboxylic group linked with 1,2,3-triazole on one side *via* propargylation, accompanied by copper-catalyzed azide–alkyne [3 + 2] cycloaddition and varying groups (Boc, acyl and benzoyl) on the NH of piperazine moiety on the other side of ciprofloxacin was carried out. The hybrid molecules, *i.e.*, 6, 7, 10, 10(a–f), 11(a–f) and 12(a–f), were tested against a variety of bacterial strains to determine their antibacterial action and minimum inhibitory concentration (MIC). Three Gram-positive bacterial strains, *i.e.*, *Enterococcus faecalis* (ATCC29212), *Staphylococcus aureus* (ATCC25923), and *Staphylococcus epidermidis* (clinical isolate), and six Gram-negative bacterial strains, *i.e.*, *Escherichia coli* (ATCC25922), *Pseudomonas aeruginosa* (ATCC27853), *Salmonella typhi* (clinical isolate), *Proteus mirabilis* (clinical isolate), *Acinetobacter baumannii* (clinical isolate) and *Klebsiella pneumoniae* (clinical isolate), were used to test the antibacterial activity of the compounds. The MIC results demonstrated that the substituent type, position, and isomeric effects on the benzene ring have a major impact on the antibacterial assay of the compounds (Table 2). According to Table 2, it is obvious that several of the prepared compounds are more potent against certain bacterial strains except for compounds 11b, 11c and 11d, which were not active against any of the tested bacterial strains. The most potent active compounds possess higher or equal antibacterial activity in terms of minimum inhibitory concentration (MIC) compared to the standard drug, as seen in Table 2.

#### Structure–activity relationship

**Fig. 4:**
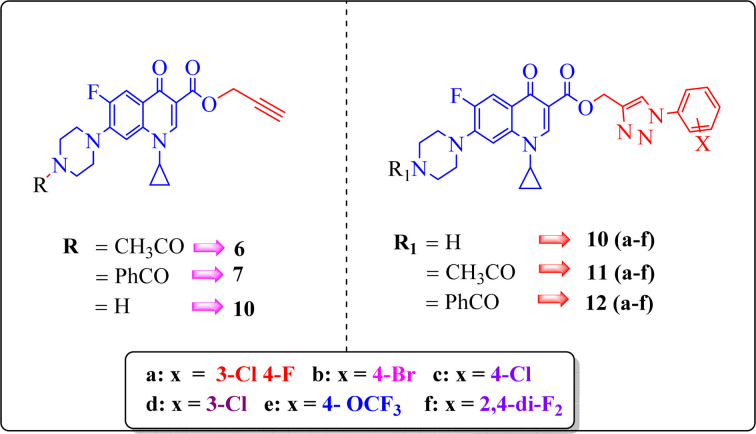
Structure–activity relationship of synthesized ciprofloxacin hybrids.

Here, we summarize the antibacterial activity and effect of the substituents in the synthesized compounds, as presented in Fig. 4. Compound 6, having an acetyl group on piperazine together with a propargyl chain on the carboxylic oxygen moiety of ciprofloxacin, showed activity against *S. aureus* (ATCC25923) and *E. coli* (ATCC25922) with MIC of 6.25 μg mL^−1^ and 0.391 μg mL^−1^, which are similar to that of the control drug of 6.25 μg mL^−1^ and 0.391 μg mL^−1^, respectively. Compound 7 with a benzoyl group on piperazine and a propargyl moiety at the carboxylic side showed reasonably good activity against *A. baumannii* (clinical isolate) with an MIC of 12.5 μg mL^−1^, which was 50% more active than the control drug with an MIC of 25 μg mL^−1^. Compound 10 with a propargyl moiety on the carboxylic oxygen of ciprofloxacin showed excellent to moderate antibacterial activity in the largest number of strains except *S. typhi* (clinical isolate) and *P. mirabilis* (clinical isolate).

Compound 10 again showed excellent activity against *S. aureus* (ATCC25923), and it was found to be 32-times more active with an MIC of 0.195 μg mL^−1^ in comparison with the standard drug (MIC 6.25 of μg mL^−1^), whereas against *E. coli* (ATCC25922), *P. aeruginosa* (ATCC27853) and *K. pneumoniae* (clinical isolate) with an MIC of 0.195 μg mL^−1^, it possessed very good antibacterial activity compared to the MIC of the control drug ciprofloxacin of 0.391 μg mL^−1^, 0.781 μg mL^−1^ and 0.391 μg mL^−1^, respectively. Further, *E. faecalis* (ATCC29212) and *A. baumannii* (clinical isolate) showed moderate to good activity with MIC of 0.781 μg mL^−1^ and 12.5 μg mL^−1^, while that of the standard drug was found to be 0.781 μg mL^−1^ and 25 μg mL^−1^, respectively. However, compound 10 was found to be ineffective against *S. typhi* (clinical isolate) and *P. mirabilis* (clinical isolate). Compound 10a containing a 3-Cl,4-F group on the phenyl-substituted triazole ring was found to be active against three strains, *i.e.*, *S. aureus* (ATCC25923) and *E. coli* (ATCC25922) with MIC of 0.195 μg mL^−1^, while in *A. baumannii* (clinical isolate) it showed an MIC of 12.5 μg mL^−1^, which is half that of the standard drug. Furthermore, it showed excellent activity against *S. aureus* (ATCC25923), whereas it displayed moderate to weak activity against the remaining strains. Compound 10b, having a 4-Br phenyl-substituted triazole ring, was found to be 16-times more active against *S. aureus* (ATCC25923) with an MIC of 0.391 μg mL^−1^ in comparison with the standard drug ciprofloxacin (MIC 6.25 μg mL^−1^), while against *E. coli* (ATCC25922), it showed an MIC of ≤0.195 μg mL^−1^, which is half that of the standard drug of 0.391 μg mL^−1^. Compound 10c, having a 4-Cl substituent on the phenyl ring, also showed similar activity against *E. coli* (ATCC25922) as compound 10b. Compound 10c was found to be active against *S. aureus* (ATCC25923) and *A. baumannii* (clinical isolate) with an MIC of 1.56 μg mL^−1^ and 12.5 μg mL^−1^, respectively. Compound 10d, having a 3-Cl-substituted phenyl ring, showed excellent activity against *S. aureus* (ATCC25923) with an MIC of 0.391 μg mL^−1^ in comparison to that of the control drug of 6.25 μg mL^−1^. Against *P. aeruginosa* (ATCC27853) and *A. baumannii* (clinical isolate), it showed MIC values of 0.781 μg mL^−1^ and 12.5 μg mL^−1^, respectively. The control showed an MIC of 0.781 μg mL^−1^ and 25 μg mL^−1^ for these strains, respectively. The same compound was found to be ineffective against other strains. Further, compound 10e with a *p*-trifluoromethoxy-substituted phenyl ring showed activity against only one strain, *S. aureus* (ATCC25923), with an MIC value of 6.25 μg mL^−1^, similar to the MIC of the standard drug. Compound 10f, having a fluoro group on the *ortho* and *para* positions of the phenyl ring, did not show any activity against the tested strains. Similarly, compounds 11(a–d) did not show activity against any of the tested strains, except compound 11a, which has an acetyl group on piperazine together with a 3-Cl,4-F phenyl-substituted triazole ring, showing moderate activity against *A. baumannii* (clinical isolate) with an MIC of 12.5 μg mL^−1^. In contrast, the control drug showed an MIC of 25 μg mL^−1^. Similarly, compounds 11e and 11f, having 4-OCF_3_ and 2,4-di fluoro groups, and 12a, having a benzoyl group on piperazine together with a 3-Cl and 4-F phenyl-substituted triazole ring, showed good to moderate activity against *S. aureus* (ATCC25923) with MIC values of 6.25 μg mL^−1^, 0.391 μg mL^−1^, and 12.5 μg mL^−1^, respectively, while that of the control was 6.25 μg mL^−1^. Further, compounds 12b and 12d, again having a benzoyl group on piperazine at carbon 7 (C-7) with 4-Br and 3-Cl, were found to be ineffective against various strains. Compound 12c, having 4-Cl on the phenyl ring, was found to show good to moderate activity against *S. aureus* (ATCC25923) and *S. typhi* (clinical isolate) with MIC values of 1.56 μg mL^−1^ and 6.25 μg mL^−1^, respectively, while that of the control was 6.25 μg mL^−1^, and no activity was found in the other strains. Compound 12e, having 4-OCF_3_, showed excellent activity against *S. aureus* (ATCC25923), *E. coli* (ATCC25922), *S. typhi* (clinical isolate), and *A. baumannii* (clinical isolate) with MIC values of 0.391 μg mL^−1^, ≤0.195 μg mL^−1^, 1.56 μg mL^−1^ and 12.5 μg mL^−1^, while that of the control was 6.25 μg mL^−1^, 0.391 μg mL^−1^, 6.25 μg mL^−1^, and 25 μg mL^−1^ respectively. However, this compound did not show activity against *E. faecalis* (ATCC29212), *S. epidermidis* (clinical isolate), *P. aeruginosa* (ATCC27853), *P. mirabilis* (clinical isolate) and *K. pneumoniae* (clinical isolate). Compound 12f, having a 2,4-difluoro-substituted phenyl ring, did not show activity against any strain except *E. faecalis* (ATCC29212) with an MIC of 0.781 μg mL^−1^, which is the same as that of the control. Thus, according to Table 2 most of the compounds showed excellent activity against *S. aureus* with extremely good MIC values. Thus, this work can be further tuned to obtain useful results, where second-generation synthesis depending on the activity data is required and lead compounds should be used in the SAR experiments.

#### Hemolytic activity

The hemolytic activity of substances occurs through several mechanisms, such as an increase in cell membrane permeability, which results in total cell lysis. It has been noted that the drugs found in oils cause latent damage to the erythrocyte membranes, causing them to lyse and release hemoglobin. This hemolytic activity aims to optimize the percentage of hemolyzed compounds when they interact with red blood cells. This serves as an additional criterion for evaluating the relevance of the incorporated compounds and may also serve as a guide for the development of these compounds as drugs. Following the study reported by Nielson *et al.*,^42^ the hemolytic activities of the synthesized compounds were assessed to determine their toxicity profiles at a stable concentration of 100 μM on human RBCs. The results revealed that these compounds produced 1.17–24.5% hemolysis. Additionally, most of the synthesized compounds showed less than 20% hemolysis except 11d and 12f, whereas, ciprofloxacin, a widely used drug, demonstrated 5.23% hemolysis. Table 2 displays the results of the hemolytic assay of the compounds together with information about their antibacterial activities.

#### Molecular docking studies

**Fig. 5:**
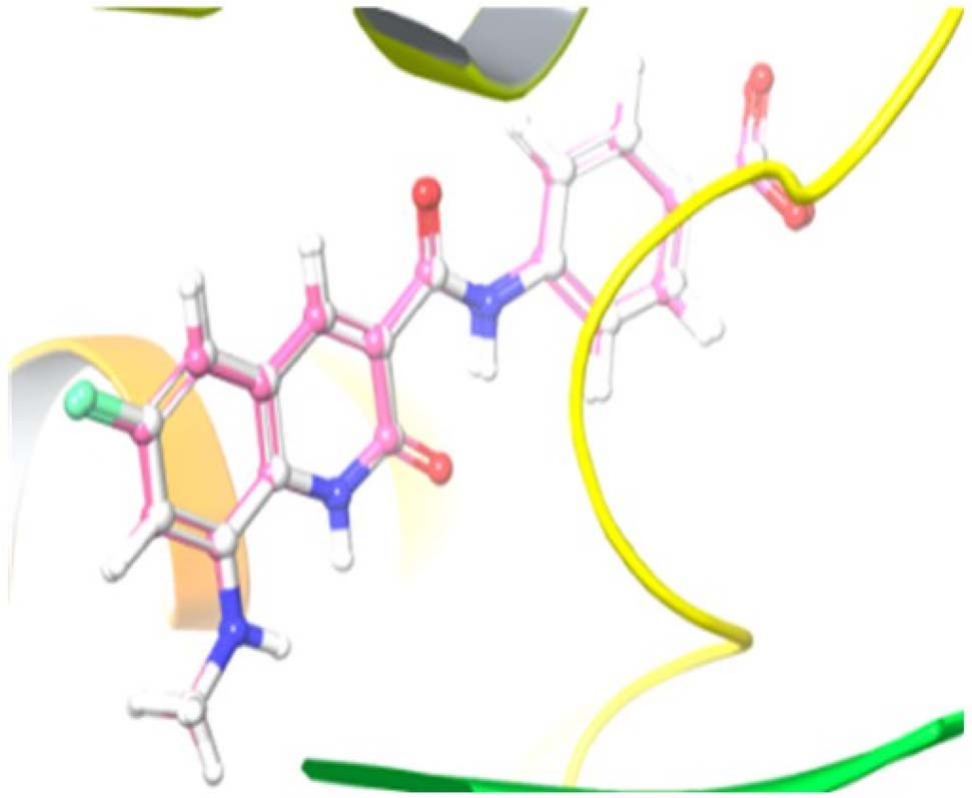
Illustration of the co-crystallized ligand (white) and redocked ligand (pink) for the calculation of RMSD (0.18 Å).

The molecular docking of five selected compounds was performed using the protein structure of *E. coli* DNA gyrase B, which has 6-fluoro-8-(methylamino)-2-*oxo*-1,2-dihydroquinoline as a co-crystallized ligand (PDB: 7C7N).^43^ The structure of the protein was obtained from the Protein Data Bank (“https://www.rcsb.org/”) and further refined using the “Protein Preparation Wizard” in the Schrodinger software. All the selected synthetic compounds were sketched and saved in “sdf or mol” format using Chem Draw software (Chem Draw Professional 15.0). Further, all ligands were prepared using the LigPrep module and the next grid generation was carried out *via* the “Receptor Grid Generation” module. Finally, the Extra Precision Glide (XP) mode was used, where selected compounds were docked against the protein. The co-crystalized ligand was redocked against the protein to validate the docking protocol and compute the root mean square deviation (RMSD), which is 0.18 Å, as shown in Fig. 5.

**Fig. 6:**
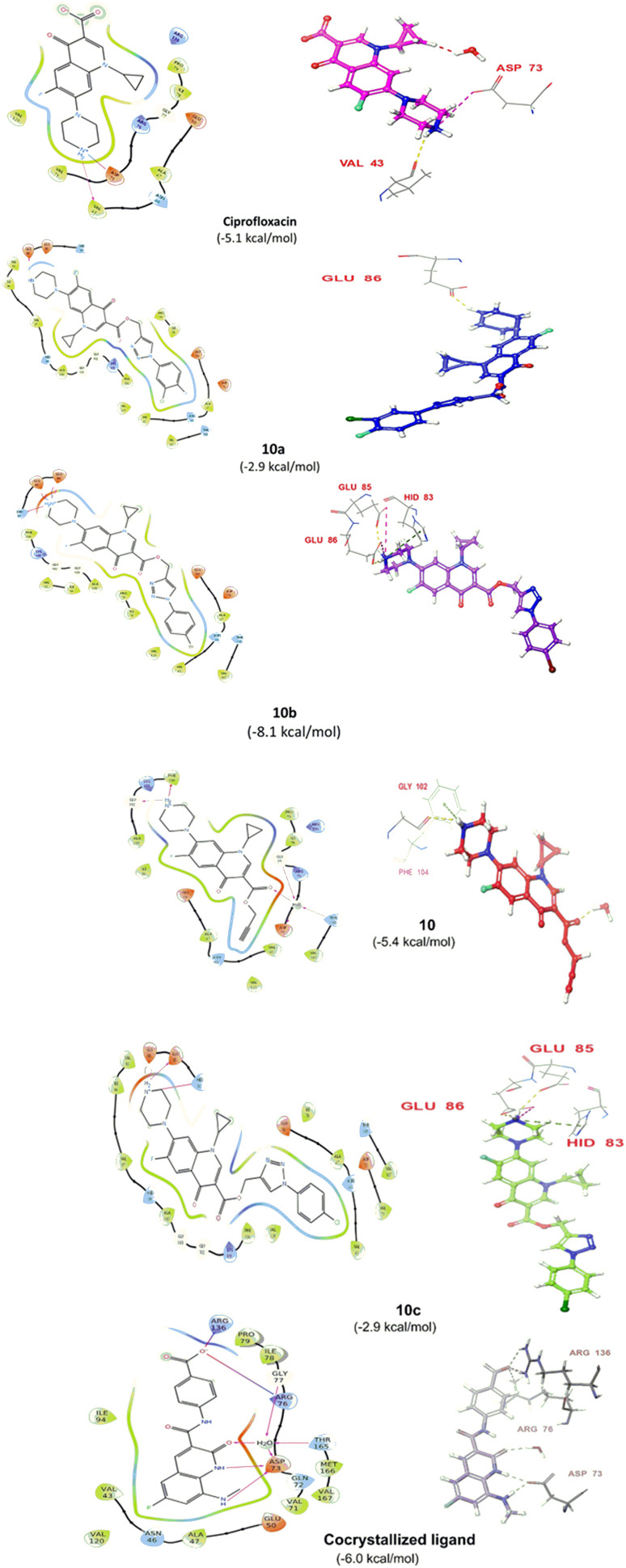
2D and 3D interactions of compounds 10, 10a, 10b and 10c together with the co-crystallized ligand and ciprofloxacin.

Compound 12e did not give any docking score because of its larger size and smaller grid size, and thus we excluded it. Among the compounds, compound 10b showed the highest docking score of −8.1 kcal mol^−1^ and the HIS83, GLU85, and GLU86 amino acids formed hydrogen bond interaction with the ligand. The other compounds showed docking scores in the range of −5.4 to −2.4 kcal mol^−1^. All the docked compounds with their vital amino acid interactions and docking scores are depicted in Fig. 6.

#### Molecular dynamics (MD) analysis

**Fig. 7:**
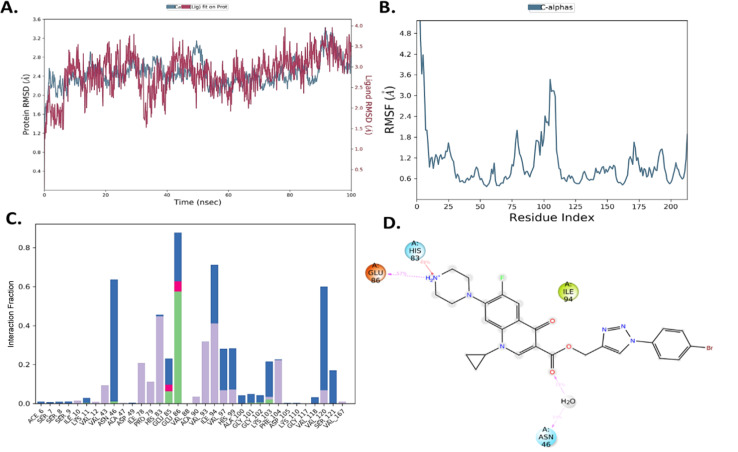
(A) PL complex RMSD of compound 10b. (B) RMSF of the protein. (C) PL-contacts of compound 10b. (D) Ligand–protein contacts of compound 10b.

Compound 10b with the highest docking score was further subjected to MD analysis with *E coli.* DNA gyrase B complex protein for 100 ns. Firstly, in MD, the root mean square deviation diagram (RMSD) of the ligand–protein complex was analyzed. The RMSD graph showed that both the ligand and protein have slight fluctuations in the range of 1.2–3.5 Å, as depicted in Fig. 7A.

However, both ligand–protein complexes remain intact, which describes the ligand remaining in the protein cavity throughout the simulation. Both protein–ligand (PL) complex fluctuations were in the acceptable range, *i.e.* 3 Å. The root mean square fluctuation (RMSF) graph was also plotted to determine the deviations in the protein amino acid residues throughout 100 ns simulation. The RMSF graph showed that the fluctuations of the amino acid residues in the protein backbone occurred near amino acids 100–120 (3.6 Å), as shown in Fig. 7B. The PL contact histogram indicated that GLU86 formed H-bonding with the amine group of piperidine at about 57%, HIS83 was involved in the hydrophobic interaction, particularly the π-cation interaction with the amine group of piperidine, and other amino acid residues such as PRO79, ILE78 and PHE104 involved in the hydrophobic interactions provide stability to the complex. GLU85 is also engaged in hydrogen bonding for a very short time, about 40%, as depicted in Fig. 7(C and D).

#### DNA gyrase expression assay

**Fig. 8:**
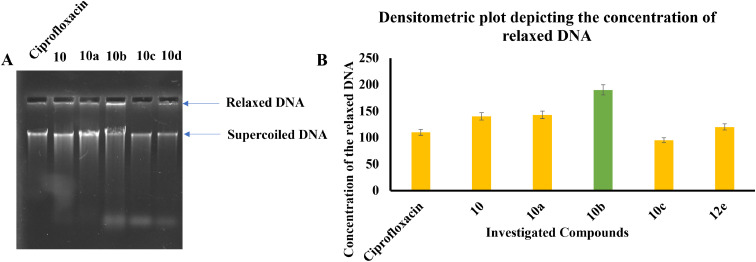
(A) Image of agarose gel depicting the movement and concentration of relaxed and supercoiled DNA upon treatment with the investigated compounds. (B) Densitometry plot illustrating the precise concentration of supercoiled DNA in the untreated sample. A decrease in supercoiled DNA suggests the inhibition of DNA gyrase.

We performed an *in vitro* assay to evaluate the inhibition of DNA gyrase by the investigated compounds 10, 10a, 10b, 10c, and 12e using ciprofloxacin as a positive control. DNA gyrase is responsible for introducing negative supercoils into DNA. In this assay, the migration of supercoiled DNA molecules in agarose gel is compared to that of linear or relaxed DNA. DNA gyrase causes faster-migrating bands to move toward the top of the gel. A decrease in supercoiled DNA concentration or the formation of relaxed DNA indicates the inhibition of DNA gyrase. Our results shown in Fig. 8A and B demonstrate that treatment with 10 μM of the investigated compounds and ciprofloxacin increased the concentration of relaxed DNA (higher concentration high inhibition), respectively. Compound 10b was found to be the most potent inhibitor followed by compound 10. All the compounds except 10c were found to be potent inhibitors of DNA gyrase compared to ciprofloxacin employed as the positive control.

## Experimental

All the chemicals and solvents used in the current study were purchased from E. Merck (India) and Sigma-Aldrich. The reactions during synthesis were monitored *via* thin layer chromatography (TLC) on precoated silica gel 60 F254 (mesh), and the spots were visualized using UV light. Silica gel (60–120 mesh) was employed for column chromatography. The melting points of all the synthesized compounds were determined using the open capillary method and may be uncorrected. The structural assignments of the synthesized products were based on ^1^H NMR, ^13^C NMR, ^19^F NMR, HRMS, IR and single-crystal XRD. NMR data were collected using a 400 MHz, JEOL JNM-ECS spectrometer in DMSO-d_6_ and CDCl_3_ using TMS as the internal standard and Delta software to process the data. In the reported spectral data, abbreviations such as s = singlet, bs = broad singlet, d = doublet, dd = doublet of doublets, t = triplet, and m = multiple are used. Mass data was produced with the use of a Bruker Compass spectrometer. X-ray analysis was performed using a Rigaku XtaLAB Synergy-i single crystal X-ray diffractometer with a CCD detector (HyPix-Bantam) using graphite monochromatized Cu Kα radiation (*λ* = 1.54184 Å).

### Regeneration of free 1-cyclopropyl-6-fluoro-4-*oxo*-7-piperazin-1-ylquinoline-3-carboxylic acid (1)

A clear solution was obtained by dissolving ciprofloxacin hydrochloride (5.0 g, 13.59 mmol) in water (100 mL). This solution was treated with excess 5% aqueous sodium bicarbonate solution, resulting in the formation of a white precipitate.^44^ The precipitate was collected by filtration to obtain hydrochloride-free ciprofloxacin 1. The free ciprofloxacin was sufficiently pure and used as the starting material for Boc protection, acetylation, and benzoylation. White solid; yield 4.5 g (96%); NMR & mp: reported^45^1.

### Boc protection of 1-cyclopropyl-6-fluoro-4-*oxo*-7-piperazin-1-ylquinoline-3-carboxylic acid (2)

Compound 1 (0.5 g, 1.51 mmol) was dissolved in 1 M NaOH (5 mL) and THF (10 mL) was added, followed by the dropwise addition of Boc_2_O (0.36 g, 1.66 mmol), and the reaction mixture was stirred at room temperature for 16 h. The solvent was removed under reduced pressure and the resulting material was diluted with water (10 mL) and neutralized with sat. NH_4_Cl (aq.) solution. The precipitate was collected by vacuum filtration and washed with water to afford the product.^46^

White solid; yield 500 mg (77%); mp: 210–212 °C; ^1^H NMR (400 MHz, CDCl_3_) *δ*: 14.95 (s, 1H, –COOH), 8.78 (s, 1H, Ar–H), 8.05 (d, *J* = 12.9 Hz, 1H, Ar–H), 7.37 (d, *J* = 7.1 Hz, 1H, Ar–H), 3.73–3.62 (m, 4H, 2×-NCH_2_), 3.53 (m, 1H, –NCH), 3.34–3.25 (m, 4H, 2×-NCH_2_), 1.50 (s, 9H, –C(CH_3_)_3_), 1.43–1.37 (m, 2H, –CH_2_), 1.24–1.17 (m, 2H, –CH_2_); ^13^C NMR (101 MHz, CDCl_3_) *δ*: 177.18 (–CO), 166.99 (–CO), 154.80 (d, *J*_C–F_ = 34.2 Hz), 147.58, 145.87 (d, *J*_C–F_ = 10.6 Hz), 139.10, 120.17 (d, *J*_C–F_ = 17.7 Hz), 112.65 (d, *J*_C–F_ = 23.3 Hz), 108.29, 105.07 (d, *J*_C–F_ = 2.1 Hz), 80.44, 77.41, 77.09, 76.78, 49.84, 35.37, 28.48, 8.34.

### Acetylation of 1-cyclopropyl-6-fluoro-4-*oxo*-7-piperazin-1-ylquinoline-3-carboxylic acid (3)

To a solution of compound 1 (0.5 g, 1.3 mmol) in 1,4-dioxane (20 mL) and 2 M NaOH (5 mL) at 0 °C, acetyl chloride (0.12 g, 1.5 mmol) was added dropwise. The reaction mixture was stirred at room temperature for 2 h and 1 M HCl (10 mL) was added to reach pH 1. The separated solid was collected by filtration and purified by column chromatography using a CH_2_Cl_2_ : MeOH (98 : 2) mixture as the eluent.^47^

White solid; yield 499 mg (89%); mp: 270–272 °C; ^1^H NMR (400 MHz, CDCl_3_) *δ*: 14.90 (s, 1H, –COOH), 8.69 (s, 1H, Ar–H), 7.95 (d, *J* = 12.7 Hz, 1H, Ar–H), 7.36 (d, *J* = 6.9 Hz, 1H, Ar–H), 3.87 (t, *J* = 4.4 Hz, 2H, –NCH_2_), 3.70–3.73 (m, 2H, –NCH_2_), 3.57 (m, 1H, –NCH), 3.39–3.40 (m, 2H, –NCH_2_), 3.31 (t, *J* = 4.4 Hz, 2H, –NCH_2_), 2.18 (s, 3H, –CH_3_), 1.37–1.46 (m, 2H, –CH_2_), 1.22–1.29 (m, 2H, –CH_2_); ^13^C NMR (101 MHz, CDCl_3_) *δ*: 176.97 (–COCH_3_), 169.19 (–CO), 166.83, 154.43, 152.76, 147.53, 145.45 (d, *J*_C–F_ = 7.1 Hz), 139.02, 120.13 (d, *J*_C–F_ = 5.1 Hz), 112.44 (d, *J*_C–F_ = 15.15 Hz), 108.07, 105.18, 67.11, 50.16, 49.39, 46.10, 41.06, 35.40, 21.35, 8.29.

### Benzoylation of 1-cyclopropyl-6-fluoro-4-*oxo*-7-piperazin-1-ylquinoline-3-carboxylic acid (4)

To a solution of compound 1 (0.5 g, 1.4 mmol) in 1,4-dioxane (20 mL) and 2 M NaOH (5 mL) at 0 °C, benzoyl chloride (0.31 g, 2.2 mmol) was added dropwise. The reaction mixture was stirred at room temperature for 2 h and 1 M HCl (10 mL) was added to reach pH 1. The separated solid was collected by filtration and purified by column chromatography using a CH_2_Cl_2_ : MeOH (98 : 2) mixture as the eluent.^47^

White solid; yield 450 mg (68.5%); mp: 285–287 °C; ^1^H NMR (400 MHz, CDCl_3_ : CD_3_OD (10% v/v)) *δ*: 8.83 (s, 1H, Ar–H), 8.02 (d, *J* = 12.7 Hz, 1H, Ar–H), 7.58 (m, 1H, Ar–H), 7.47–7.54 (m, 5H, Ar–H), 4.14–4.24 (m, 1H, –NCH), 3.65–4.04 (m, 4H, 2× –NCH_2_), 3.35–3.50 (m, 4H, 2×-NCH_2_), 1.40–1.47 (m, 2H, –CH_2_), 1.19–1.27 (m, 2H, –CH_2_); ^13^C NMR (101 MHz, CDCl_3_ : CD_3_OD (10% v/v)) *δ*: 177.49 (–COAr), 171.74 (–CO), 153.87, 152.95, 148.27, 139.59 (d, *J*_C–F_ = 6.1 Hz), 135.01, 133.98, 132.31, 130.62 (d, *J*_C–F_ = 4.0 Hz), 128.99, 127.21, 112.46 (d, *J*_C–F_ = 11.1 Hz), 107.66, 106.09, 77.97, 77.97, 77.75, 77.54, 77.54, 49.13, 48.99, 48.99, 48.85, 48.71, 48.71, 48.56, 48.42, 48.42, 48.28, 36.20, 8.22.

### General procedure for the propargylation of 7-(4-(*tert*-butoxycarbonyl)piperazin-1-yl)/7-(4-acetylpiperazin-1-yl)/7-(4-benzoylpiperazin-1-yl)-1-cyclopropyl-6-fluoro-4-*oxo*-1,4dihydroquinoline-3-carboxylic acid (5), (6), (7)

Propargylation was performed using a method reported in the literature.^48^ To a solution of compounds 2, 3 and 4 (1 mmol) in *N*,*N*-dimethylformamide (15 mL) in separate round-bottom flasks, 25 mL of NaHCO_3_ solution (1.2 mmol) and (1.2 mmol) of propargyl bromide were added to each flask under vigorous stirring at room temperature. The mixture was allowed to react at 100 °C for 48 h. The progress of the reaction was monitored by TLC. After evaporating the solvent, the residue was purified by column chromatography using a CHCl_3_ : MeOH (98 : 2) mixture as the eluent to obtain the desired propargylated products 5, 6 and 7.

#### Prop-2-yn-1-yl7-(4-(*tert*-butoxycarbonyl)piperazin-1-yl)-1-cyclopropyl-6-fluoro-4-*oxo*-1,4-dihydroquinoline-3-carboxylate (5)

White solid; yield 405 mg (74%); mp: 190–192 °C; ^1^H NMR (400 MHz, CDCl_3_) *δ*: 8.43 (s, 1H, Ar–H), 7.85 (d, *J* = 13.2 Hz, 1H, Ar–H), 7.18 (d, *J* = 7.1 Hz, 1H, Ar–H), 4.83 (d, *J* = 2.3 Hz, 2H, –OCH_2_), 3.58 (t, *J* = 4.8 Hz, 4H, 2×-NCH_2_), 3.35–3.40 (m, 1H, –NCH), 3.14 (t, *J* = 4.9 Hz, 4H, 2×-NCH_2_), 2.43 (t, *J* = 2.5 Hz, 1H, –C≡CH), 1.43 (s, 9H, –C(CH_3_)_3_), 1.17–1.28 (m, 2H, –CH_2_), 1.08–1.10 (m, 2H, –CH_2_); ^13^C NMR (101 MHz, CDCl_3_) *δ*: 171.64 (–CO), 163.36 (–CO), 153.35 (d, *J*_C–F_ = 27.3 Hz), 151.24, 147.41, 143.34 (d, *J*_C–F_ = 8.1 Hz), 136.76, 121.91 (d, *J*_C–F_ = 6.1 Hz), 112.11 (d, *J*_C–F_ = 19.2 Hz), 108.11, 103.99, 79.10, 77.00, 76.18, 75.93, 75.67, 73.66, 50.93, 48.77, 33.55, 28.55, 27.29, 7.03; HRMS (ESI): anal. calcd. For C_25_H_28_FN_3_O_5_, 469.5134 [M]^+^; found 470.2075 [M + H]^+^.

#### Prop-2-yn-1-yl7-(4-acetylpiperazin-1-yl)-1-cyclopropyl-6-fluoro-4-*oxo*-1,4 dihydroquinoline-3-carboxylate (6)

White solid; yield 480 mg (87%); mp: 238–240 °C; ^1^H NMR (400 MHz, CDCl_3_) *δ*: 8.54 (s, 1H, Ar–H), 7.99 (d, *J* = 13.1 Hz, 1H, Ar–H), 7.27 (s, 1H, Ar–H), 4.91 (s, 2H, –OCH_2_), 3.69–3.91 (m, 4H, 2×-NCH_2_), 3.42–3.49 (m, 1H, –NCH), 3.21–3.30 (m, 4H, 2×-NCH_2_), 2.50 (s, 1H, –C≡CH), 2.17 (s, 3H, –CH_3_), 1.31–1.36 (m, 2H, –CH_2_), 1.14–1.18 (m, 2H, –CH_2_); ^13^C NMR (101 MHz, CDCl_3_) *δ*: 172.81 (–COCH_3_), 169.15 (–CO), 164.58, 154.62, 152.15, 148.67, 144.14 (d, *J*_C–F_ = 10.6 Hz), 137.93, 123.41 (d, *J*_C–F_ = 7.1 Hz), 113.47 (d, *J*_C–F_ = 23.1 Hz), 109.40, 105.26, 78.13, 77.41, 77.09, 76.77, 74.86, 52.16, 50.46, 49.57, 46.25, 41.18, 34.71, 21.39, 8.23; ^19^F NMR (565 MHz CDCl_3_) *δ*: −123.65; HRMS (ESI): anal. calcd. For C_22_H_22_FN_3_O_4_,411.4334 [M]^+^; found 412.1651 [M + H]^+^.

#### Prop-2-yn-1-yl7-(4-benzoylpiperazin-1-yl)-1-cyclopropyl-6-fluoro-4-*oxo*-1,4-dihydroquinoline-3-carboxylate (7)

White solid, yield 430 mg (79%); mp: 239–241 °C; ^1^H NMR (400 MHz, CDCl_3_) *δ*: 8.52 (s, 1H, Ar–H), 7.93 (d, *J* = 13.1 Hz, 1H, Ar–H), 7.39–7.45 (m, 5H, Ar–H), 7.27 (d, *J* = 8.0 Hz, 1H, Ar–H), 4.90 (s, 2H, –OCH_2_), 3.59–4.19 (m, 4H, 2×-NCH_2_), 3.46–3.54 (m, 1H, –NCH), 3.10–3.34 (m, 4H, 2×-NCH_2_), 2.50 (s, 1H, –C≡CH), 1.25–1.35 (m, 2H, –CH_2_), 1.12–1.21 (m, 2H, –CH_2_); ^13^C NMR (101 MHz, CDCl_3_) *δ*: 175.61 (–COAr), 172.84 (–CO), 170.66 (–CO), 164.44, 154.19, 152.54, 148.64, 144.14 (d, *J*_C–F_ = 7.1 Hz), 137.93, 135.17, 130.14, 128.67, 127.20, 123.27 (d, *J*_C–F_ = 5.1 Hz), 113.37 (d, *J*_C–F_ = 15.2 Hz), 109.32, 105.34, 78.11, 77.30, 77.09, 76.88, 74.86, 52.13, 50.27, 34.77, 20.81, 8.22; ^19^F NMR (565 MHz CDCl_3_) *δ*: −123.62; HRMS (ESI): anal. calcd. For C_27_H_24_FN_3_O_4_, 473.5044 [M]^+^; found 474.1840 [M + H]^+^.

### General procedure for the synthesis of azides 8(a–f)

Azides were synthesized according to the established method.^49^ Briefly, aniline (1 eq.) was dissolved in 6 N HCl solution (10 mL mmol^−1^ of aniline) at room temperature and cooled to 0 °C, and then NaNO_2_ (1.2 eq.) solution added under stirring. After 10 min, sodium azide (1.2 eq.) was added to the reaction mixture at the same temperature under stirring. Again, this mixture was stirred at room temperature for 2–3 h. Subsequently, the mixture was extracted with ethyl acetate. The organic layer was washed with brine solution and dried over Na_2_SO_4_. After evaporation of the solvent, crude products 8(a–f) were sufficiently pure for further reactions.

### General procedure for the synthesis of 1,2,3-triazole scaffolds 9–12(a–f)

Triazoles were synthesized according to the literature-reported method.^50^ In brief, compounds 5, 6 and 7 (1 eq.) and substituted aromatic azides (a–f, 1.2 eq.) were suspended in a mixture of DMF and water (4 : 1, 25 mL mmol^−1^ of alkyne). Then, a solution of sodium ascorbate (0.4 eq. in minimum water) was added, followed by copper(ii) sulfate pentahydrate solution (0.2 eq. in minimum water). At room temperature, the heterogeneous mixture was rapidly stirred until the consumption of alkyne and the progress of the reaction was monitored by TLC. After completion of the reaction, the reaction mixture was poured into ice water to get the precipitate, which was collected by filtration. The required products were purified by column chromatography using a CHCl_3_ : MeOH (98 : 3) mixture as the eluent.

#### (1-(3-Chloro-4-fluorophenyl)-1*H*-1,2,3-triazol-4-yl)methyl7-(4-(*tert* butoxycarbonyl)piperazin-1-yl)-1-cyclopropyl-6-fluoro-4-*oxo*-1,4-dihydroquinoline-3 carboxylate (9a)

Light yellow solid; yield 126 mg (92%); mp: 208–210 °C; ^1^H NMR (400 MHz, DMSO-d_6_) *δ*: 8.94 (s, 1H, Ar–H), 8.47 (s, 1H, –C=CH of triazole), 8.25 (d, *J* = 4.0 Hz, 1H, Ar–H), 7.98 (d, *J* = 8.7 Hz, 1H, Ar–H), 7.75 (d, *J* = 13.1 Hz, 1H, Ar–H), 7.68 (t, *J* = 8.9 Hz, 1H, Ar–H), 7.46 (d, *J* = 7.3 Hz, 1H, Ar–H), 5.38 (s, 2H, –OCH_2_), 3.64–3.81 (m, 1H, –NCH), 3.54 (s, 4H, 2×-NCH_2_), 3.20 (s, 4H, 2×-NCH_2_), 1.43 (s, 9H, –C(CH_3_)_3_), 1.23–1.28 (m, 2H, –CH_2_), 1.09–1.18 (m, 2H, –CH_2_); ^13^C NMR (101 MHz, DMSO-d_6_) *δ*: 172.60 (–CO), 164.08 (–CO), 161.55, 156.21 (d, *J*_C–F_ = 14 Hz), 148.58, 143.78 (d, *J*_C–F_ = 5 Hz), 138.57 (d, *J*_C–F_ = 5.1 Hz), 135.68, 132.87, 123.12, 122.18 (d, *J*_C–F_ = 7.1 Hz), 121.50, 111.71, 108.52, 106.81, 103.65, 79.20, 56.87, 49.45, 40.00, 39.86, 39.72, 39.58, 39.44, 39.31, 39.17, 28.11, 7.64; HRMS (ESI): anal. calcd. For C_31_H_31_ClF_2_N_6_O_5_,640.2013 [M]^+^; found 641.2063 [M + H]^+^.

#### (1-(4-Bromophenyl)-1*H*-1,2,3-triazol-4-yl)methyl7-(4-(*tert*-butoxycarbonyl)piperazin-1-yl)-1-cyclopropyl-6-fluoro-4-*oxo*-1,4-dihydroquinoline-3-carboxylate (9b)

Brown solid; yield 128 mg (90%); mp: 210–212 °C; ^1^H NMR (400 MHz, DMSO-d_6_) *δ*: 8.57 (s, 1H, Ar–H), 8.32 (s, 1H, –C=CH of triazole), 8.04 (d, *J* = 13.1 Hz, 1H, Ar–H), 7.82 (d, *J* = 8.7 Hz, 2H, Ar–H), 7.38 (d, *J* = 8.7 Hz, 2H, Ar–H), 7.27 (d, *J* = 6.2 Hz, 2H, Ar–H), 5.54 (s, 2H, –OCH_2_), 3.65–3.73 (m, 4H, 2×-NCH_2_), 3.42 (s, 1H, –NCH), 3.22 (s, 4H, 2×-NCH_2_), 1.49 (s, 9H, –C(CH_3_)_3_), 1.23–1.34 (m, 2H, –CH_2_), 1.14–1.18 (m, 2H, –CH_2_); ^13^C NMR (101 MHz, DMSO-d_6_) *δ*: 171.86 (–CO), 164.37 (–CO), 163.81, 154.09 (d, *J*_C–F_ = 9.1 Hz), 148.93 (d, *J*_C–F_ = 13.3), 138.45, 135.66, 133.52, 130.23, 123.46, 122.19, 112.16 (d, *J*_C–F_ = 34.3 Hz), 108.91, 107.19, 79.38, 57.97, 49.82, 40.28, 40.14, 40.00, 39.86, 39.72, 39.58, 39.31, 39.44, 28.39, 7.91; HRMS (ESI): anal. calcd. For C_31_H_32_BrFN_6_O_5_, 666.1602 [M]^+^; found 667.1660 [M + H]^+^.

#### (1-(4-Chlorophenyl)-1*H*-1,2,3-triazol-4-yl)methyl7-(4-(*tert*-butoxycarbonyl)piperazin-1-yl)-1-cyclopropyl-6-fluoro-4-*oxo*-1,4-dihydroquinoline-3-carboxylate (9c)

Off white solid; yield 121 mg (91%); mp: 208–210 °C; ^1^H NMR (400 MHz, DMSO-d_6_) *δ*: 8.57 (s, 1H, Ar–H), 8.32 (s, 1H, –C=CH of triazole), 8.04 (d, *J* = 13.1 Hz, 1H, Ar–H), 7.82 (d, *J* = 8.7 Hz, 2H, Ar–H), 7.38 (d, *J* = 8.7 Hz, 2H, Ar–H), 7.27 (d, *J* = 6.2 Hz, 2H, Ar–H), 5.54 (s, 2H, –OCH_2_), 3.65–3.73 (m, 4H, 2×-NCH_2_), 3.42 (s, 1H, –NCH), 3.22 (s, 4H, 2×-NCH_2_), 1.49 (s, 9H, –C(CH_3_)_3_), 1.23–1.34 (m, 2H, –CH_2_), 1.14–1.18 (m, 2H, –CH_2_); ^13^C NMR (101 MHz, DMSO-d_6_) *δ*: 171.86 (–CO), 164.37 (–CO), 163.81, 154.10 (d, *J*_C–F_ = 9.1 Hz), 148.92 (d, *J*_C–F_ = 13.1 Hz), 138.45, 135.66, 133.52, 130.23, 123.46, 122.19, 112.16 (d, *J*_C–F_ = 34.3 Hz), 111.99, 108.91, 107.19, 79.38, 75.97, 57.19, 49.82, 40.28, 40.14, 40.00, 39.86, 39.72, 39.58, 39.44, 35.20, 28.39, 7.91; HRMS (ESI): anal. calcd. For C_31_H_32_ClFN_6_O_5_, 622.2107 [M]^+^; found 623.2218 [M + H]^+^.

#### (1-(3-Chlorophenyl)-1*H*-1,2,3-triazol-4-yl)methyl7-(4-(*tert*-butoxycarbonyl)piperazin-1-yl)-1-cyclopropyl-6-fluoro-4-*oxo*-1,4-dihydroquinoline-3-carboxylate (9d)

Light pink solid; yield 117 mg (88%); mp: 204–206 °C; ^1^H NMR (400 MHz, DMSO-d_6_) *δ*: 8.98 (s, 1H, Ar–H), 8.48 (s, 1H, –C=CH of triazole), 8.07 (s, 1H, Ar–H), 7.91–7.95 (m, 1H, Ar–H), 7.76 (d, *J* = 13.4 Hz, 1H, Ar–H), 7.64 (t, *J* = 8.2 Hz, 1H, Ar–H), 7.58 (d, *J* = 7.6 Hz, 1H, Ar–H), 7.44–7.47 (m, 1H, Ar–H), 5.39 (s, 2H, –OCH_2_), 3.65–3.82 (m, 1H, –NCH), 3.54 (s, 4H, 2×-NCH_2_), 3.21 (s, 4H, 2×-NCH_2_), 1.43 (s, 9H, –C(CH_3_)_3_), 1.19–1.28 (m, 2H, –CH_2_), 1.09–1.19 (m, 2H, –CH_2_); ^13^C NMR (101 MHz, DMSO-d_6_) *δ*: 171.91 (–CO), 164.42 (–CO), 154.16 (d, *J*_C–F_ = 5.1 Hz), 148.94, 144.13 (d, *J*_C–F_ = 4 Hz), 138.42, 137.97, 134.64, 132.07, 129.02, 123.64, 122.50, 120.37, 119.17, 111.98 (d, *J*_C–F_ = 15.2 Hz), 108.95, 107.16 (d, *J*_C–F_ = 3 Hz), 79.56, 57.20, 49.90, 49.90, 40.36, 40.22, 40.08, 39.94, 39.80, 39.66, 39.53, 35.30, 28.47, 8.00; HRMS (ESI): anal. calcd. For C_31_H_32_ClFN_6_O_5_, 622.2145 [M]^+^; found 623.2218 [M + H]^+^.

#### (1-(4-(Trifluoromethoxy)phenyl)-1*H*-1,2,3-triazol-4-yl)methyl7-(4-(*tert*-butoxycarbonyl)piperazin-1-yl)-1-cyclopropyl-6-fluoro-4-*oxo*-1,4-dihydroquinoline-3-carboxylate (9e)

Light brown solid; yield 116 mg (83%); mp: 218–220 °C; ^1^H NMR (400 MHz, DMSO-d_6_) *δ*: 8.57 (s, 1H, Ar–H), 8.32 (s, 1H, –C=CH of triazole), 8.04 (d, *J* = 13.1 Hz, 1H, Ar–H), 7.82 (d, *J* = 8.7 Hz, 2H, Ar–H), 7.38 (d, *J* = 8.7 Hz, 2H, Ar–H), 7.27 (d, *J* = 6.2 Hz, 2H, Ar–H), 5.54 (s, 2H, –OCH_2_), 3.65–3.73 (m, 4H, 2×-NCH_2_), 3.42 (s, 1H, –NCH), 3.22 (s, 4H, 2×-NCH_2_), 1.49 (s, 9H, –C(CH_3_)_3_), 1.23–1.34 (m, 2H, –CH_2_), 1.14–1.18 (m, 2H, –CH_2_); ^13^C NMR (101 MHz, DMSO-d_6_) *δ*: 171.95 (–CO), 166.33 (–CO), 164.01 (d, *J*_C–F_ = 4 Hz), 154.18 (d, *J*_C–F_ = 8.1 Hz), 152.46, 149.09, 148.48, 144.18 (d, *J*_C–F_ = 10.1 Hz), 139.55, 138.43, 135.82, 123.84, 123.05, 122.61, 112.07, 108.93, 108.51, 107.18, 79.57, 77.88, 57.45, 56.82, 51.85, 49.83 (d, *J*_C–F_ = 18.2 Hz), 40.37, 40.23, 40.09, 39.95, 39.81, 39.67, 39.53, 36.63, 35.35, 28.48, 8.00; HRMS (ESI): anal. calcd. For C_32_H_32_F_4_N_6_O_6_, 672.6376 [M]^+^; found 673.2434 [M + H]^+^.

#### (1-(2,4-Difluorophenyl)-1*H*-1,2,3-triazol-4-yl)methyl7-(4-(*tert*-butoxycarbonyl)piperazin-1-yl)-1-cyclopropyl-6-fluoro-4-*oxo*-1,4-dihydroquinoline-3-carboxylate (9f)

Creamy white solid; yield 97 mg (73%); mp: 180–182 °C; ^1^H NMR (400 MHz, DMSO-d_6_) *δ*: 8.69 (s, 1H, Ar–H), 8.47 (s, 1H, –C=CH of triazole), 7.88–7.96 (m, 1H, Ar–H), 7.68–7.76 (m, 2H, Ar–H), 7.41–7.47 (m, 1H, Ar–H), 7.38 (dd, *J* = 16.8, 9.0 Hz, 1H, Ar–H), 5.40 (s, 2H, –OCH_2_), 3.64 (s, 1H, –NCH), 3.54 (m, 4H, 2×-NCH_2_), 3.20 (m, 4H, 2×-NCH_2_), 1.43 (s, 9H, –C(CH_3_)_3_), 1.23–1.30 (m, 2H, –CH_2_), 1.09–1.19 (m, 2H, –CH_2_); ^13^C NMR (101 MHz, DMSO-d_6_) *δ*: 171.96 (–CO), 164.52 (–CO), 154.21 (d, *J*_C–F_ = 12.5 Hz), 148.97, 144.20 (d, *J*_C–F_ = 10.1 Hz), 143.48, 138.43, 128.11 (d, *J*_C–F_ = 10.5 Hz), 126.75 (d, *J*_C–F_ = 9.8 Hz), 113.21, 113.00, 112.00 (d, *J*_C–F_ = 21.1 Hz), 108.95, 107.14, 106.17, 79.57, 57.25, 49.92, 40.58, 40.37, 40.16, 39.95, 39.75, 39.54, 39.33, 35.31, 29.44, 28.48, 8.00; HRMS (ESI): anal. calcd. For C_31_H_31_F_3_N_6_O_5_, 624.6212 [M]^+^; found 625.2357 [M + H]^+^.

### General procedure for the removal of di-*tert*-butyl dicarbonate (Boc) 10, 10(a–f)

Removal of Boc group was carried out according to the method reported in the literature.^51^ Briefly compounds 5 and 9(a–f) (0.156 mmol) were dissolved in a mixture of trifluoroacetic acid and dichloromethane (1 : 4 v/v, 5 mL) and stirred at room temperature for 24 h, respectively. The reaction mixture was diluted with dichloromethane and washed with saturated aqueous NaHCO_3_ to remove the acid. The organic phase was dried over Na_2_SO_4_, filtered, and evaporated *in vacuo* to obtain the deprotected 1,2,3-triazole scaffolds 10 and 10(a–f) as a powder, respectively.

#### Prop-2-yn-1-yl1-cyclopropyl-6-fluoro-4-*oxo*-7-(piperazin-1-yl)-1,4-dihydroquinoline-3-carboxylate (10)

White solid; yield 66 mg (84%); mp: 178–180 °C; ^1^H NMR (400 MHz, CDCl_3_) *δ*: 8.53 (s, 1H, Ar–H), 7.97 (d, *J* = 13.4 Hz, 1H, Ar–H), 7.26 (t, *J* = 7.1 Hz, 2H, Ar–H), 4.91 (d, *J* = 2.5 Hz, 2H, –OCH_2_), 3.43–3.47 (m, 1H, –NCH), 3.31–3.32 (m, 1H, –NH(CH_2_)_2_), 3.25 (t, *J* = 4.5 Hz, 4H, 2×-NCH_2_), 3.11 (t, *J* = 4.5 Hz, 4H, 2×-NCH_2_), 2.49 (t, *J* = 2.4 Hz, 1H, –C≡CH), 1.29–1.41 (m, 2H, –CH_2_), 1.12–1.20 (m, 2H, –CH_2_); ^13^C NMR (101 MHz, CDCl_3_) *δ*: 172.89 (–CO), 164.62 (–CO), 154.29, 152.64, 148.46, 145.04 (d, *J*_C–F_ = 7.1 Hz), 137.96, 122.78 (d, *J*_C–F_ = 4 Hz), 113.23 (d, *J*_C–F_ = 16.2 Hz), 109.22, 104.80, 78.16, 77.25, 77.04, 76.83, 74.75, 52.07, 51.08, 45.93, 34.64, 8.16; ^19^F NMR (565 MHz CDCl_3_) *δ*: −123.38; IR (KBr) cm^−1^: 3192, 2975, 2864, 1729, 1701, 1621, 1584, 1482, 1423, 1243, 1159, 1078, 1032, 998, 889, 754, 618,549; HRMS (ESI): anal. calcd. For C_20_H_21_FN_3_O_3_, 369.3964 [M]^+^; found 370.1583 [M + H]^+^.

##### (1-(3-Chloro-4-fluorophenyl)-1*H*-1,2,3-triazol-4-yl)methyl1-cyclopropyl-6-fluoro-4-*oxo*-7-(piperazin-1-yl)-1,4-dihydroquinoline-3-carboxylate (10a)

Pale yellow solid; yield 72 mg (85%); mp: 205–207 °C; ^1^H NMR (400 MHz, CDCl_3_) *δ*: 8.48 (s, 1H, Ar–H), 8.28 (s, 1H, –C=CH of triazole), 7.83–7.92 (m, 2H, Ar–H), 7.60 (dd, *J* = 5.2, 3.1 Hz, 1H, Ar–H), 7.19–7.24 (m, 4H, Ar–H), 5.44 (s, 2H, –OCH_2_), 3.31–3.37 (m, 1H, –NCH), 3.20 (m, 4H, 2×-NCH_2_), 3.05 (m, 4H, 2×-NCH_2_), 1.26 (m, 2H, –CH_2_), 1.15–1.20 (m, 2H, –CH_2_), 1.07 (m, 1H, –NH(CH_2_)_2_); ^13^C NMR (101 MHz, CDCl_3_) *δ*: 172.32 (–CO), 163.69 (–CO), 157.92, 155.92, 153.48, 151.51, 147.42, 143.96, 143.53, 137.00, 132.51, 122.09, 121.83, 121.55, 121.40, 119.31 (d, *J*_C–F_ = 6.1 Hz), 116.59 (d, *J*_C–F_ = 19.2 Hz), 112.20 (d, *J*_C–F_ = 19.2 Hz), 108.15, 103.88, 76.28, 76.03, 75.77, 56.76, 44.61, 33.71, 30.96, 28.67, 28.34, 21.68, 13.27, 7.20; ^19^F NMR (565 MHz CDCl_3_) *δ*: −123.27, −114.24; IR (KBr) cm^−1^: 3154, 2972, 2919, 2825, 1723, 1697, 1620, 1469, 1401, 1340, 1246, 1160, 1015, 904, 829, 773, 712, 462; HRMS (ESI): anal. calcd. For C_26_H_23_ClF_2_N_6_O_3_, 540.9558 [M]^+^; found 541.1583 [M + H]^+^.

##### (1-(4-Bromophenyl)-1*H*-1,2,3-triazol-4-yl)methyl1-cyclopropyl-6-fluoro-4-*oxo*-7-(piperazin-1-yl)-1,4-dihydroquinoline-3-carboxylate (10b)

Green solid; yield 70 mg (82%); mp: 218–220 °C; ^1^H NMR (400 MHz, CDCl_3_) *δ*: 8.52 (s, 1H, Ar–H), 8.31 (s, 1H, –C=CH of triazole), 7.92–8.00 (m, 1H, Ar–H), 7.60–7.65 (m, 4H, Ar–H), 7.24–7.39 (m, 1H, Ar–H), 5.53 (s, 2H, –OCH_2_), 3.42–3.48 (m, 1H, –NCH), 3.21 (s, 4H, 2×-NCH_2_), 3.06 (s, 4H, 2×-NCH_2_), 1.21–1.41 (m, 3H, –CH_2,_ and –NH(CH_2_)_2_), 1.05–1.14 (m, 2H, –CH_2_); ^13^C NMR (101 MHz, CDCl_3_) *δ*: 173.12 (–CO), 164.67 (CO), 152.53, 148.50, 144.65, 137.96, 136.26, 132.84, 122.60 (d, *J*_C–F_ = 14.9 Hz), 121.97, 113.25 (d, *J*_C–F_ = 20.0 Hz), 109.18 (d, *J*_C–F_ = 23.23 Hz), 104.71, 77.53, 57.41, 50.74, 45.84, 40.53, 39.90, 39.10, 34.29, 8.18; IR (KBr) cm^−1^: 3147, 2977, 2360, 1724, 1697, 1619, 1489, 1422, 1389, 1341, 1246, 1159, 1077, 1019, 904, 831, 774, 617, 544; HRMS (ESI): anal. calcd. For calculated for C_26_H_24_BrFN_6_O_3_, 566.1077 [M]^+^; found 567.1171 [M + H]^+^.

##### (1-(4-Chlorophenyl)-1*H*-1,2,3-triazol-4-yl)methyl1-cyclopropyl-6-fluoro-4-*oxo*-7-(piperazin-1-yl)-1,4-dihydroquinoline-3-carboxylate (10c)

Light brown solid; yield 80 mg (95%); mp: 215–217 °C; ^1^H NMR (400 MHz, CDCl_3_) *δ*: 8.55 (s, 1H, Ar–H), 8.33 (s, 1H, –C=CH of triazole), 7.99 (d, *J* = 13.3 Hz, 1H, Ar–H), 7.72 (d, *J* = 8.8 Hz, 2H, Ar–H), 7.49 (d, *J* = 8.8 Hz, 2H, Ar–H), 7.26 (d, *J* = 8.2 Hz, 1H, Ar–H), 5.52 (s, 2H, –OCH_2_), 3.43–3.57 (m, 1H, –NCH), 3.25–3.31 (m, 4H, 2×-NCH_2_), 3.11 (s, 4H, 2×-NCH_2_), 1.22–1.41 (m, 3H, –CH_2,_ and –NH(CH_2_)_2_), 1.14–1.22 (m, 2H, –CH_2_); ^13^C NMR (101 MHz, CDCl_3_) *δ*: 173.35 (–CO), 164.64 (–CO), 148.44, 145.16 (d, *J*_C–F_ = 7.5 Hz), 138.07, 135.53, 129.97, 122.73 (d, *J*_C–F_ = 8.2 Hz), 113.29 (d, *J*_C–F_ = 23.5 Hz), 109.31, 104.87, 77.43, 77.12, 76.80, 57.88, 51.13, 45.98, 34.73, 29.77, 8.27; IR (KBr) cm^−1^: 3074, 2973, 1694, 1619, 1476, 1421, 1333, 1245, 1161, 1122, 1028, 892, 831, 800, 775, 703, 703, 623, 544, 464; HRMS (ESI): anal. calcd. For C_26_H_24_ClFN_6_O_3_, 522.9654 [M]^+^; found 523.1685 [M + H]^+^.

##### (1-(3-Chlorophenyl)-1*H*-1,2,3-triazol-4-yl)methyl1-cyclopropyl-6-fluoro-4-*oxo*-7-(piperazin-1-yl)-1,4-dihydroquinoline-3-carboxylate (10d)

White solid; yield 65 mg (77%); mp: 190–192 °C; ^1^H NMR (400 MHz, CDCl_3_) *δ*: 8.55 (s, 1H, Ar–H), 8.35 (s, 1H, –C=CH of triazole), 7.98 (d, *J* = 13.3 Hz, 1H, Ar–H), 7.86 (d, *J* = 14.8 Hz, 1H, Ar–H), 7.63–7.68 (m, 1H, Ar–H), 7.39–7.52 (m, 2H, Ar–H), 7.25–7.28 (m, 1H, Ar–H), 5.52 (s, 2H, –OCH_2_), 3.44 (s, 1H, –NCH), 3.25–3.32 (m, 4H, 2×-NCH_2_), 3.11 (s, 4H, 2×-NCH_2_), 1.25–1.40 (m, 3H, –CH_2,_ and –NH(CH_2_)_2_), 1.14–1.22 (m, 2H, –CH_2_); ^13^C NMR (101 MHz, CDCl_3_) *δ*: 172.60 (–CO), 164.75 (–CO), 152.38, 149.26, 144.52 (d, *J*_C–F_ = 4.5 Hz), 138.59 (d, *J*_C–F_ = 39.3 Hz), 135.19, 132.14, 129.19, 123.81 (d, *J*_C–F_ = 1.8 Hz), 122.28, 120.29, 119.25, 112.49 (d, *J*_C–F_ = 9.0 Hz), 109.01, 106.54, 79.66, 57.61, 51.51, 46.00, 41.24, 40.23, 35.70, 29.81, 8.44; HRMS (ESI): anal. calcd. For C_26_H_24_ClFN_6_O_3_, 522.9654 [M]^+^; found 523.1679 [M + H]^+^.

##### (1-(4-(Trifluoromethoxy)phenyl)-1*H*-1,2,3-triazol-4-yl)methyl1-cyclopropyl-6-fluoro-4-*oxo*-7-(piperazin-1-yl)-1,4-dihydroquinoline-3-carboxylate (10e)

White solid; yield 70 mg (80%); mp: 205–207 °C; ^1^H NMR (400 MHz, CDCl_3_) *δ*: 8.57 (s, 1H, Ar–H), 8.32 (s, 1H, –C=CH of triazole), 8.04 (d, *J* = 13.1 Hz, 1H, Ar–H), 7.82 (d, *J* = 8.7 Hz, 2H, Ar–H), 7.38 (d, *J* = 8.7 Hz, 2H, Ar–H), 7.27 (d, *J* = 6.2 Hz, 1H, Ar–H), 5.54 (s, 2H, –OCH_2_), 3.65–3.73 (m, 4H, 2×-NCH_2_), 3.42 (m, 1H, –NCH), 3.22 (m, 4H, 2×-NCH_2_), 1.23–1.34 (m, 3H, –NH(CH_2_)_2_), 1.14–1.18 (m, 2H, –CH_2_); ^13^CNMR (101 MHz, CDCl_3_) *δ*: 172.43 (–CO), 164.58 (–CO), 152.22, 149.10, 144.35 (d, *J*_C–F_ = 4.5 Hz), 138.42 (d, *J*_C–F_ = 39.3 Hz), 135.03, 131.98, 129.03, 123.64 (d, *J*_C–F_ = 1.8 Hz), 122.12, 120.12, 119.09, 112.33 (d, *J*_C–F_ = 9.0 Hz), 109.31, 104.87, 106.38, 77.43, 77.12, 76.80, 57.88, 51.19 (d, *J*_C–F_ = 29.9 Hz), 45.98, 34.73, 8.27; ^19^F NMR (565 MHz CDCl_3_) *δ*: −123.56, −57.98; HRMS (ESI): anal. calcd. For C_27_H_24_F_4_N_6_O_3_, 572.5206 [M]^+^; found 573.1847 [M + H]^+^.

##### (1-(2,4-Difluorophenyl)-1*H*-1,2,3-triazol-4-yl)methyl1-cyclopropyl-6-fluoro-4-*oxo*-7-(piperazin-1-yl)-1,4-dihydroquinoline-3-carboxylate (10f)

Brown solid; yield 79 mg (94%); mp: 197–199 °C; ^1^H NMR (400 MHz, CDCl_3_) *δ*: 8.56 (s, 1H, Ar–H), 8.31 (s, 1H, –C=CH of triazole), 7.97 (d, *J* = 13.1 Hz, 1H, Ar–H), 7.86 (dd, *J* = 14.0, 8.2 Hz, 1H, Ar–H), 7.28 (d, *J* = 3.6 Hz, 1H, Ar–H), 7.06 (t, *J* = 8.4 Hz, 2H, Ar–H), 5.53 (s, 2H, –OCH_2_), 3.44–3.50 (m, 1H, –NCH), 3.24–3.35 (m, 4H, 2×-NCH_2_), 3.11–3.24 (m, 4H, 2×-NCH_2_), 1.22–1.34 (m, 3H, –NH(CH_2_)_2_), 1.14–1.23 (m, 2H, –CH_2_); ^13^C NMR (101 MHz, CDCl_3_) *δ*: 173.33 (–CO), 164.62 (–CO), 154.30, 152.65, 148.39, 144.96, 143.81, 138, 126.44 (d, *J*_C–F_ = 7.1 Hz), 125.67 (d, *J*_C–F_ = 3 Hz), 122.74, 121.80, 113.18 (d, *J*_C–F_ = 16.2 Hz), 112.51 (d, *J*_C–F_ = 15.2 Hz), 109.16, 105.49 (d, *J*_C–F_ = 13 Hz), 105.23, 104.92, 77.25, 77.04, 76.83, 65.92, 58.22, 57.63, 50.47, 45.55, 34.71, 29.64, 18.43, 15.27, 8.21; ^19^F NMR (565 MHz CDCl_3_) *δ*: −123.51, −121.23, −114.33; HRMS (ESI): anal. calcd. For C_26_H_23_F_3_N_6_O_3_, 524.5042 [M]^+^; found 525.1843 [M + H]^+^.

##### (1-(3-Chloro-4-fluorophenyl)-1*H*-1,2,3-triazol-4-yl)methyl7-(4-acetylpiperazin-1-yl)-1-cyclopropyl-6-fluoro-4-*oxo*-1,4-dihydroquinoline-3-carboxylate (11a)

Pale pink solid; yield 94 mg (95%); mp: 169–171 °C; ^1^H NMR (400 MHz, CDCl_3_) *δ*: 8.57 (s, 1H, Ar–H), 8.32 (s, 1H, –C=CH of triazole), 7.96–8.03 (m, 1H, Ar–H), 7.90 (dd, *J* = 6.2, 2.6 Hz, 1H, Ar–H), 7.65–7.68 (m, 1H, Ar–H), 7.27–7.33 (m, 3H, Ar–H), 5.52 (s, 2H, –OCH_2_), 3.69–3.91 (m, 4H, 2×-NCH_2_), 3.42–3.46 (m, 1H, –NCH), 3.25 (m, 4H, 2×-NCH_2_), 2.17 (s, 3H, –CH_3_), 1.25–1.37 (m, 2H, –CH_2_), 1.13–1.21 (m, 2H, –CH_2_); ^13^C NMR (101 MHz, CDCl_3_) *δ*: 173.30 (–CO), 169.07 (–COCH_3_), 164.59 (–CO), 159.36, 156.6 (d, *J*_C–F_ = 26.3 Hz), 148.48, 144.11 (d, *J*_C–F_ = 26.4 Hz), 137.89, 133.65, 122.89 (d, *J*_C–F_ = 31.8 Hz), 120.22, 117.57 (d, *J* = 23.2 Hz), 113, 109.75, 105.17, 77.33, 77.00, 76.68, 57.72, 49.92, 46.14, 41.08, 34.65, 21.27, 8.18; ^19^F NMR (565 MHz CDCl_3_) *δ*: −123.54, −114.39; HRMS (ESI): anal. calcd. For C_28_H_25_ ClF_2_N_6_O_4_, 582.9928 [M]^+^; found 583.1649 [M + H]^+^.

##### (1-(4-Bromophenyl)-1*H*-1,2,3-triazol-4-yl)methyl7-(4-acetylpiperazin-1-yl)-1-cyclopropyl-6-fluoro-4-*oxo*-1,4-dihydroquinoline-3-carboxylate (11b)

Brick red solid; yield 68 mg (66%); mp: 201–203 °C; ^1^H NMR (400 MHz, CDCl_3_) *δ*: 8.55 (s, 1H, Ar–H), 8.29 (s, 1H, –C=CH of triazole), 8.00 (d, *J* = 13.1 Hz, 1H, Ar–H), 7.62–7.73 (m, 4H, Ar–H), 7.27 (d, *J* = 8.1 Hz, 1H, Ar–H), 5.52 (s, 2H, –OCH_2_), 3.68–3.91 (m, 4H, 2×-NCH_2_), 3.41–3.45 (m, 1H, –NCH), 3.22–3.29 (m, 4H, 2×-NCH_2_), 2.17 (s, 3H, –CH_3_), 1.25–1.34 (m, 2H, –CH_2_), 1.12–1.19 (m, 2H, –CH_2_); ^13^C NMR (101 MHz, CDCl_3_) *δ*: 173.27 (–CO), 169.04 (–COCH_3_), 164.65 (–CO), 154.29, 151.71, 148.43, 144.21 (d, *J*_C–F_ = 18.0 Hz), 137.85, 135.71 (d, *J*_C–F_ = 21.7 Hz), 132.81, 122.14 (d, *J*_C–F_ = 50.0 Hz), 121.89, 113.32 (d, *J*_C–F_ = 23.0 Hz), 109.29, 105.16, 77.32, 77.00, 76.67, 57.70, 50.35, 49.44, 46.12, 41.06, 34.61, 29.63, 21.28, 8.16; HRMS (ESI): anal. calcd. For C_28_H_26_BrFN_6_O_4_, 609.4564 [M]^+^; found 610.1307 [M + H]^+^.

##### (1-(4-Chlorophenyl)-1*H*-1,2,3-triazol-4-yl)methyl7-(4-acetylpiperazin-1-yl)-1-cyclopropyl-6-fluoro-4-*oxo*-1,4-dihydroquinoline-3-carboxylate (11c)

Light brick red solid; yield 69 mg (72%); mp: 197–199 °C; ^1^H NMR (400 MHz, CDCl_3_) *δ*: 8.55 (s, 1H, Ar–H), 8.30 (s, 1H, –C=CH of triazole), 8.01 (d, *J* = 12.2 Hz, 1H, Ar–H), 7.72 (d, *J* = 6.5 Hz, 2H, Ar–H), 7.50 (d, *J* = 6.7 Hz, 2H, Ar–H), 7.28 (s, 1H, Ar–H), 5.52 (s, 2H, –OCH_2_), 3.69–3.84 (m, 4H, 2×-NCH_2_), 3.37–3.43 (m, 1H, –NCH), 3.25 (m, 4H, 2×-NCH_2_), 2.17 (s, 3H, –CH_3_), 1.26–1.40 (m, 2H, –CH_2_), 1.03–1.20 (m, 2H, –CH_2_); ^13^C NMR (101 MHz, CDCl_3_) *δ*: 173.06 (–CO), 169.04 (–COCH_3_), 164.52 (–CO), 155.83, 152.07, 148.44, 144.25 (d, *J*_C–F_ = 9.1 Hz), 137.85, 135.35, 134.51, 129.85, 122.39 (d, *J*_C–F_ = 32.3 Hz), 121.67, 113.35 (d, *J*_C–F_ = 23.4 Hz), 109.43, 105.16, 77.32, 77.00, 76.68, 57.72, 50.37, 49.44, 46.12, 41.06, 34.61, 21.28, 8.17; HRMS (ESI): anal. calcd. For C_28_H_26_ClFN_6_O_4_, 565.0024 [M]^+^; found 566.1810 [M + H]^+^.

##### (1-(3-Chlorophenyl)-1*H*-1,2,3-triazol-4-yl)methyl7-(4-acetylpiperazin-1-yl)-1cyclopropyl-6-fluoro-4-*oxo*-1,4-dihydroquinoline-3-carboxylate (11d)

Light pink solid; yield 70 mg (73%); mp: 205–207 °C; ^1^H NMR (400 MHz, CDCl_3_) *δ*: 8.56 (s, 1H, Ar–H), 8.32 (s, 1H, –C=CH of triazole), 8.01 (d, *J* = 13.1 Hz, 1H, Ar–H), 7.83 (s, 1H, Ar–H), 7.66 (d, *J* = 7.8 Hz, 1H, Ar–H), 7.40–7.48 (m, 2H, Ar–H), 7.27 (d, *J* = 6.3 Hz, 1H, Ar–H), 5.52 (s, 2H, –OCH_2_), 3.68–3.86 (m, 4H, 2×-NCH_2_), 3.41–3.45 (m, 1H, –NCH), 3.22–3.29 (m, 4H, 2×-NCH_2_), 2.17 (s, 3H, –CH_3_), 1.29–1.36 (m, 2H, –CH_2_), 1.14–1.21 (m, 2H, –CH_2_); ^13^C NMR (101 MHz, CDCl_3_) *δ*: 173.05 (–CO), 169.04 (–COCH_3_), 164.54 (–CO), 154.66, 151.92, 148.44, 144.17 (d, *J*_C–F_ = 29.7 Hz), 137.77 (d, *J*_C–F_ = 16.7 Hz), 135.48, 130.76, 128.80, 122.55 (d, *J*_C–F_ = 18.2 Hz), 120.75, 118.44, 113.32 (d, *J*_C–F_ = 23.2 Hz), 109.27, 105.16, 77.32, 77.00, 76.68, 57.70, 50.35, 49.46, 46.12, 41.06, 34.63, 29.63, 21.27, 8.16; HRMS (ESI): anal. calcd. For C_28_H_26_ClFN_6_O_4_, 565.0024 [M]^+^; observed 566.1810 [M + H]^+^.

##### (1-(4-(Trifluoromethoxy)phenyl)-1*H*-1,2,3-triazol-4-yl)methyl7-(4-acetylpiperazin-1-yl)-1-cyclopropyl-6-fluoro-4-*oxo*-1,4-dihydroquinoline-3-carboxylate (11e)

Radish brown solid; yield 61 mg (58%); mp: 220–222 °C; ^1^H NMR (400 MHz, CDCl_3_) *δ*: 8.55 (s, 1H, Ar–H), 8.30 (s, 1H, –C=CH of triazole), 8.01 (d, *J* = 12.2 Hz, 1H, Ar–H), 7.72 (d, *J* = 6.5 Hz, 2H, Ar–H), 7.50 (d, *J* = 6.7 Hz, 2H, Ar–H), 7.28 (s, 1H, Ar–H), 5.52 (s, 2H, –OCH_2_), 3.69–3.84 (m, 4H, 2×-NCH_2_), 3.37–3.43 (m, 1H, –NCH), 3.25 (m, 4H, 2×-NCH_2_), 2.17 (s, 3H, –CH_3_), 1.26–1.40 (m, 2H, –CH_2_), 1.03–1.20 (m, 2H, –CH_2_); ^13^C NMR (101 MHz, CDCl_3_) *δ*: 169.09 (–CO), 166.35 (–COCH_3_), 164.62 (–CO), 148.62, 144.14 (d, *J*_C–F_ = 31 Hz), 137.91, 123.19, 113.51 (d, *J*_C–F_ = 56.6 Hz), 109.48, 105.14, 78.06, 77.33, 77.01, 76.69, 74.78, 52.13 (d, *J*_C–F_ = 22.2 Hz), 50.97, 49.50, 46.20, 41.13, 34.62, 29.68, 21.31, 8.18; ^19^F NMR (565 MHz CDCl_3_) *δ*: −123.56, −57.98 HRMS (ESI): anal. calcd. For C_29_H_26_F_4_N_6_O_5_, 614.5576 [M]^+^; found 615.1995 [M + H]^+^.

##### (1-(2,4-Difluorophenyl)-1*H*-1,2,3-triazol-4-yl)methyl7-(4-acetylpiperazin-1-yl)-1-cyclopropyl-6-fluoro-4-*oxo*-1,4-dihydroquinoline-3-carboxylate (11f)

Creamy white solid; yield 34 mg (35%); mp: 219–221 °C; ^1^H NMR (400 MHz, CDCl_3_) *δ*: 8.54 (s, 1H, Ar–H), 8.33 (s, 1H, –C=CH of triazole), 8.00–8.07 (m, 1H, Ar–H), 7.81–7.89 (m, 2H, Ar–H), 7.38 (d, *J* = 8.5 Hz, 2H, Ar–H), 7.27 (d, *J* = 4.8 Hz, 1H, Ar–H), 5.53 (s, 2H, –OCH_2_), 3.68–3.97 (m, 4H, 2× –NCH_2_), 3.43 (s, 1H, –NCH), 3.21–3.29 (m, 4H, 2×-NCH_2_), 2.17 (s, 3H, –CH_3_), 1.29–1.41 (m, 2H, –CH_2_), 1.14–1.20 (m, 2H, –CH_2_); ^13^C NMR (101 MHz, CDCl_3_) *δ*: 173.02 (–CO), 169.05 (–COCH_3_), 164.58 (–CO), 154.98, 152.09, 148.71 (d, *J*_C–F_ = 50.7 Hz), 144.16 (d, *J*_C–F_ = 21.4 Hz), 137.88, 135.43 (d, *J*_C–F_ = 36.4 Hz), 122.68, 122.19, 121.97, 118.93, 113.39 (d, *J*_C–F_ = 22.8 Hz), 109.35, 105.16, 77.32, 77.00, 76.68, 57.75, 50.34, 49.90, 46.13, 41.06, 34.62, 21.29, 8.18; ^19^F NMR (565 MHz CDCl_3_) *δ*: −123.69, −121.30, −118.55; HRMS (ESI): anal. calcd. For C_28_H_25_F_3_N_6_O_4_, 566.5412 [M]^+^; found 567.1976 [M + H]^+^.

##### (1-(3-Chloro-4-fluorophenyl)-1*H*-1,2,3-triazol-4-yl)methyl7-(4-benzoylpiperazin-1-yl)-1-cyclopropyl-6-fluoro-4-*oxo*-1,4-dihydroquinoline-3-carboxylate (12a)

Light green solid; yield 105 mg (96%); mp: 210–212 °C; ^1^H NMR (400 MHz, CDCl_3_) *δ*: 8.56 (s, 1H, Ar–H), 8.30 (s, 1H, –C=CH of triazole), 8.00 (dd, *J* = 12.9, 8.5 Hz, 1H, Ar–H), 7.89 (dd, *J* = 6.2, 2.5 Hz, 1H, Ar–H), 7.63–7.67 (m, 1H, Ar–H), 7.45 (s, 5H, Ar–H), 7.27–7.32 (m, 3H, Ar–H), 5.52 (s, 2H, –OCH_2_), 3.70–4.01 (m, 4H, 2×-NCH_2_), 3.23–3.43 (m, 5H, –NCH and 2×-NCH_2_), 1.33–1.40 (m, 2H, –CH_2_), 1.15–1.22 (m, 2H, –CH_2_); ^13^C NMR (101 MHz, CDCl_3_) *δ*: 173.01 (–CO), 170.50 (–COAr), 164.59 (–CO), 148.49, 144.11 (d, *J*_C–F_ = 31.3 Hz), 143.95, 138.17, 137.89, 135.13, 130.07, 128.61, 127.11, 123.07, 122.90 (d, *J*_C–F_ = 33.0 Hz), 120.30, 117.57 (d, *J*_C–F_ = 22.8 Hz), 113.53, 109.45, 105.22, 77.32, 77.00, 76.68, 57.75, 51.51, 50.54, 49.78, 35.27, 34.65, 8.21; ^19^F NMR (565 MHz CDCl_3_) *δ*: −123.52, −114.37; HRMS (ESI): anal. calcd. For C_33_H_27_ClF_2_N_6_O_4_, 645.0638 [M]^+^; found 646.1860 [M + H]^+^.

##### (1-(4-Bromophenyl)-1*H*-1,2,3-triazol-4-yl)methyl7-(4-benzoylpiperazin-1-yl)-1-cyclopropyl-6-fluoro-4-*oxo*-1,4-dihydroquinoline-3-carboxylate (12b)

Light green solid; yield 74 mg (65.5%); mp: 172–174 °C; ^1^H NMR (400 MHz, CDCl_3_) *δ*: 8.56 (s, 1H, Ar–H), 8.31 (s, 1H, –C=CH of triazole), 7.98 (d, *J* = 12.8 Hz, 1H, Ar–H), 7.65–7.73 (m, 4H, Ar–H), 7.45–7.53 (m, 5H, Ar–H), 7.27–7.33 (m, 2H, Ar–H), 5.55 (s, 2H, –OCH_2_), 3.68–4.13 (m, 4H, 2×-NCH_2_), 3.22–3.43 (m, 5H, –NCH and 2×-NCH_2_), 1.28–1.34 (m, 2H, –CH_2_), 1.14 (m, 2H, –CH_2_); ^13^C NMR (101 MHz, CDCl_3_) *δ*: 172.29 (–CO), 170.48 (–COAr), 164.54 (–CO), 148.45, 144.58 (d, *J*_C–F_ = 52.8 Hz), 135.48 (d, *J*_C–F_ = 70.7 Hz), 132.81, 130.04, 128.58, 127.09, 122.48, 121.90, 113.25, 109.27, 105.22, 77.32, 77.00, 76.67, 57.73, 50.52, 49.46, 34.62, 8.18; HRMS (ESI): anal. calcd. For C_33_H_28_BrFN_6_O_4_, 671.5274 [M]^+^; found 672.1319 [M + H]^+^.

##### (1-(4-Chlorophenyl)-1*H*-1,2,3-triazol-4-yl)methyl7-(4-benzoylpiperazin-1-yl)-1-cyclopropyl-6-fluoro-4-*oxo*-1,4-dihydroquinoline-3-carboxylate (12c)

Light mint solid; yield 75 mg (71%); mp: 168–170 °C; ^1^H NMR (400 MHz, CDCl_3_) *δ*: 8.57 (s, 1H, Ar–H), 8.30 (s, 1H, –C=CH of triazole), 8.03 (d, *J* = 12.9 Hz, 1H, Ar–H), 7.71 (d, *J* = 8.7 Hz, 2H, Ar–H), 7.45–7.53 (m, 7H, Ar–H), 7.28 (d, *J* = 7.8 Hz, 2H, Ar–H), 5.52 (s, 2H, –OCH_2_), 3.69–4.10 (m, 4H, 2×-NCH_2_), 3.42–3.32 (m, 5H, –NCH and 2×-NCH_2_), 1.23–1.36 (m, 2H, –CH_2_), 1.10–1.19 (m, 2H, –CH_2_); ^13^C NMR (101 MHz, CDCl_3_) *δ*: 173.10 (–CO), 170.50 (–COAr), 164.57 (–CO), 155.92, 154.74, 148.45, 144.34, 141.72, 137.88, 136.55, 135.16, 134.34, 129.96 (d, *J*_C–F_ = 19.2 Hz), 128.61, 127.12, 123.36 (d, *J*_C–F_ = 6.1 Hz), 122.55, 121.71, 113.45 (d, *J*_C–F_ = 23.2 Hz), 109.40, 105.22, 77.32, 77.00, 76.68, 57.78, 41.73, 34.62, 32.31, 8.20; HRMS (ESI): anal. calcd. For C_33_H_28_ClFN_6_O_4_, 627.0734 [M]^+^; found 628.1961 [M + H]^+^.

##### (1-(3-Chlorophenyl)-1*H*-1,2,3-triazol-4-yl)methyl7-(4-benzoylpiperazin-1-yl)-1-cyclopropyl-6-fluoro-4-*oxo*-1,4-dihydroquinoline-3-carboxylate (12d)

Light orange solid; yield 98 mg (92%); mp: 189–191 °C; ^1^H NMR (400 MHz, CDCl_3_) *δ*: 8.58 (s, 1H, Ar–H), 8.35 (s, 1H, –C=CH of triazole), 8.03 (d, *J* = 13.1 Hz, 1H, Ar–H), 7.85 (s, 1H, Ar–H), 7.67–7.70 (m, 1H, Ar–H), 7.40–7.50 (m, 7H, Ar–H), 7.29–7.31 (m, 1H, Ar–H), 5.55 (s, 2H, –OCH_2_), 3.71–4.12 (m, 4H, 2×-NCH_2_), 3.43–3.35 (m, 5H, –NCH and 2×-NCH_2_), 1.28–1.38 (m, 2H, –CH_2_), 1.14–1.23 (m, 2H, –CH_2_); ^13^C NMR (101 MHz, CDCl_3_) *δ*: 173.00 (–CO), 170.51 (–COAr), 164.57 (–CO), 154.75, 152.40, 148.49, 144.33, 137.79 (d, *J*_C–F_ = 17.5 Hz), 135.32 (d, *J*_C–F_ = 36.9 Hz), 131.10, 130.38, 129.16, 128.72 (d, *J*_C–F_ = 22.1 Hz), 127.43, 122.92, 121.11, 118.79, 113.38 (d, *J*_C–F_ = 23.0 Hz), 109.32, 105.25, 77.34, 77.02, 76.71, 57.73, 50.60, 49.72, 41.65, 34.65, 29.66, 8.20; HRMS (ESI): anal. calcd. For C_33_H_28_ClFN_6_O_4_, 627.0734 [M]^+^; found 628.1966 [M + H]^+^.

##### (1-(4-(Trifluoromethoxy)phenyl)-1*H*-1,2,3-triazol-4-yl)methyl7-(4-benzoylpiperazin-1-yl)-1-cyclopropyl-6-fluoro-4-*oxo*-1,4-dihydroquinoline-3-carboxylate (12e)

Light peach solid; yield 99 mg (87%); mp: 195–197 °C; ^1^H NMR (400 MHz, CDCl_3_) *δ*: 8.58 (s, 1H, Ar–H), 8.32 (s, 1H, –C=CH of triazole), 7.99–8.07 (m, 1H, Ar–H), 7.81 (d, *J* = 8.9 Hz, 2H, Ar–H), 7.37–7.53 (m, 7H, Ar–H), 7.27–7.29 (m, 2H, Ar–H), 5.54 (s, 2H, –OCH_2_), 3.69–4.16 (m, 4H, 2×-NCH_2_), 3.45–3.22 (m, 5H, –NCH and 2×-NCH_2_), 1.29–1.38 (m, 2H, –CH_2_), 1.14–1.29 (m, 2H, –CH_2_); ^13^C NMR (101 MHz, CDCl_3_) *δ*: 170.68 (–CO), 164.63 (–CO), 159.41, 149.12 (d, *J*_C–F_ = 21.7 Hz), 148.36, 144.29, 137.92, 134.86 (d, *J*_C–F_ = 29.2 Hz), 130.08, 128.62, 127.13, 122.11 (d, *J*_C–F_ = 21.1 Hz), 113.65 (d, *J*_C–F_ = 20.9 Hz), 104.99 (d, *J*_C–F_ = 26.3), 77.31, 77.00, 76.68, 57.49 (d, *J*_C–F_ = 13.6 Hz).49.76, 40.71, 34.62, 8.22; ^19^F NMR (565 MHz CDCl_3_) *δ* −123.21, −57.86; IR (KBr) cm^−1^: 3145, 2975, 2360, 1725, 1619, 1493, 1424, 1388, 1340, 1314, 1252, 1213, 1157, 1077, 1009, 906, 832, 802, 777, 702, 618, 544; HRMS (ESI): anal. calcd. For C_34_H_28_F_4_N_6_O_5_, 676.6286 [M]^+^; found 677.2151 [M + H]^+^.

##### (1-(2,4-Difluorophenyl)-1*H*-1,2,3-triazol-4-yl)methyl7-(4-benzoylpiperazin-1-yl)-1-cyclopropyl-6-fluoro-4-*oxo*-1,4-dihydroquinoline-3-carboxylate (12f)

Creamy white solid; yield 70.5 mg (66.5%); mp: 203–205 °C; ^1^H NMR (400 MHz, CDCl_3_) *δ*: 8.57 (s, 1H, Ar–H), 8.26 (s, 1H, –C=CH of triazole), 8.01–8.06 (m, 1H, Ar–H), 7.82–7.89 (m, 1H, Ar–H), 7.47 (s, 5H, Ar–H), 7.28 (d, *J* = 7.7 Hz, 1H, Ar–H), 7.04–7.09 (m, 2H, Ar–H), 5.54 (s, 2H, –OCH_2_), 3.66–4.10 (m, 4H, 2×-NCH_2_), 3.22–3.44 (m, 5H, –NCH and 2×-NCH_2_), 1.25–1.43 (m, 2H, –CH_2_), 1.12–1.23 (m, 2H, –CH_2_); ^13^C NMR (101 MHz, CDCl_3_) *δ*: 172.94 (–CO), 170.46 (–COAr), 164.56 (–CO), 154.41, 148.41, 145.96, 143.44 (d, *J*_C–F_ = 14.14 Hz), 138.04, 135.20 (d, *J*_C–F_ = 18.2 Hz), 130.02, 128.56, 127.08, 126.38, 125.91 (d, *J*_C–F_ = 73.2 Hz), 123.45, 113.32, 112.43 (d, *J*_C–F_ = 15.9 Hz), 109.24, 105.12 (d, *J*_C–F_ = 5.4 Hz), 77.27, 76.95, 76.63, 57.63, 34.56, 8.16; ^19^F NMR (565 MHz CDCl_3_) *δ*: −123.51, −121.23, −114.33; HRMS (ESI): anal. calcd. For C_33_H_27_F_3_N_6_O_4_, 628.6122 [M]^+^; found 629.214 [M + H]^+^.

### X-ray crystallographic analysis

**Fig. 9:**
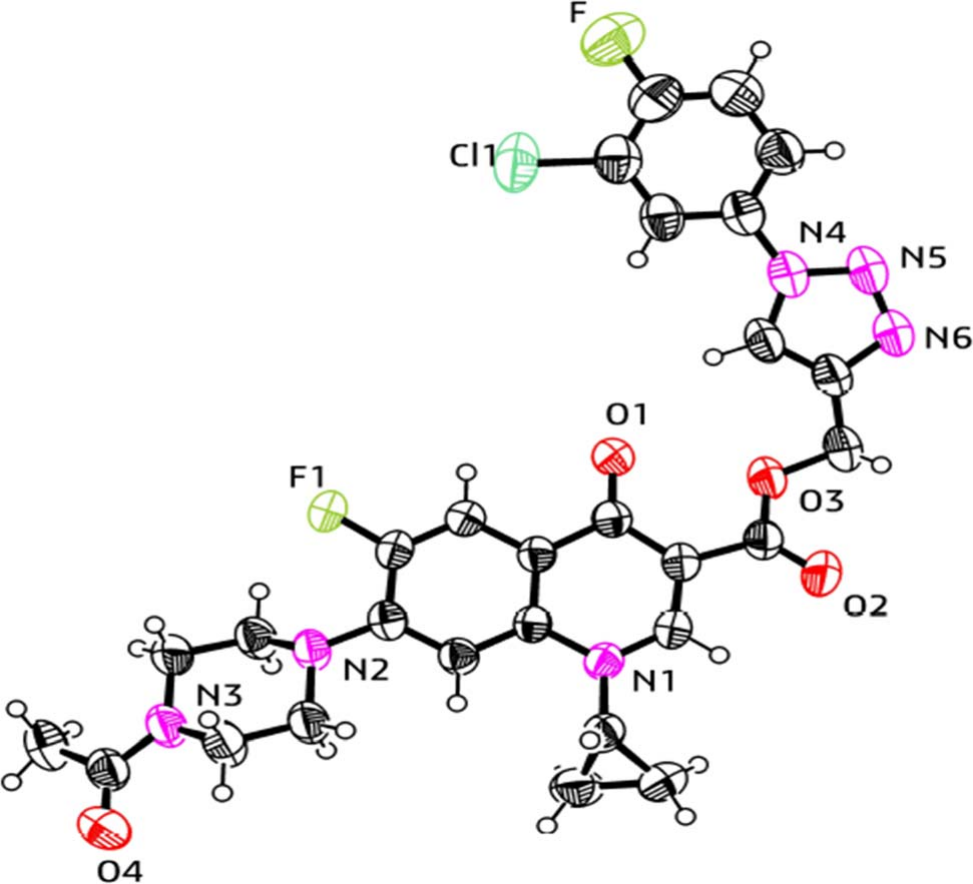
X-ray structure of the 1,2,3-triazole-linked carboxylic group of ciprofloxacin conjugate 11a.

**Fig. 10:**
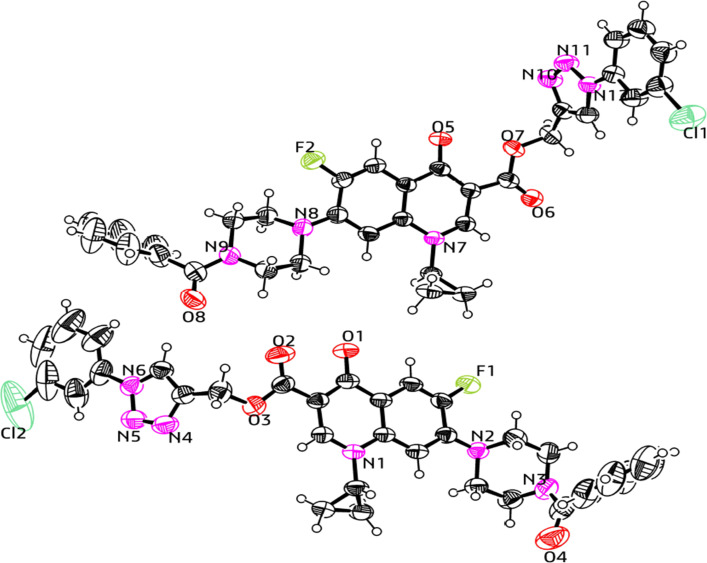
X-ray structure of the 1,2,3-triazole-linked carboxylic group of ciprofloxacin conjugate 12d.

The single-crystal X-ray analysis further verified the structure of the synthesized triazole derivatives 11a and 12d. Briefly, the crystals were formed *via* the slow evaporation solution technique with ethyl alcohol. Graphite monochromatized Cu Kα radiation (*λ* = 1.54184 Å) was used to measure the X-ray diffraction intensity data at 293 K using the X-ray scan method on a Rigaku XtaLAB Synergy-i single-crystal X-ray diffractometer with a CCD-detector (HyPix-Bantam). The structure of compounds 11a and 12d was established by the direct method using the Olex2-1.5 software.^52^ Then, it was refined using the full-matrix least-squares method on F2 by SHELXL.^53^Fig. 9 and 10 show the thermal ellipsoid plot prepared using ORTEP III^54^ of compounds 11a and 12d, which were crystallized in an orthorhombic and triclinic system with the *Pbcn* and *P̄*1 space group, respectively. Table 1 provides information about the single crystal X-ray crystallographic structures of compounds 11a and 12d.

**Table 1:** Crystal data, data collection and structure refinement details for 1,2,3-triazole compounds 11a and 12d

Compound no.	11a	12d
CCDC no.	2268965	2268988
Empirical formula	C_28_H_25_ClF_2_N_6_O_4_	C_33_H_28_ClFN_6_O_4_
Formula weight	582.99	627.06
Temperature	293(2) K	293(2) K
Wavelength	1.54184 Å	1.54184 Å
Crystal system	Orthorhombic	Triclinic
Space group	*Pbcn*	*P̄*1
Hall group	−*P*2*n*2*ab*	−*P*1
Unit cell dimensions	*a* = 22.1700(4) Å, *α* = 90	*a* = 11.7334(2) Å, *α* = 97.2620(10)
	*b* = 7.54141(12) Å, *β* = 90	*b* = 12.3293(2) Å, *β* = 98.652(2)
	*c* = 34.4865(4) Å, *γ* = 90	*c* = 22.1027(4) Å, *γ* = 102.2660(10)
Volume	5765.92(16)	3047.14(9)
*Z*	8	4
Density	1.343 g cm^−3^	1.367 g cm^−3^
Absorption coefficient	1.668 mm^−1^	1.572 mm^−1^
*F*(000)	2416.0	1304.0
Theta range for data collection	5.124 to 136.332	7.438 to 144.282
Index ranges	−25 ≤ *h* ≤ 26	−14 ≤ *h* ≤ 14
	−8 ≤ *k* ≤ 9	−15 ≤ *k* ≤ 14
	−41 ≤ *l* ≤ 41	−27 ≤ *l* ≤ 27
Reflections collected	34 455	58 235
Completeness to theta	99.9%	99.9%
Absorption correction	Multi-scan	Multi-scan
Refinement method	Full-matrix least-squares on *F*2	Full-matrix least-squares on *F*2
Goodness-of-fit on *F*2	1.063	1.069
Final *R* indices [*I* > 2sigma(*I*)]	*R*_1_ = 0.0539, w*R*_2_ = 0.1532	*R*_1_ = 0.0655, w*R*_2_ = 0.1963

**Table 2:** Antibacterial activity (MIC μg mL^−1^) and % hemolysis of compounds 6, 7, 10 and 10–12(a–f)

S. No.	Compound	Gram +ve strains			Gram −ve strains						% Hemolysis
		*E. faecalis* (ATCC 29212)	*S. aureus* (ATCC 25923)	*S. epidermidis* (clinical)	*E. coli* (ATCC 25922)	*P. aeruginosa* (ATCC 27853)	*S. typhi* (clinical)	*P. mirabilis* (clinical)	*A. baumannii* (clinical)	*K. pneumoniae* (clinical)	
6	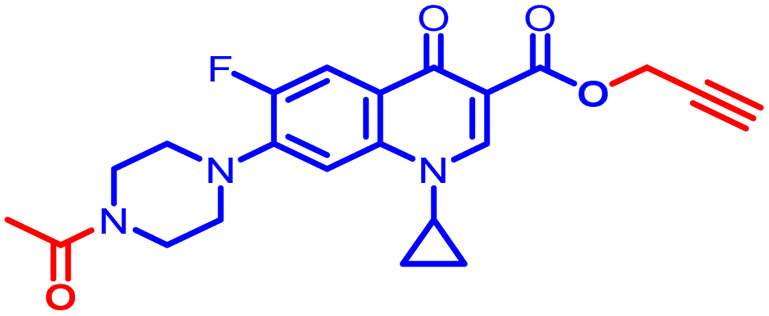	50	**6.25**	100	**0.391**	12.5	100	50	100	12.5	10.20
7	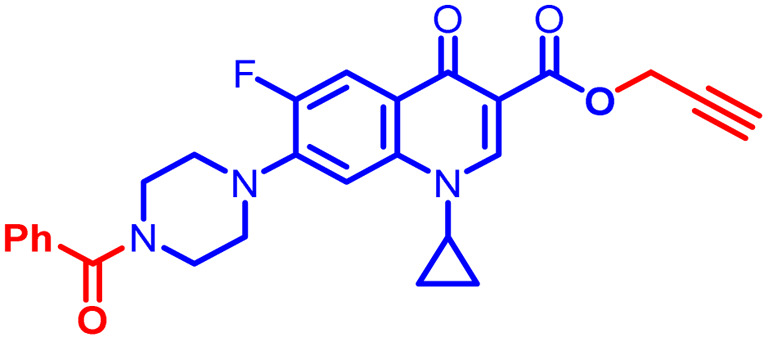	100	100	≥50	3.15	100	50	100	**12.5**	50	6.15
10	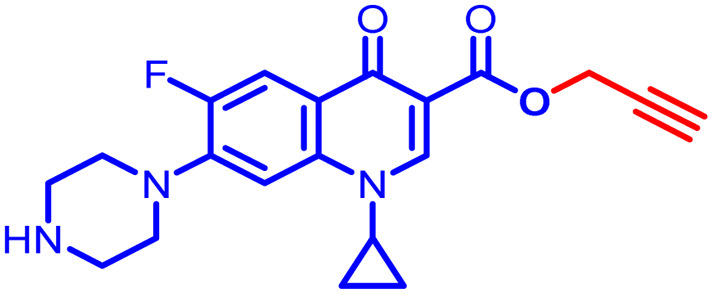	**0.781**	**0.195**	**6.25**	**0.195**	**0.195**	12.5	12.5	**12.5**	**0.195**	3.43
10a	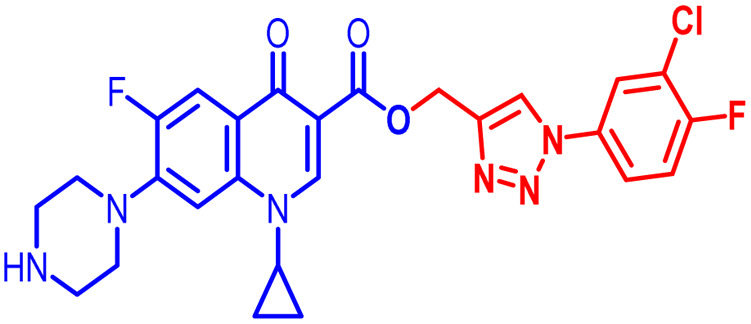	6.25	**0.195**	12.5	**≤0.195**	6.25	25	50	**12.5**	6.25	6.58
10b	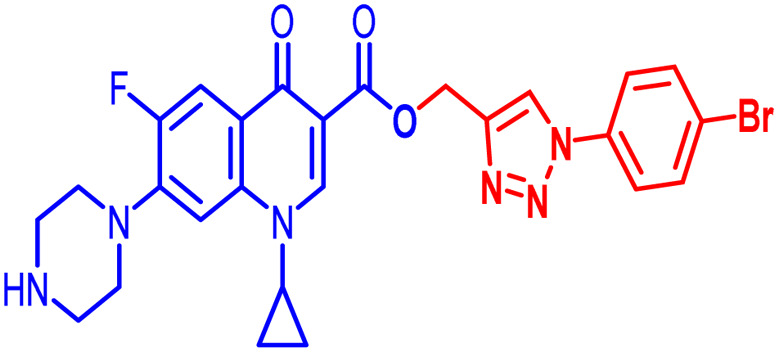	6.25	**0.391**	50	**≤0.195**	3.12	25	50	**12.5**	3.12	4.06
10c	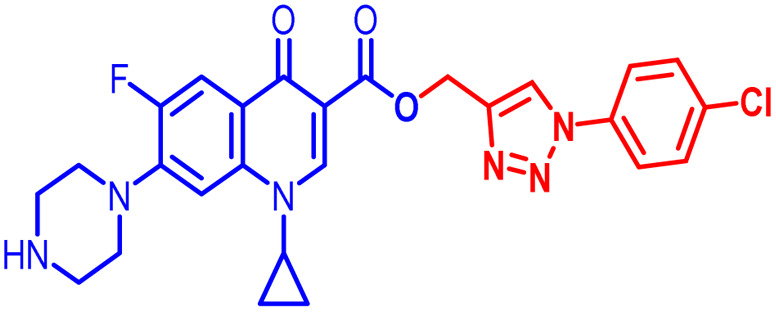	6.25	**1.56**	25	**≤0.195**	6.25	25	50	**12.5**	6.25	1.68
10d	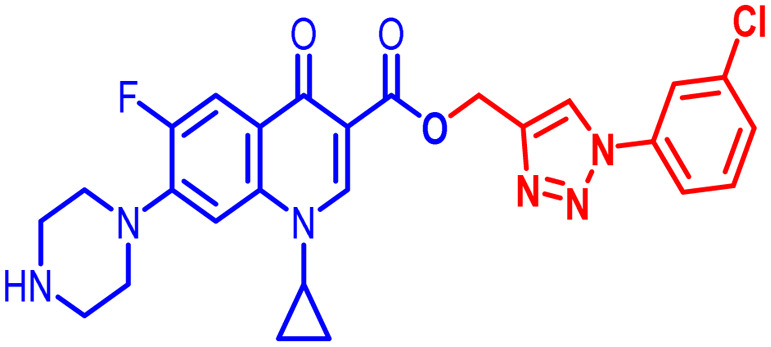	12.5	**0.391**	50	100	**0.781**	25	50	**12.5**	1.56	1.17
10e	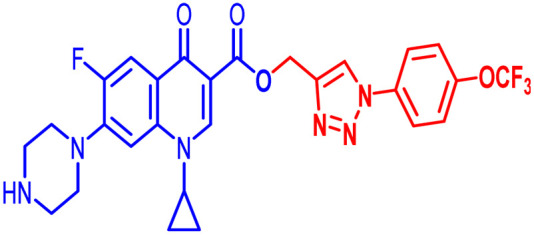	3.12	**6.25**	50	12.5	100	50	25	100	100	7.65
10f	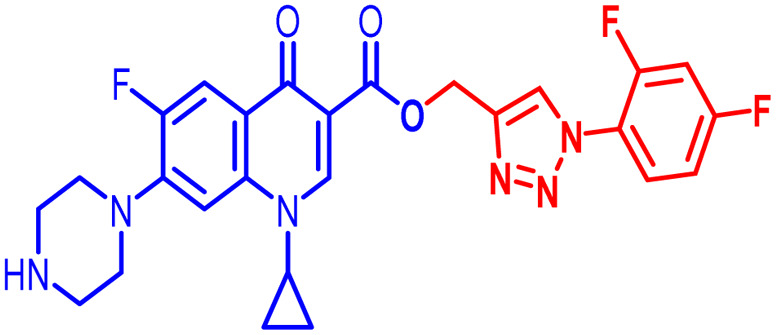	100	12.5	100	25	50	25	25	100	25	7.00
11a	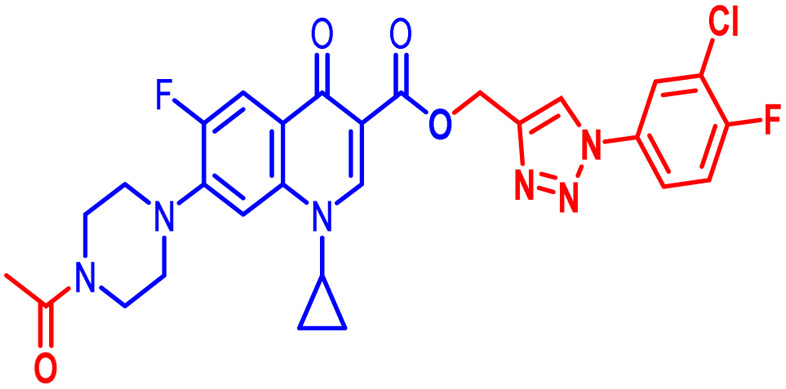	25	100	50	100	100	≥100	100	**12.5**	12.5	5.74
11b	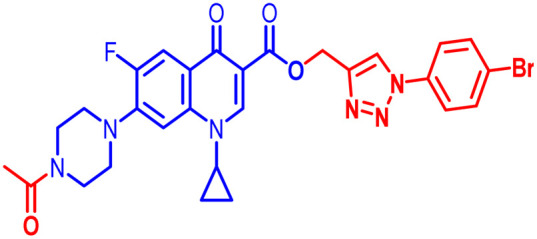	100	100	≥100	100	100	25	100	100	50	2.99
11c	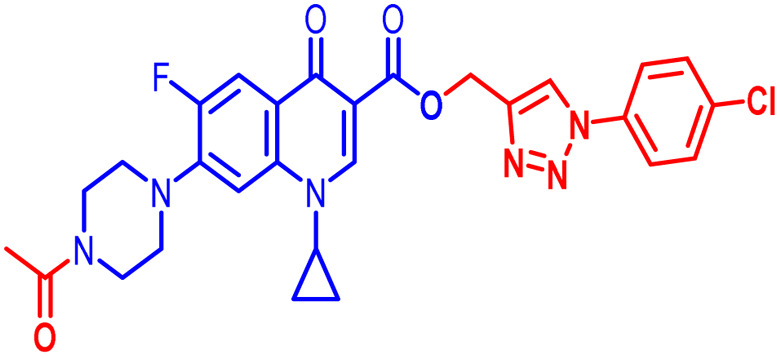	100	100	≥100	100	100	100	100	100	50	7.62
11d	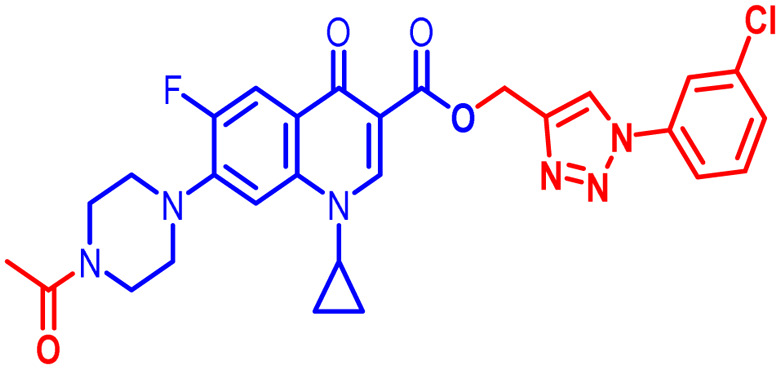	100	100	50	25	50	50	50	50	25	24.50
11e	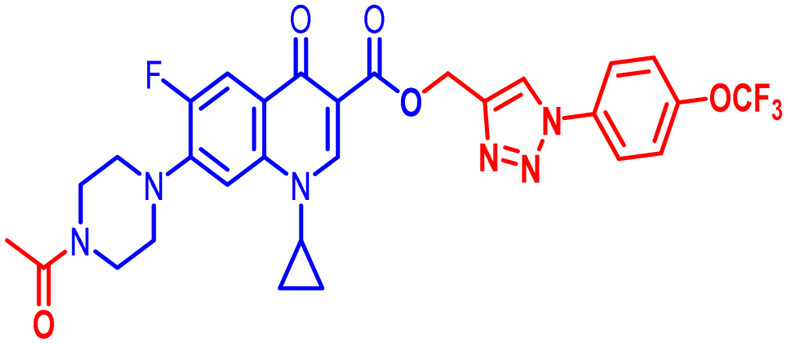	50	**6.25**	≥100	25	50	25	25	50	50	7.09
11f	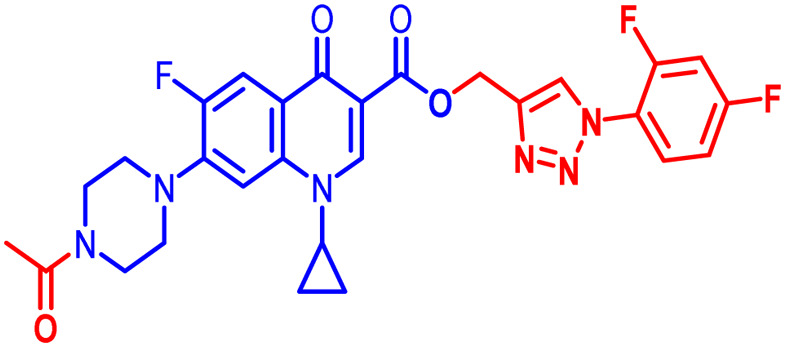	25	**0.391**	50	25	50	50	25	50	50	1.42
12a	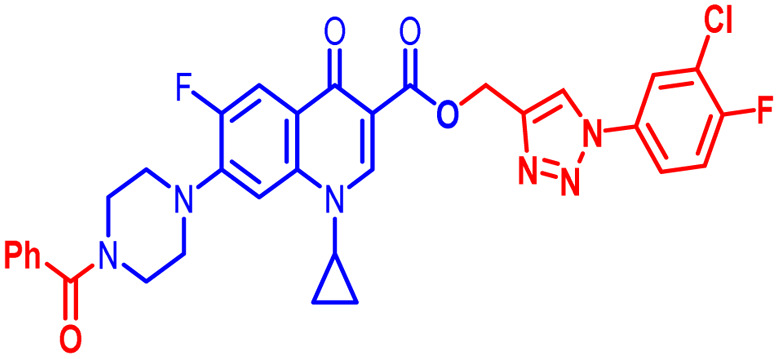	100	12.5	50	100	50	25	100	50	≥ 50	7.23
12b	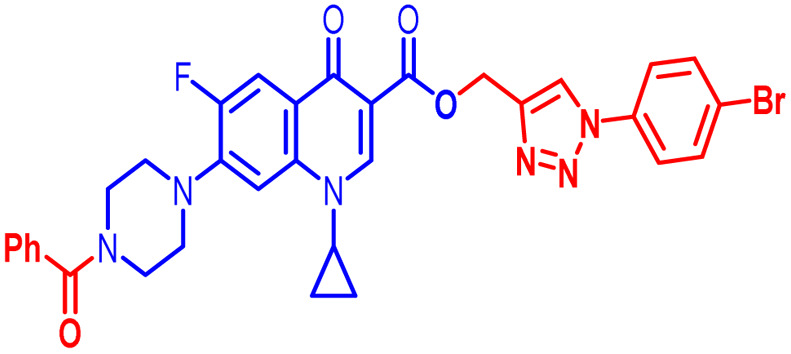	100	25	100	100	100	12.5	100	50	≥ 50	8.06
12c	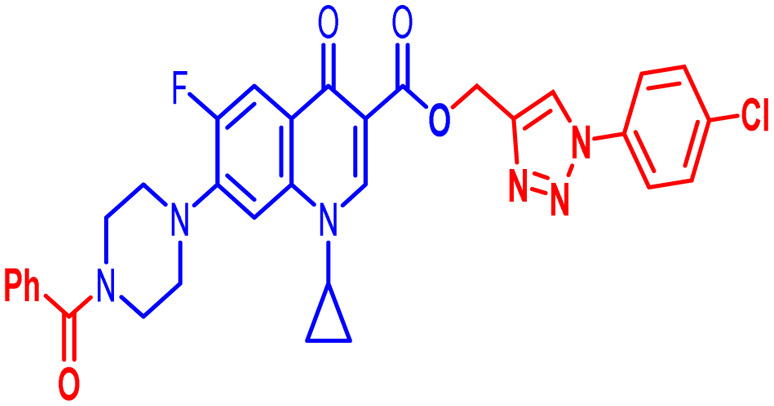	100	**1.56**	100	100	100	**6.25**	50	100	50	6.24
12d	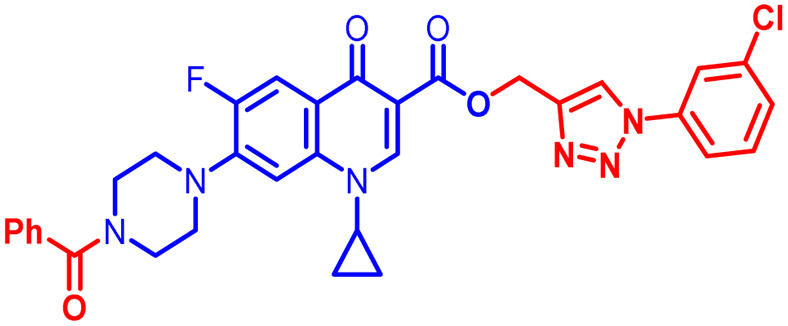	**3.12**	100	>100	12.5	100	50	12.5	100	50	4.80
12e	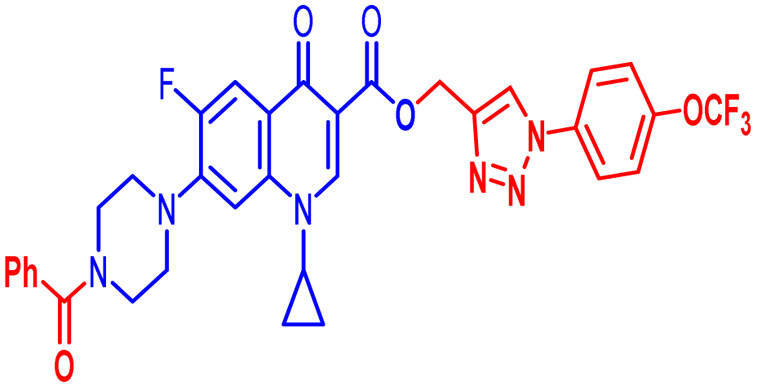	100	**0.391**	50	**≤0.195**	1.56	**1.56**	50	**12.5**	1.56	8.41
12f	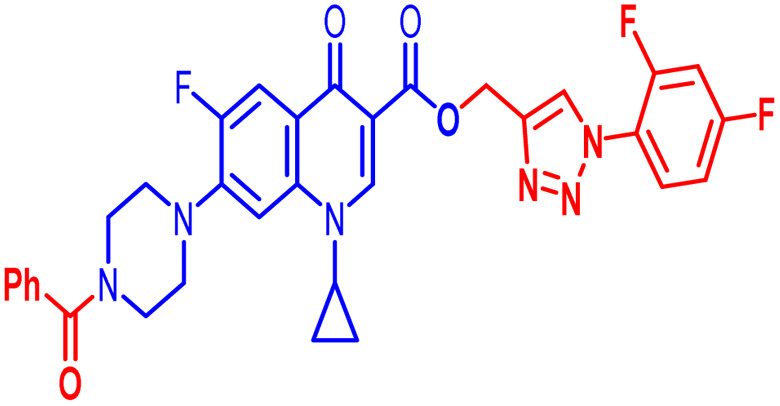	**0.781**	100	≥100	100	100	25	100	100	50	24.29
Standard	Ciprofloxacin	0.781	6.25	0.391	0.391	0.781	6.25	1.56	25	0.391	5.23

### Biological assays

#### Determination of antibacterial activity

The bacterial strains used in the *in vitro* antibacterial investigations included *E. faecalis* (ATCC29212), *S. aureus* (ATCC25923), *S. epidermidis* (clinical isolate), *E. coli* (ATCC25922), *P. aeruginosa* (ATCC27853), *S. typhi* (clinical isolate), *P. mirabilis* (clinical isolate), *A. baumannii* (clinical isolate) and *K. pneumoniae* (clinical isolate). The cultures used in this study were preserved at the Department of Microbiology, Institute of Medical Sciences, Banaras Hindu University, Varanasi, India. All cultures and clinical strains were obtained from the American Type Culture Collection (ATCC). Prior to the screening process, the new microbial broth cultures were prepared in a regular saline solution. The reference medication for evaluating antibacterial effectiveness was ciprofloxacin. The micro-dilution approach was used to calculate the minimum inhibitory concentration (MIC) using a series of dilutions (10-fold) of each chemical.^55^ In a microtiter plate, the various chemical concentrations were serially diluted. In each tube of the microtiter plate, 10 μL of standardized inoculum (1–2 × 10^7^ cfu mL^−1^) was introduced. Subsequently, the plates were incubated for 24 h aerobically at 37 °C. Compared to the wells containing the control, the lowest concentration of the compounds at which they illustrated no sign of bacterial growth and no turbidity in the solution was regarded as the MIC.

#### Determination of the hemolytic activity of compounds on hRBC

The procedure reported by Nielson *et al.* was employed to investigate the hemolytic activity.^56^ Briefly, sterile phosphate-buffered saline (PBS) solution was used to wash fresh human blood three times after it was drawn from a hospital. The cells were centrifuged at 3000 rpm for 7 min at room temperature following each washing and the supernatant was discarded. The RBCs were placed in PBS and concentrated to a final level of 5 × 10^8^ RBCs per mL. To 100 μL of buffer solution containing 100 μM of the test chemicals in 1% v/v dimethyl sulfoxide (DMSO) in PBS, an aliquot (10 μL) of the cell suspension was added. Additionally, the controls included 1% v/v DMSO in PBS and sterile water. The cell suspensions were incubated for 1 h at 37 °C with continuous shaking. After 1 h, the solution was centrifuged at room temperature for 5 min at 1300 rpm, and its absorbance at 540 nm was measured. To calculate the percentage of hemolysis, the UV-vis absorbance values of the tested compounds were calculated as a percentage of the absorbance of sterile water (equal to 100% hemolysis).

#### Molecular docking studies

Furthermore, molecular docking studies of selected potent molecules were carried out. Molecular docking is an *in silico* method for determining the binding affinity between selected ligands and macromolecules such as proteins, RNA, and DNA.^57^ The four main steps used for conducting the molecular docking studies are as follows: (i) protein preparation, which is carried out in the “Protein Preparation wizard”, where all the missing loops and hydrogen are minimized; (ii) ligand preparation, which is done in the LigPrep module; (iii) receptor grid generation, in a square box or grid generated outside the co-crystalized ligand^58^ and (iv) docking of compounds, the final docking step, where selected molecule is docked inside the protein based on its binding affinity and interaction with amino acids, which gives the docking score. All these processes were carried out using the Glide module of the Schrodinger software.^59^

#### Molecular dynamics analysis

MD simulation is performed to understand the conformational deviations and interactions between ligands and proteins throughout the simulation. MD simulation analysis was conducted using the Desmond module in the Schrodinger software.^60^ The protein–ligand complex is initially loaded into the projectable for the MD simulation investigation. After that, the analysis is carried out in three major steps, as follows: (i) system builder: an orthorhombic boundary box encloses the protein–ligand interaction in the system builder panel with the addition of salt and water. The complex solution used the single point charge (SPC) water model and force field and utilized OPLS3. (ii) Energy minimization: the energy minimization ligand–protein complex takes place. (iii) Molecular dynamics: following energy minimization, an MD simulation study was performed for 100 ns using an NPT ensemble at atmospheric pressure of 1.01 bars and temperature (300 K).^61^

#### DNA gyrase inhibition assay

The *E. coli* strain was cultured in 5 mL of LB broth (HI media) in a 15 mL Falcon tube for 24 h at 37 °C. Control strains were grown in LB medium without ciprofloxacin for the experimental setup. At the same time, another tube contained ciprofloxacin (10 mg mL^−1^). In addition, two more tubes were treated with 5c (10 μM) and 5f (10 μM), respectively, and incubated at 37 °C for 24 h. After incubation, the cells were harvested for further DNA extraction. The DNA extraction was performed with the cell pellet obtained from 2.0 mL of 24-hold culture, which was centrifuged at 14 000 rpm for 10 min. The DNeasy Tissue Kit (Qiagen, Cat. No. 69504) was used according to the manufacturer's protocol to recover DNA efficiently. The extracted DNA preparation was quantified by measuring the absorbance at 260 nm. The value of one absorbance at A260 is equivalent to 50 μg mL^−1^ for standard DNA. The integrity of the isolated genomic DNA was determined by 0.8% agarose (Ameresco, USA) gel electrophoresis, which was carried out for 1 h at 75 V against a 1 kb molecular weight marker (Fermentas, USA). The ratio of absorbance at 260 nm and 280 nm was calculated to check the purity of DNA. The genomic DNA was obtained from all treatments using a commercially available kit method and resolved in agarose gel electrophoresis. The results showed that the DNA was intact, and the concentration of all samples was nearly uniform.

## Conclusions

In total, 21 new synthetic ciprofloxacin analogues were tested against nine different antibacterial strains. Five compounds from a pool of a small library, *i.e.*, 10, 10a, 10b, 10c and 12e, showed excellent activity against *E. coli* (ATCC 25922) strain with MIC of ≤0.195 μg mL^−1^, while compound 10 showed excellent activity against *S. aureus* (ATCC 25923), *E. coli* (ATCC 25922), *P. aeruginosa* (ATCC27853) and *K. pneumonia* (clinical isolate) with an MIC of 0.195 μg mL^−1^, respectively. Moreover, four compounds, *i.e.*, 10b, 10d, 11f and 12e, showed excellent activity with an MIC of 0.391 μg mL^−1^ against *S. aureus* (ATCC25923), whereas the control drug, ciprofloxacin, showed an MIC of 6.25 μg mL^−1^. Further, the electron-withdrawing groups such as OCF_3_, F, Cl, and Br in the benzene ring at the *para*-position play a significant role in defining the antibacterial activity. The hemotoxicity testing results showed that all substances had a very low toxicity profile. To create second-generation compounds for antibacterial investigations, it is necessary to analyze the structure–activity relationship (SAR) of potential lead molecules and lower their further effective doses, while increasing their antibacterial action. Subsequently, the lead molecules from the second-generation compounds are coupled with current clinical medications to create multifunctional hybrids. This approach will undoubtedly help reduce the problem of bacterial drug resistance to a certain extent. Moreover, *in silico* studies were performed on the most potent compounds and it was found that compound 10b showed the highest docking score of −8.1 kcal mol^−1^. Thus, due to its highest docking score, it was further subjected to MD analysis with *E. coli.* DNA gyrase B complex protein for 100 ns. Also, the *in vitro* assay indicated that 10b was a more potent inhibitor of DNA gyrase compared to ciprofloxacin employed as the positive control. The single-crystal X-ray analysis of the compounds further confirmed their structure and design criteria. Extensive studies involving a large number of compounds are required to reach any meaningful conclusion in the future.

## Author contributions

Upendra Kumar Patel did the conceptualization, methodology, software, visualization, experimental work and writing the main text. Punit Tiwari did the antibacterial and hemolytic activity under the supervision of Ragini Tilak. Gaurav Joshi and Roshan Kumar did molecular docking studies, molecular dynamic (MD) analysis, DNA gyrase expression assay, software and visualization. Alka Agarwal helped with supervision, reviewing, and editing the original draft. All authors reviewed the manuscript.

## Conflicts of interest

The authors have no conflict of Interest for manuscript publication.

## Supplementary Material

RA-014-D4RA01332H-s001

RA-014-D4RA01332H-s002
