# Supplementary material for: Synthesis of ciprofloxacin-linked 1,2,3-triazole conjugates as potent antibacterial agents using click chemistry: exploring their function as DNA gyrase inhibitors via in silico- and in vitro-based studies
Source: RSC Adv. 2024 May 30;14(24):17051–70. doi: 10.1039/d4ra01332h (PMC11138863; doi:10.1039/d4ra01332h)
Supplement: RA-014-D4RA01332H-s001 [file RA-014-D4RA01332H-s001.pdf]

Electronic Supplementary Material (ESI) for RSC Advances.
This journal is © The Royal Society of Chemistry 2024

Supplementary Data

Synthesis of ciprofloxacin linked 1,2,3-triazole conjugates as potent antibacterial agents using click chemistry: Exploring the mechanistic insights as DNA gyrase inhibitors via *in silico* and *in vitro* based studies

Upendra Kumar Patel, [a] Punit Tiwari, [b] Ragini Tilak, [b] Gaurav Joshi, [c] Roshan Kumar, [d] Alka Agarwal[a]*

[a]Department of Medicinal Chemistry, Institute of Medical Sciences, Banaras Hindu University, Varanasi, UP-221005, India.

[b]Department of Microbiology, Institute of Medical Sciences, Banaras Hindu University, Varanasi, UP-221005, India.

[c]Department of Pharmaceutical Sciences, Hemvati Nandan Bahuguna Garhwal University (Central University) Srinagar – 246174, Dist. Garhwal (Uttarakhand), India

[d]Department of Microbiology, Central University of Punjab, Ghudda, Bathinda-151401, India.

***Corresponding Author**

Prof. Alka Agarwal

Department of Medicinal Chemistry

Institute of medical sciences

Banaras Hindu University

Varanasi, 221005, U.P. India.

Email: agarwal.dralka@gmail.com

LIST OF CONTESTS: page no.

1. General Information 2

2. ^{1}H, ^{13}C, ^{19}F NMR, IR and Mass spectra 3

All the chemicals and solvents used in the current study have been purchased from E. Merck (India) and Sigma-Aldrich. On precoated silica gel 60 F254 (mesh), the reactions during synthesis were monitored via thin layer chromatography (TLC), and spots were visualized using UV light. Silica gel (60–120 mesh) was employed for column chromatography. Melting points of all synthesized compounds were determined by using an open capillary method and may be uncorrected. The structural assignments of the synthesized products were based on ^{1}H NMR, ^{13}C & ^{19}F NMR, HRMS, IR and single-crystal XRD. NMR data were collected using 400 MHz, JEOL JNM-ECS spectrometer in DMSO-d_6 and $CDCl_3$ using TMS as an internal standard and Delta software to process the data. In reporting spectra, abbreviations such as s= singlet, bs = broad singlet, d= doublet, dd= doublet of doublets, t= triplet, and m= multiple were used. Mass data was produced with the use of a Bruker Compass spectrometer. X-ray analysis was performed using Rigaku XtaLAB Synergy-i Single Crystal X-ray Diffractometer with a CCD detector (HyPix-Bantam) using graphite mono-chromatized Cu-Kα radiation (λ = 1.54184 Å).

^{1}H-NMR spectrum of compound **2**

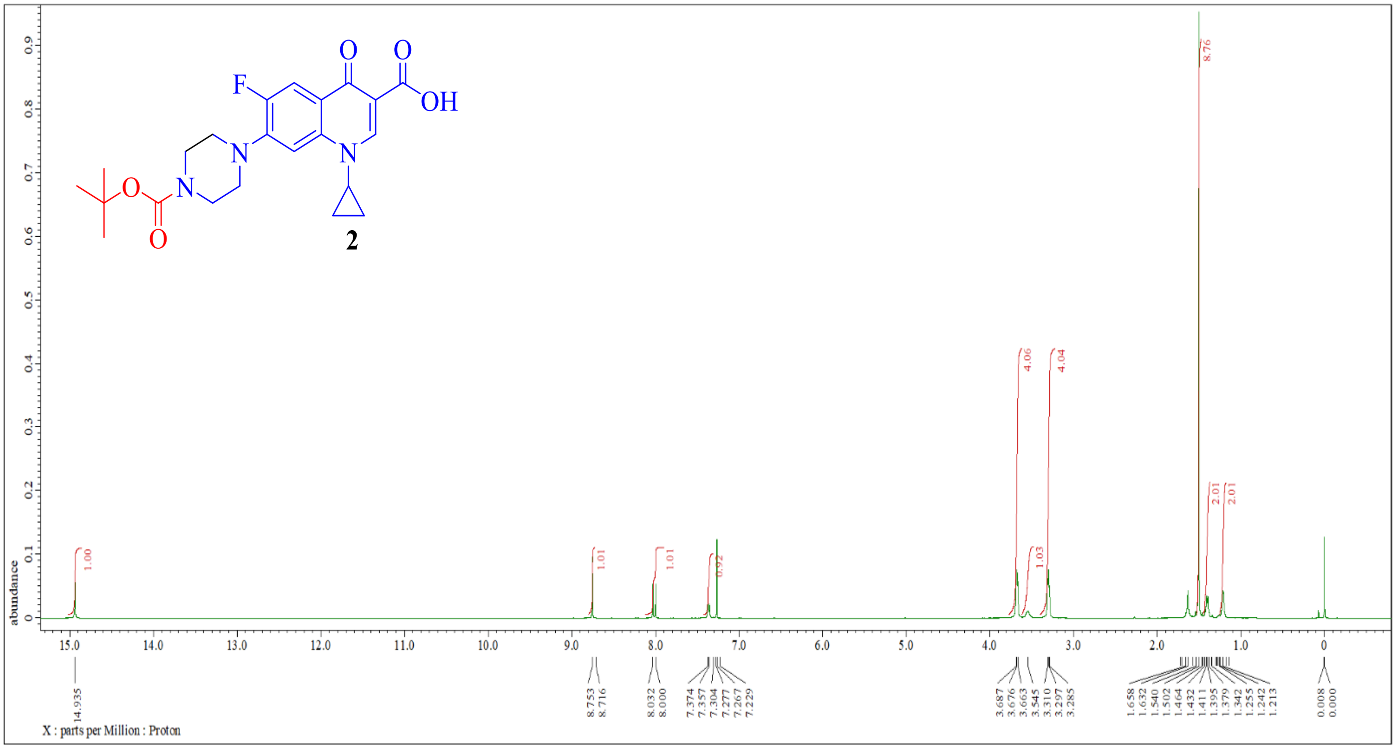

^{13}C-NMR spectrum of compound **2**

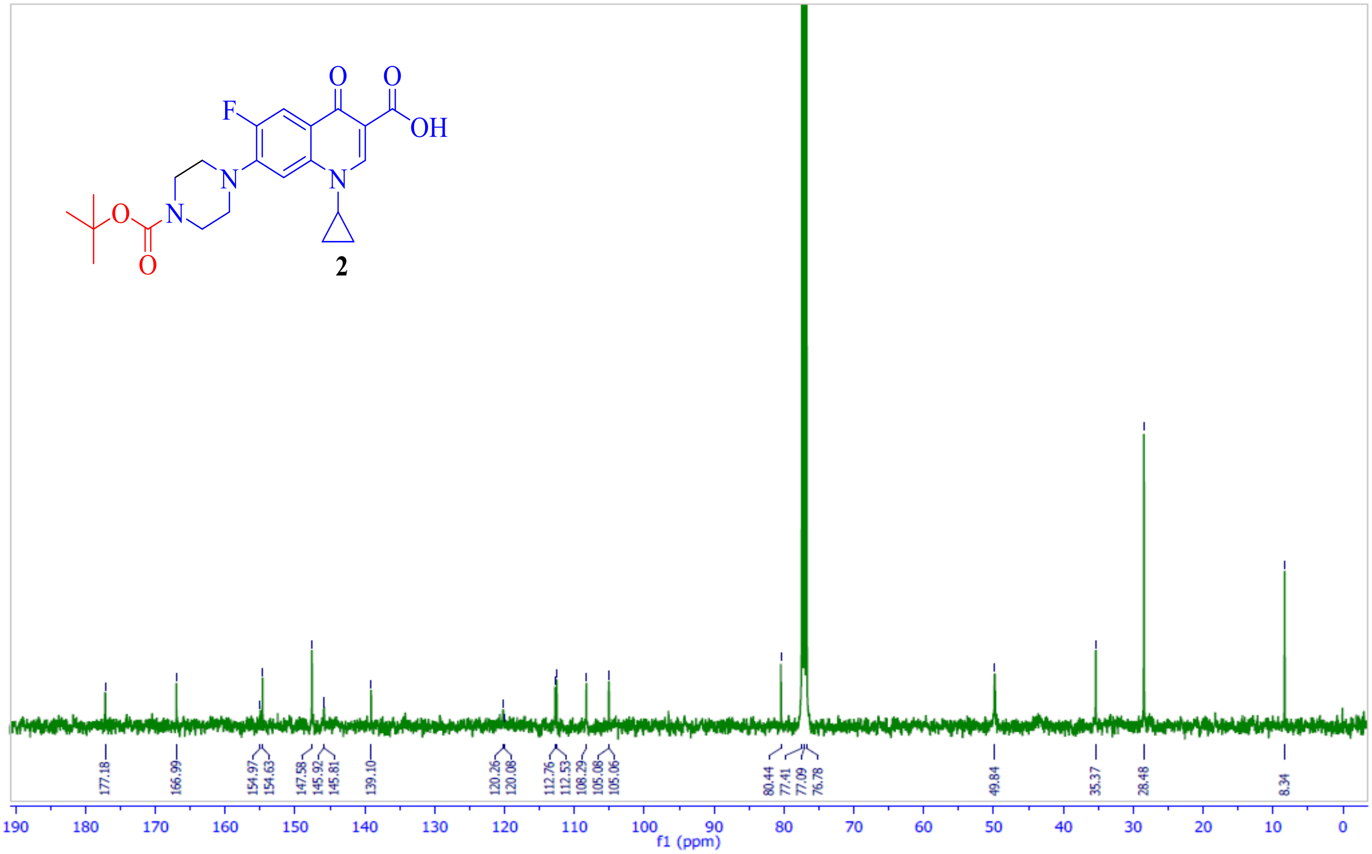

^{1}H-NMR spectrum of compound **3**

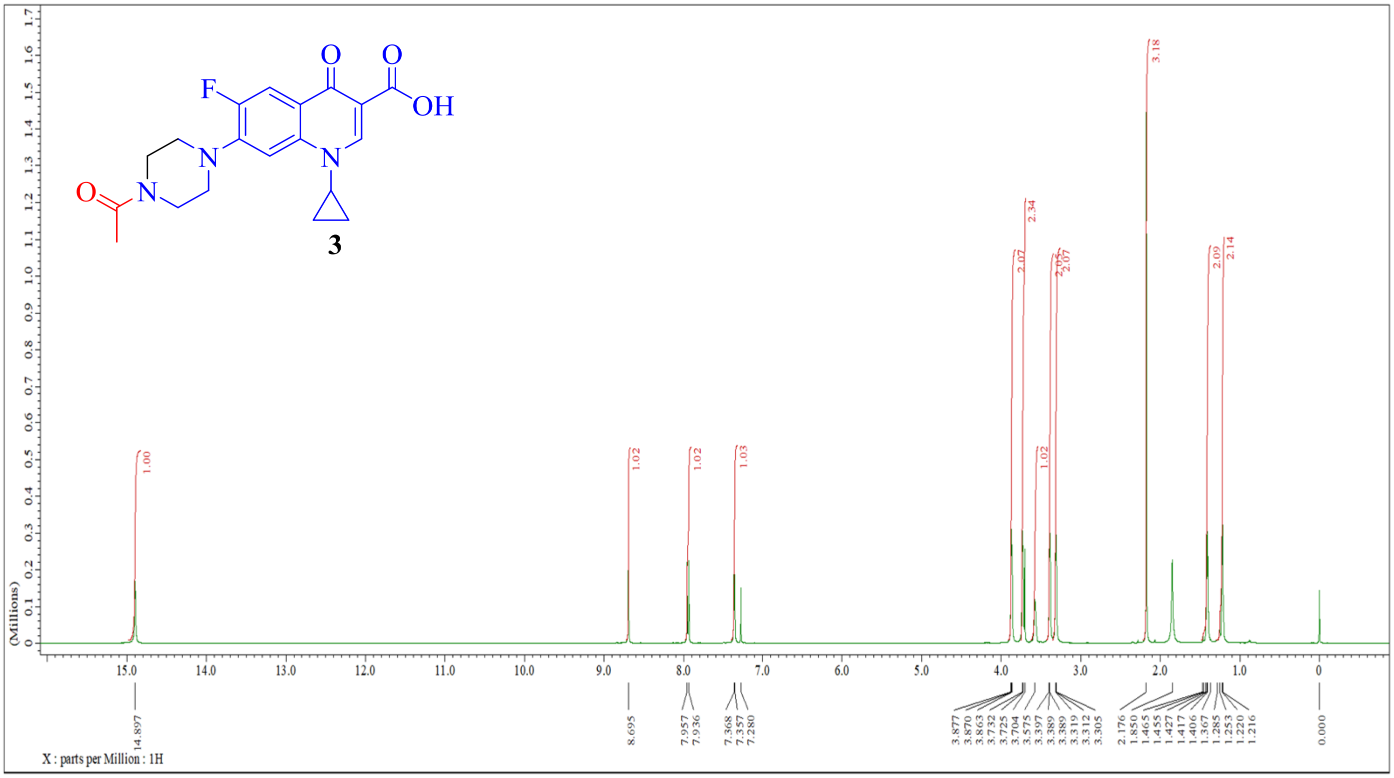

Figure S4. ^{13}C-NMR spectrum of compound **3**

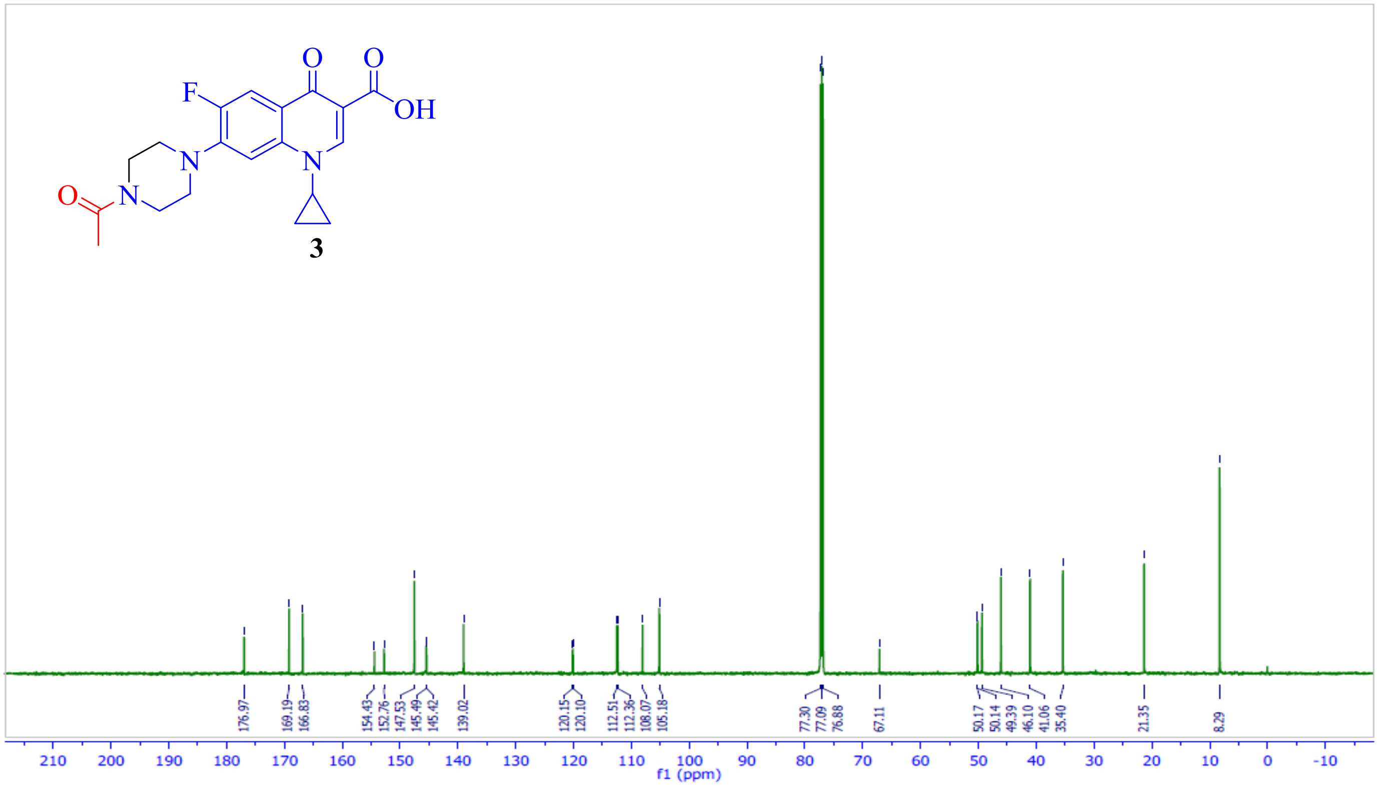

^{1}H-NMR spectrum of compound **4**

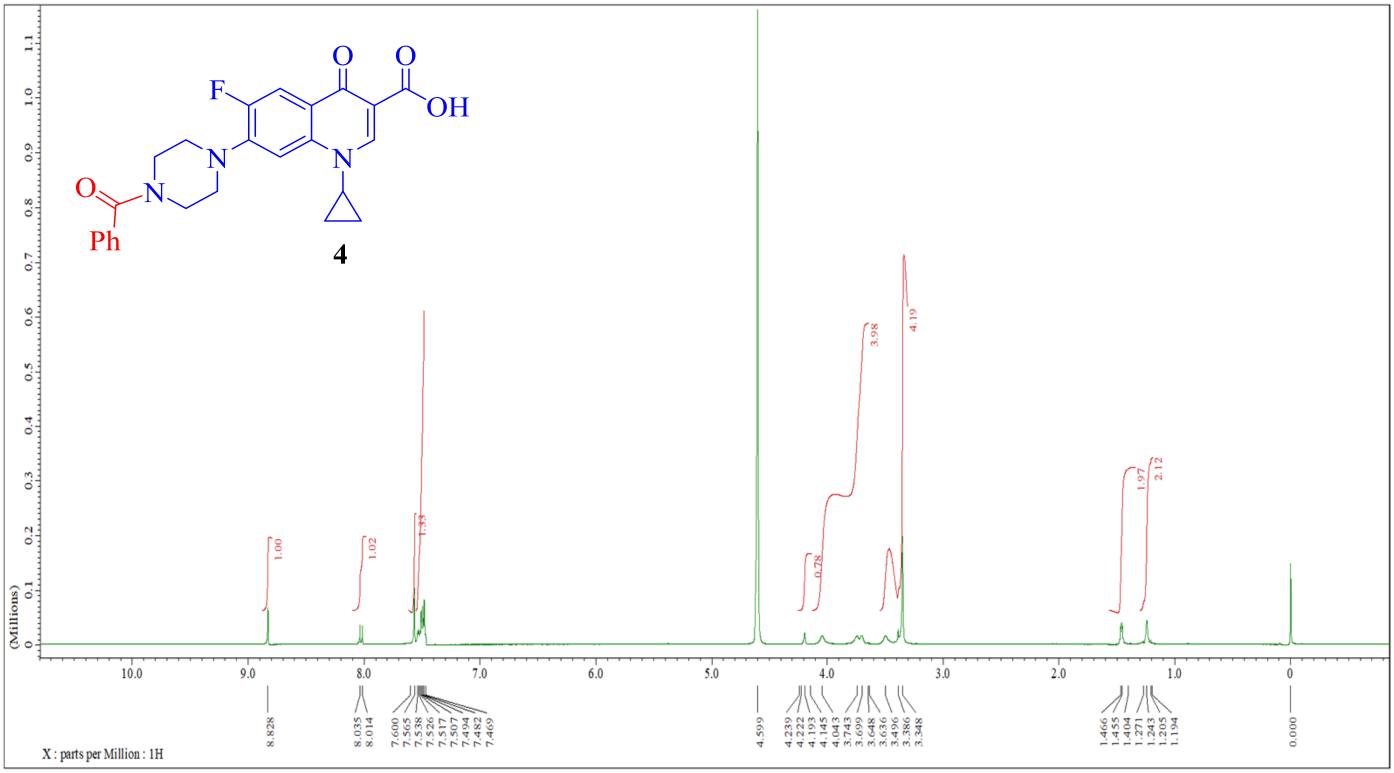

^{13}C-NMR spectrum of compound **4**

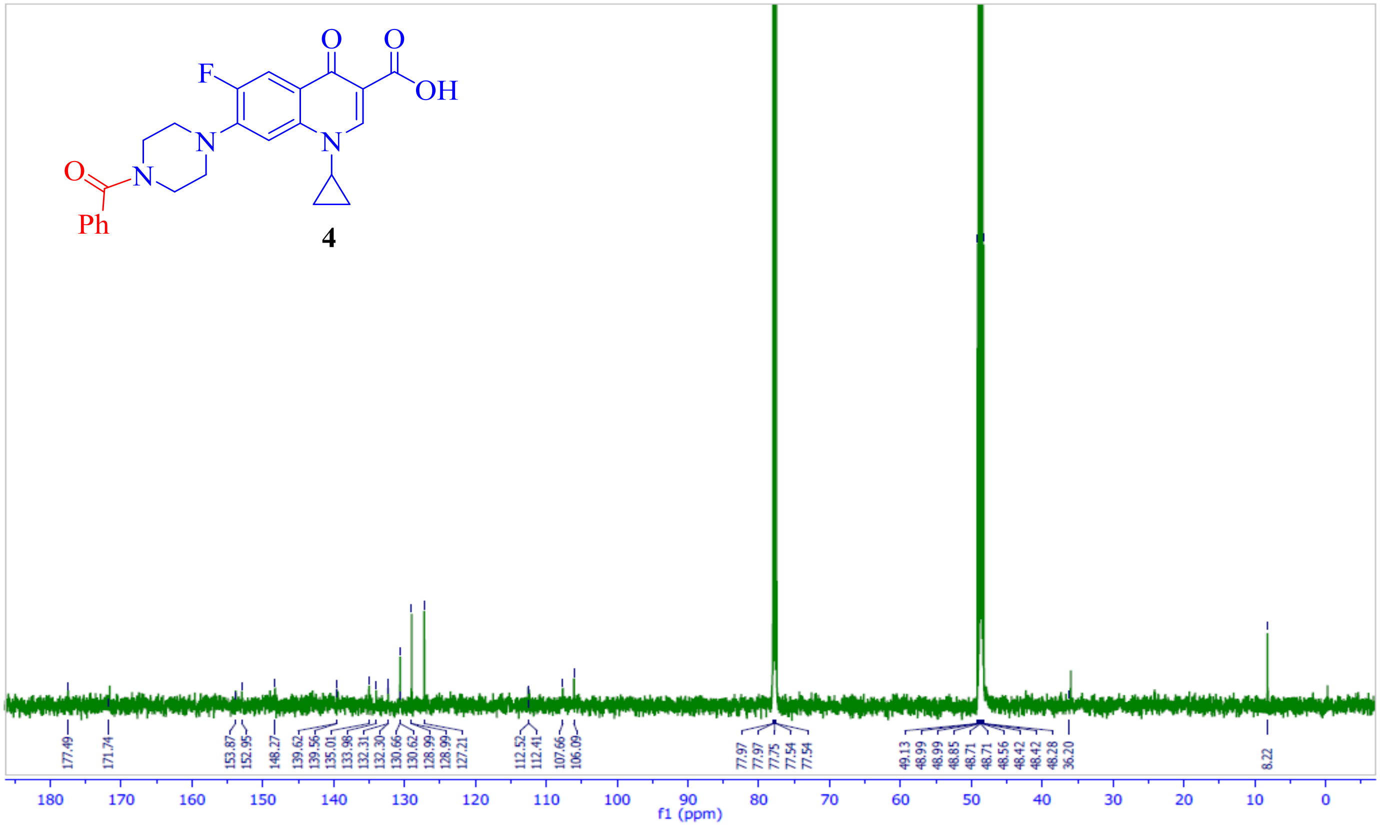

^{1}H-NMR spectrum of compound **5**

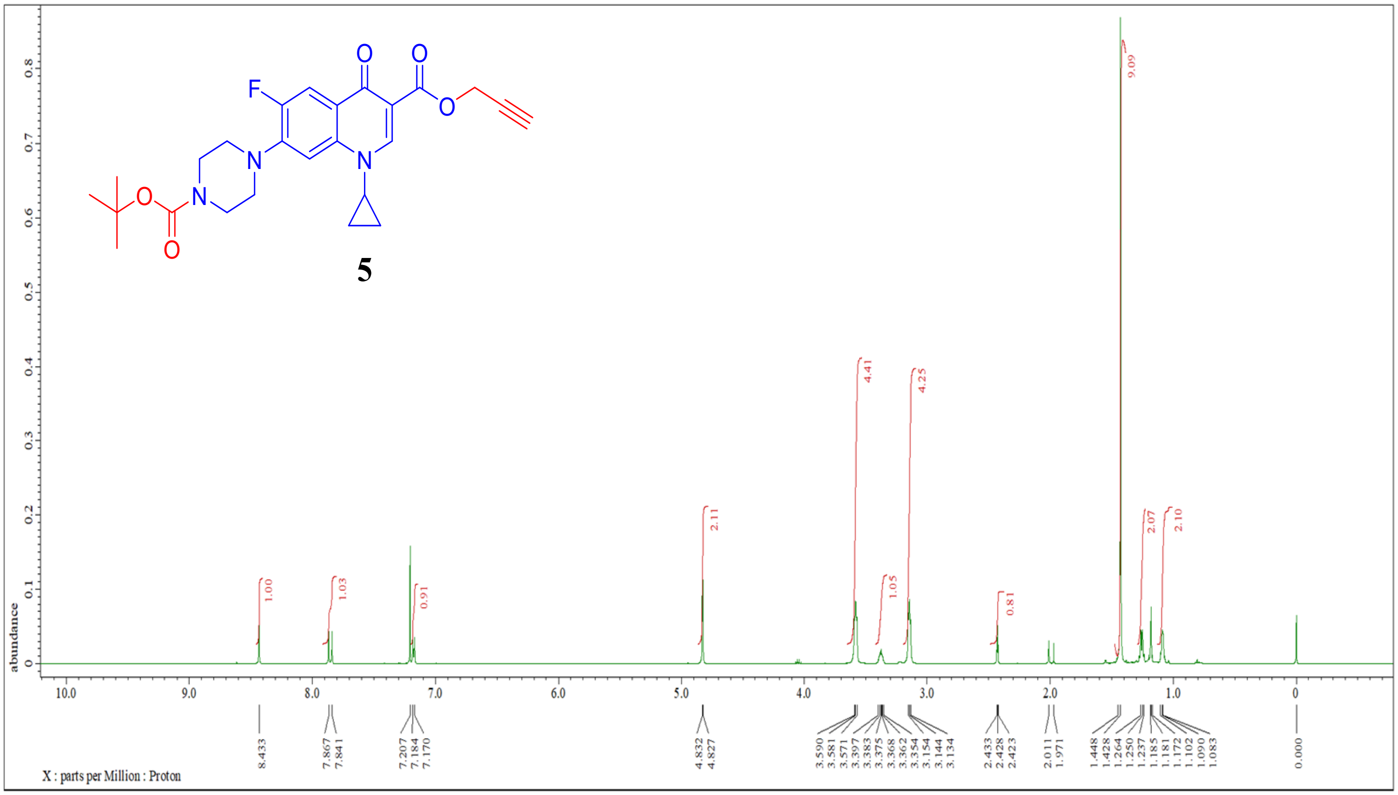

^{13}C-NMR spectrum of compound **5**

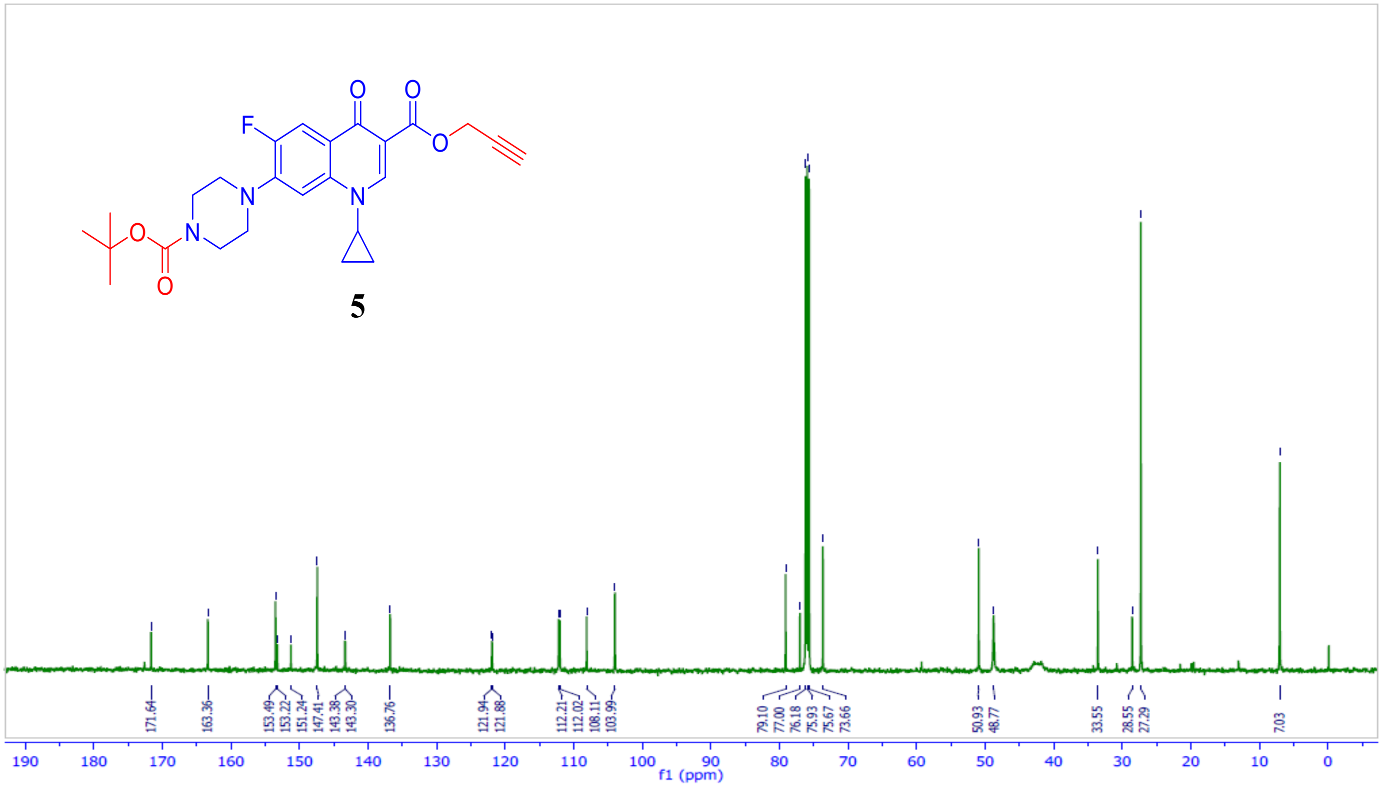

HRMS of compound **5**

**Data File**	CPR-03.d	**Sample Name**	CPR-03
**Sample Type**	Sample	**Position**	P1-D4
**Instrument Name**	Instrument 1	**User Name**	
**Acq Method**	MS Scan.m	**Acquired Time**	14-12-2022 15:01:09
**IRM Calibration Status**	Success	**DA Method**	Default.m
**Comment**			

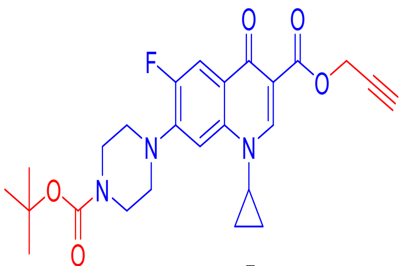

5

**Sample Group**		**Info.**	3
**Acquisition SW Version**	6200 series TOF/6500 series Q-TOF B.05.01 (B5125)		

Compound Table

Compound Label	RT	Mass	Abund	Formula	Tgt Mass	Diff (ppm)	MFG Formula	DB Formula
Cpd 1: C25 H28 F N3 O5	1.849	469.2003	18016	C25 H28 F N3 O5	469.2013	-2.07	C25 H28 F N3 O5	C25 H28 F N3 O5

Compound Label	*m/z*	RT	Algorithm	Mass
Cpd 1: C25 H28 F N3 O5	470.2075	1.849	Find By Formula	469.2003

MS Zoomed Spectrum

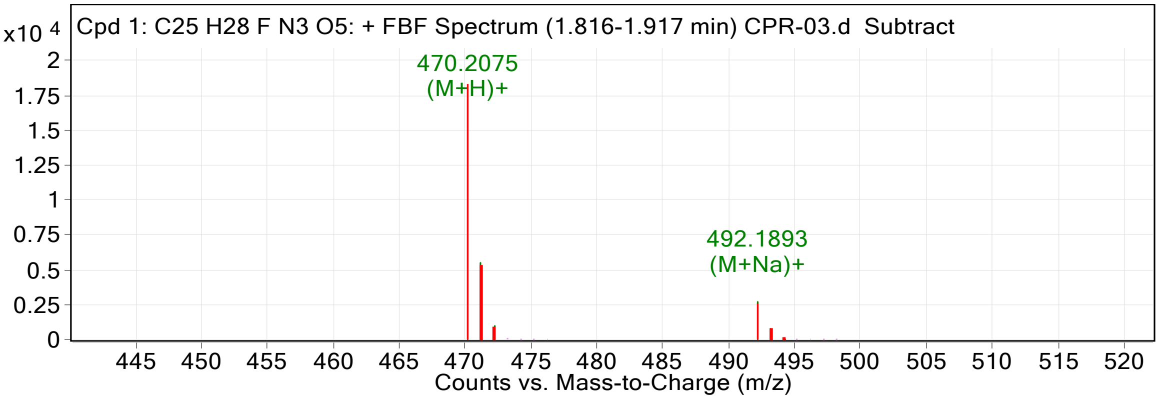

MS Spectrum Peak List

m/z	z	Abund	Formula	Ion
470.2075	1	18015.81	C25H29FN3O5	(M+H)+
471.2111	1	5512.48	C25H29FN3O5	(M+H)+
472.2142	1	975.06	C25H29FN3O5	(M+H)+
492.1893	1	2698.42	C25H28FN3NaO5	(M+Na)+
493.193	1	749.99	C25H28FN3NaO5	(M+Na)+
494.1997	1	51.79	C25H28FN3NaO5	(M+Na)+

--- End Of Report ---

^{1}H-NMR spectrum of compound **6**

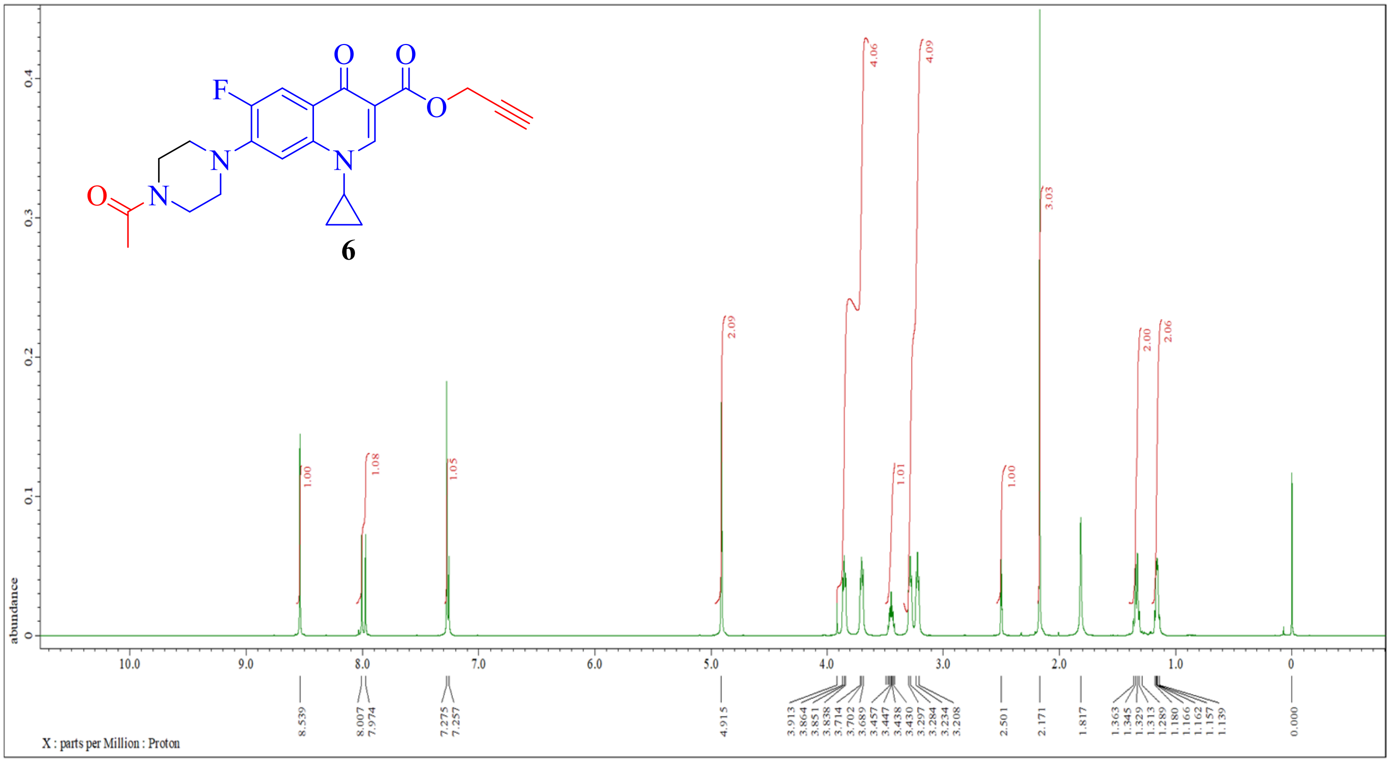

^{13}C-NMR spectrum of compound **6**

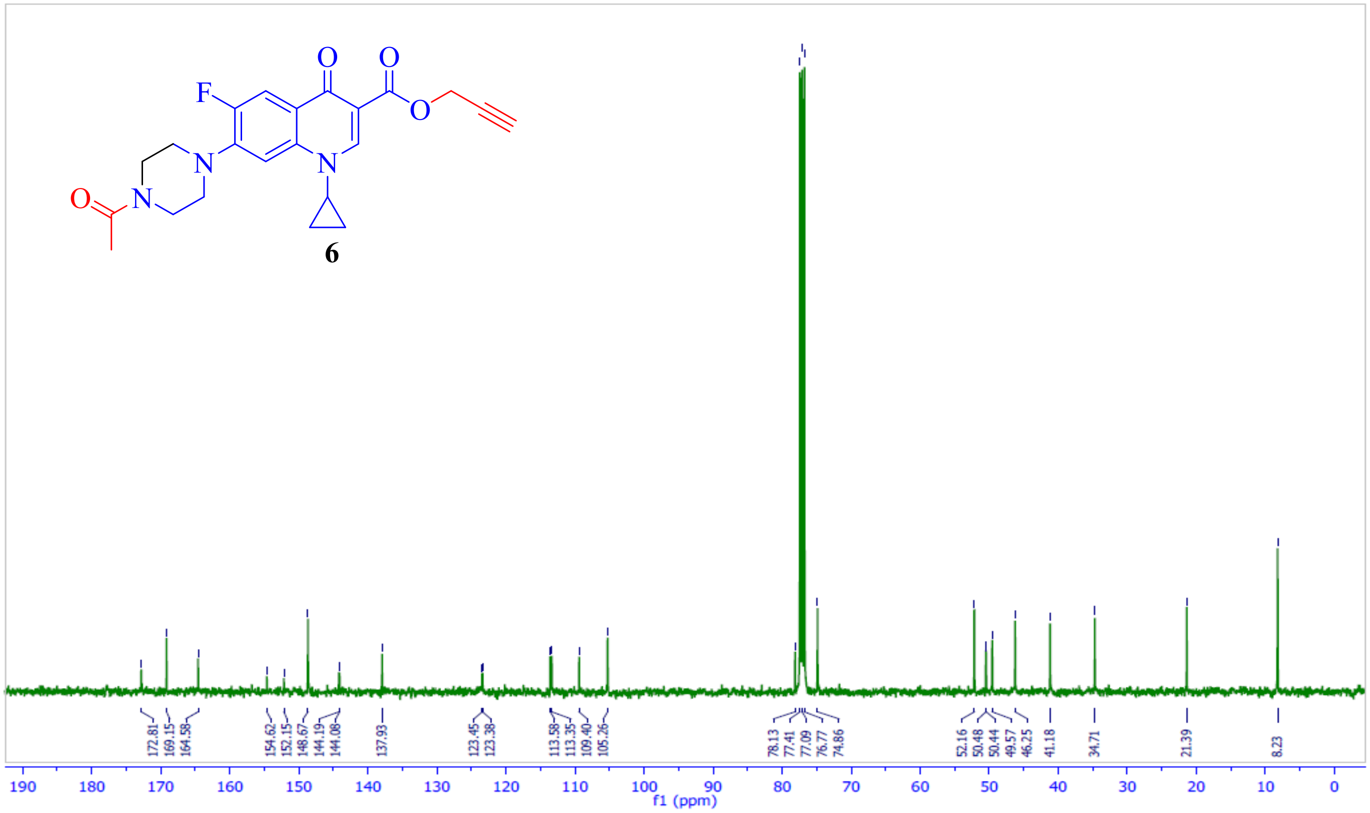

^{19}F-NMR spectrum of compound **6**

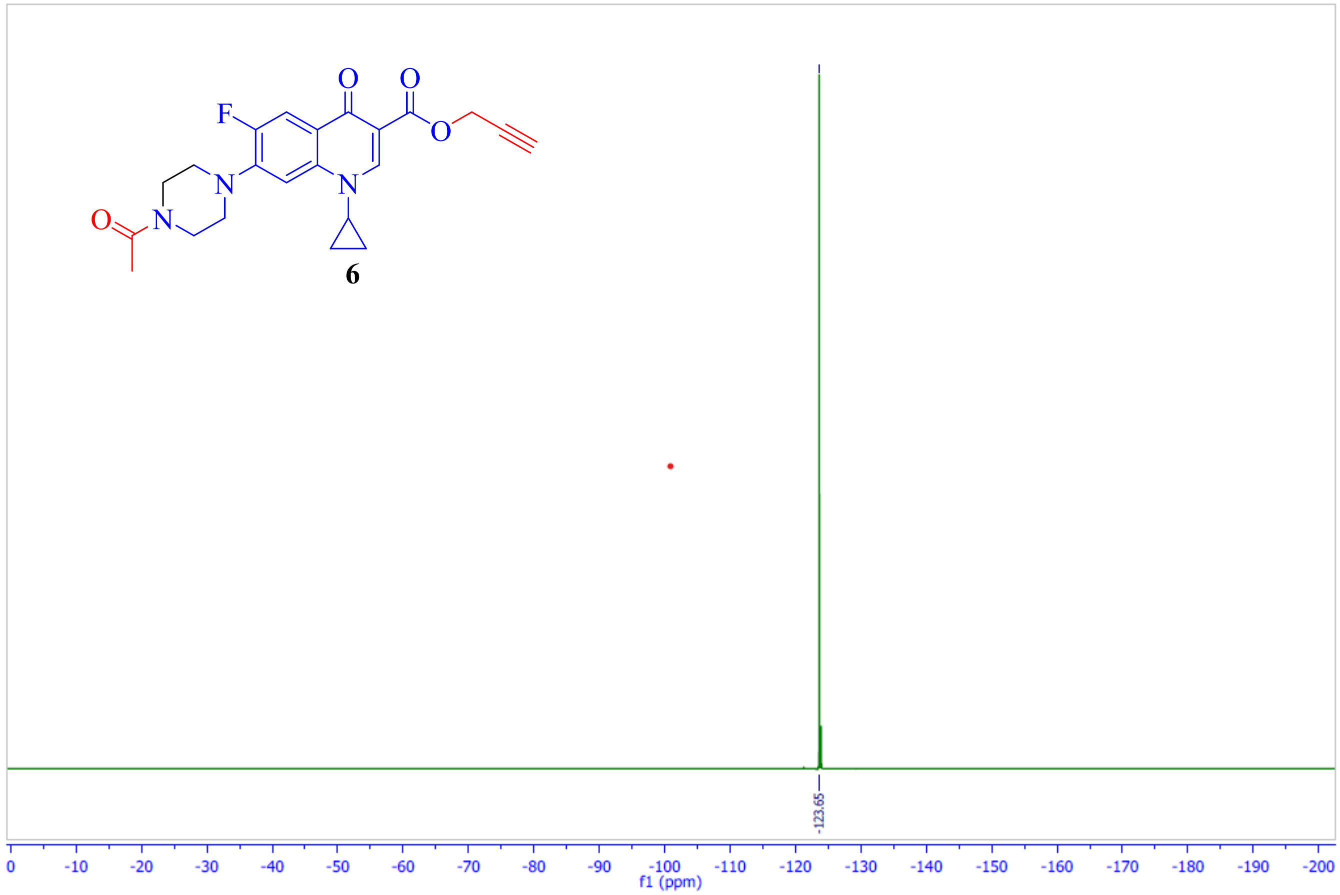

HRMS of compound **6**

**Data File**	CPR-18.d	**Sample Name**	CPR-18
**Sample Type**	Sample	**Position**	P1-C3
**Instrument Name**	Instrument 1	**User Name**	
**Acq Method**	MS Scan.m	**Acquired Time**	24-05-2023 13:50:37
**IRM Calibration Status**	Success	**DA Method**	Default.m
**Comment**			

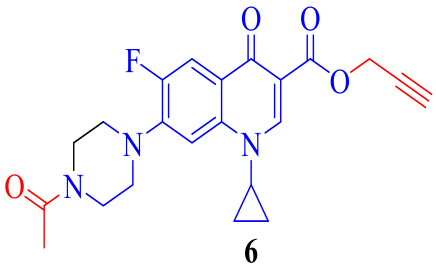

6

**Sample Group**		**Info.**	3
**Acquisition SW Version**	6200 series TOF/6500 series Q-TOF B.05.01 (B5125)		

Compound Table

Compound Label	RT	Mass	Abund	Formula	Tgt Mass	Diff (ppm)	MFG Formula	DB Formula
Cpd 1: C22 H22 F N3O4	0.137	411.1578	41486	C22 H22 F N3 O4	411.1594	-3.95	C22 H22 F N3 O4	C22 H22 F N3 O4

**Compound Label**	***m/z***	**RT**	**Algorithm**	**Mass**
Cpd 1: C22 H22 F N3 O4	434.1471	0.137	Find By Formula	411.1578

MS Zoomed Spectrum

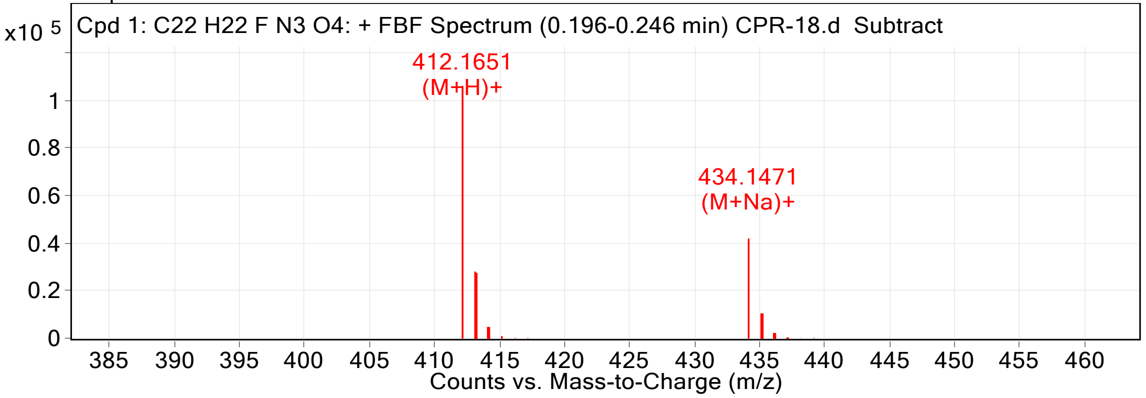

MS Spectrum Peak List

m/z	**z**	**Abund**	**Formula**	**Ion**
412.1651	1	105651.67	C22H23FN3O4	(M+H)+
413.1683	1	27719.85	C22H23FN3O4	(M+H)+
414.1706	1	3926.74	C22H23FN3O4	(M+H)+
415.1746	1	436.17	C22H23FN3O4	(M+H)+
434.1471	1	41485.79	C22H22FN3NaO4	(M+Na)+
435.1499	1	10222.06	C22H22FN3NaO4	(M+Na)+
436.1522	1	1431.65	C22H22FN3NaO4	(M+Na)+
437.1556	1	87.13	C22H22FN3NaO4	(M+Na)+

--- End Of Report ---

^{1}H-NMR spectrum of compound **7**

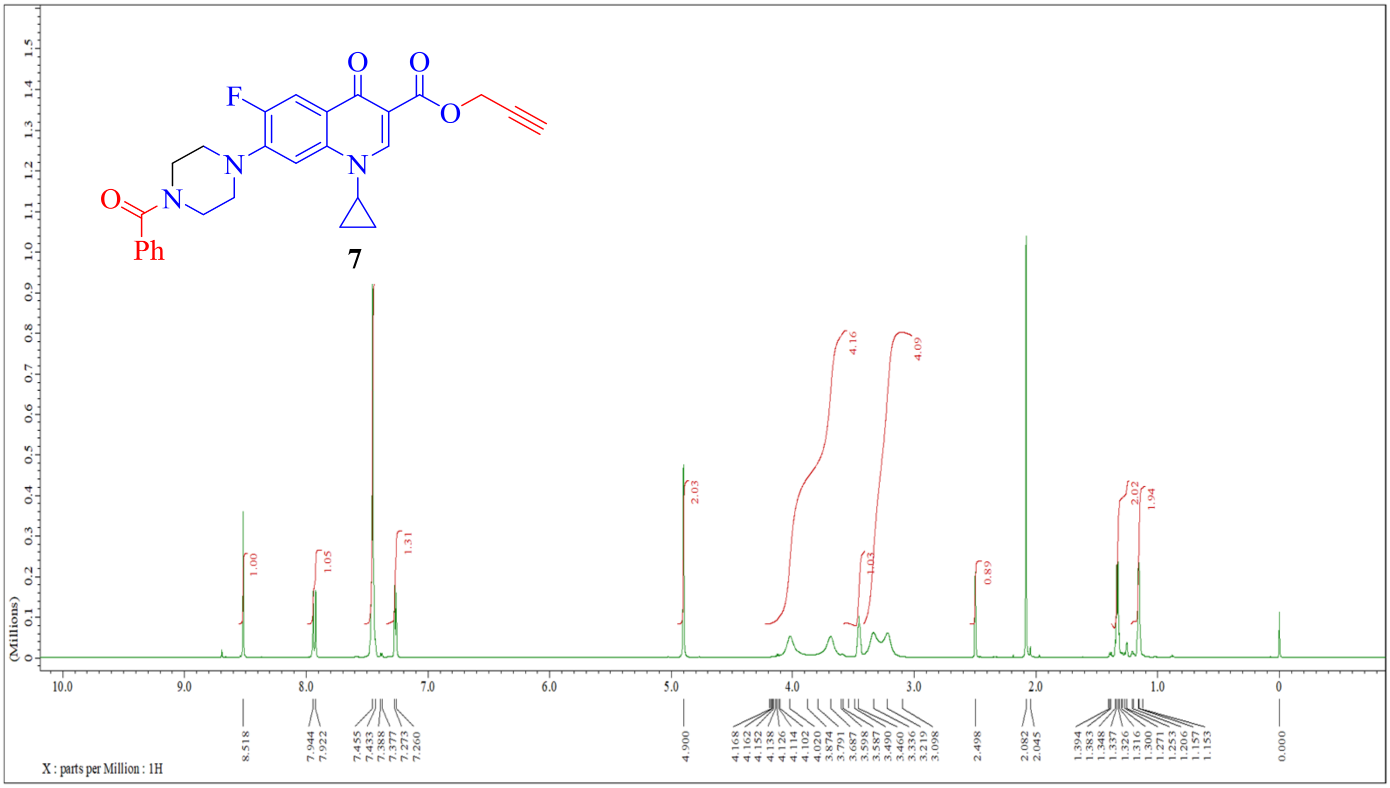

^{13}C-NMR spectrum of compound **7**

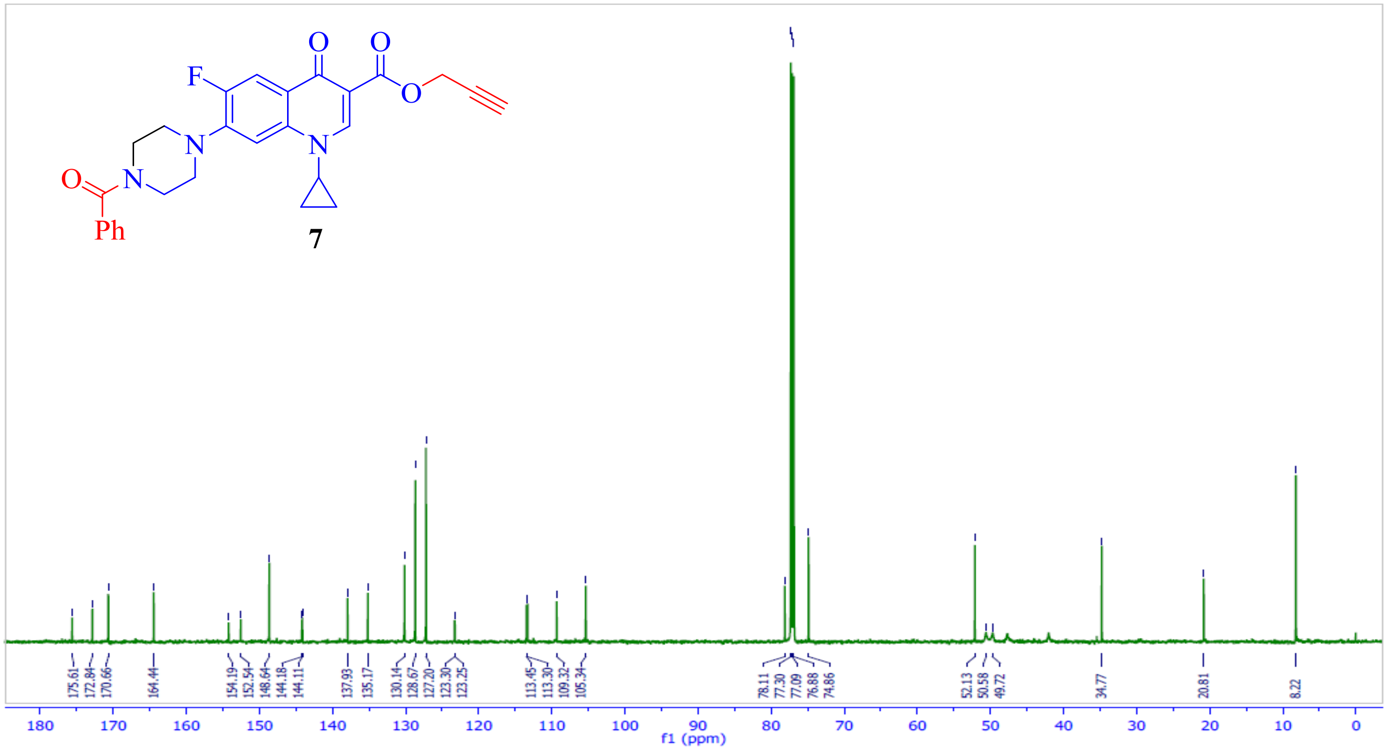

^{19}F-NMR spectrum of compound **7**

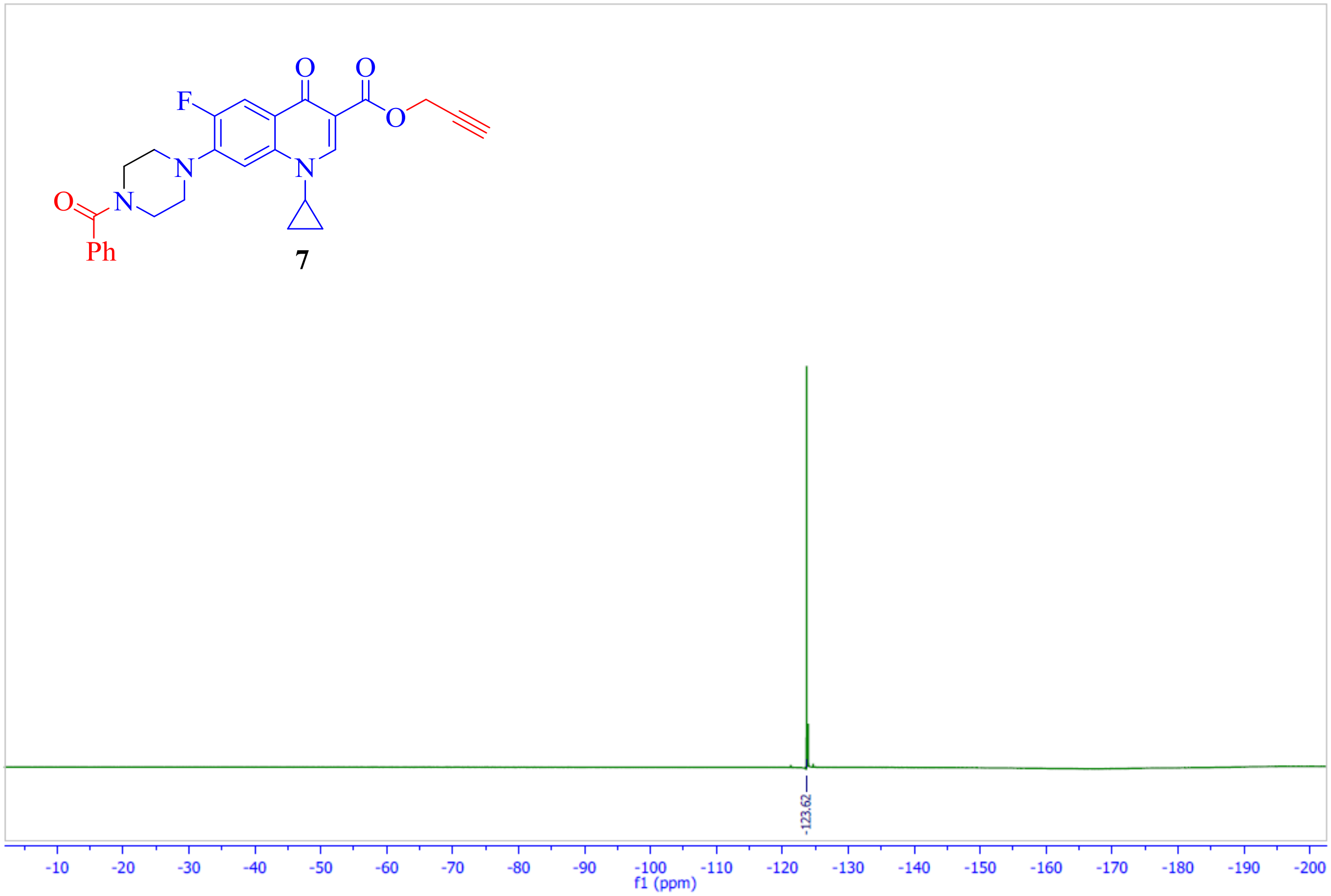

HRMS of compound **7**

**Data File**	CPR-26.d	**Sample Name**	CPR-26
**Sample Type**	Sample	**Position**	P1-E7
**Instrument Name**	Instrument 1	**User Name**	
**Acq Method**	MS Scan.m	**Acquired Time**	01-06-2023 16:39:20
**IRM Calibration Status**	Success	**DA Method**	Default.m
**Comment**			

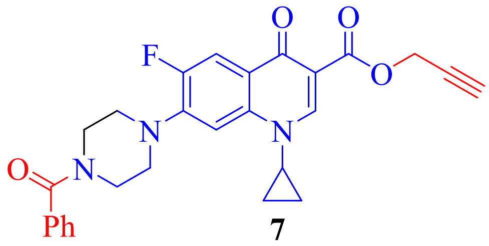

7

**Sample Group**		**Info.**	3
**Acquisition SW Version**	6200 series TOF/6500 series Q-TOF B.05.01 (B5125)		

Compound Table

Compound Label	RT	Mass	Abund	Formula	Tgt Mass	Diff (ppm)	MFG Formula	DB Formula
Cpd 1: C27 H24 F N3 O4	0.106	473.1766	91189	C27 H24 F N3 O4	473.1751	3.26	C27 H24 F N3 O4	C27 H24 F N3 O4

Compound Label	*m/z*	RT	Algorithm	Mass
Cpd 1: C27 H24 F N3 O4	474.184	0.106	Find By Formula	473.1766

MS Zoomed Spectrum

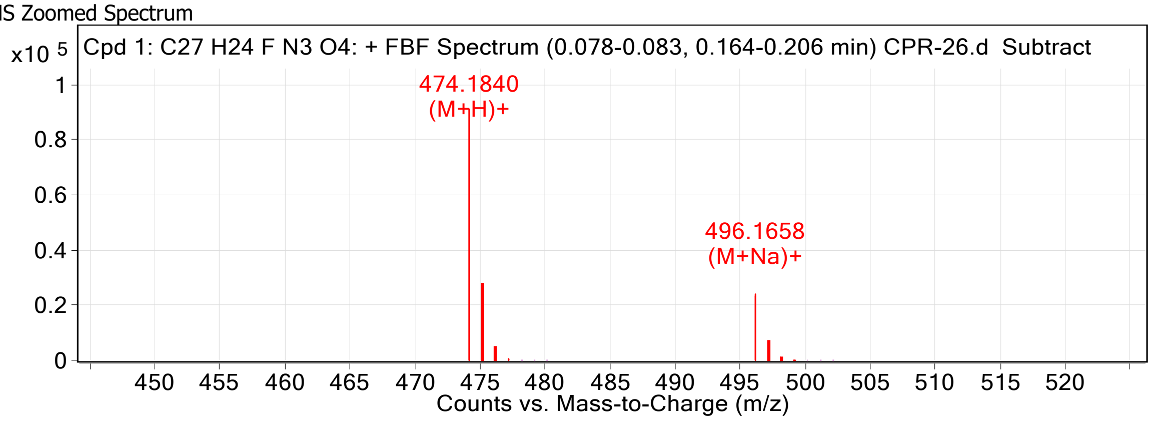

MS Spectrum Peak List

m/z	z	Abund	Formula	Ion
474.184	1	91188.99	C27H25FN3O4	(M+H)+
475.1871	1	28074.91	C27H25FN3O4	(M+H)+
476.1896	1	4533.76	C27H25FN3O4	(M+H)+
477.192	1	569.29	C27H25FN3O4	(M+H)+
496.1658	1	24142.34	C27H24FN3NaO4	(M+Na)+
497.1687	1	6712	C27H24FN3NaO4	(M+Na)+
498.1718	1	1075.6	C27H24FN3NaO4	(M+Na)+
499.1744	1	82.46	C27H24FN3NaO4	(M+Na)+

--- End Of Report ---

^{1}H-NMR spectrum of compound **9a**

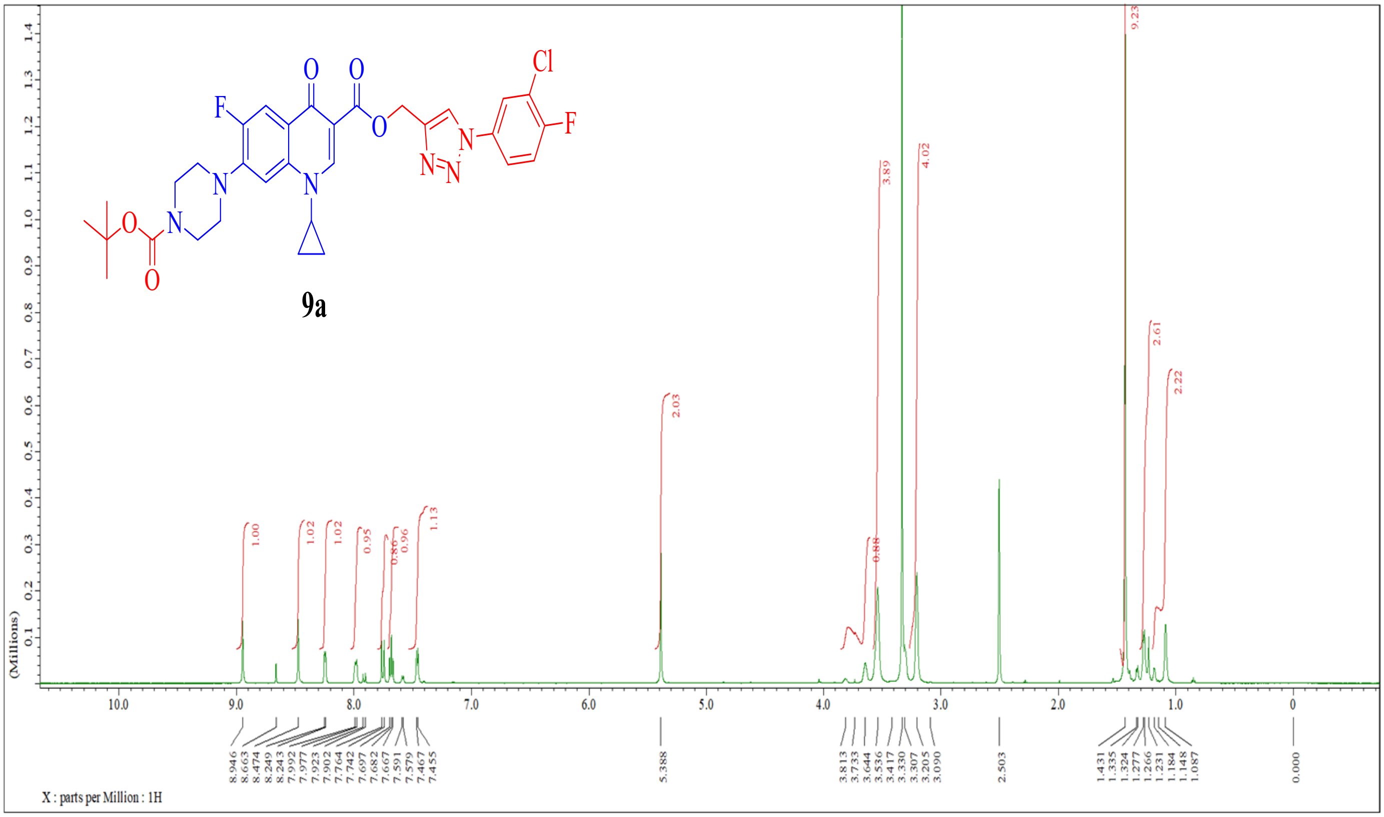

^{13}C-NMR spectrum of compound **9a**

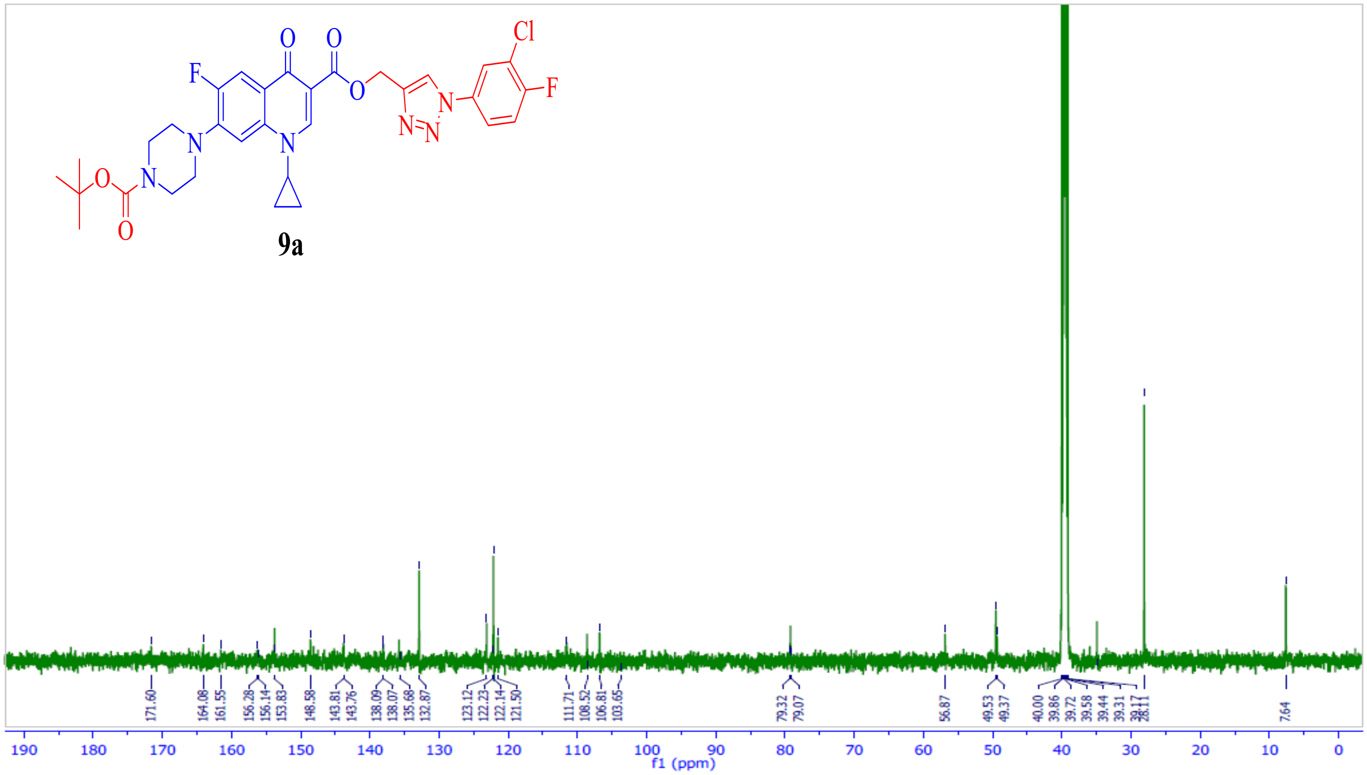

HRMS of compound **9a**

**Data File**	CPR-04.d	**Sample Name**	CPR-04
**Sample Type**	Sample	**Position**	P1-B8
**Instrument Name**	Instrument 1	**User Name**	
**Acq Method**	MS Scan.m	**Acquired Time**	24-05-2023 13:43:17
**IRM Calibration Status**	Success	**DA Method**	Default.m
**Comment**			

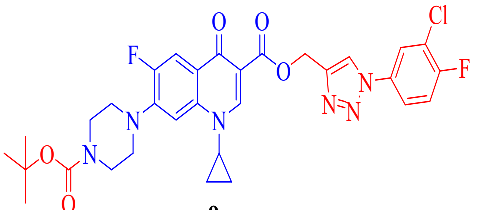

9a

**Sample Group**		**Info.**	3
**Acquisition SW Version**	6200 series TOF/6500 series Q-TOF B.05.01 (B5125)		

Compound Table

Compound Label	RT	Mass	Abund	Formula	Tgt Mass	Diff (ppm)	MFG Formula	DB Formula
Cpd 1: C31 H31 Cl F2 N6 O5	0.1	640.199	89395	C31 H31 Cl F2 N6 O5	640.2013	-3.55	C31 H31 Cl F2 N6 O5	C31 H31 Cl F2 N6 O5

Compound Label	*m/z*	RT	Algorithm	Mass
Cpd 1: C31 H31 Cl F2 N6 O5	641.2063	0.1	Find By Formula	640.199

MS Zoomed Spectrum

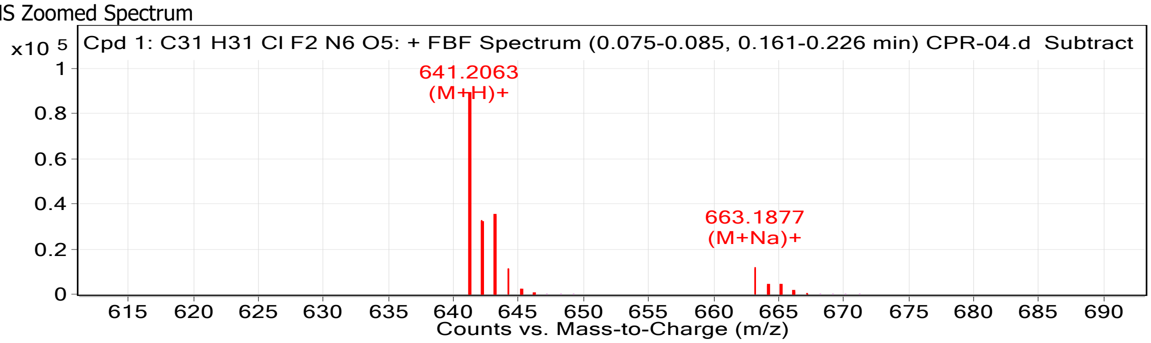

MS Spectrum Peak List

m/z	z	Abund	Formula	Ion
641.2063	1	89395.34	C31H32ClF2N6O5	(M+H)+
642.2095	1	32762.94	C31H32ClF2N6O5	(M+H)+
643.2052	1	34802.82	C31H32ClF2N6O5	(M+H)+
644.2067	1	11105.19	C31H32ClF2N6O5	(M+H)+
645.2089	1	2049.92	C31H32ClF2N6O5	(M+H)+
646.2106	1	242.11	C31H32ClF2N6O5	(M+H)+
663.1877	1	11554.72	C31H31ClF2N6NaO5	(M+Na)+
664.1904	1	3927.82	C31H31ClF2N6NaO5	(M+Na)+
665.1861	1	4251.23	C31H31ClF2N6NaO5	(M+Na)+
666.1878	1	1250.41	C31H31ClF2N6NaO5	(M+Na)+
667.1898	1	235.08	C31H31ClF2N6NaO5	(M+Na)+

--- End Of Report ---

^{1}H-NMR spectrum of compound **9b**

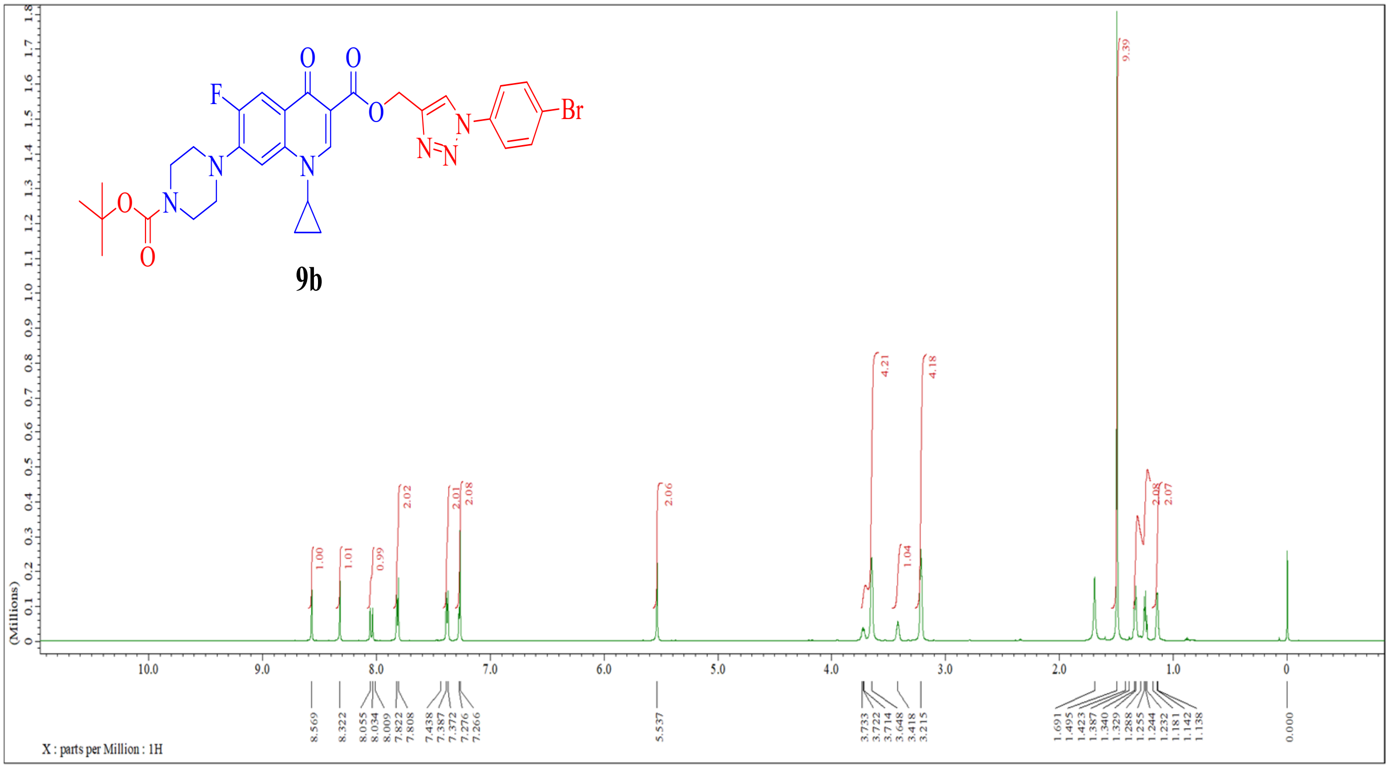

^{13}C-NMR spectrum of compound **9b**

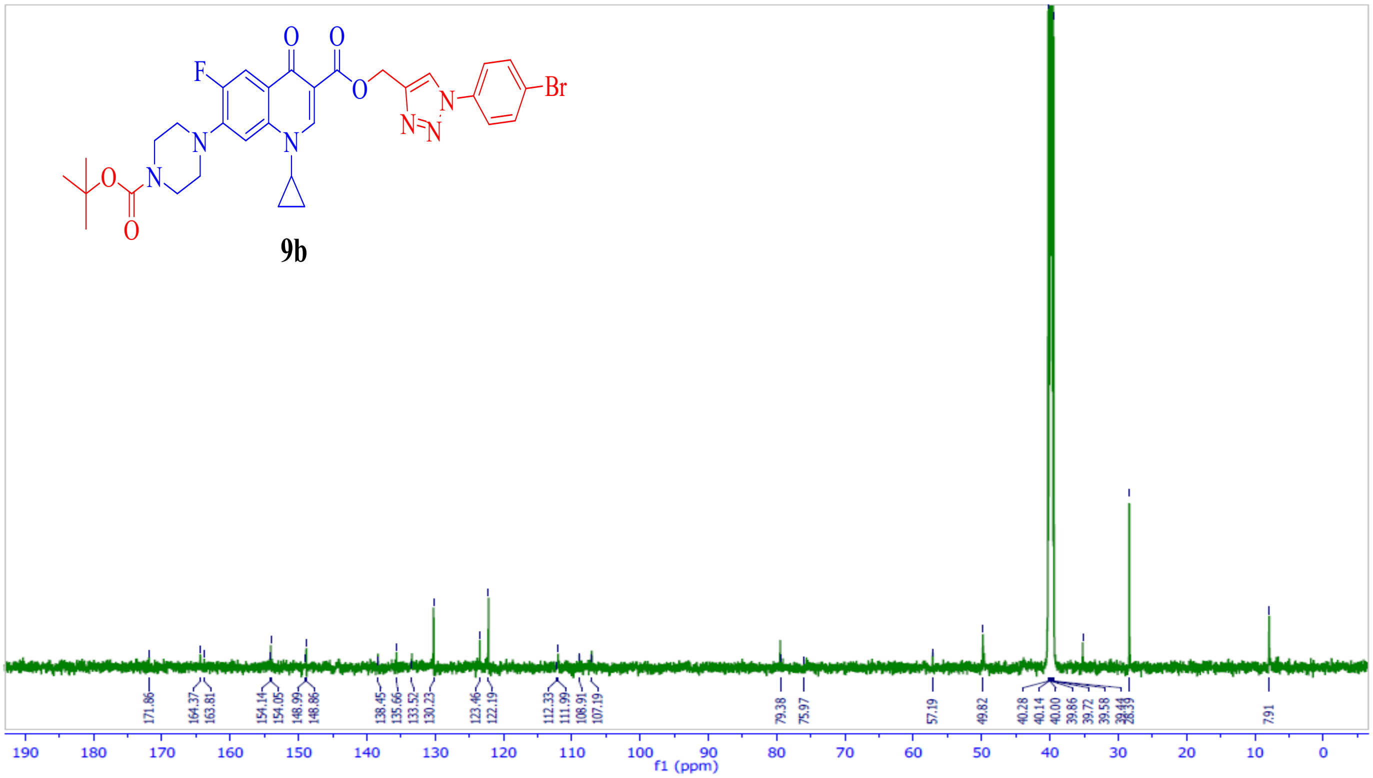

HRMS of compound **9b**

**Data File**	CPR-05.d	**Sample Name**	CPR-05
**Sample Type**	Sample	**Position**	P1-D6
**Instrument Name**	Instrument 1	**User Name**	
**Acq Method**	MS Scan.m	**Acquired Time**	14-12-2022 15:08:40
**IRM Calibration Status**	Success	**DA Method**	Default.m
**Comment**			

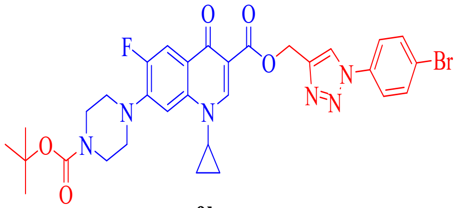

9b

**Sample Group**		**Info.**	3
**Acquisition SW Version**	6200 series TOF/6500 series Q-TOF B.05.01 (B5125)		

Compound Table

Compound Label	RT	Mass	Abund	Formula	Tgt Mass	Diff (ppm)	MFG Formula	DB Formula
Cpd 1: C31 H32 Br F N6 O5	0.525	666.1591	7827	C31 H32 Br F N6 O5	666.1602	-1.64	C31 H32 Br F N6 O5	C31 H32 Br F N6 O5

Compound Label	*m/z*	RT	Algorithm	Mass
Cpd 1: C31 H32 Br F N6 O5	669.1645	0.525	Find By Formula	666.1591

MS Zoomed Spectrum

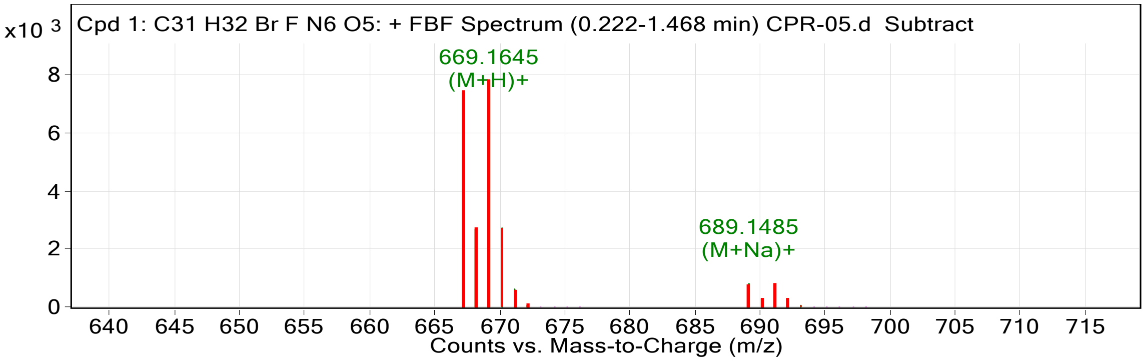

MS Spectrum Peak List

m/z	z	Abund	Formula	Ion
667.166	1	7412.58	C31H33BrFN6O5	(M+H)+
668.1694	1	2682.78	C31H33BrFN6O5	(M+H)+
669.1645	1	7827.28	C31H33BrFN6O5	(M+H)+
670.1675	1	2733.04	C31H33BrFN6O5	(M+H)+
671.1732	1	624.4	C31H33BrFN6O5	(M+H)+
672.1624	1	31.42	C31H33BrFN6O5	(M+H)+
689.1485	1	788.61	C31H32BrFN6NaO5	(M+Na)+
690.1544	1	270.97	C31H32BrFN6NaO5	(M+Na)+
691.1485	1	752.61	C31H32BrFN6NaO5	(M+Na)+
692.1497	1	285.21	C31H32BrFN6NaO5	(M+Na)+
693.172	1	32.4	C31H32BrFN6NaO5	(M+Na)+

--- End Of Report ---

^{1}H-NMR spectrum of compound **9c**

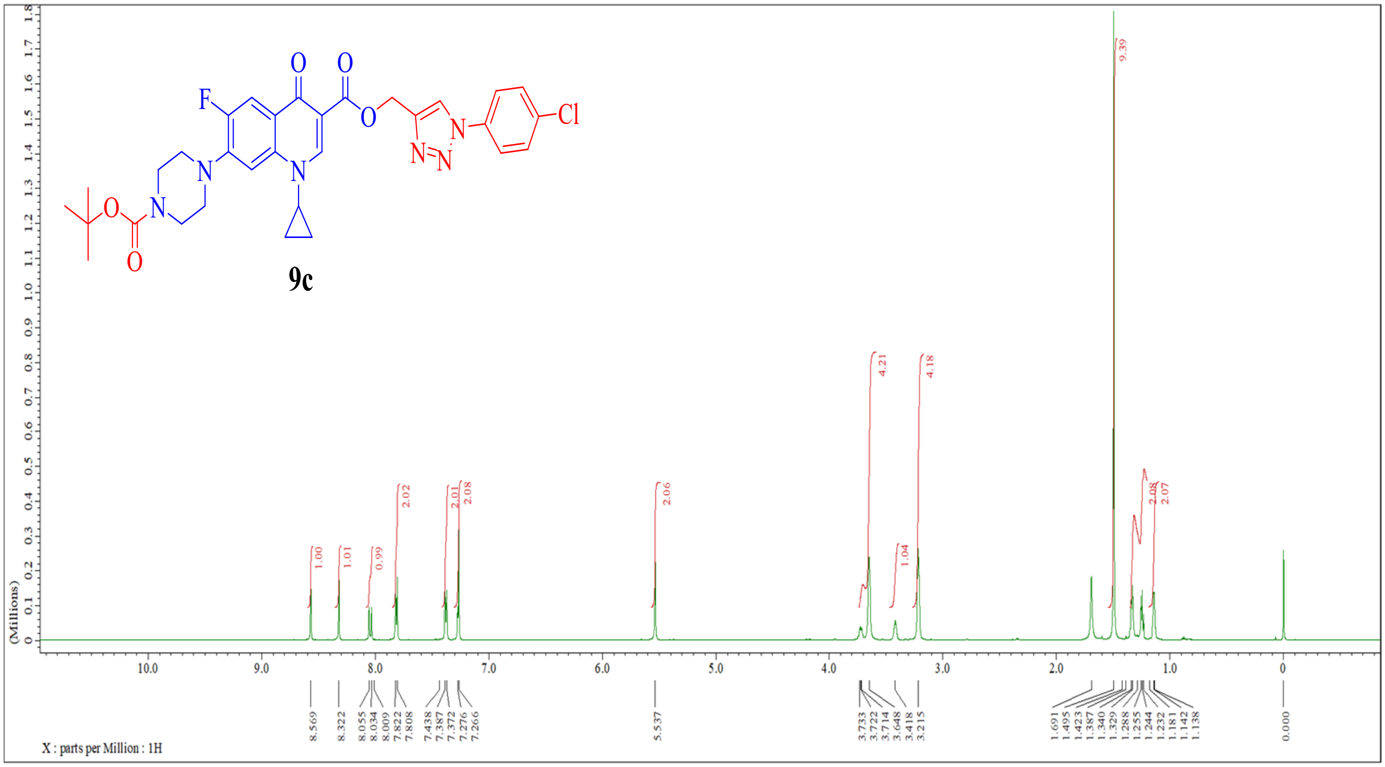

^{13}C-NMR spectrum of compound **9c**

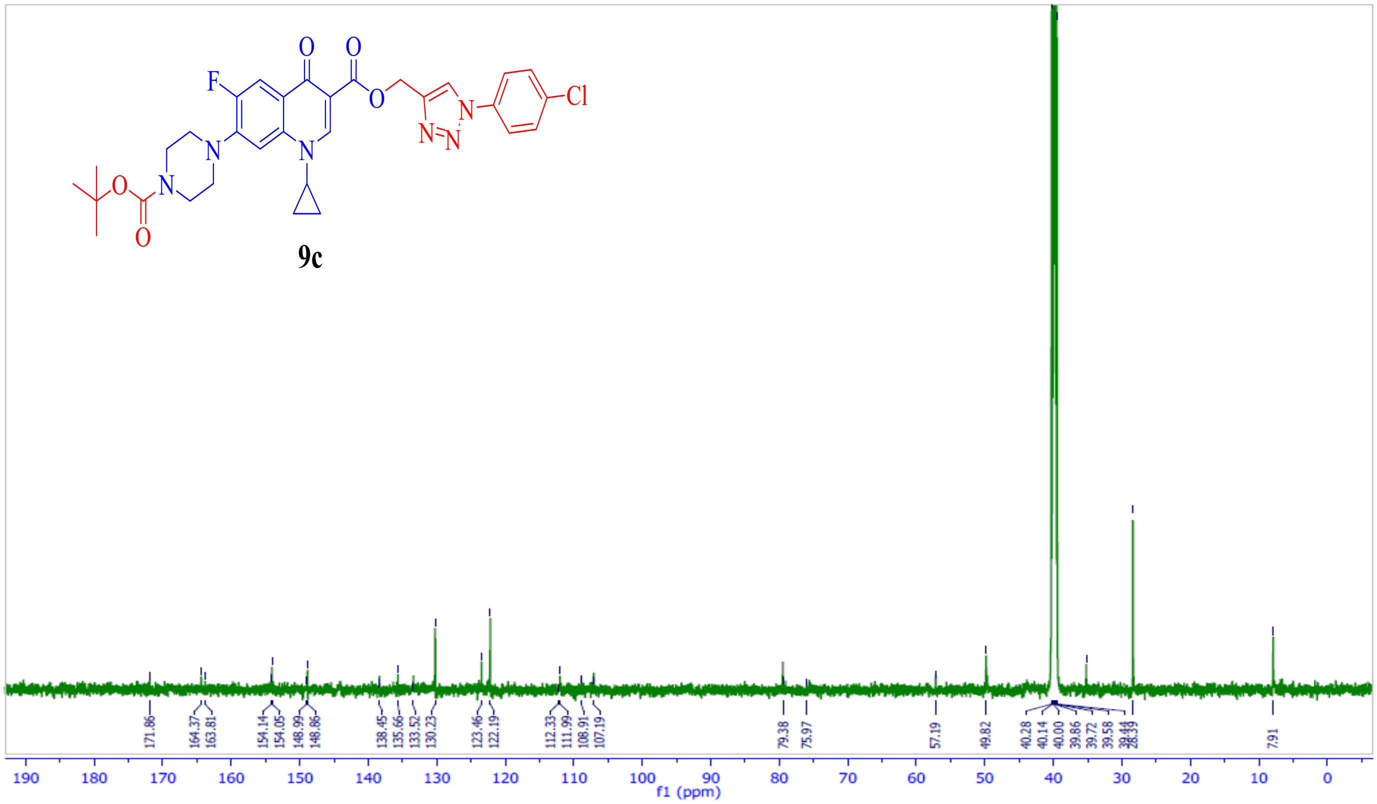

^{1}H-NMR spectrum of compound **9d**

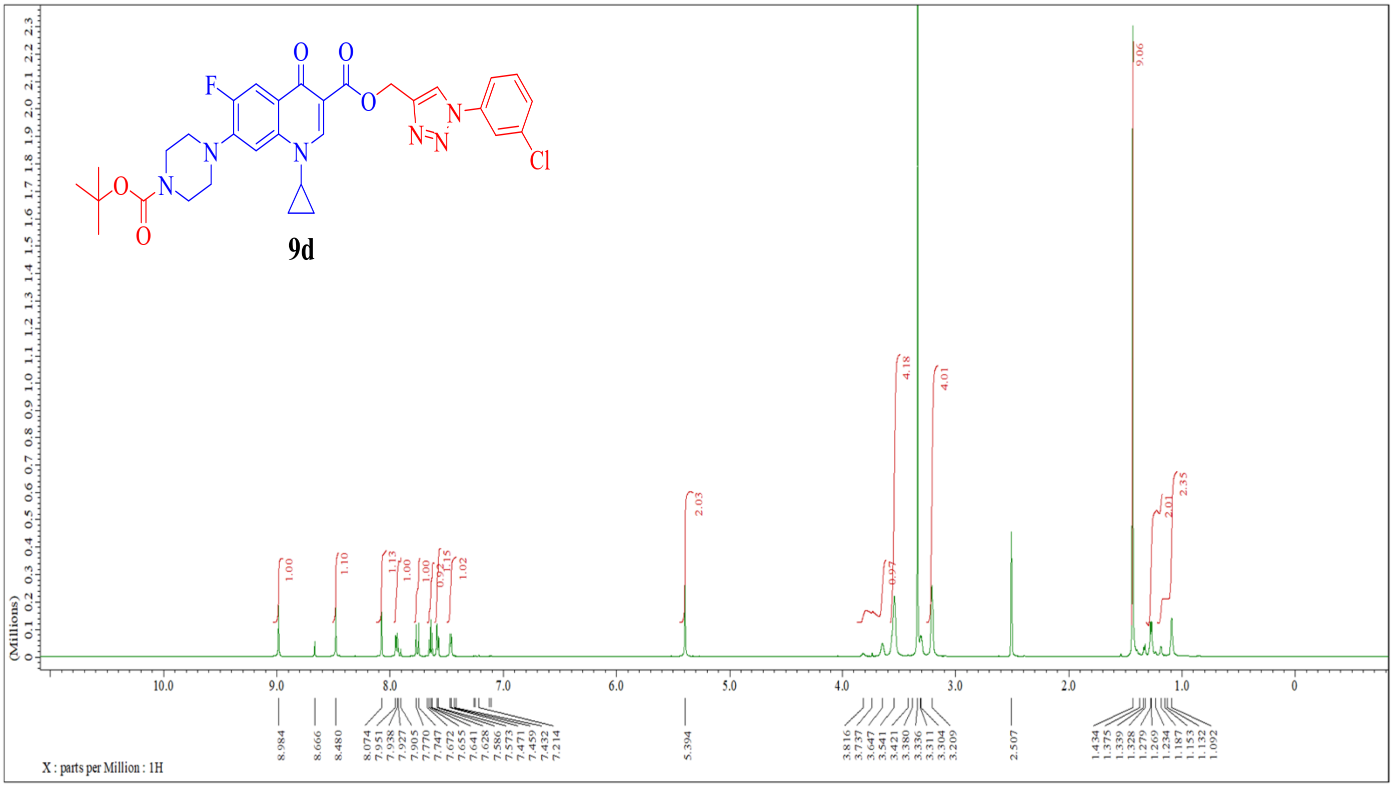

^{13}C-NMR spectrum of compound **9d**

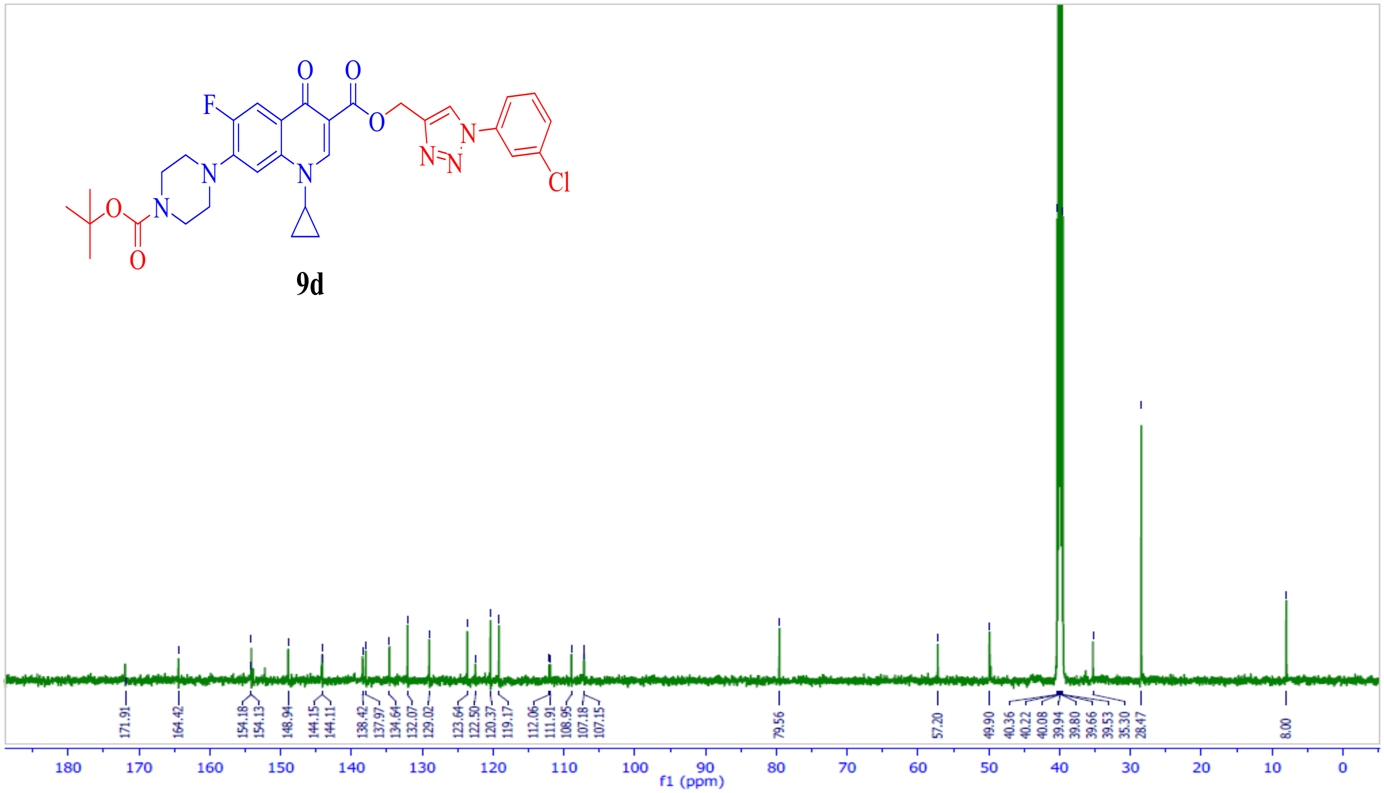

HRMS of compound **9d**

**Data File**	CPR-07.d	**Sample Name**	CPR-07
**Sample Type**	Sample	**Position**	P1-D3
**Instrument Name**	Instrument 1	**User Name**	
**Acq Method**	MS Scan.m	**Acquired Time**	28-12-2022 16:37:36
**IRM Calibration Status**	Success	**DA Method**	Default.m
**Comment**			
**Sample Group**		**Info.**	3
**Acquisition SW Version**	6200 series TOF/6500 series Q-TOF B.05.01 (B5125)		

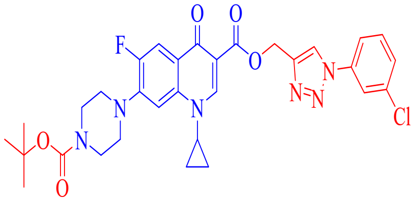

9d

Compound Table

Compound Label	RT	Mass	Abund	Formula	Tgt Mass	Diff (ppm)	MFG Formula	DB Formula
Cpd 1: C31 H32 Cl F N6 O5	0.193	622.2145	45475	C31 H32 Cl F N6 O5	622.2107	6.16	C31 H32 Cl F N6 O5	C31 H32 Cl F N6 O5

Compound Label	*m/z*	RT	Algorithm	Mass
Cpd 1: C31 H32 Cl F N6 O5	645.2029	0.193	Find By Formula	622.2145

MS Zoomed Spectrum

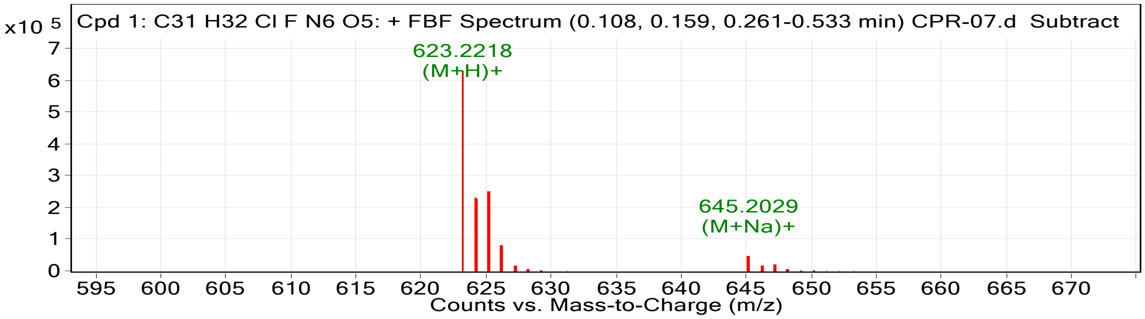

MS Spectrum Peak List

m/z	z	Abund	Formula	Ion
623.2218	1	630233.25	C31H33ClFN6O5	(M+H)+
624.2251	1	228400.81	C31H33ClFN6O5	(M+H)+
625.2207	1	246488.89	C31H33ClFN6O5	(M+H)+
626.2222	1	79270.67	C31H33ClFN6O5	(M+H)+
627.2242	1	15238.84	C31H33ClFN6O5	(M+H)+
628.2269	1	2054.51	C31H33ClFN6O5	(M+H)+
629.2251	1	362.38	C31H33ClFN6O5	(M+H)+
645.2029	1	45474.88	C31H32ClFN6NaO5	(M+Na)+
646.2057	1	15853.03	C31H32ClFN6NaO5	(M+Na)+
647.2015	1	16617.12	C31H32ClFN6NaO5	(M+Na)+
648.2037	1	5387.89	C31H32ClFN6NaO5	(M+Na)+
649.2059	1	1126.82	C31H32ClFN6NaO5	(M+Na)+
650.2034	1	158.21	C31H32ClFN6NaO5	(M+Na)+

--- End Of Report ---

^{1}H-NMR spectrum of compound **9e**

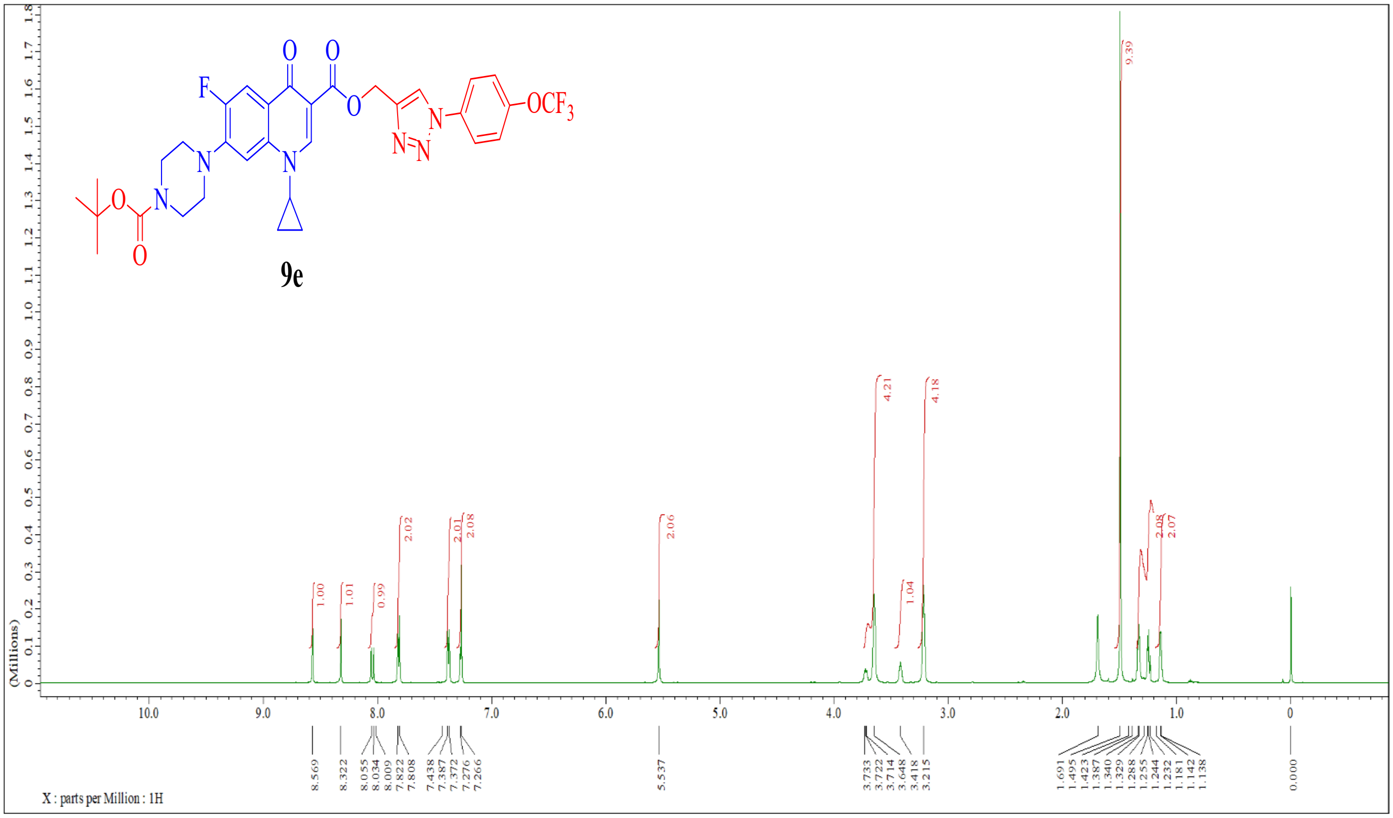

^{13}C-NMR spectrum of compound **9e**

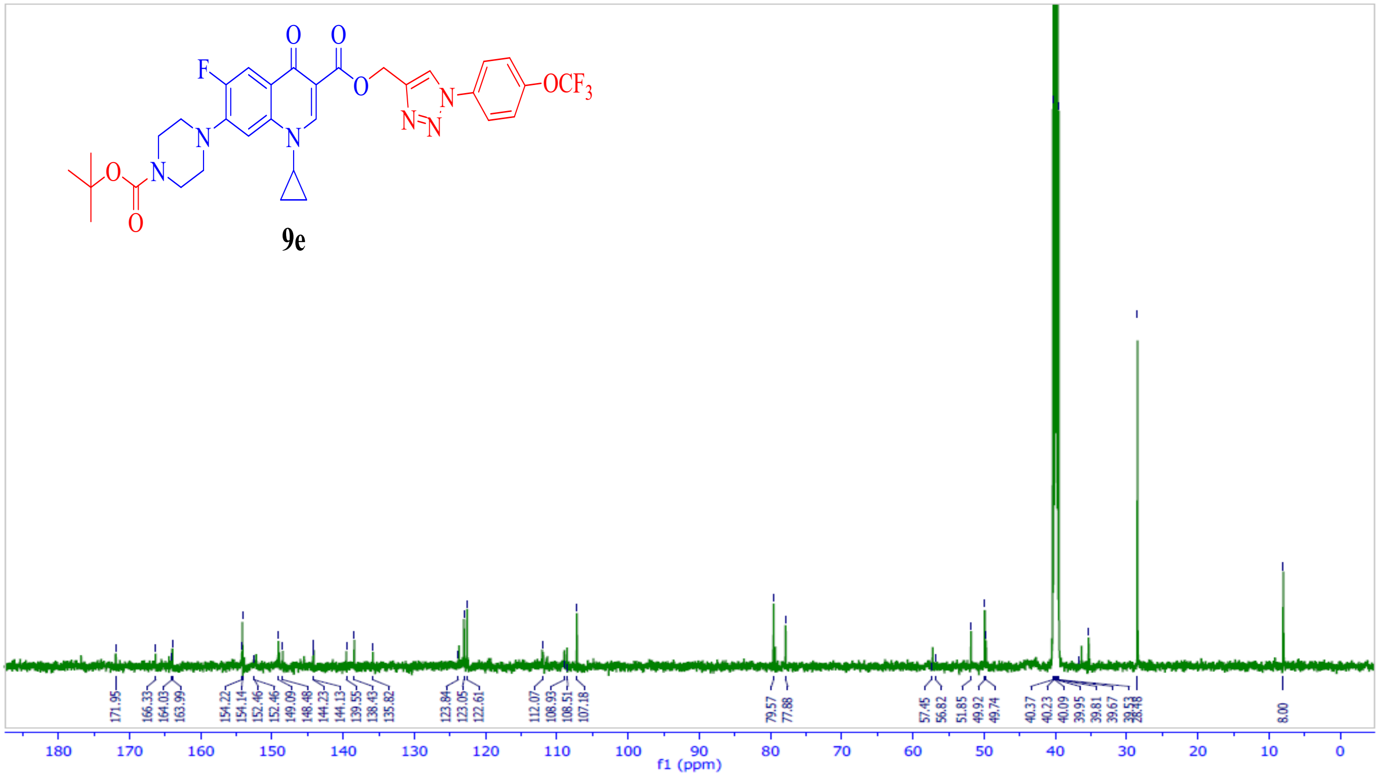

HRMS of compound **9e**

**Data File**	CPR-08.d	**Sample Name**	CPR-08
**Sample Type**	Sample	**Position**	P1-D4
**Instrument Name**	Instrument 1	**User Name**	
**Acq Method**	MS Scan.m	**Acquired Time**	28-12-2022 16:38:20
**IRM Calibration Status**	Success	**DA Method**	Default.m
**Comment**			
**Sample Group**		**Info.**	3
**Acquisition SW Version**	6200 series TOF/6500 series Q-TOF B.05.01 (B5125)		

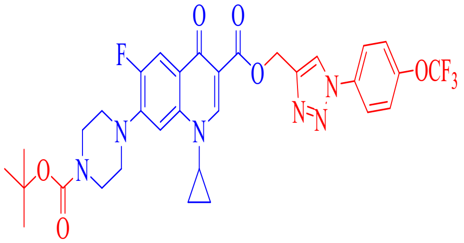

9e

Compound Table

Compound Label	RT	Mass	Abund	Formula	Tgt Mass	Diff (ppm)	MFG Formula	DB Formula
Cpd 1: C32 H32 F4 N6 O6	0.205	672.236	14339	C32 H32 F4 N6 O6	672.2319	5.98	C32 H32 F4 N6 O6	C32 H32 F4 N6 O6

Compound Label	*m/z*	RT	Algorithm	Mass
Cpd 1: C32 H32 F4 N6 O6	695.2243	0.205	Find By Formula	672.236

MS Zoomed Spectrum

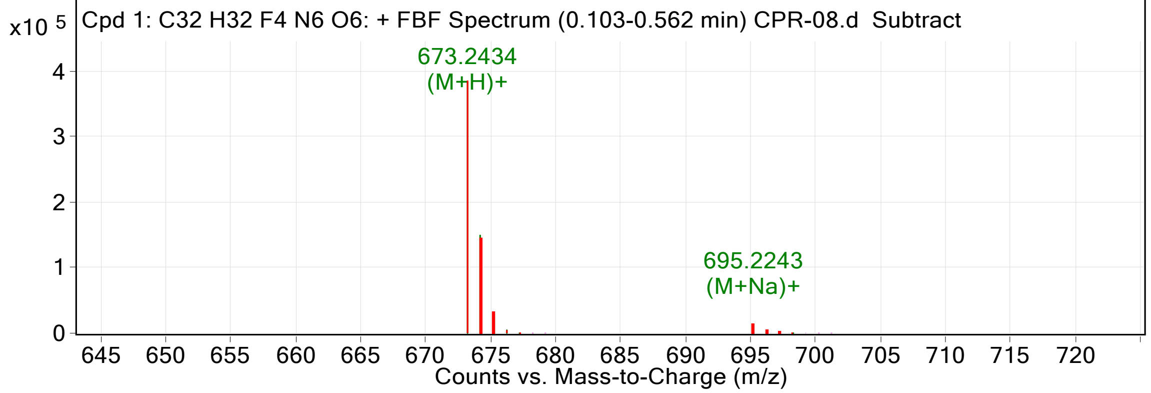

MS Spectrum Peak List

m/z	z	Abund	Formula	Ion
673.2434	1	383980.75	C32H33F4N6O6	(M+H)+
674.2461	1	148412.72	C32H33F4N6O6	(M+H)+
675.2483	1	29916.4	C32H33F4N6O6	(M+H)+
676.2509	1	4662.44	C32H33F4N6O6	(M+H)+
677.2529	1	625.35	C32H33F4N6O6	(M+H)+
695.2243	1	14338.55	C32H32F4N6NaO6	(M+Na)+
696.2271	1	5335.1	C32H32F4N6NaO6	(M+Na)+
697.2317	1	1204.33	C32H32F4N6NaO6	(M+Na)+
698.2398	1	185.15	C32H32F4N6NaO6	(M+Na)+

--- End Of Report ---

^{1}H-NMR spectrum of compound **9f**

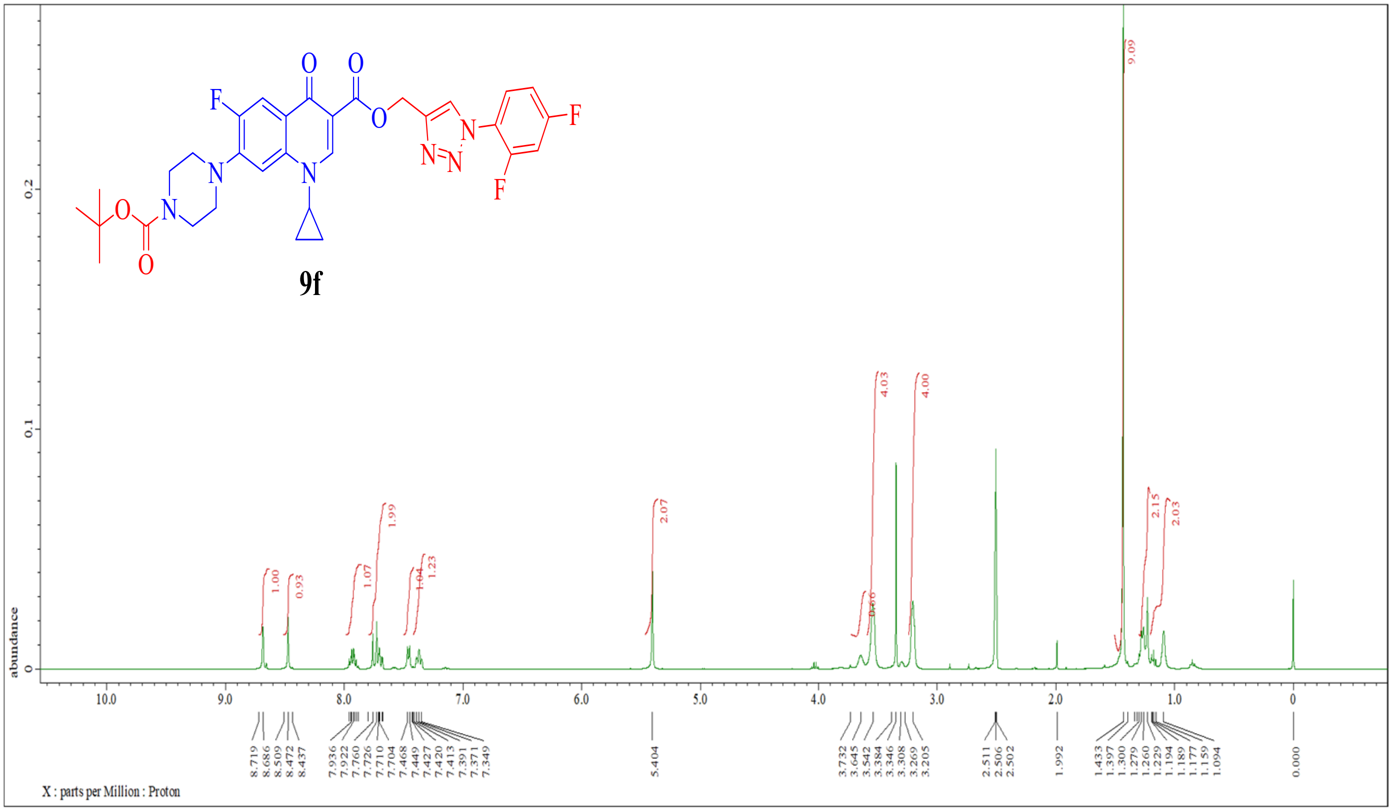

^{13}C-NMR spectrum of compound **9f**

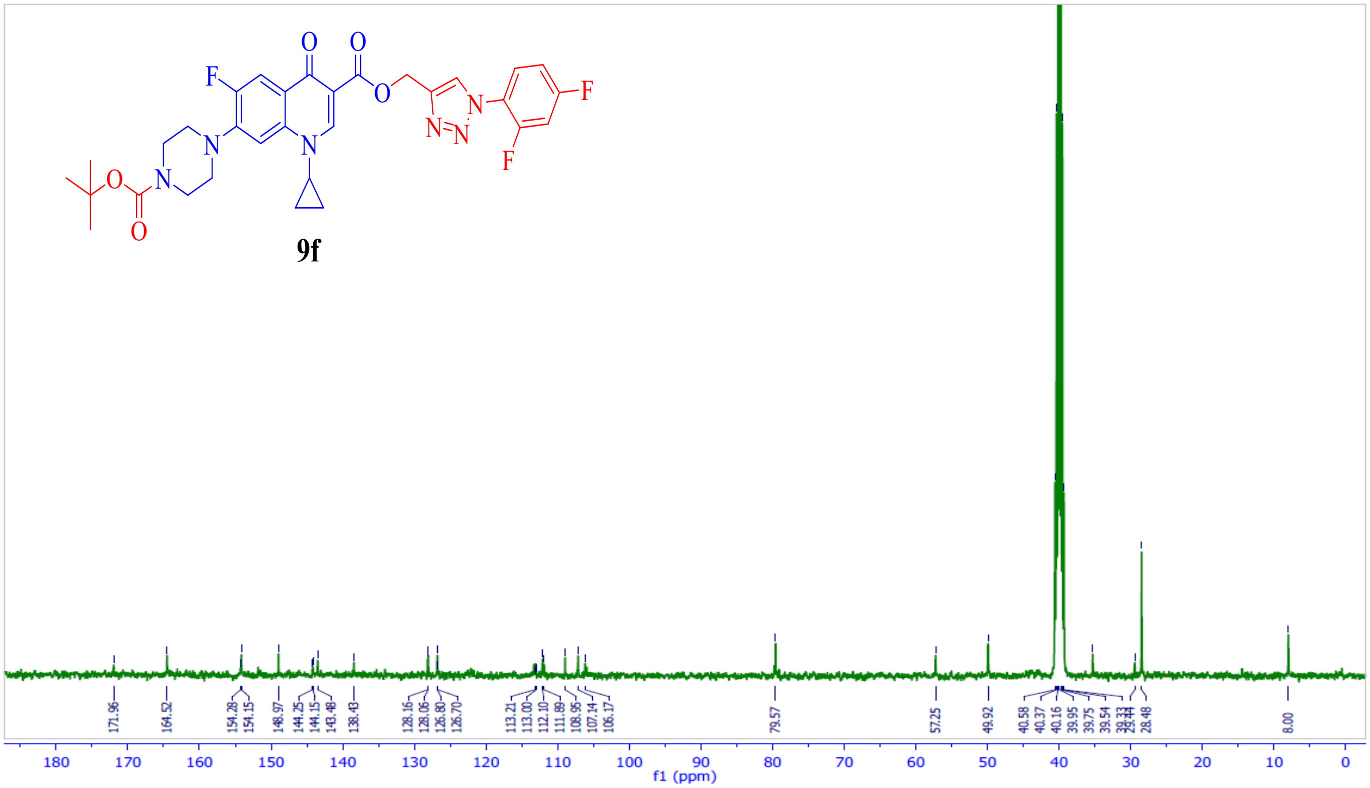

HRMS of compound **9f**

**Data File**	CPR-09.d	**Sample Name**	CPR-09
**Sample Type**	Sample	**Position**	P1-B9
**Instrument Name**	Instrument 1	**User Name**	
**Acq Method**	MS Scan.m	**Acquired Time**	24-05-2023 13:45:33
**IRM Calibration Status**	Success	**DA Method**	Default.m
**Comment**			

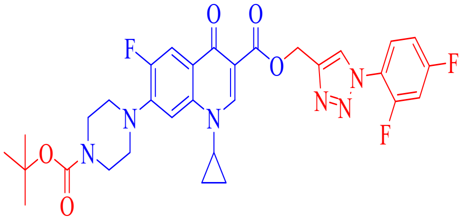

9f

**Sample Group**		**Info.**	3
**Acquisition SW Version**	6200 series TOF/6500 series Q-TOF B.05.01 (B5125)		

Compound Table

Compound Label	RT	Mass	Abund	Formula	Tgt Mass	Diff (ppm)	MFG Formula	DB Formula
Cpd 1: C31 H31 F3 N6 O5	0.098	624.2284	110742	C31 H31 F3 N6 O5	624.2308	-3.87	C31 H31 F3 N6 O5	C31 H31 F3 N6 O5

Compound Label	*m/z*	RT	Algorithm	Mass
Cpd 1: C31 H31 F3 N6 O5	625.2357	0.098	Find By Formula	624.2284

MS Zoomed Spectrum

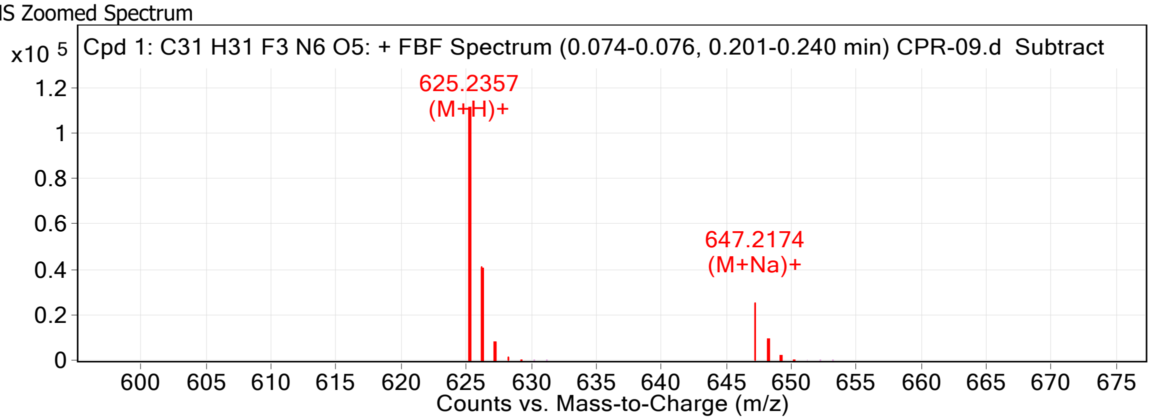

MS Spectrum Peak List

m/z	z	Abund	Formula	Ion
625.2357	1	110742.09	C31H32F3N6O5	(M+H)+
626.239	1	40953.04	C31H32F3N6O5	(M+H)+
627.2412	1	7987.41	C31H32F3N6O5	(M+H)+
628.2438	1	1142.87	C31H32F3N6O5	(M+H)+
629.2457	1	71.75	C31H32F3N6O5	(M+H)+
647.2174	1	25471.64	C31H31F3N6NaO5	(M+Na)+
648.2201	1	8874.06	C31H31F3N6NaO5	(M+Na)+
649.2228	1	1592.32	C31H31F3N6NaO5	(M+Na)+
650.2248	1	221.78	C31H31F3N6NaO5	(M+Na)+

--- End Of Report ---

^{1}H-NMR spectrum of compound **10**

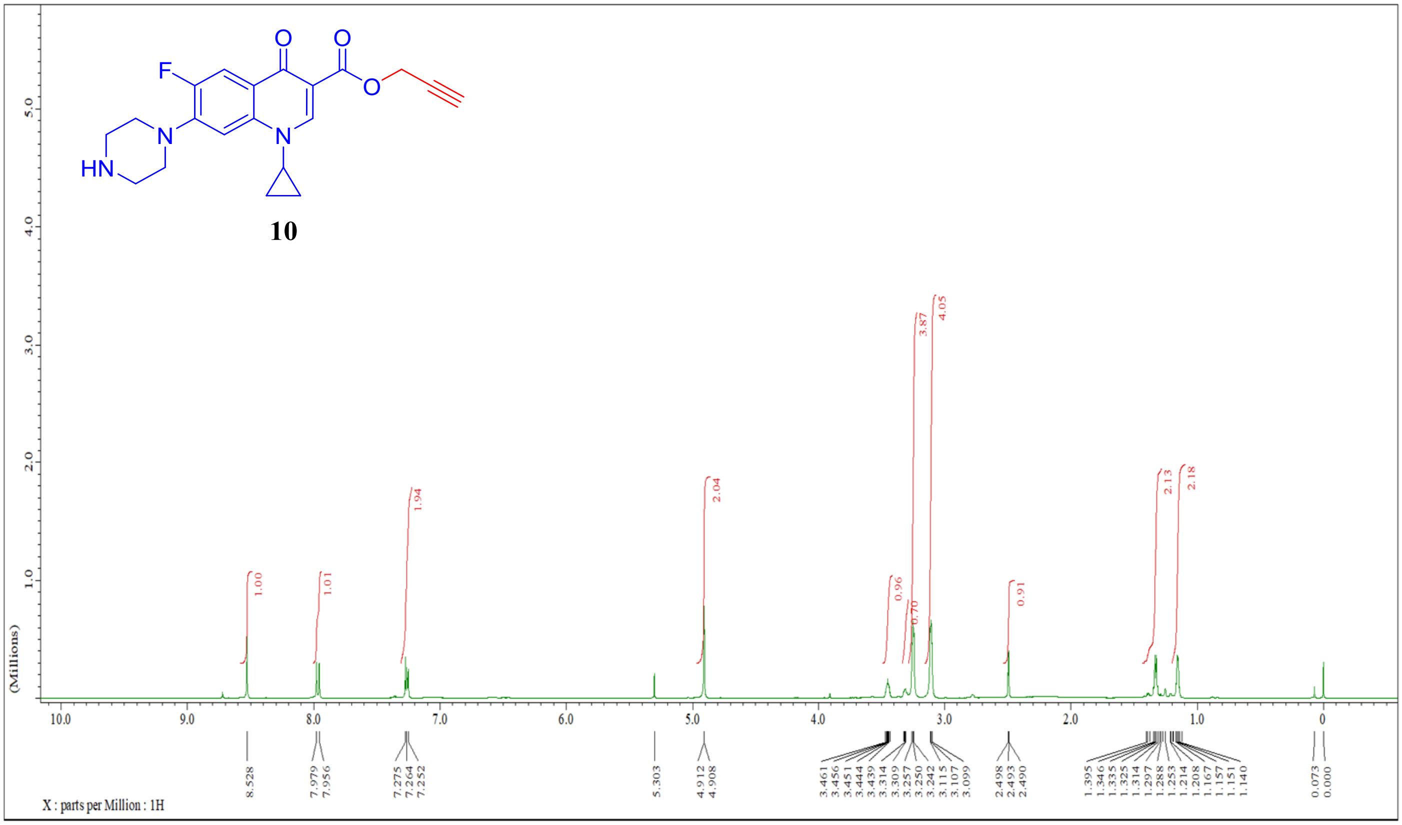

^{13}C-NMR spectrum of compound **10**

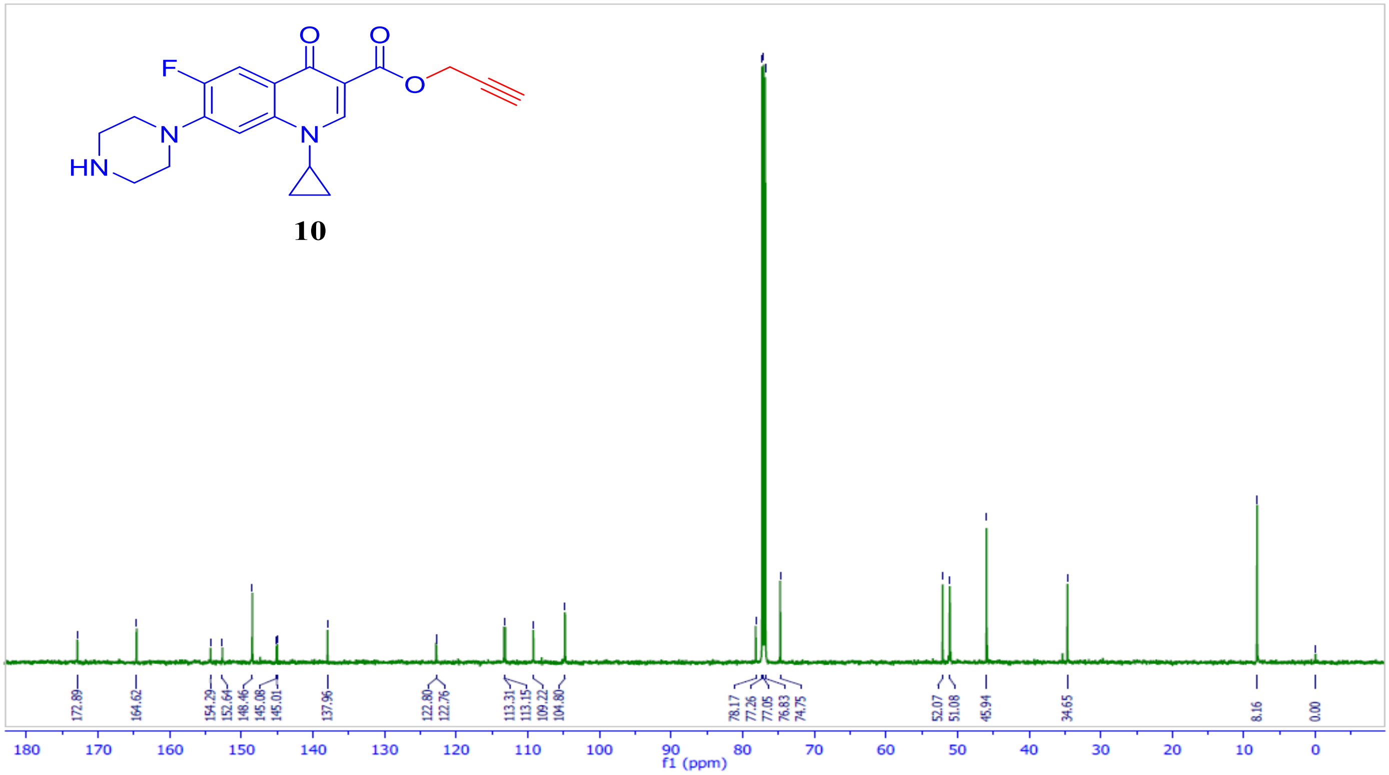

^{19}F-NMR spectrum of compound **10**

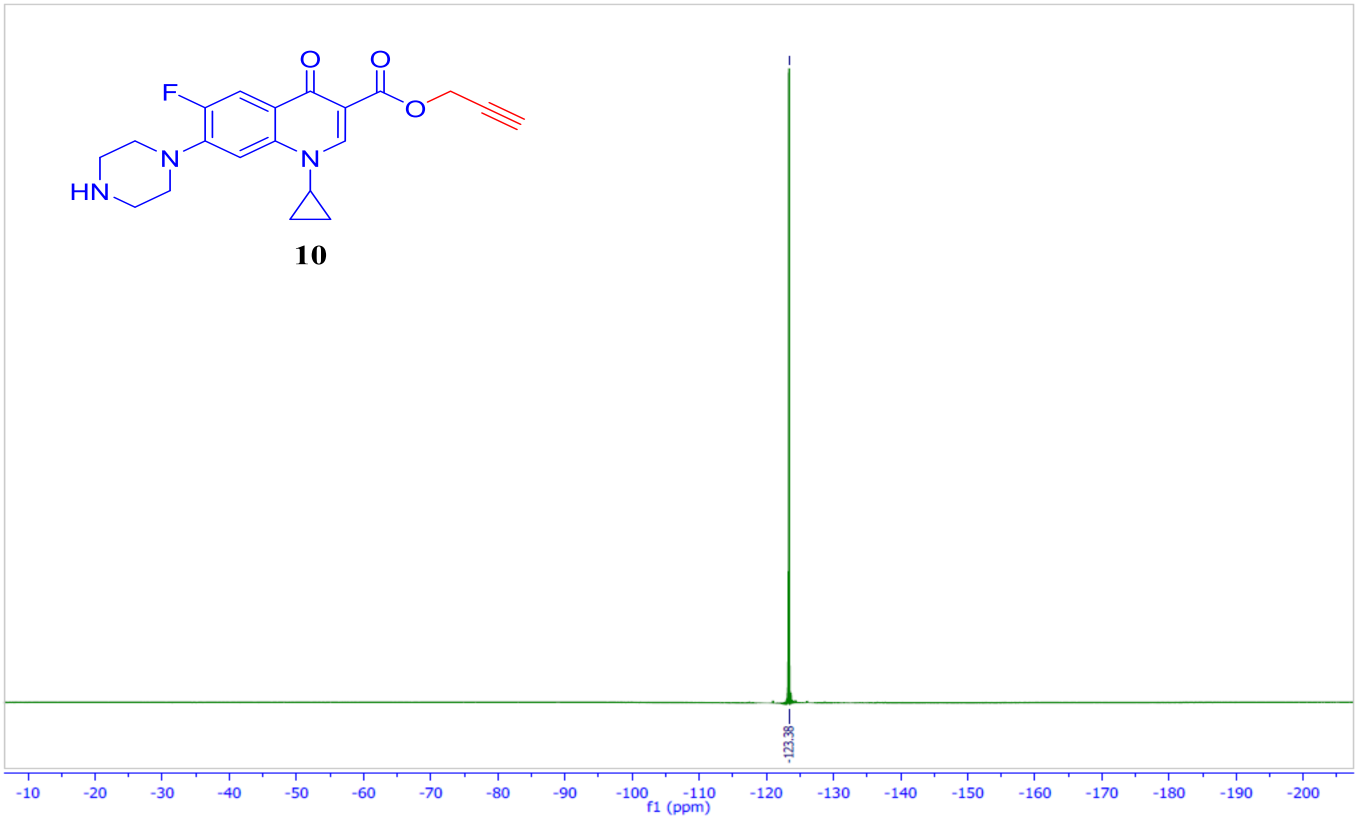

IR spectrum of compound **10**

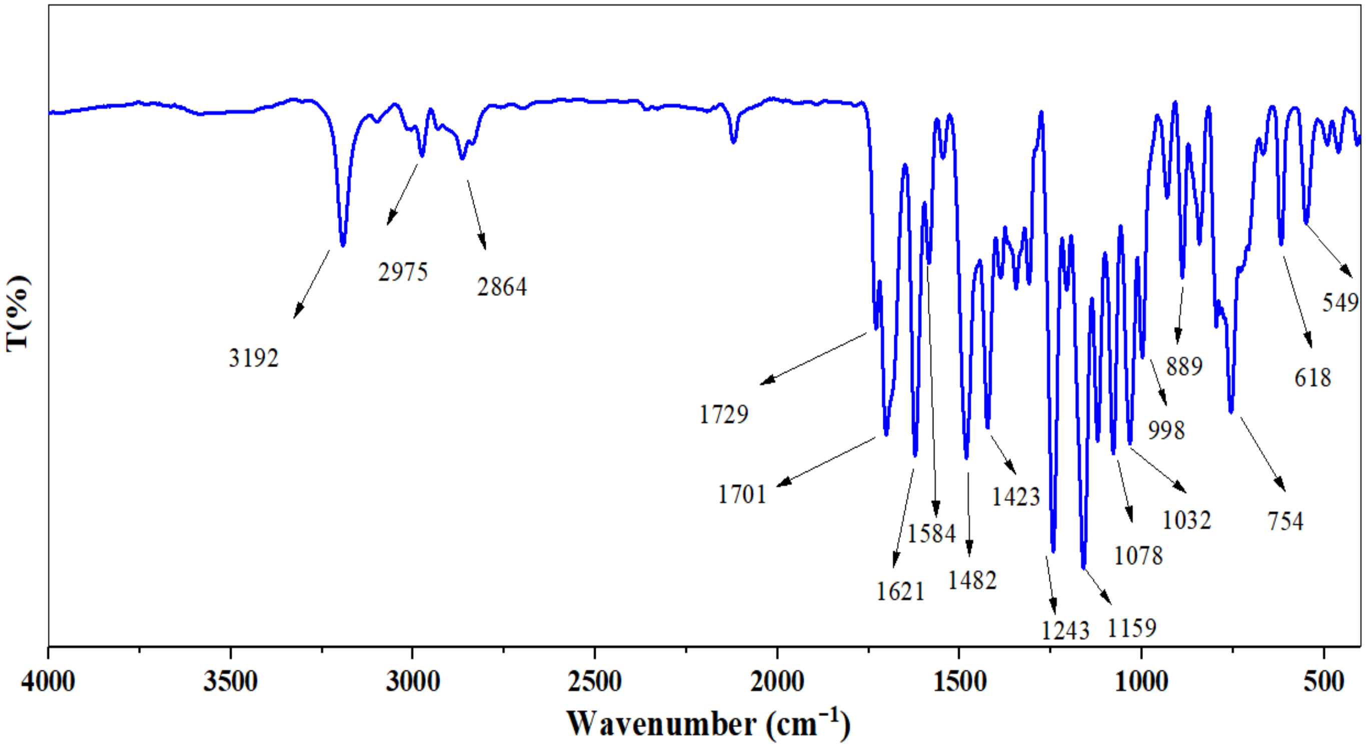

HRMS spectrum of compound **10**

**Data File**	CPR-10.d	**Sample Name**	CPR-10
**Sample Type**	Sample	**Position**	P1-E9
**Instrument Name**	Instrument 1	**User Name**	
**Acq Method**	MS Scan.m	**Acquired Time**	28-12-2022 17:12:39
**IRM Calibration Status**	Success	**DA Method**	Default.m
**Comment**			

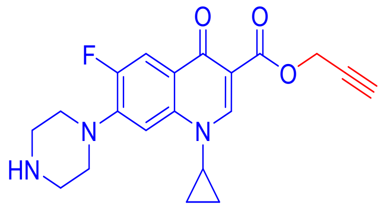

10

**Sample Group**		**Info.**	3
**Acquisition SW Version**	6200 series TOF/6500 series Q-TOF B.05.01 (B5125)		

Compound Table

Compound Label	RT	Mass	Abund	Formula	Tgt Mass	Diff (ppm)	MFG Formula	DB Formula
Cpd 1: C20 H20 F N3 O3	0.14	369.1509	86939	C20 H20 F N3 O3	369.1489	5.6	C20 H20 F N3 O3	C20 H20 F N3 O3

Compound Label	*m/z*	RT	Algorithm	Mass
Cpd 1: C20 H20 F N3 O3	392.1397	0.14	Find By Formula	369.1509

MS Zoomed Spectrum

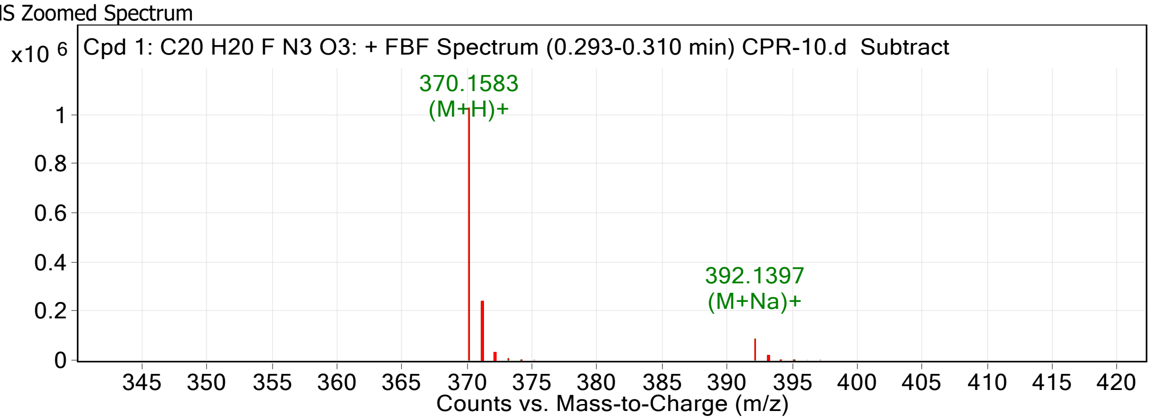

MS Spectrum Peak List

m/z	z	Abund	Formula	Ion
370.1583	1	1024152.25	C20H21FN3O3	(M+H)+
371.1613	1	241879.64	C20H21FN3O3	(M+H)+
372.1636	1	32302.36	C20H21FN3O3	(M+H)+
373.1653	1	3716.09	C20H21FN3O3	(M+H)+
374.16	1	635.4	C20H21FN3O3	(M+H)+
392.1397	1	86939.17	C20H20FN3NaO3	(M+Na)+
393.1429	1	18818.83	C20H20FN3NaO3	(M+Na)+
394.1443	1	2822.77	C20H20FN3NaO3	(M+Na)+
395.1512	1	334.69	C20H20FN3NaO3	(M+Na)+

--- End Of Report ---

^{1}H-NMR spectrum of compound **10a**

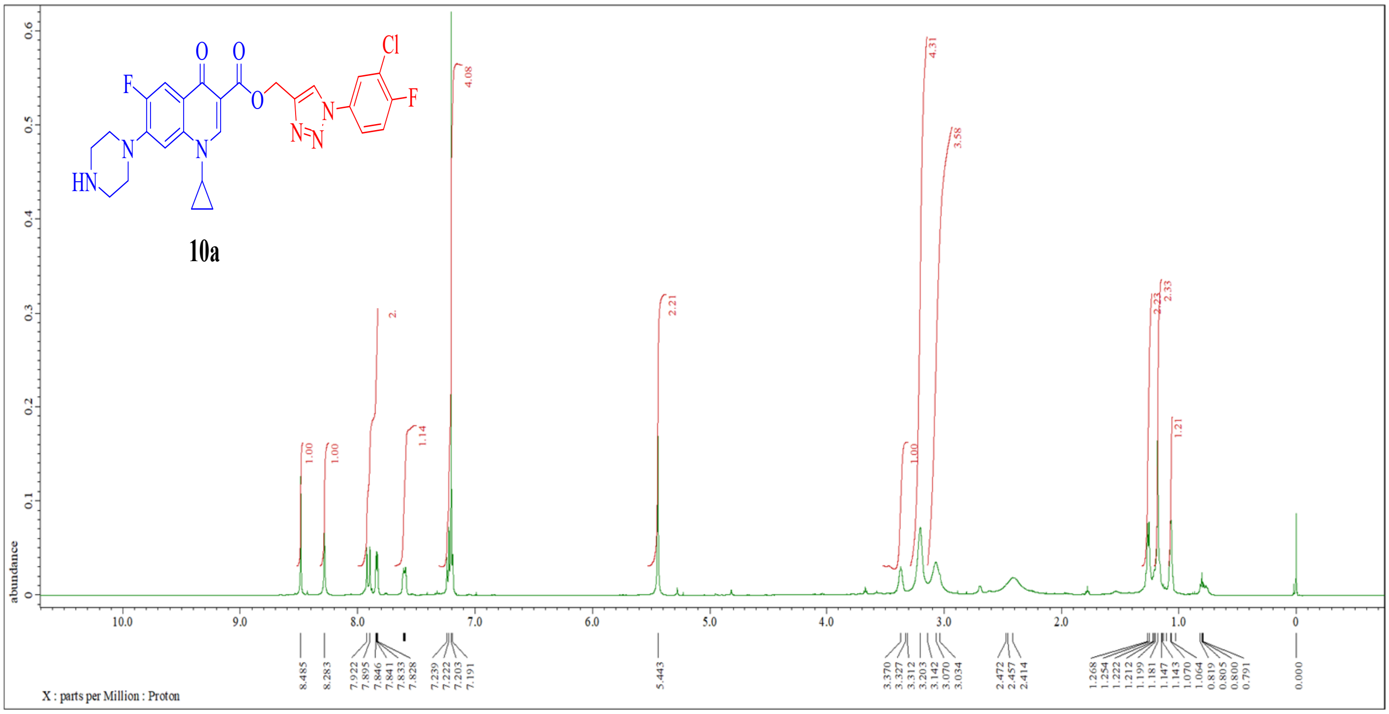

^{13}C-NMR spectrum of compound **10a**

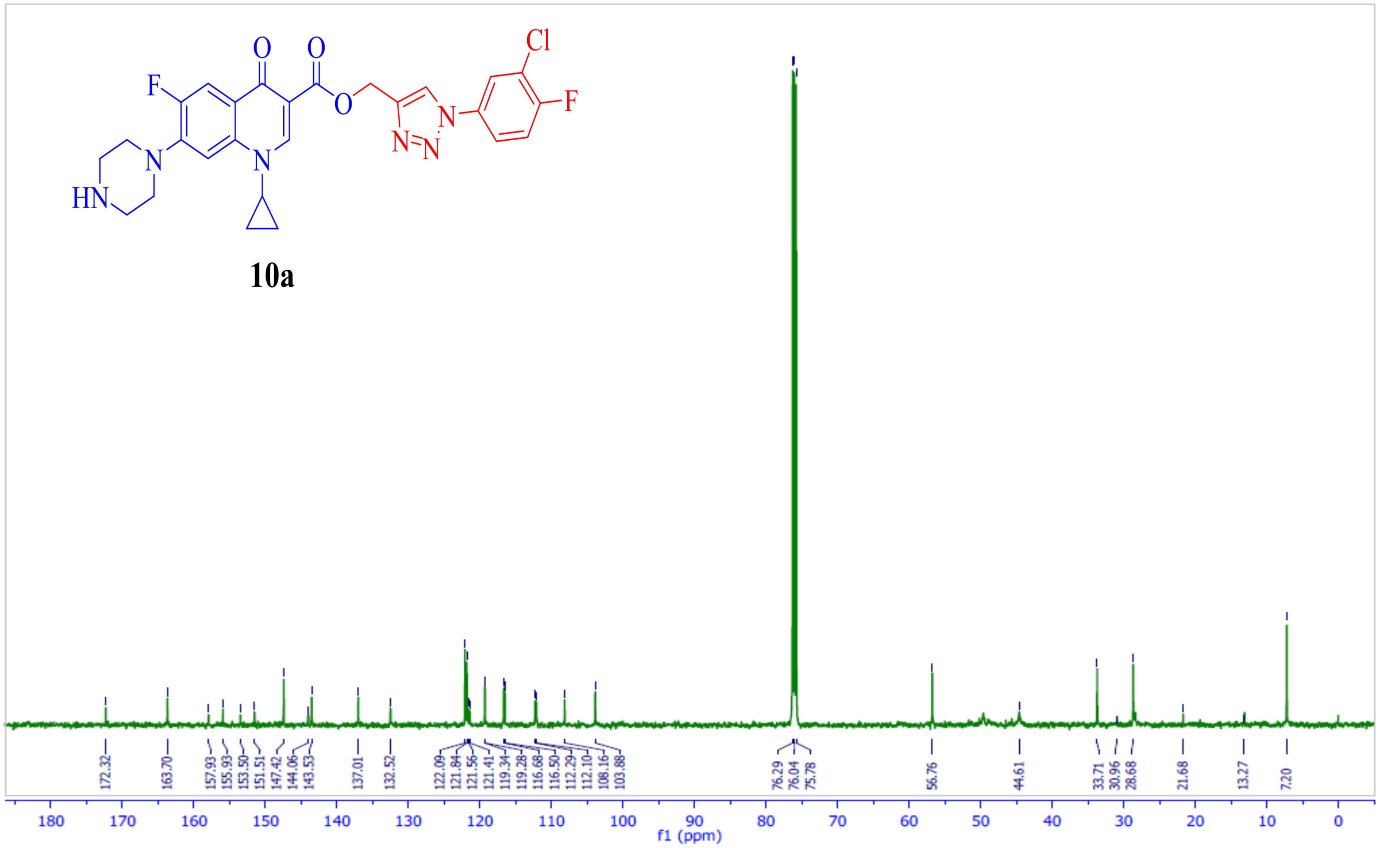

^{19}F-NMR spectrum of compound **10a**

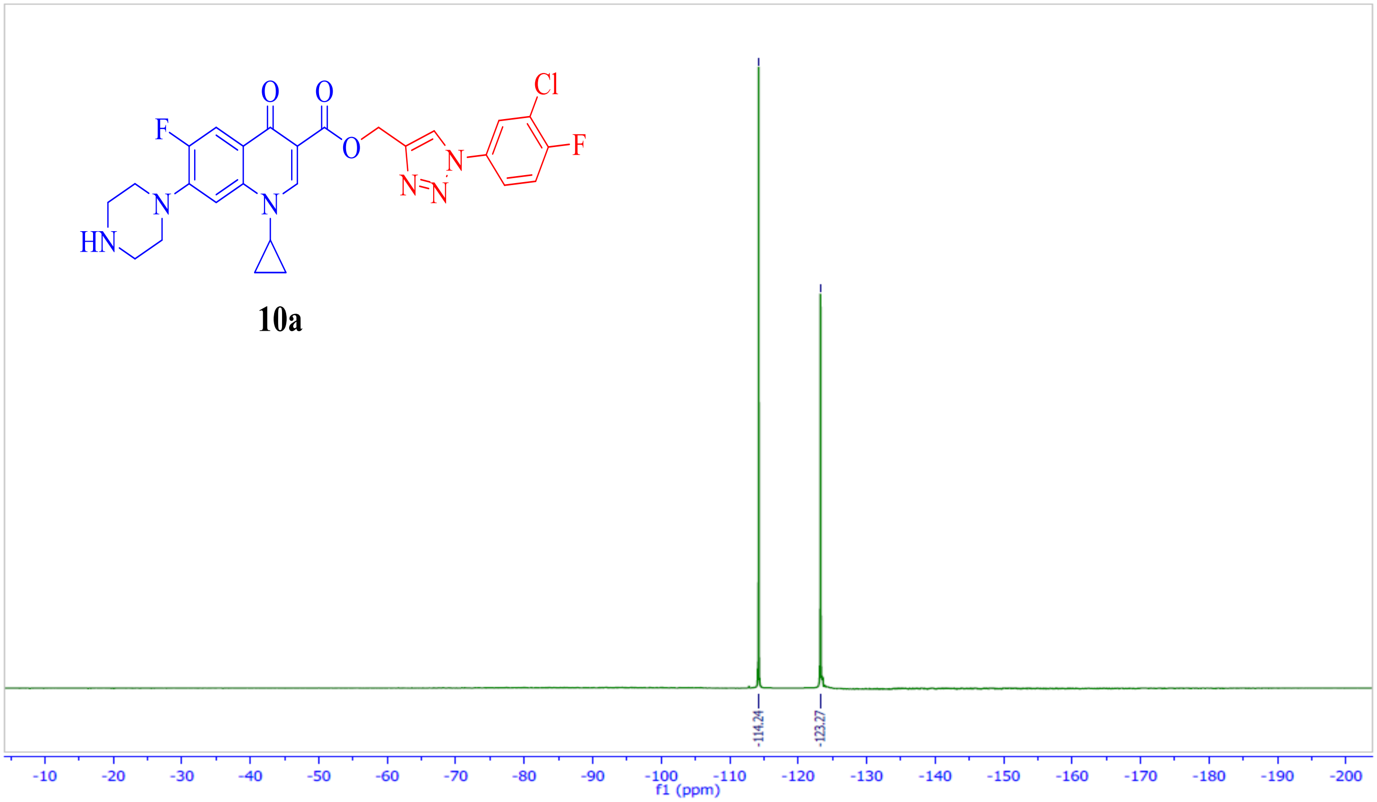

IR spectrum of compound **10a**

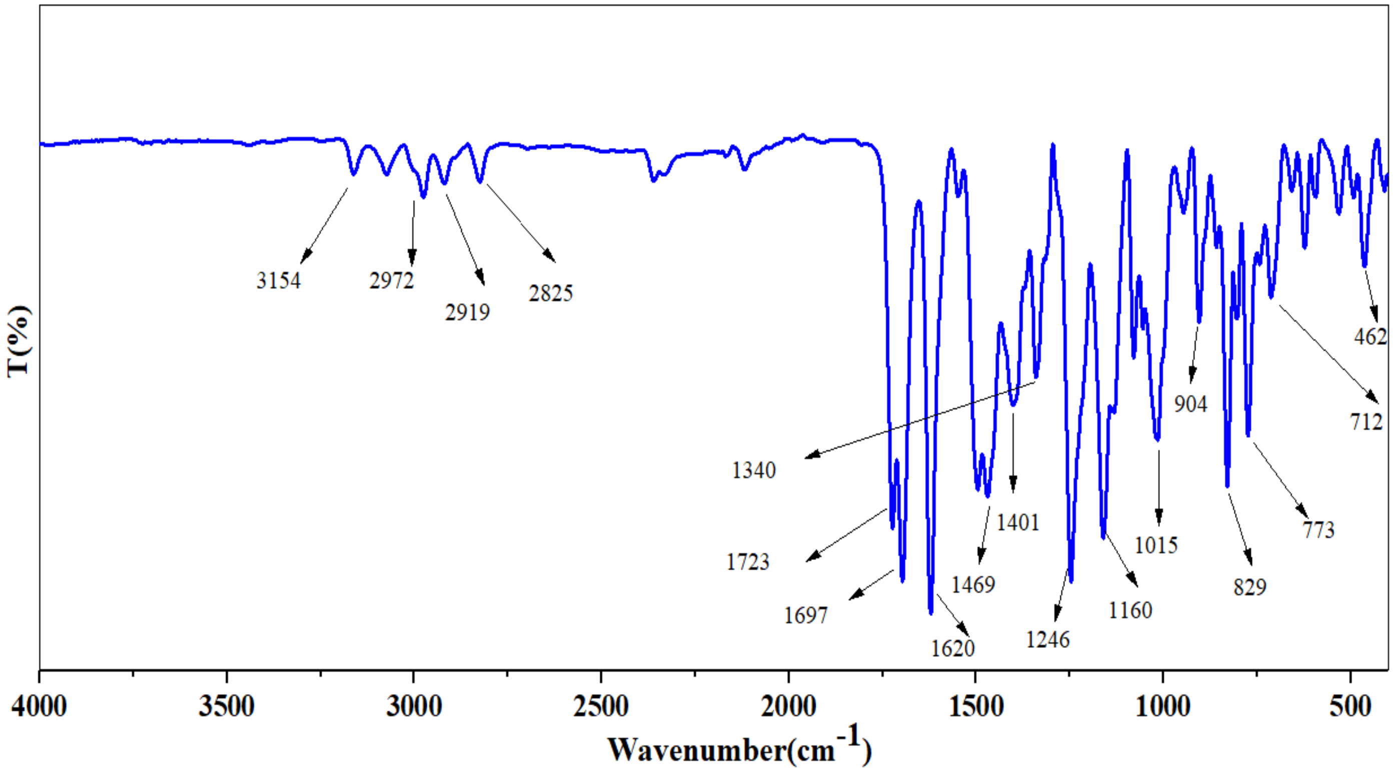

HRMS spectrum of compound **10a**

**Data File**	CPR-11.d	**Sample Name**	CPR-11
**Sample Type**	Sample	**Position**	P1-F1
**Instrument Name**	Instrument 1	**User Name**	
**Acq Method**	MS Scan.m	**Acquired Time**	28-12-2022 17:13:25
**IRM Calibration Status**	Success	**DA Method**	Default.m
**Comment**			

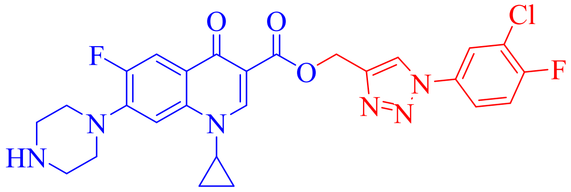

10a

**Sample Group**		**Info.**	3
**Acquisition SW Version**	6200 series TOF/6500 series Q-TOF B.05.01 (B5125)		

Compound Table

Compound Label	RT	Mass	Abund	Formula	Tgt Mass	Diff (ppm)	MFG Formula	DB Formula
Cpd 1: C26 H23 Cl F2N6 O3	0.142	540.1509	41520	C26 H23 Cl F2 N6 O3	540.1488	3.91	C26 H23 Cl F2 N6O3	C26 H23 Cl F2N6 O3

Compound Label	*m/z*	RT	Algorithm	Mass
Cpd 1: C26 H23 Cl F2 N6 O3	563.1396	0.142	Find By Formula	540.1509

MS Zoomed Spectrum

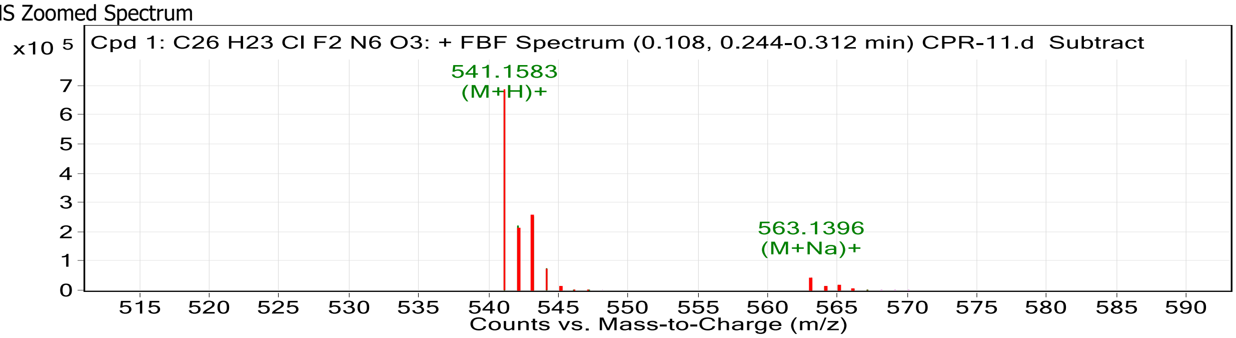

MS Spectrum Peak List

m/z	z	Abund	Formula	Ion
541.1583	1	678966.5	C26H24ClF2N6O3	(M+H)+
542.1611	1	220922.38	C26H24ClF2N6O3	(M+H)+
543.1566	1	251685.33	C26H24ClF2N6O3	(M+H)+
544.1583	1	71574.27	C26H24ClF2N6O3	(M+H)+
545.1601	1	11766.89	C26H24ClF2N6O3	(M+H)+
546.1605	1	1391.89	C26H24ClF2N6O3	(M+H)+
547.1576	1	356.16	C26H24ClF2N6O3	(M+H)+
563.1396	1	41520.03	C26H23ClF2N6NaO3	(M+Na)+
564.1423	1	12342.22	C26H23ClF2N6NaO3	(M+Na)+
565.1376	1	14865.63	C26H23ClF2N6NaO3	(M+Na)+
566.1406	1	4344.81	C26H23ClF2N6NaO3	(M+Na)+
567.1353	1	970.44	C26H23ClF2N6NaO3	(M+Na)+

--- End Of Report --

^{1}H-NMR spectrum of compound**10b**

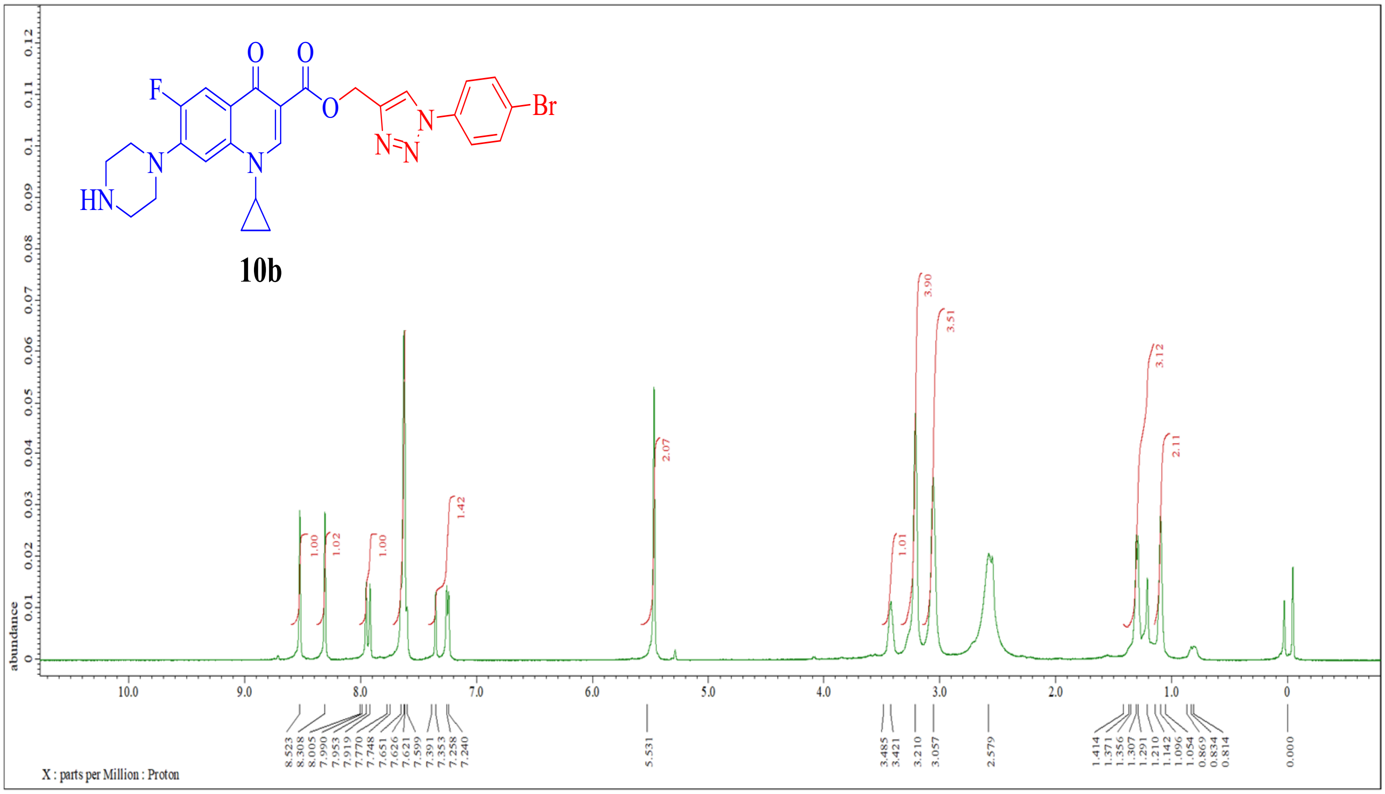

^{13}C-NMR spectrum of compound **10b**

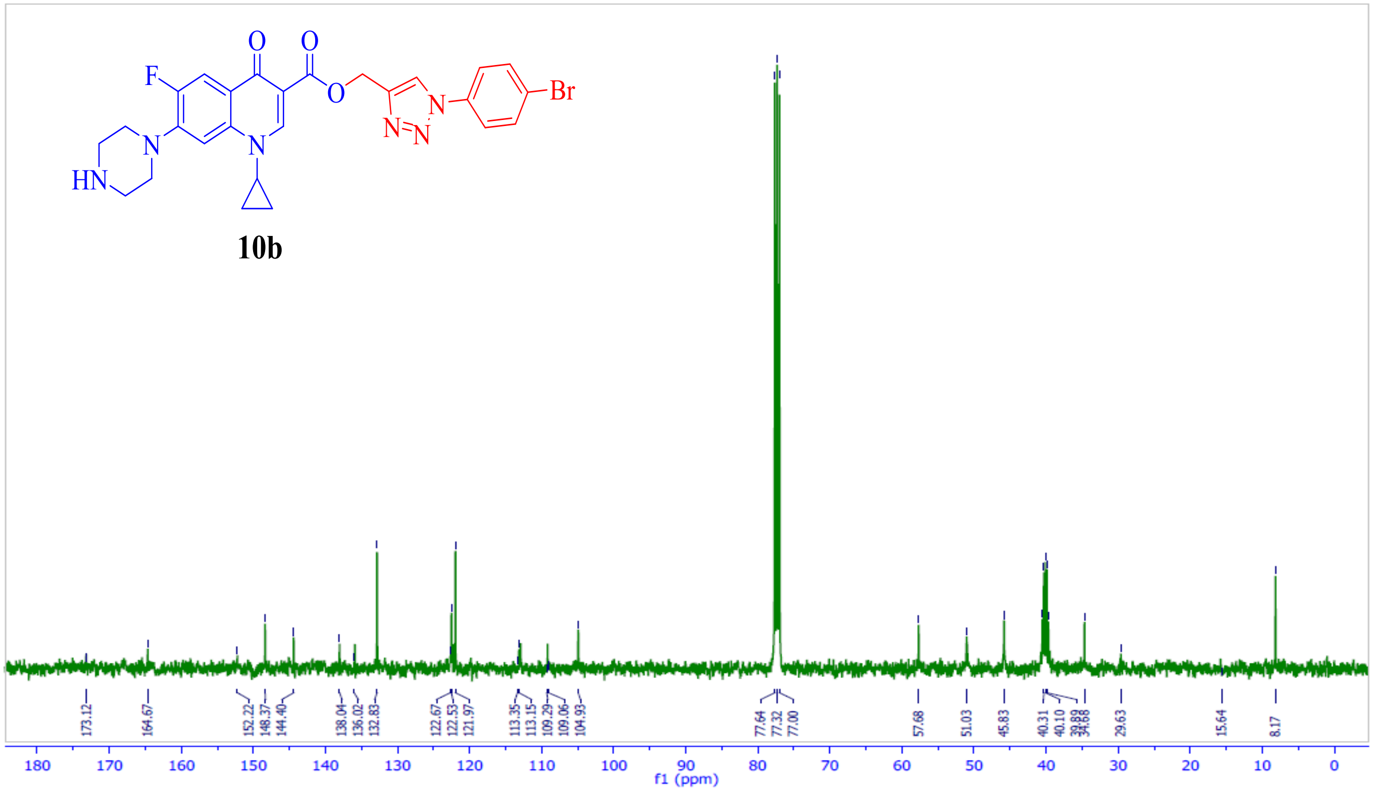

IR spectrum of compound **10b**

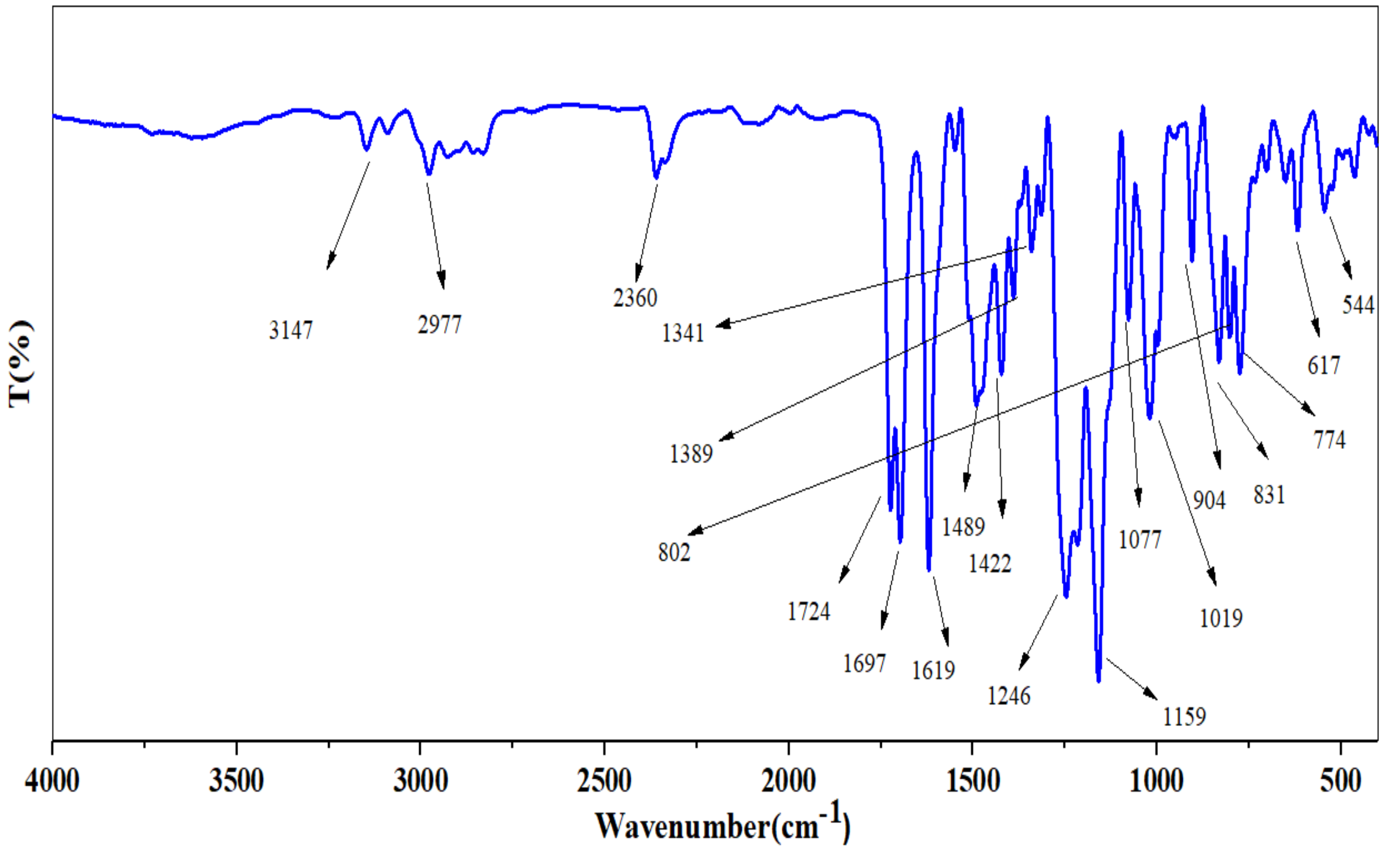

HRMS spectrum of compound **10b**

**Data File**	CPR-012.d	**Sample Name**	CPR-012
**Sample Type**	Sample	**Position**	P1-A4
**Instrument Name**	Instrument 1	**User Name**	
**Acq Method**	MS Scan.m	**Acquired Time**	29-12-2022 12:43:56
**IRM Calibration Status**	Success	**DA Method**	Default.m
**Comment**			

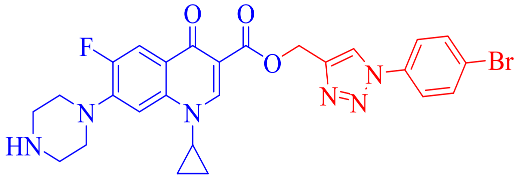

10b

**Sample Group**		**Info.**	3
**Acquisition SW Version**	6200 series TOF/6500 series Q-TOF B.05.01 (B5125)		

Compound Table

Compound Label	RT	Mass	Abund	Formula	Tgt Mass	Diff (ppm)	MFG Formula	DB Formula
Cpd 1: C26 H24 Br FN6O3	0.179	566.1097	34994	C26 H24 Br F N6 O3	566.1077	3.56	C26 H24 Br F N6 O3	C26 H24 Br F N6 O3

Compound Label	*m/z*	RT	Algorithm	Mass
Cpd 1: C26 H24 Br F N6 O3	589.0982	0.179	Find By Formula	566.1097

MS Zoomed Spectrum

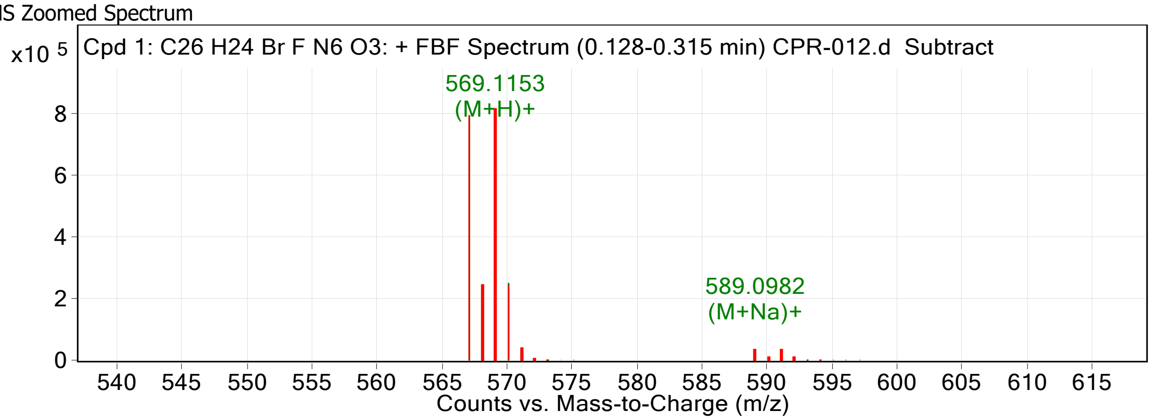

MS Spectrum Peak List

m/z	z	Abund	Formula	Ion
567.1171	1	789732.19	C26H25BrFN6O3	(M+H)+
568.1202	1	245289.77	C26H25BrFN6O3	(M+H)+
569.1153	1	813532.44	C26H25BrFN6O3	(M+H)+
570.1183	1	246250.13	C26H25BrFN6O3	(M+H)+
571.1199	1	40486.55	C26H25BrFN6O3	(M+H)+
572.1206	1	4604.6	C26H25BrFN6O3	(M+H)+
573.1203	1	691.86	C26H25BrFN6O3	(M+H)+
589.0982	1	34994.23	C26H24BrFN6NaO3	(M+Na)+
590.1011	1	10235.79	C26H24BrFN6NaO3	(M+Na)+
591.0967	1	34880.57	C26H24BrFN6NaO3	(M+Na)+
592.099	1	10204.31	C26H24BrFN6NaO3	(M+Na)+
593.1005	1	1890.17	C26H24BrFN6NaO3	(M+Na)+
594.1004	1	400.86	C26H24BrFN6NaO3	(M+Na)+

--- End Of Report ---

^{1}H-NMR spectrum of compound **10c**

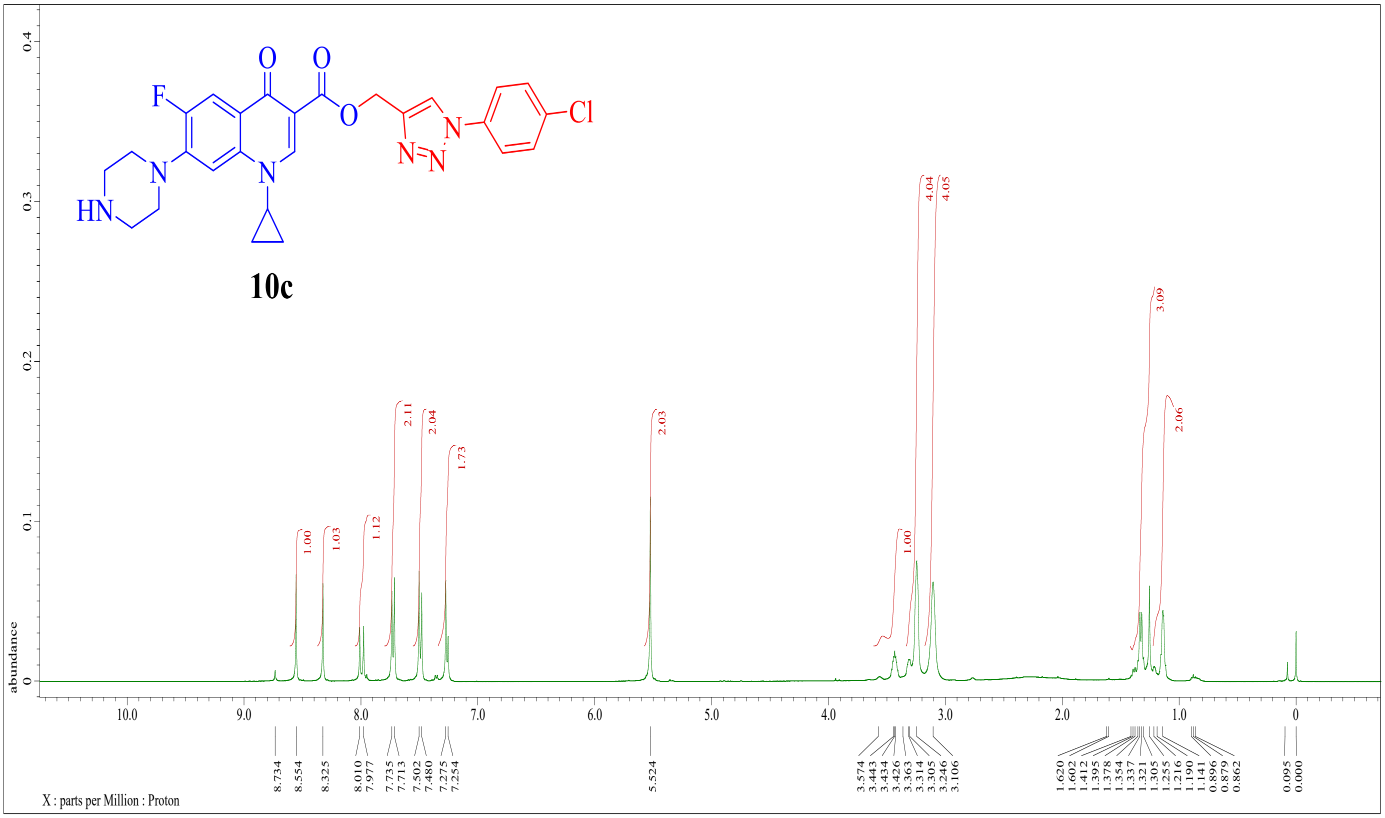

^{13}C-NMR spectrum of compound **10c**

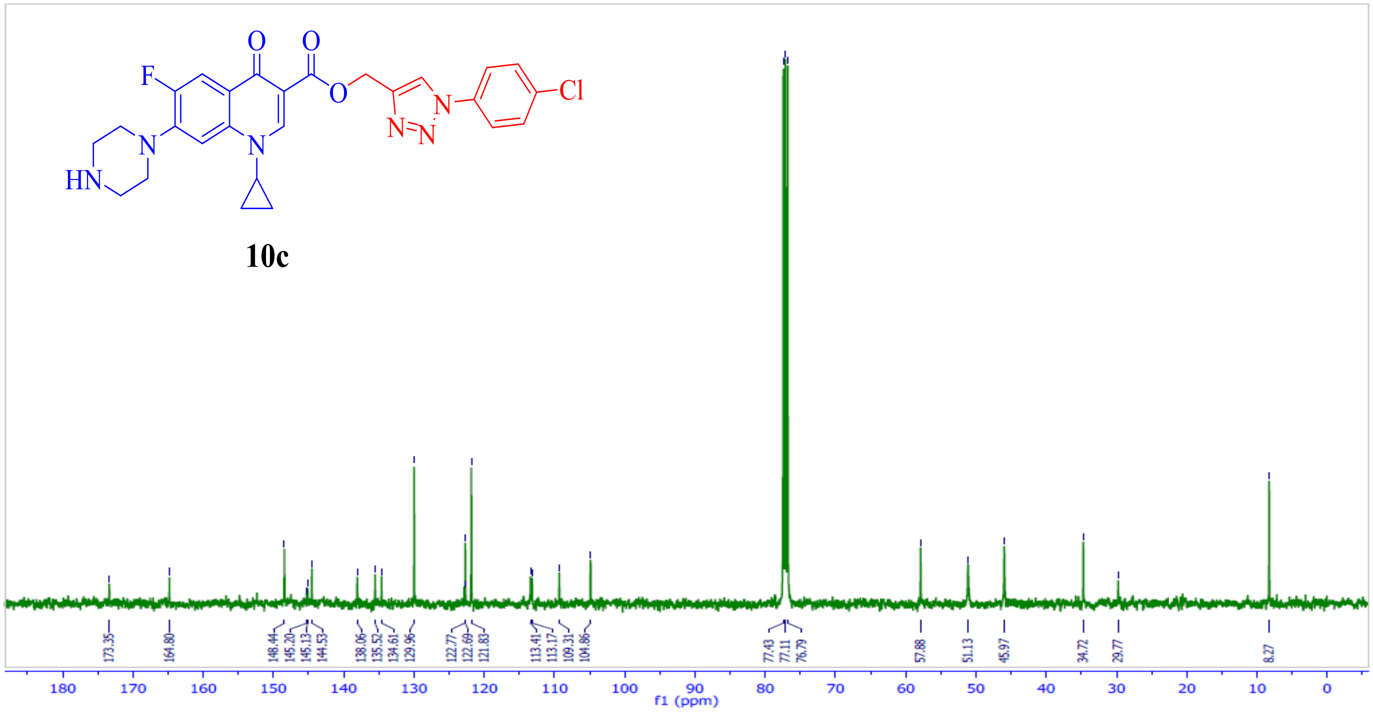

IR spectrum of compound **10c**

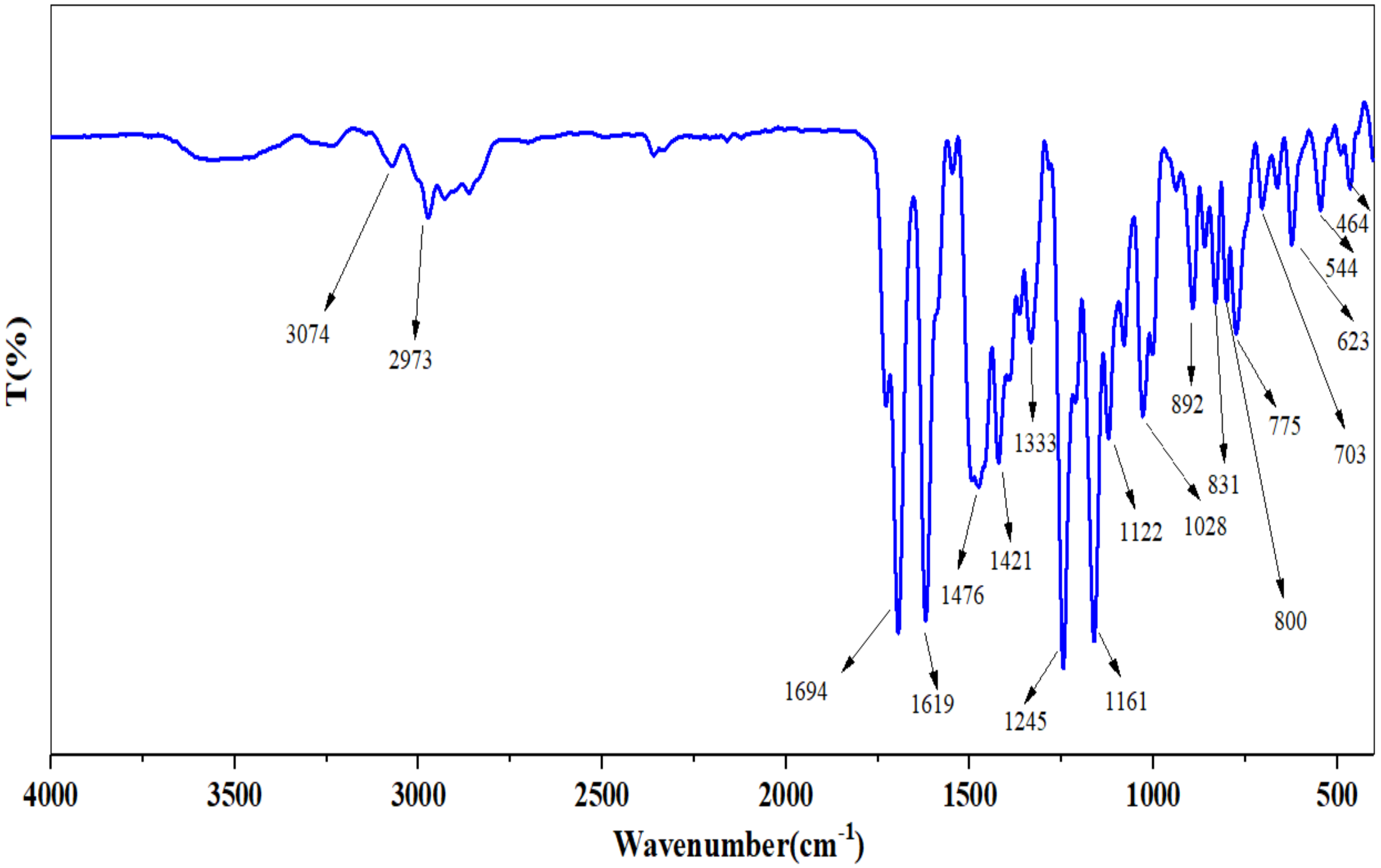

HRMS spectrum of compound **10c**

**Data File**	CPR-13.d	**Sample Name**	CPR-13
**Sample Type**	Sample	**Position**	P1-A5
**Instrument Name**	Instrument 1	**User Name**	
**Acq Method**	MS Scan.m	**Acquired Time**	29-12-2022 12:46:40
**IRM Calibration Status**	Success	**DA Method**	Default.m
**Comment**			

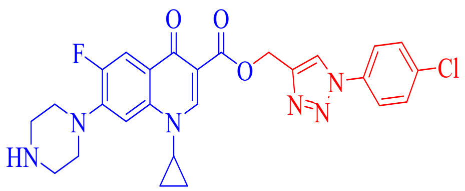

10c

**Sample Group**		**Info.**	3
**Acquisition SW Version**	6200 series TOF/6500 series Q-TOF B.05.01 (B5125)		

Compound Table

Compound Label	RT	Mass	Abund	Formula	Tgt Mass	Diff (ppm)	MFG Formula	DB Formula
Cpd 1: C26 H24 Cl F N6 O3	0.156	522.1611	43676	C26 H24 Cl F N6O3	522.1582	5.51	C26 H24 Cl F N6 O3	C26 H24 Cl F N6O3

Compound Label	*m/z*	RT	Algorithm	Mass
Cpd 1: C26 H24 Cl F N6 O3	545.1495	0.156	Find By Formula	522.1611

MS Zoomed Spectrum

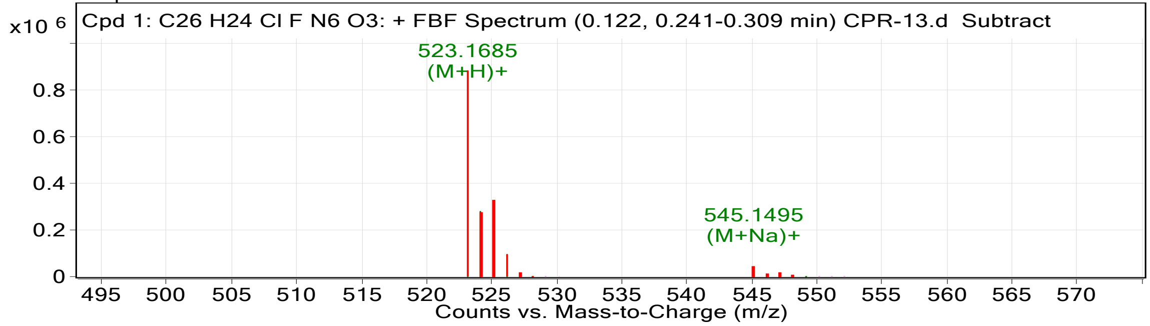

MS Spectrum Peak List

m/z	z	Abund	Formula	Ion
523.1685	1	879885.56	C26H25ClFN6O3	(M+H)+
524.1714	1	277010.97	C26H25ClFN6O3	(M+H)+
525.1668	1	327758.5	C26H25ClFN6O3	(M+H)+
526.1684	1	93453.57	C26H25ClFN6O3	(M+H)+
527.1701	1	15023.49	C26H25ClFN6O3	(M+H)+
528.1713	1	1774.71	C26H25ClFN6O3	(M+H)+
545.1495	1	43676.43	C26H24ClFN6NaO3	(M+Na)+
546.1528	1	12872.21	C26H24ClFN6NaO3	(M+Na)+
547.1478	1	15333.61	C26H24ClFN6NaO3	(M+Na)+
548.1499	1	4110.7	C26H24ClFN6NaO3	(M+Na)+
549.1527	1	854.57	C26H24ClFN6NaO3	(M+Na)+

--- End Of Report ---

^{1}H-NMR spectrum of compound **10d**

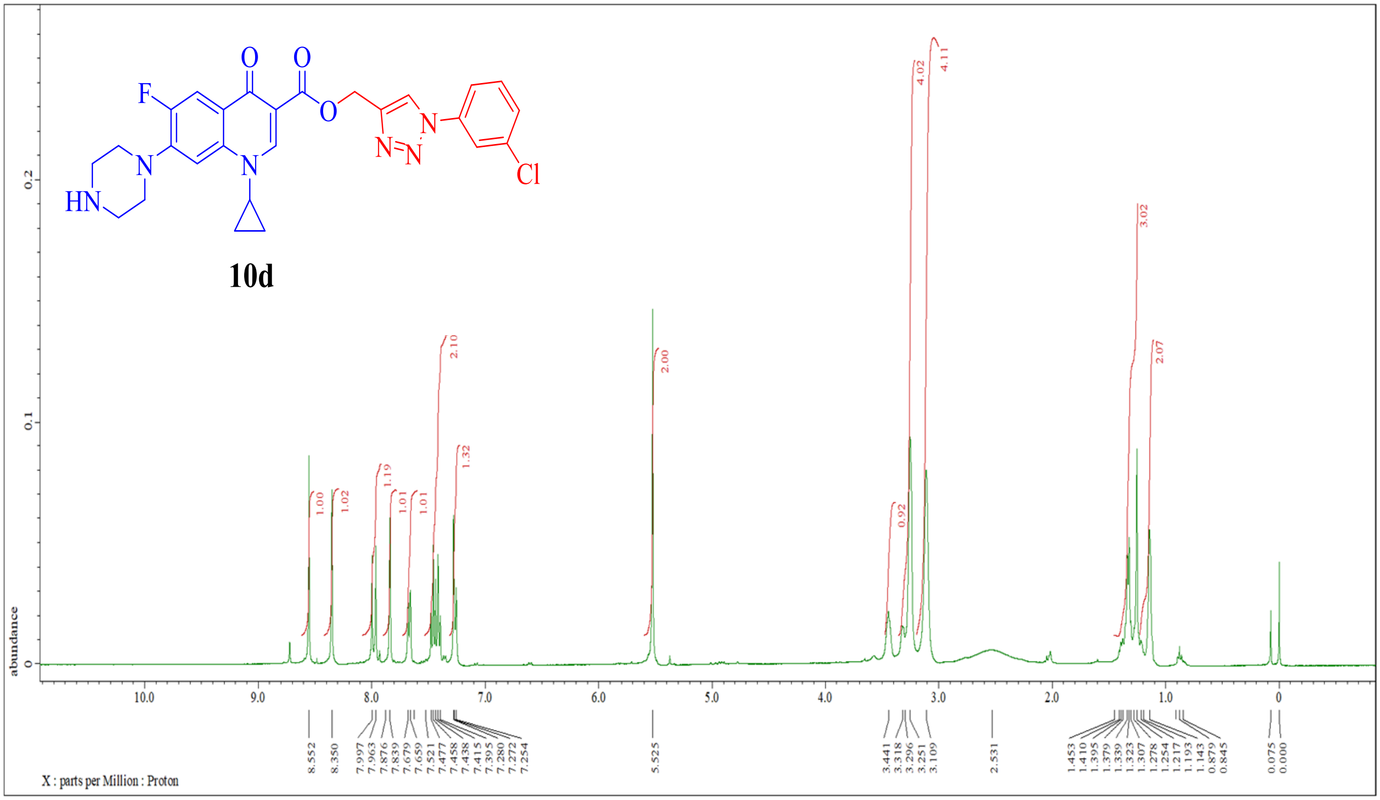

^{13}C-NMR spectrum of compound **10d**

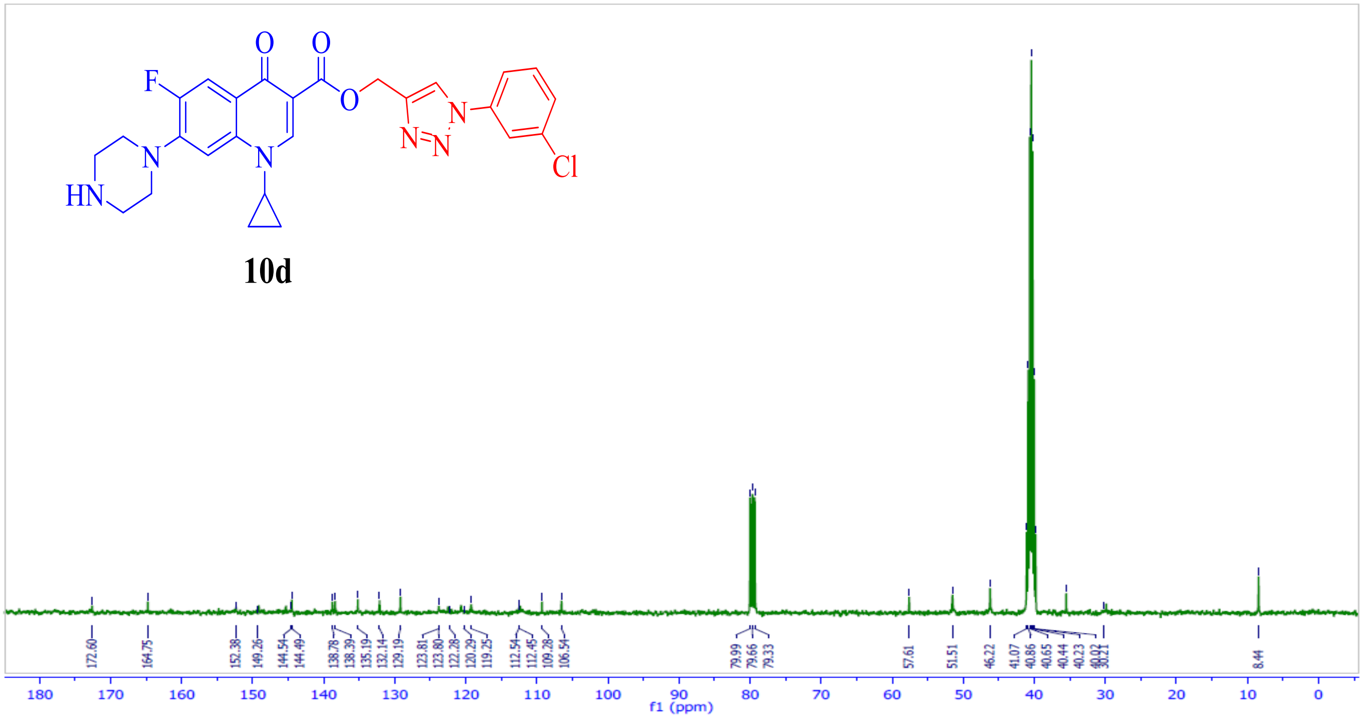

HRMS spectrum of compound **10d**

**Data File**	CPR-14.d	**Sample Name**	CPR-14
**Sample Type**	Sample	**Position**	P1-A6
**Instrument Name**	Instrument 1	**User Name**	
**Acq Method**	MS Scan.m	**Acquired Time**	29-12-2022 12:51:25
**IRM Calibration Status**	Success	**DA Method**	Default.m
**Comment**			

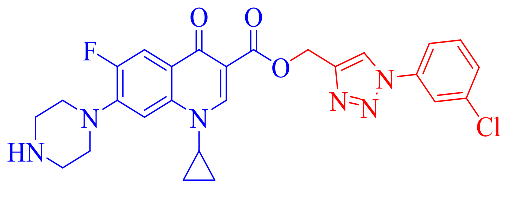

10d

**Sample Group**		**Info.**	3
**Acquisition SW Version**	6200 series TOF/6500 series Q-TOF B.05.01 (B5125)		

Compound Table

Compound Label	RT	Mass	Abund	Formula	Tgt Mass	Diff (ppm)	MFG Formula	DB Formula
Cpd 1: C26 H24 Cl F N6 O3	0.186	522.1606	45346	C26 H24 Cl F N6 O3	522.1582	4.53	C26 H24 Cl F N6O3	C26 H24 Cl F N6 O3

Compound Label	*m/z*	RT	Algorithm	Mass
Cpd 1: C26 H24 Cl F N6 O3	545.149	0.186	Find By Formula	522.1606

MS Zoomed Spectrum

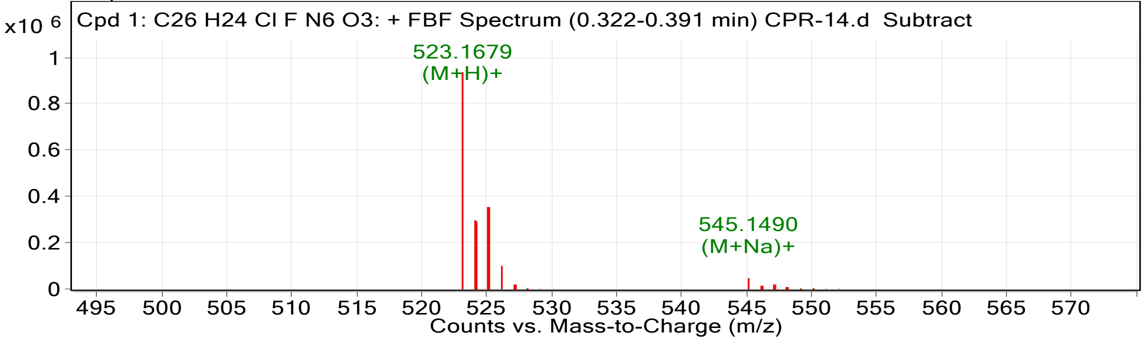

MS Spectrum Peak List

m/z	z	Abund	Formula	Ion
523.1679	1	931869.81	C26H25ClFN6O3	(M+H)+
524.1709	1	293209.81	C26H25ClFN6O3	(M+H)+
525.1664	1	349703.53	C26H25ClFN6O3	(M+H)+
526.1679	1	97192.2	C26H25ClFN6O3	(M+H)+
527.1695	1	15739	C26H25ClFN6O3	(M+H)+
528.1701	1	1794.84	C26H25ClFN6O3	(M+H)+
545.149	1	45345.54	C26H24ClFN6NaO3	(M+Na)+
546.1521	1	13486.66	C26H24ClFN6NaO3	(M+Na)+
547.1471	1	16223.66	C26H24ClFN6NaO3	(M+Na)+
548.149	1	4453.62	C26H24ClFN6NaO3	(M+Na)+
549.1542	1	877.39	C26H24ClFN6NaO3	(M+Na)+
550.152	1	233.16	C26H24ClFN6NaO3	(M+Na)+

--- End Of Report ---

^{1}H-NMR spectrum of compound **10e**

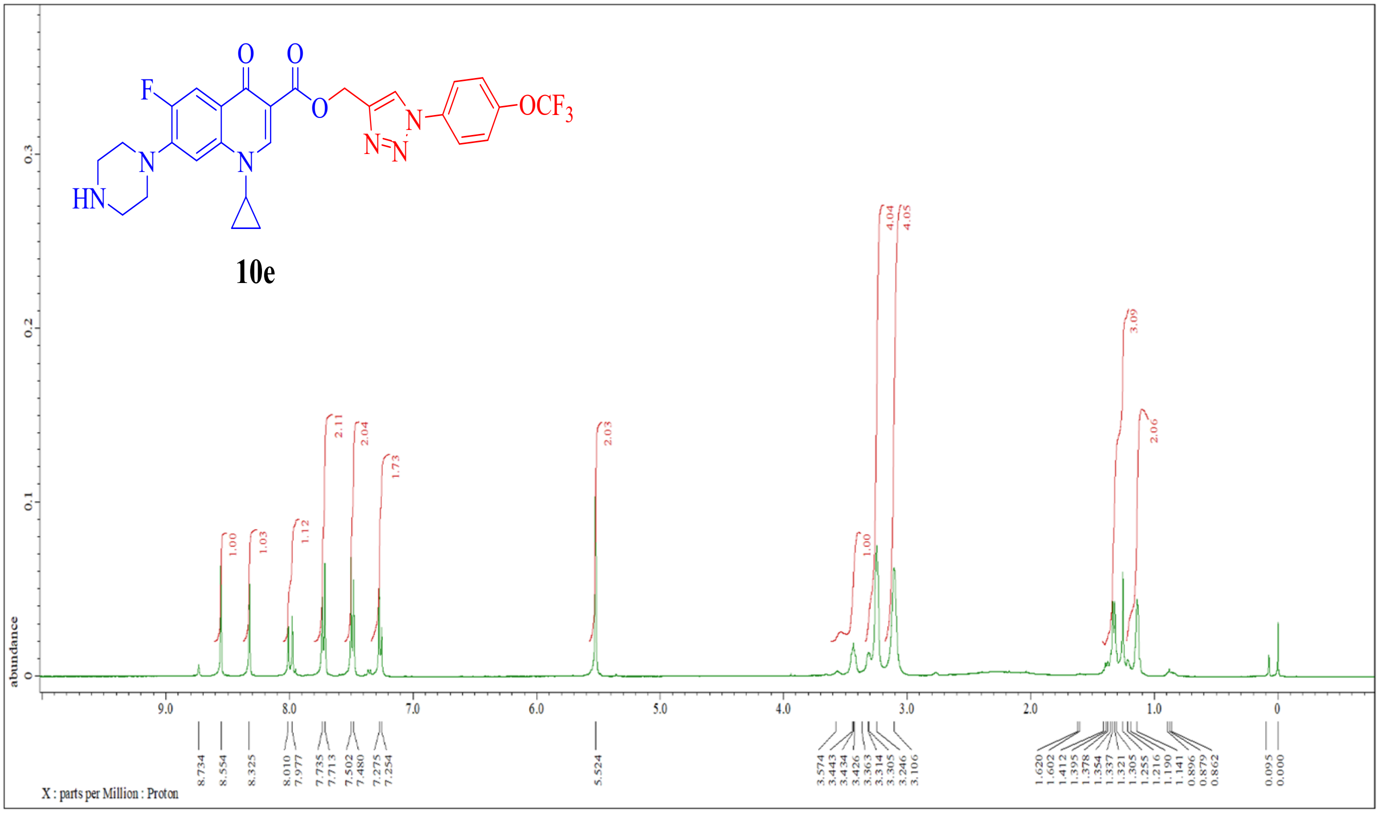

^{13}C-NMR spectrum of compound**10e**

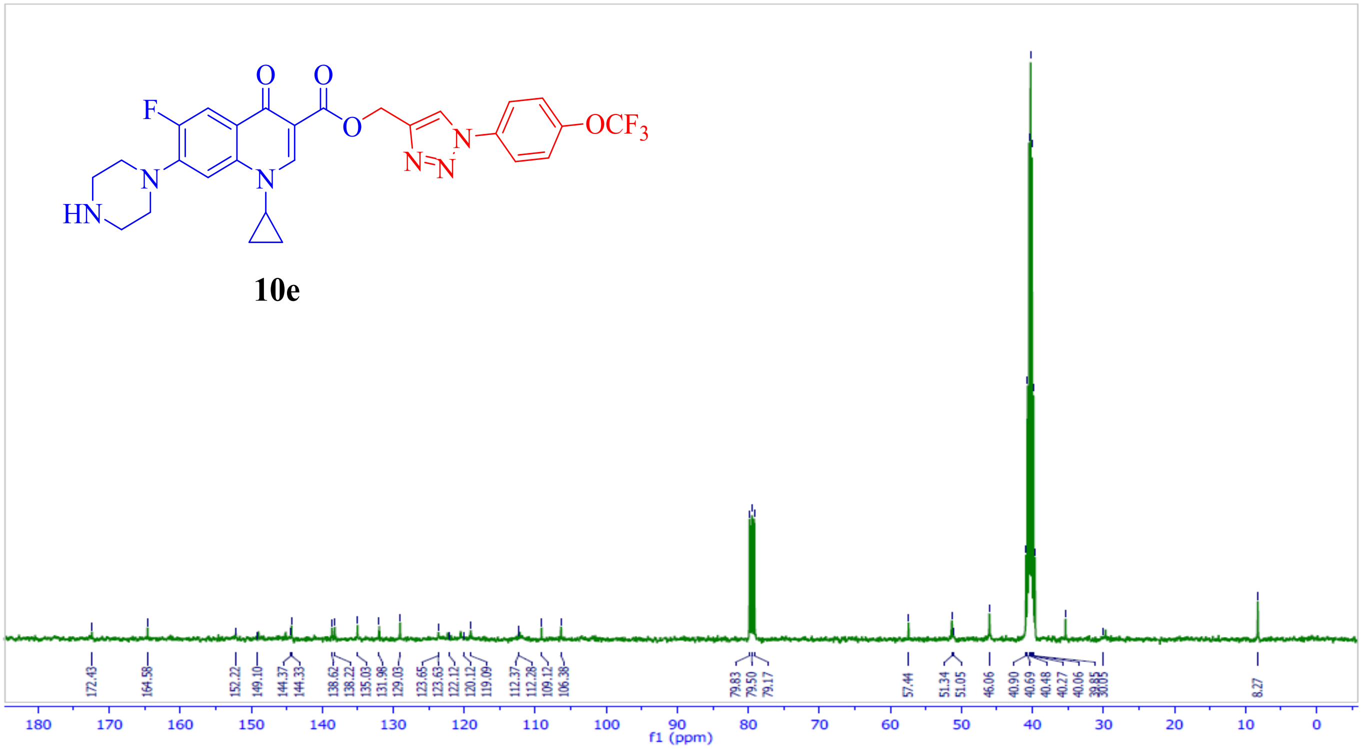

^{19}F-NMR spectrum of compound **10e**

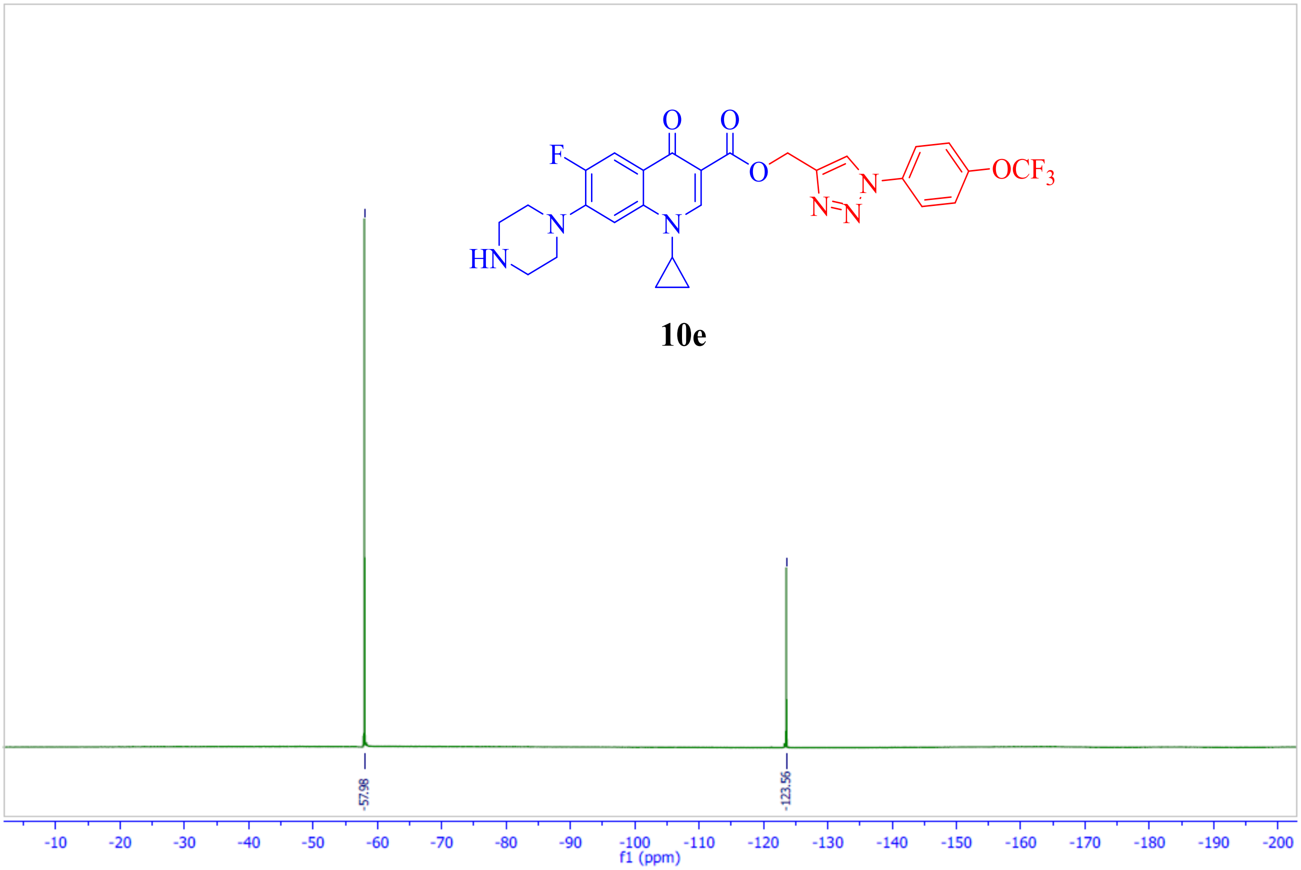

HRMS spectrum of compound**10e**

**Data File**	CPR-15.d	**Sample Name**	CPR-15
**Sample Type**	Sample	**Position**	P1-C1
**Instrument Name**	Instrument 1	**User Name**	
**Acq Method**	MS Scan.m	**Acquired Time**	24-05-2023 13:47:49
**IRM Calibration Status**	Success	**DA Method**	Default.m
**Comment**			

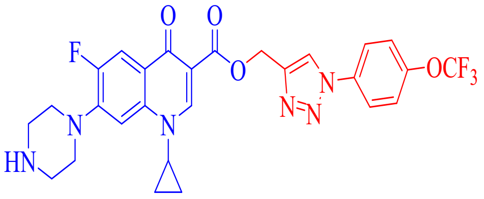

10e

Sample Group | **Info.** 3

Acquisition SW Version 6200 series TOF/6500 series Q-TOF B.05.01 (B5125)

Compound Table

Compound Label	RT	Mass	Abund	Formula	Tgt Mass	Diff (ppm)	MFG Formula	DB Formula
Cpd 1: C27 H24 F4 N6 O4	0.121	572.1774	110998	C27 H24 F4 N6 O4	572.1795	-3.69	C27 H24 F4 N6 O4	C27 H24 F4 N6 O4

Compound Label	*m/z*	RT	Algorithm	Mass
Cpd 1: C27 H24 F4 N6 O4	573.1847	0.121	Find By Formula	572.1774

MS Zoomed Spectrum

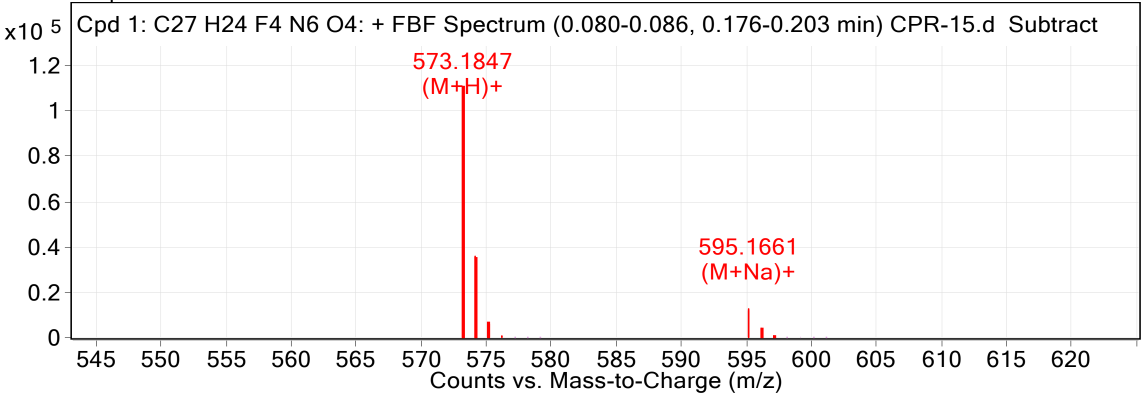

MS Spectrum Peak List

m/z	z	Abund	Formula	Ion
573.1847	1	110997.91	C27H25F4N6O4	(M+H)+
574.188	1	35606.68	C27H25F4N6O4	(M+H)+
575.1893	1	6232.95	C27H25F4N6O4	(M+H)+
576.191	1	822.14	C27H25F4N6O4	(M+H)+
595.1661	1	12344.87	C27H24F4N6NaO4	(M+Na)+
596.1689	1	3768.71	C27H24F4N6NaO4	(M+Na)+
597.171	1	697.37	C27H24F4N6NaO4	(M+Na)+

--- End Of Report ---

^{1}H-NMR spectrum of compound **10f**

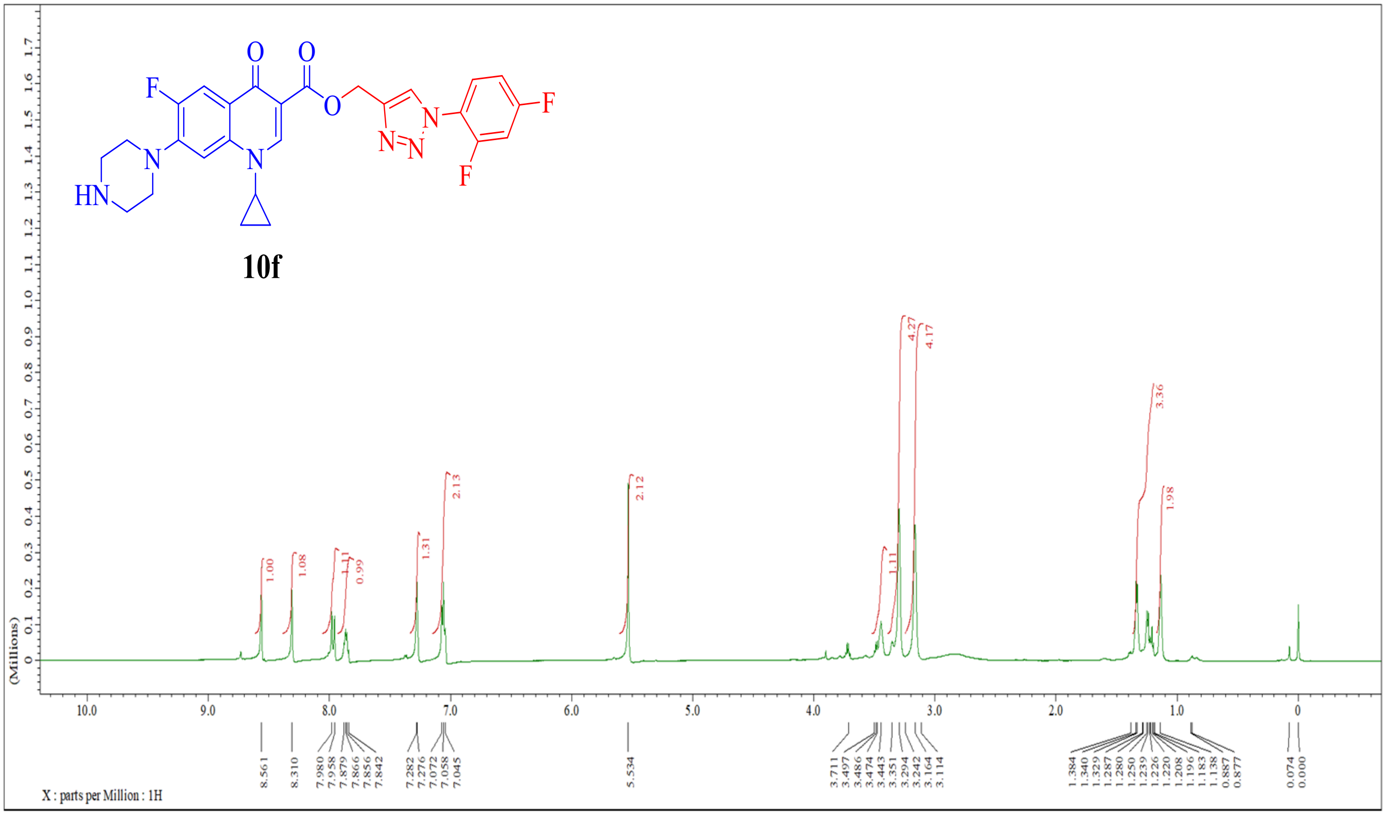

^{13}C-NMR spectrum of compound**10f**

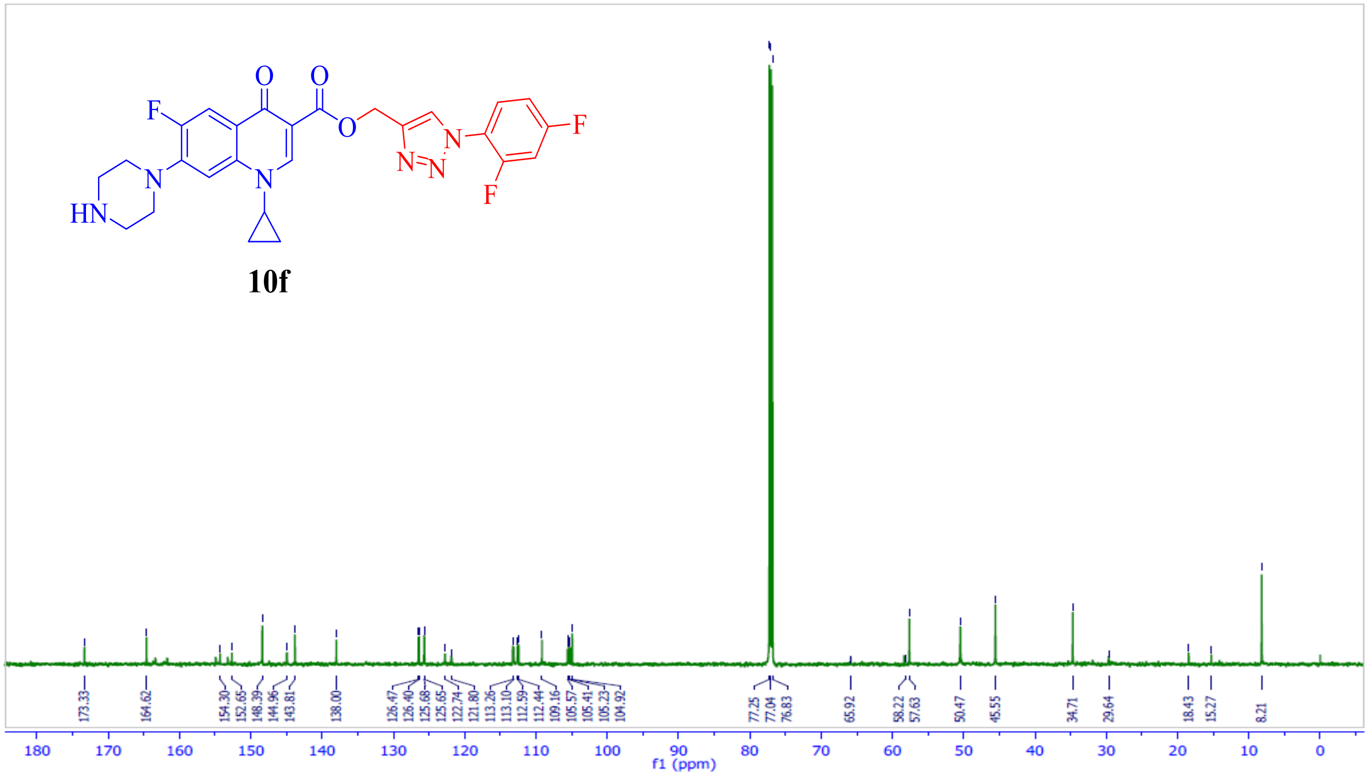

^{19}F-NMR spectrum of compound **10f**

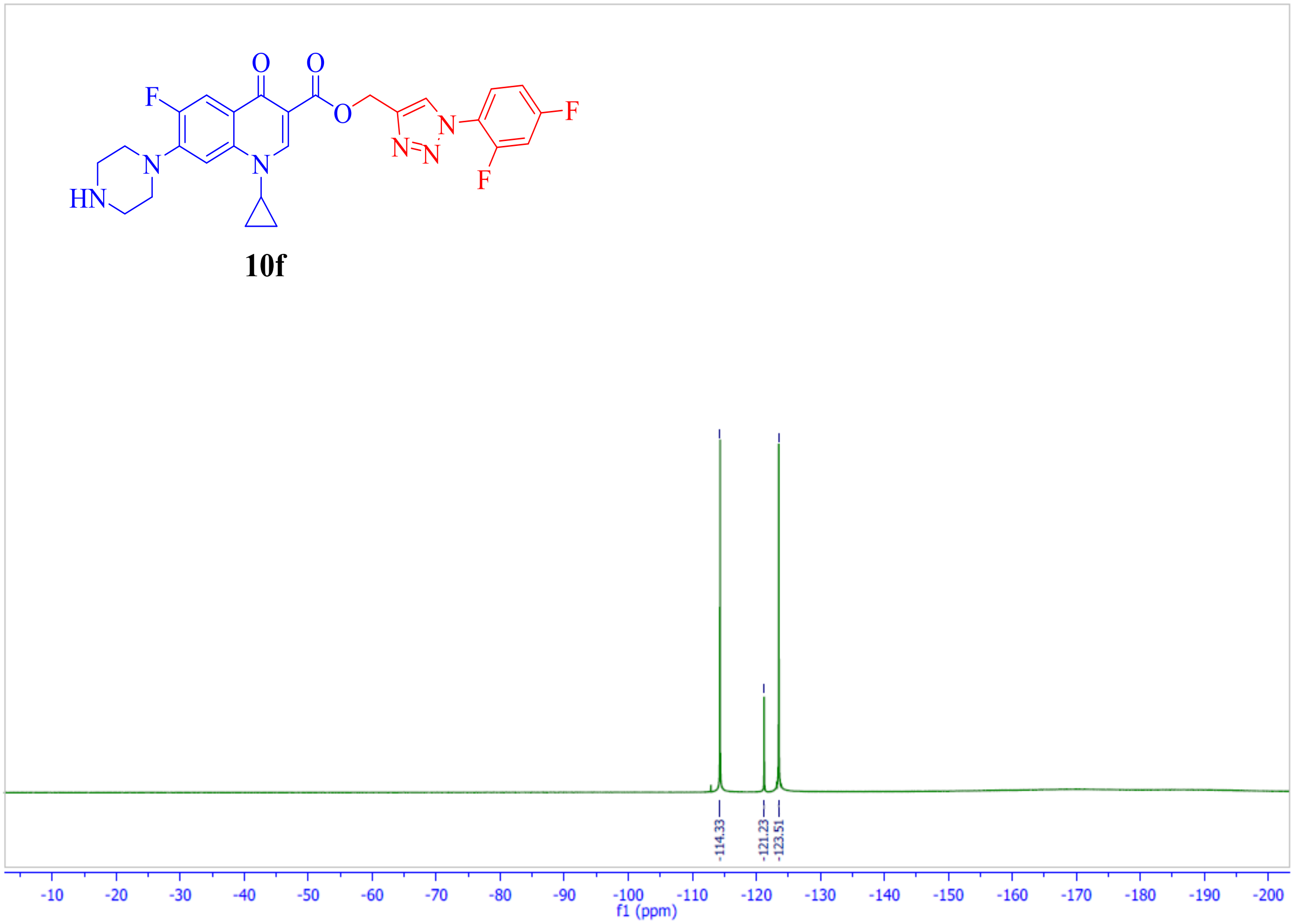

HRMS spectrum of compound**10f**

Data File CPR-16.d **Sample Name** CPR-16

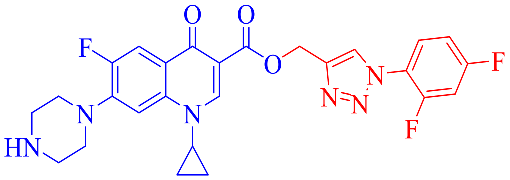

**Sample Type**	Sample	**Position**	P1-C2
**Instrument Name**	Instrument 1	**User Name**	
**Acq Method**	MS Scan.m	**Acquired Time**	24-05-2023 13:48:21
**IRM Calibration Status**	Success	**DA Method**	Default.m
**Comment**			

**Sample Group**		**Info.**	3
**Acquisition SW Version**	6200 series TOF/6500 series Q-TOF B.05.01 (B5125)		

Compound Table

Compound Label	RT	Mass	Abund	Formula	Tgt Mass	Diff (ppm)	MFG Formula	DB Formula
Cpd 1: C26 H23 F3N6 O3	0.121	524.1766	104279	C26 H23 F3 N6 O3	524.1784	-3.47	C26 H23 F3 N6 O3	C26 H23 F3 N6 O3

Compound Label	*m/z*	RT	Algorithm	Mass
Cpd 1: C26 H23 F3 N6 O3	525.1839	0.121	Find By Formula	524.1766

MS Zoomed Spectrum

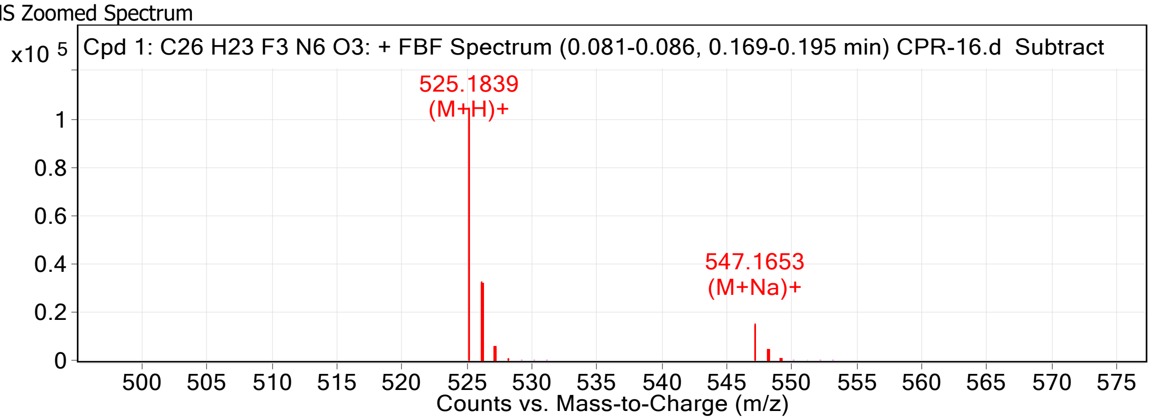

MS Spectrum Peak List

m/z	z	Abund	Formula	Ion
525.1839	1	104279.28	C26H24F3N6O3	(M+H)+
526.1869	1	32463.12	C26H24F3N6O3	(M+H)+
527.1889	1	5167.76	C26H24F3N6O3	(M+H)+
528.1914	1	635.47	C26H24F3N6O3	(M+H)+
547.1653	1	14701.31	C26H23F3N6NaO3	(M+Na)+
548.1682	1	4322.45	C26H23F3N6NaO3	(M+Na)+
549.171	1	650.53	C26H23F3N6NaO3	(M+Na)+

--- End Of Report ---

^{1}H-NMR spectrum of compound **11a**

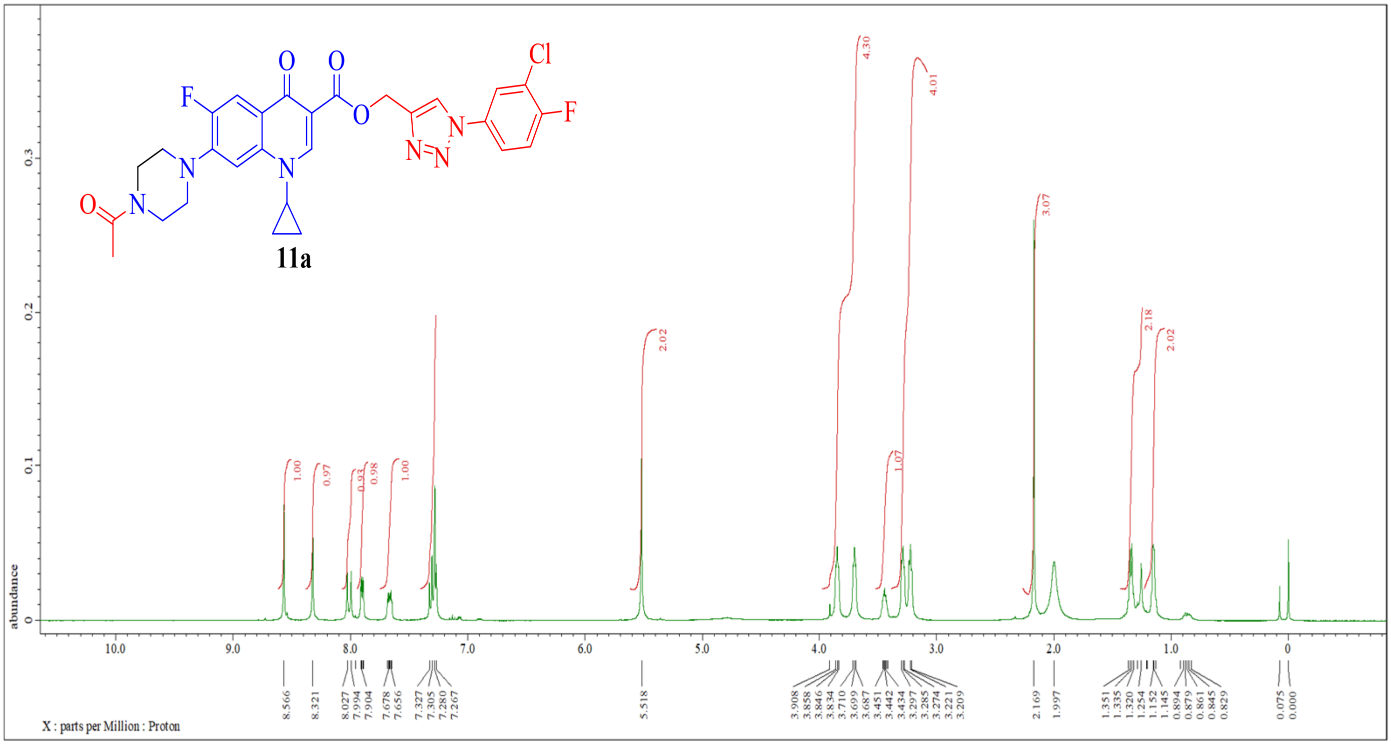

^{13}C-NMR spectrum of compound **11a**

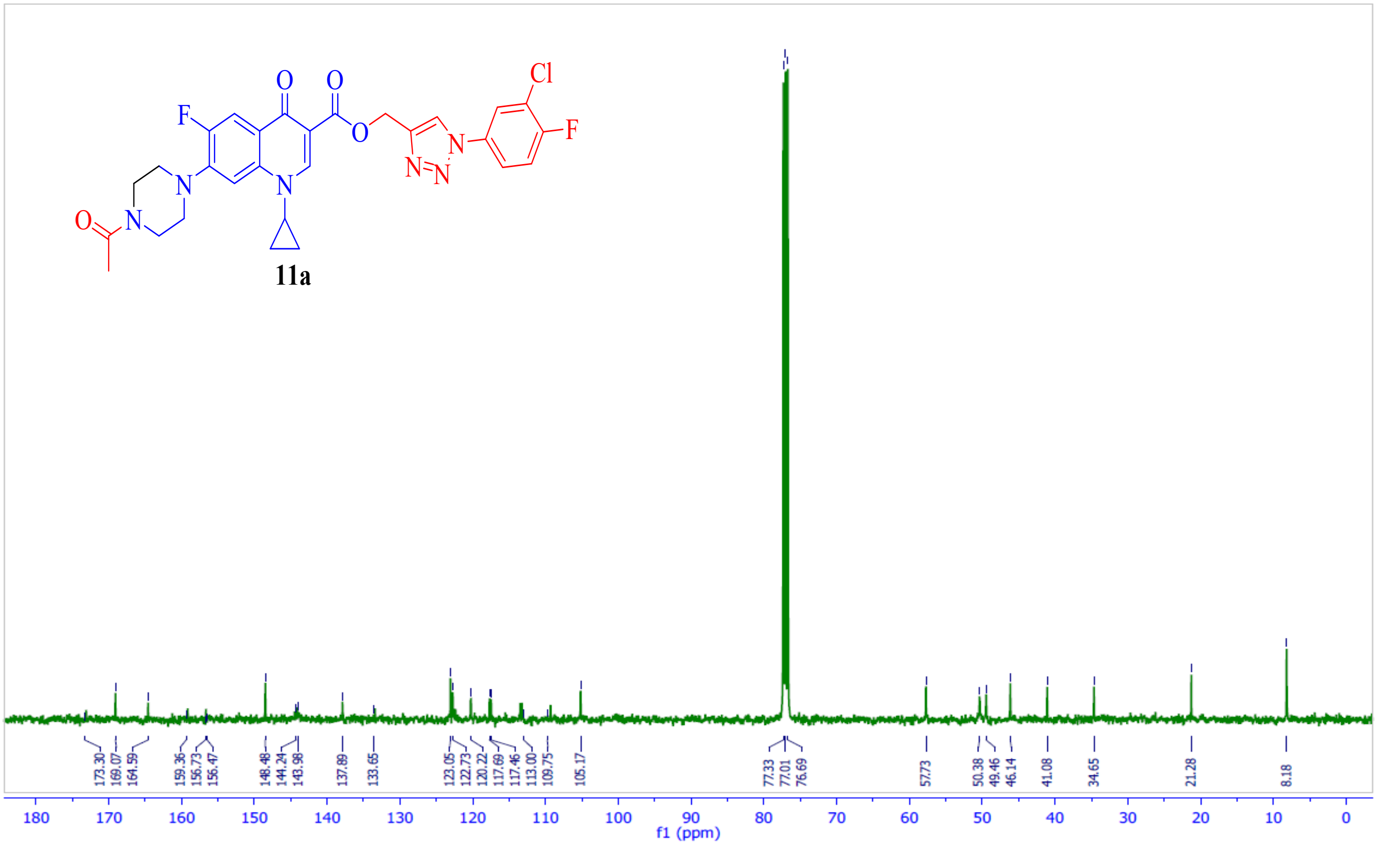

^{19}F-NMR spectrum of compound **11a**

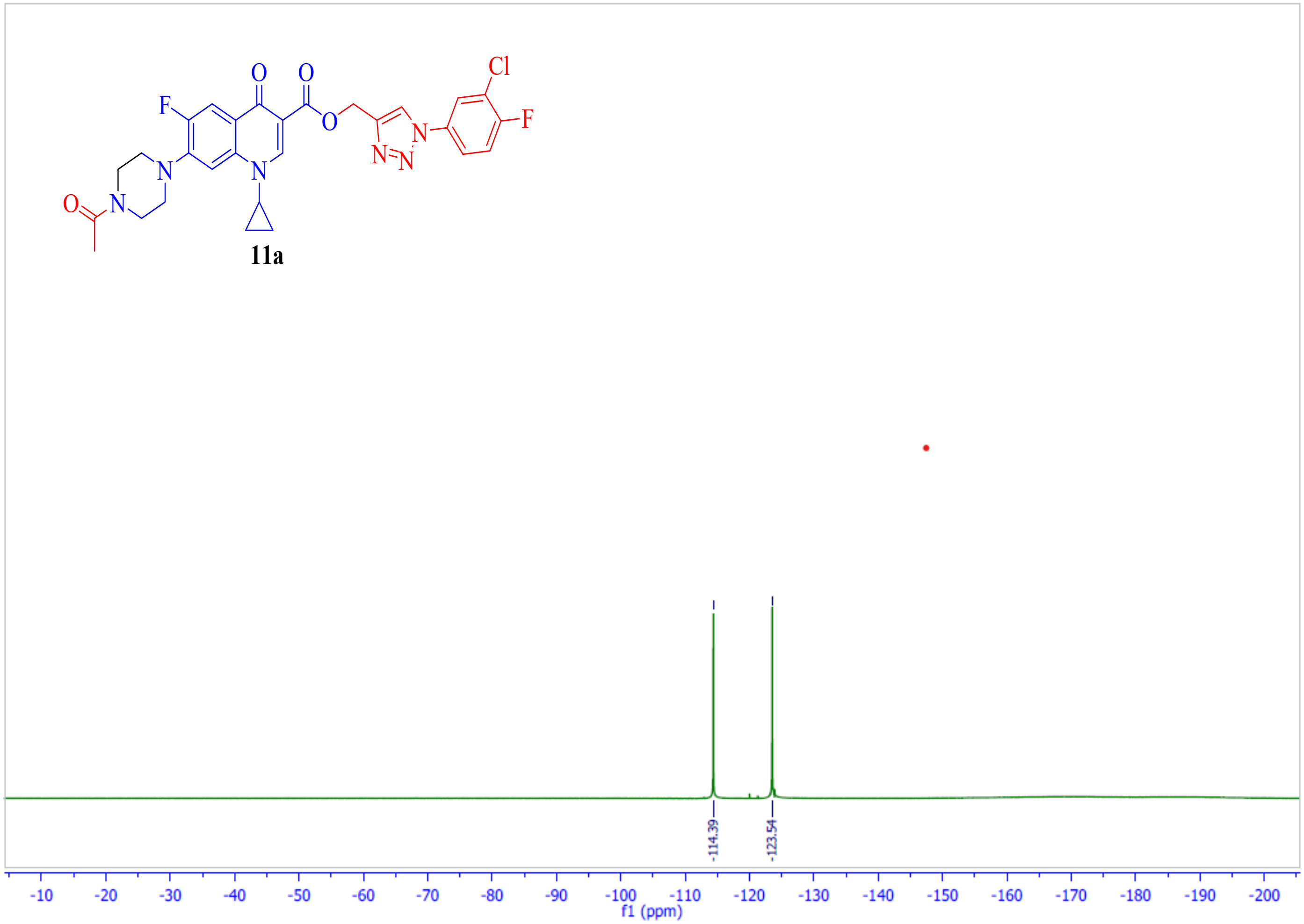

HRMS spectrum of compound **11a**

**Data File**	CPR-19.d	**Sample Name**	CPR-19
**Sample Type**	Sample	**Position**	P1-C4
**Instrument Name**	Instrument 1	**User Name**	
**Acq Method**	MS Scan.m	**Acquired Time**	24-05-2023 13:54:36
**IRM Calibration Status**	Success	**DA Method**	Default.m
**Comment**			
**Sample Group**		**Info.**	3
**Acquisition SW Version**	6200 series TOF/6500 series Q-TOF B.05.01 (B5125)		

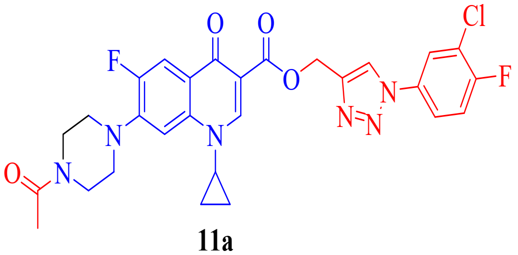

11a

Compound Table

Compound Label	RT	Mass	Abund	Formula	Tgt Mass	Diff (ppm)	MFG Formula	DB Formula
Cpd 1: C28 H25 Cl F2 N6 O4	0.103	582.1577	128259	C28 H25 Cl F2 N6 O4	582.1594	-2.83	C28 H25 Cl F2 N6 O4	C28 H25 Cl F2 N6 O4

Compound Label	*m/z*	RT	Algorithm	Mass
Cpd 1: C28 H25 Cl F2 N6 O4	583.1649	0.103	Find By Formula	582.1577

MS Zoomed Spectrum

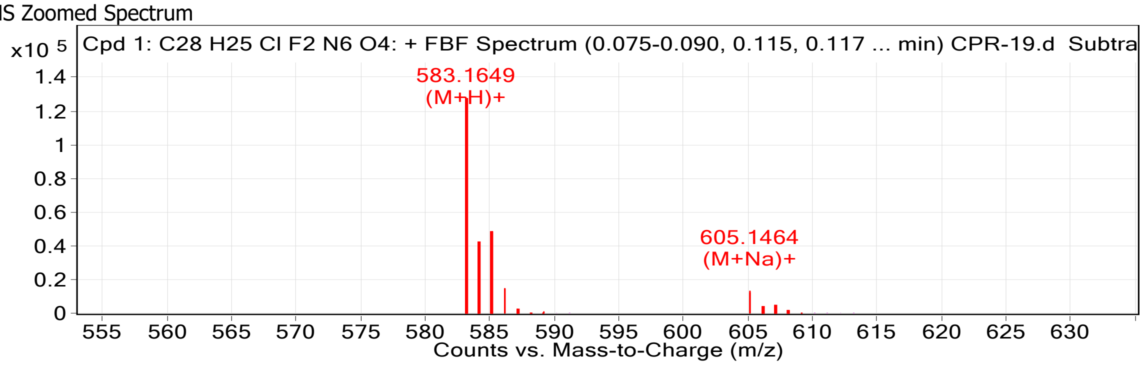

MS Spectrum Peak List

m/z	z	Abund	Formula	Ion
583.1649	1	128258.55	C28H26ClF2N6O4	(M+H)+
584.1684	1	41568.09	C28H26ClF2N6O4	(M+H)+
585.1638	1	47992.46	C28H26ClF2N6O4	(M+H)+
586.1653	1	14645.07	C28H26ClF2N6O4	(M+H)+
587.1673	1	2512.5	C28H26ClF2N6O4	(M+H)+
588.1706	1	294.41	C28H26ClF2N6O4	(M+H)+
589.1749	1	555.02	C28H26ClF2N6O4	(M+H)+
605.1464	1	12927.59	C28H25ClF2N6NaO4	(M+Na)+
606.1493	1	3954.72	C28H25ClF2N6NaO4	(M+Na)+
607.1449	1	4460.4	C28H25ClF2N6NaO4	(M+Na)+
608.1471	1	1258.04	C28H25ClF2N6NaO4	(M+Na)+
609.1494	1	189.78	C28H25ClF2N6NaO4	(M+Na)+

--- End Of Report ---

^{1}H-NMR spectrum of compound **11b**

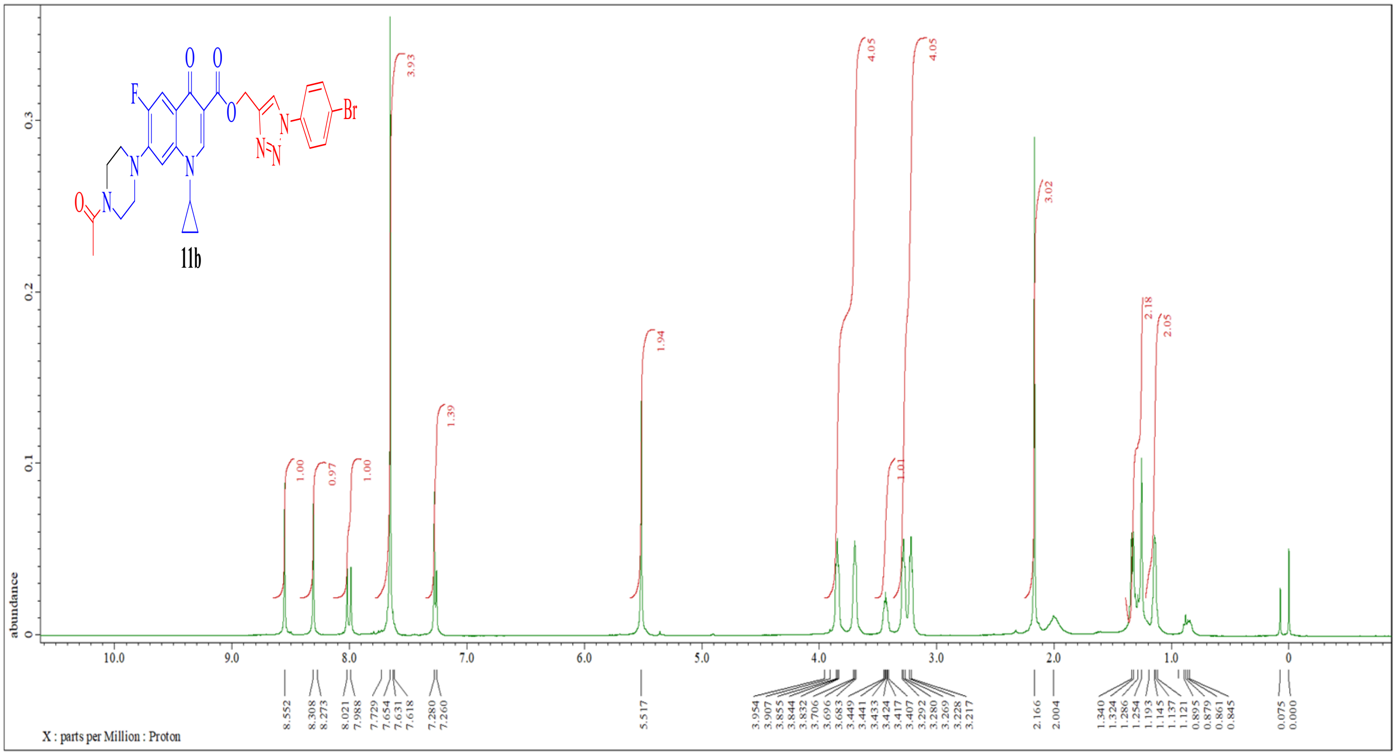

^{13}C-NMR spectrum of compound **11b**

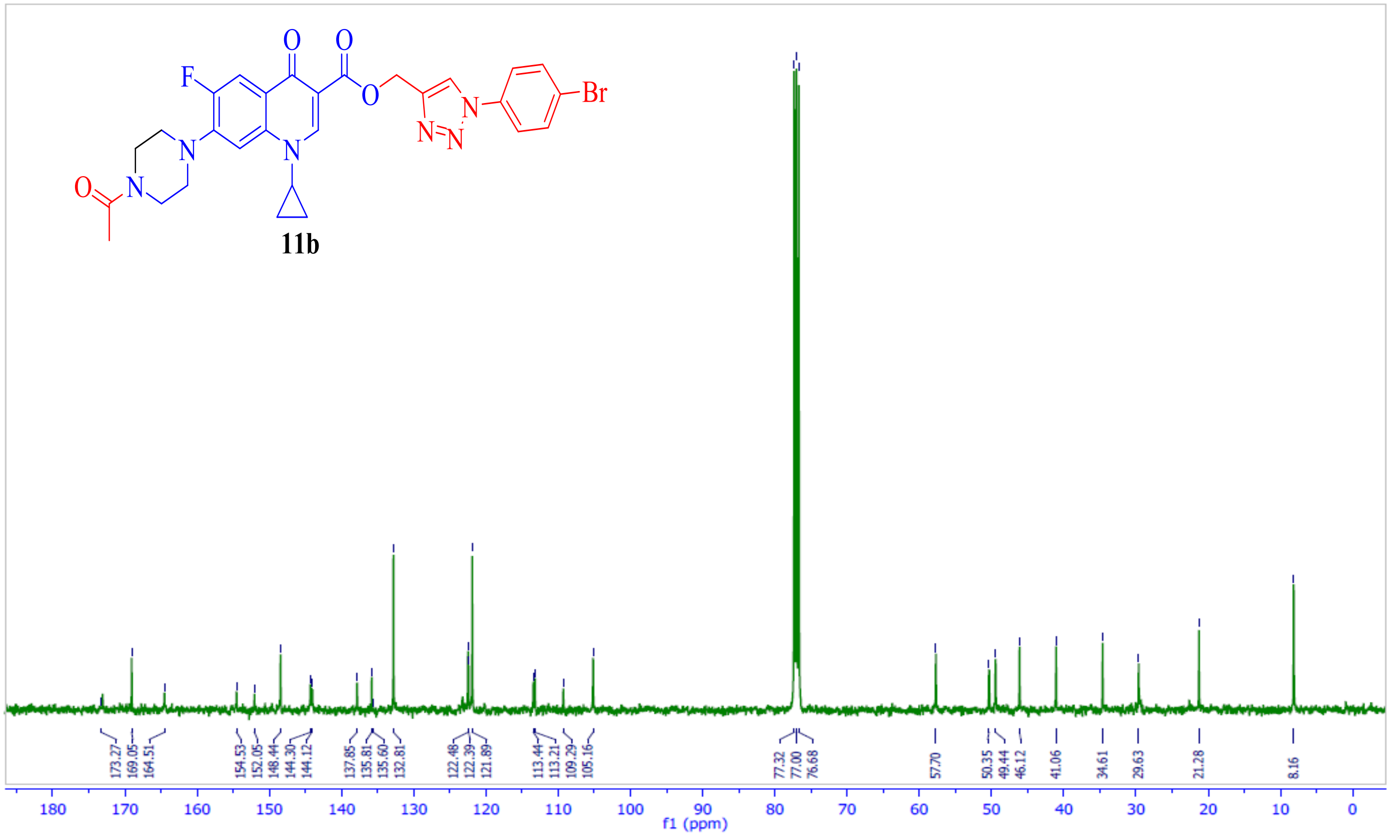

HRMS spectrum of compound **11b**

**Data File**	CPR-20.d	**Sample Name**	CPR-20
**Sample Type**	Sample	**Position**	P1-E2
**Instrument Name**	Instrument 1	**User Name**	
**Acq Method**	MS Scan.m	**Acquired Time**	01-06-2023 15:52:21
**IRM Calibration Status**	Success	**DA Method**	Default.m
**Comment**			
**Sample Group**		**Info.**	3
**Acquisition SW Version**	6200 series TOF/6500 series Q-TOF B.05.01 (B5125)		

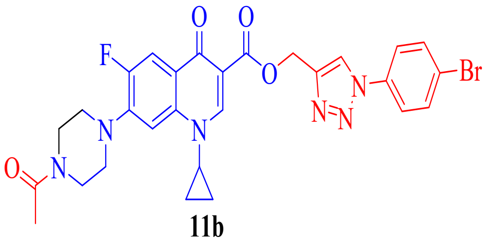

11b

Compound Table

Compound Label	RT	Mass	Abund	Formula	Tgt Mass	Diff (ppm)	MFG Formula	DB Formula
Cpd 1: C28 H26 Br F N6 O4	0.092	608.1202	5076	C28 H26 Br F N6 O4	608.1183	3.14	C28 H26 Br F N6 O4	C28 H26 Br F N6 O4

Compound Label	*m/z*	RT	Algorithm	Mass
Cpd 1: C28 H26 Br F N6 O4	633.1071	0.092	Find By Formula	608.1202

MS Zoomed Spectrum

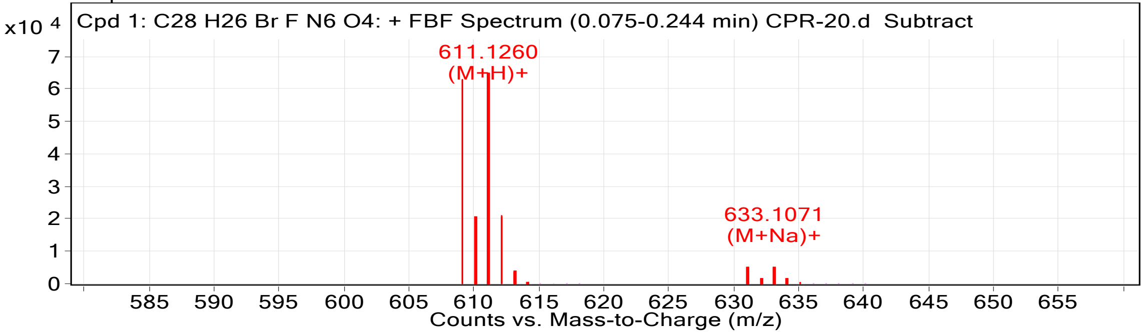

MS Spectrum Peak List

m/z	z	Abund	Formula	Ion
609.1274	1	63080.1	C28H27BrFN6O4	(M+H)+
610.1307	1	20531.09	C28H27BrFN6O4	(M+H)+
611.126	1	64951.37	C28H27BrFN6O4	(M+H)+
612.1287	1	20913.86	C28H27BrFN6O4	(M+H)+
613.131	1	3481.33	C28H27BrFN6O4	(M+H)+
614.1345	1	424.58	C28H27BrFN6O4	(M+H)+
631.1086	1	5025.58	C28H26BrFN6NaO4	(M+Na)+
632.1115	1	1522.32	C28H26BrFN6NaO4	(M+Na)+
633.1071	1	5076.27	C28H26BrFN6NaO4	(M+Na)+
634.1093	1	1514.47	C28H26BrFN6NaO4	(M+Na)+
635.1118	1	230.87	C28H26BrFN6NaO4	(M+Na)+

--- End Of Report ---

^{1}H-NMR spectrum of compound **11c**

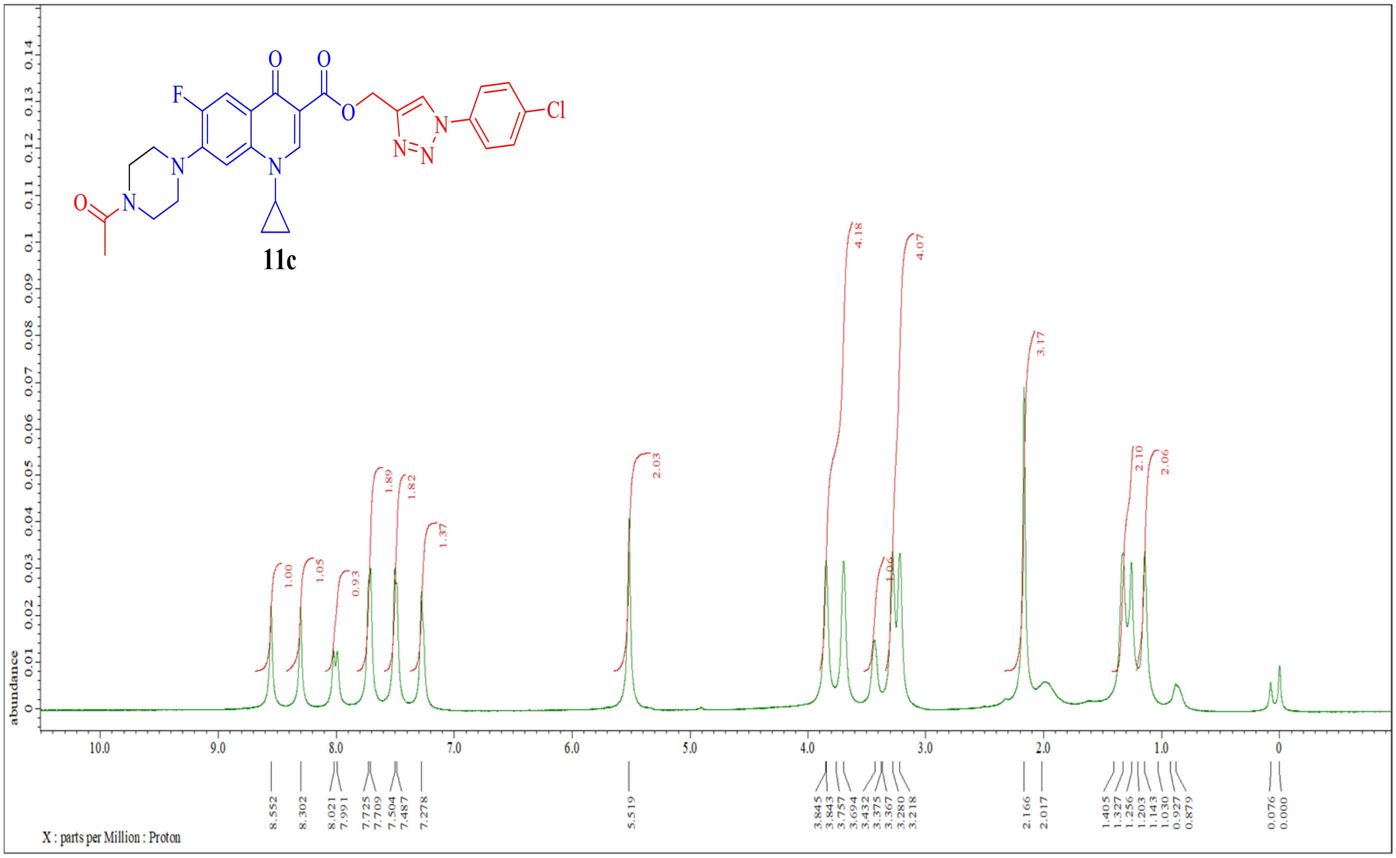

^{13}C-NMR spectrum of compound **11c**

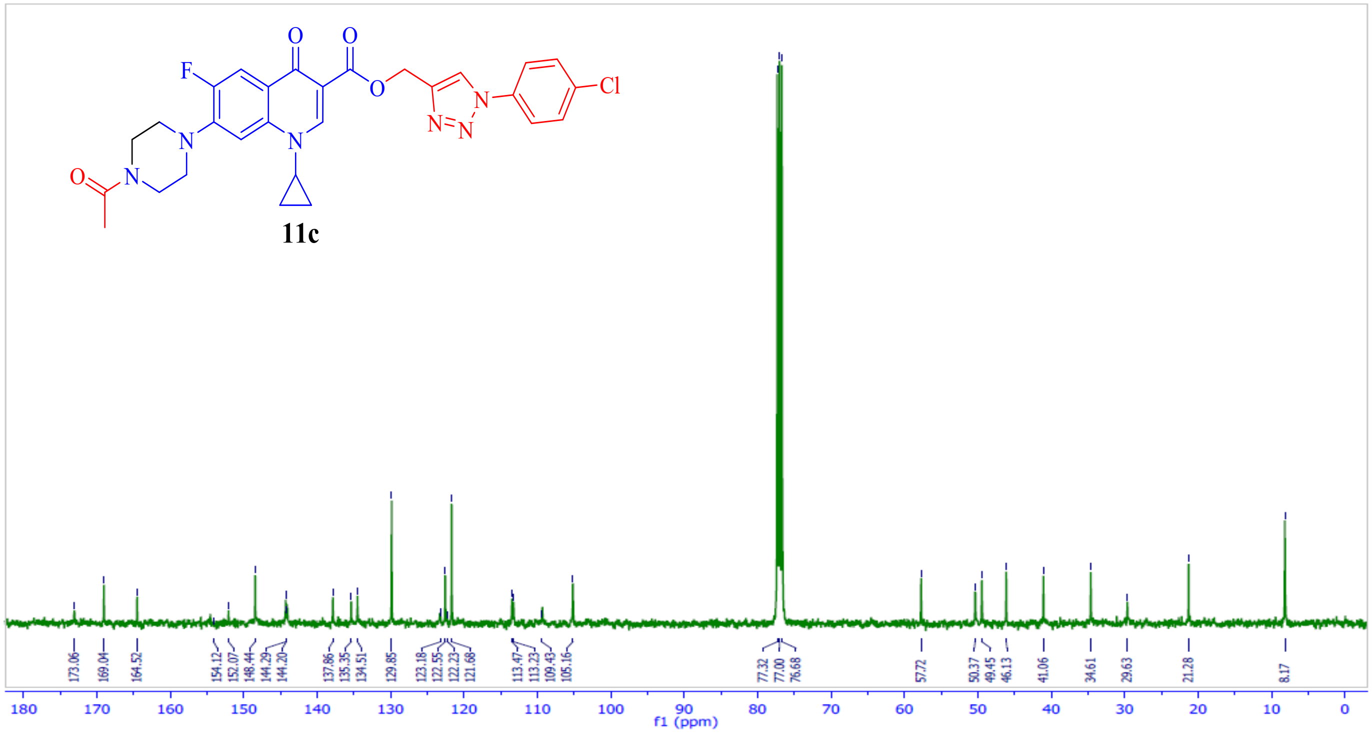

HRMS spectrum of compound **11c**

**Data File**	CPR-21.d	**Sample Name**	CPR-21
**Sample Type**	Sample	**Position**	P1-E3
**Instrument Name**	Instrument 1	**User Name**	
**Acq Method**	MS Scan.m	**Acquired Time**	01-06-2023 16:20:26
**IRM Calibration Status**	Success	**DA Method**	Default.m
**Comment**			
**Sample Group**		**Info.**	3
**Acquisition SW Version**	6200 series TOF/6500 series Q-TOF B.05.01 (B5125)		

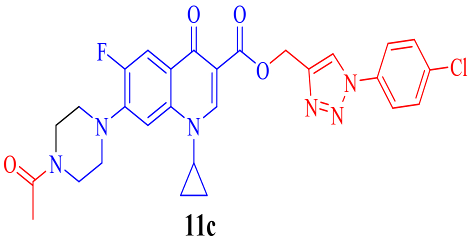

11c

Compound Table

Compound Label	RT	Mass	Abund	Formula	Tgt Mass	Diff (ppm)	MFG Formula	DB Formula
Cpd 1: C28 H26 Cl F N6 O4	0.094	564.1706	10896	C28 H26 Cl F N6 O4	564.1688	3.11	C28 H26 Cl F N6 O4	C28 H26 Cl F N6 O4

**Compound Label**	***m/z***	**RT**	**Algorithm**	**Mass**
Cpd 1: C28 H26 Cl F N6 O4	587.1593	0.094	Find By Formula	564.1706

MS Zoomed Spectrum

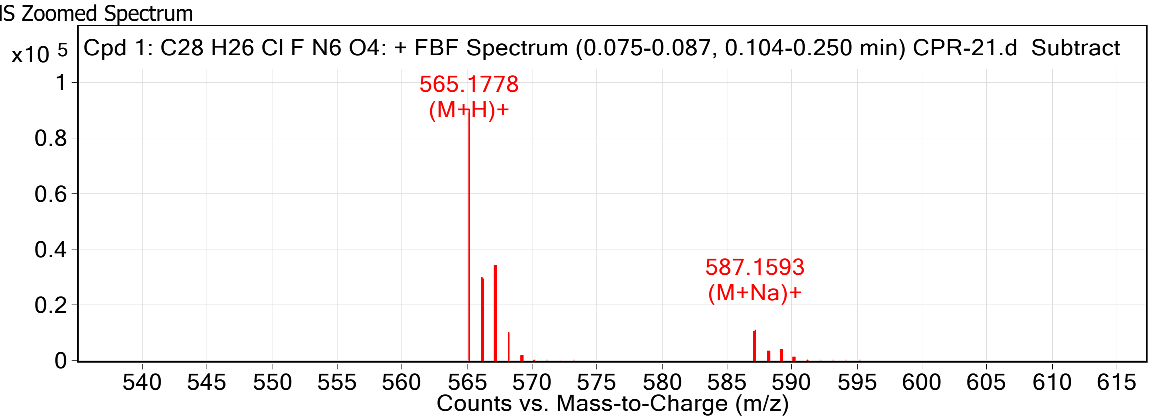

MS Spectrum Peak List

m/z	z	Abund	Formula	Ion
565.1778	1	90269.87	C28H27ClFN6O4	(M+H)+
566.181	1	29599.46	C28H27ClFN6O4	(M+H)+
567.1766	1	33482.64	C28H27ClFN6O4	(M+H)+
568.1784	1	9777.26	C28H27ClFN6O4	(M+H)+
569.1801	1	1606.94	C28H27ClFN6O4	(M+H)+
570.1822	1	185.51	C28H27ClFN6O4	(M+H)+
587.1593	1	10896.08	C28H26ClFN6NaO4	(M+Na)+
588.1619	1	3271.19	C28H26ClFN6NaO4	(M+Na)+
589.1575	1	3797.24	C28H26ClFN6NaO4	(M+Na)+
590.1595	1	1075.09	C28H26ClFN6NaO4	(M+Na)+

--- End Of Report ---

^{1}H-NMR spectrum of compound **11d**

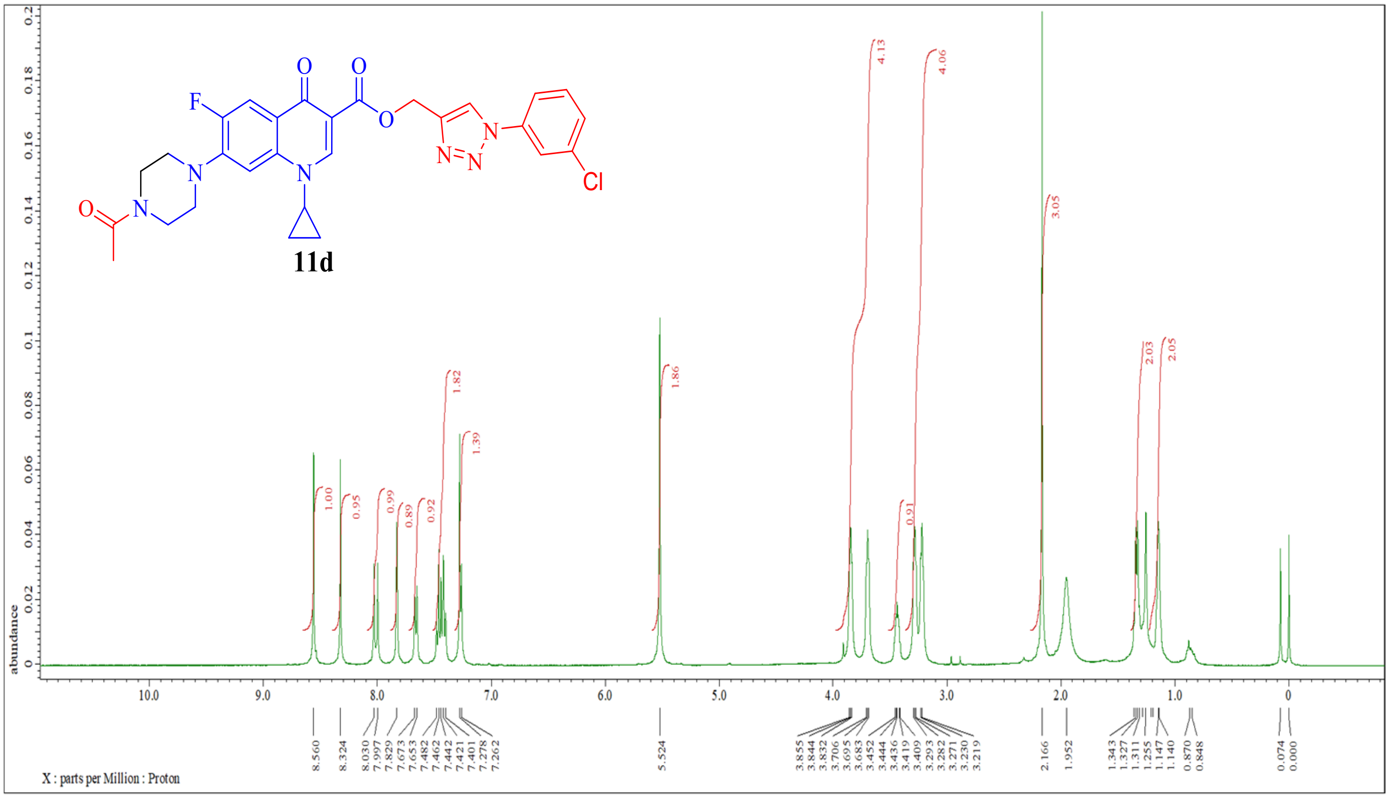

^{13}C-NMR spectrum of compound **11d**

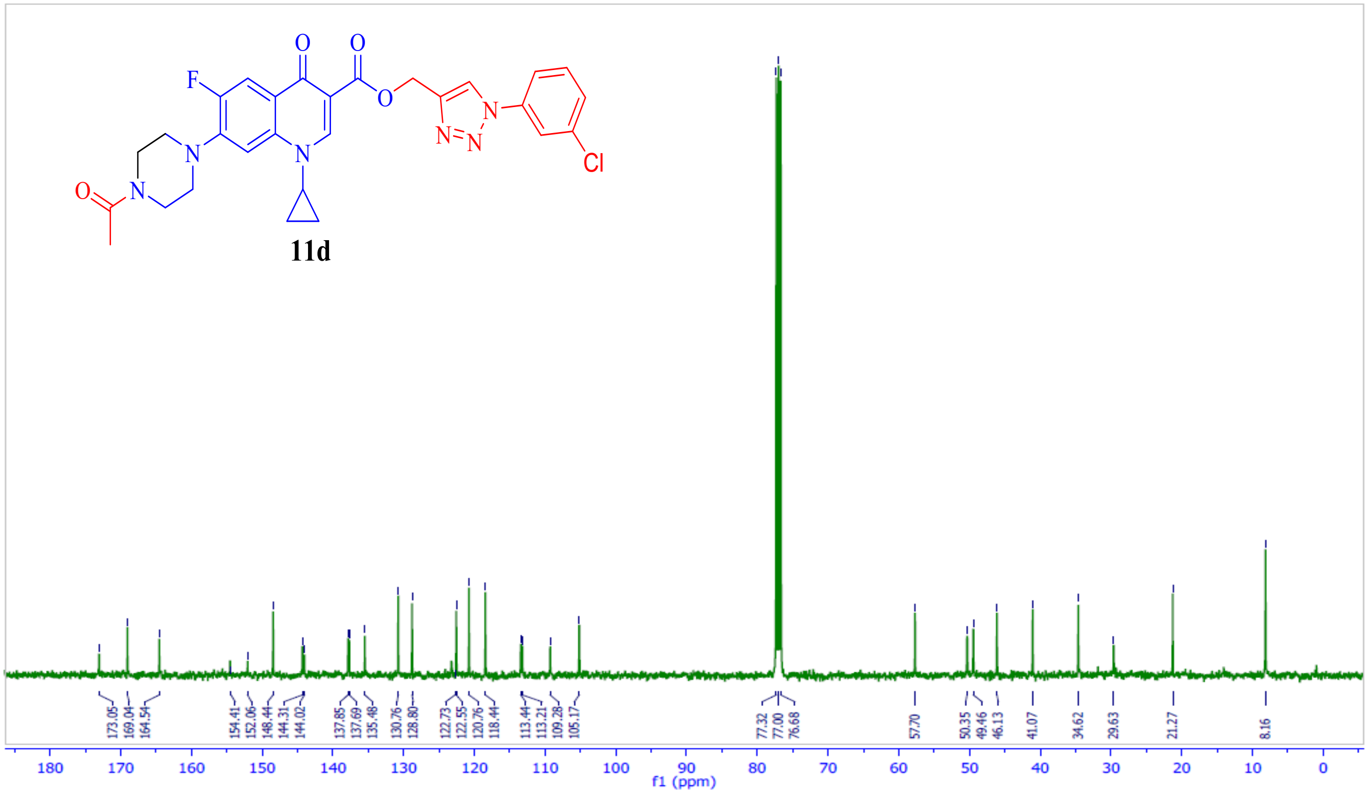

HRMS spectrum of compound **11d**

**Data File**	CPR-22.d	**Sample Name**	CPR-22
**Sample Type**	Sample	**Position**	P1-E4
**Instrument Name**	Instrument 1	**User Name**	
**Acq Method**	MS Scan.m	**Acquired Time**	01-06-2023 16:22:14
**IRM Calibration Status**	Success	**DA Method**	Default.m
**Comment**			

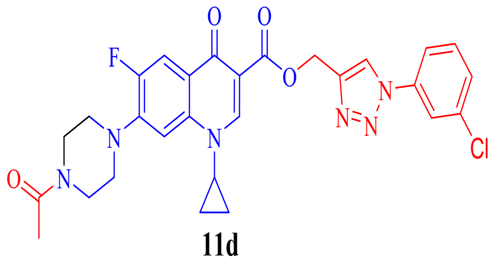

11d

**Sample Group**		**Info.**	3
**Acquisition SW Version**	6200 series TOF/6500 series Q-TOF B.05.01 (B5125)		

CompoundTable

Compound Label	RT	Mass	Abund	Formula	Tgt Mass	Diff (ppm)	MFG Formula	DB Formula
Cpd 1: C28 H26 Cl F N6 O4	0.095	564.1707	11509	C28 H26 Cl F N6 O4	564.1688	3.36	C28 H26 Cl F N6 O4	C28 H26 Cl F N6 O4

Compound Label	*m/z*	RT	Algorithm	Mass
Cpd 1: C28 H26 Cl F N6 O4	587.1596	0.095	Find By Formula	564.1707

MS Zoomed Spectrum

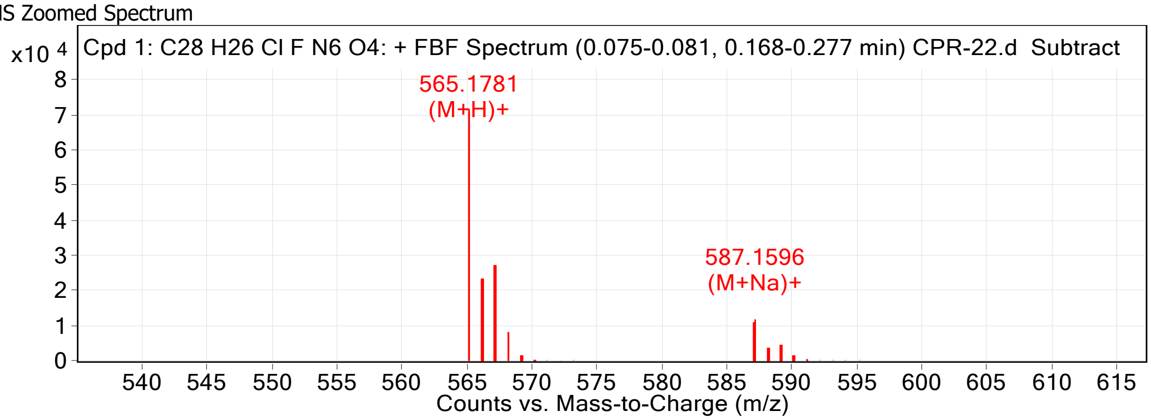

MS Spectrum Peak List

m/z	z	Abund	Formula	Ion
565.1781	1	71660.22	C28H27ClFN6O4	(M+H)+
566.181	1	23145.7	C28H27ClFN6O4	(M+H)+
567.1766	1	26514.95	C28H27ClFN6O4	(M+H)+
568.1783	1	7574.51	C28H27ClFN6O4	(M+H)+
569.1805	1	1257.58	C28H27ClFN6O4	(M+H)+
570.1837	1	118.32	C28H27ClFN6O4	(M+H)+
587.1596	1	11508.69	C28H26ClFN6NaO4	(M+Na)+
588.1622	1	3405.61	C28H26ClFN6NaO4	(M+Na)+
589.1577	1	3969.61	C28H26ClFN6NaO4	(M+Na)+
590.1599	1	1101.06	C28H26ClFN6NaO4	(M+Na)+
591.1616	1	131.83	C28H26ClFN6NaO4	(M+Na)+

--- End Of Report ---

^{1}H-NMR spectrum of compound **11e**

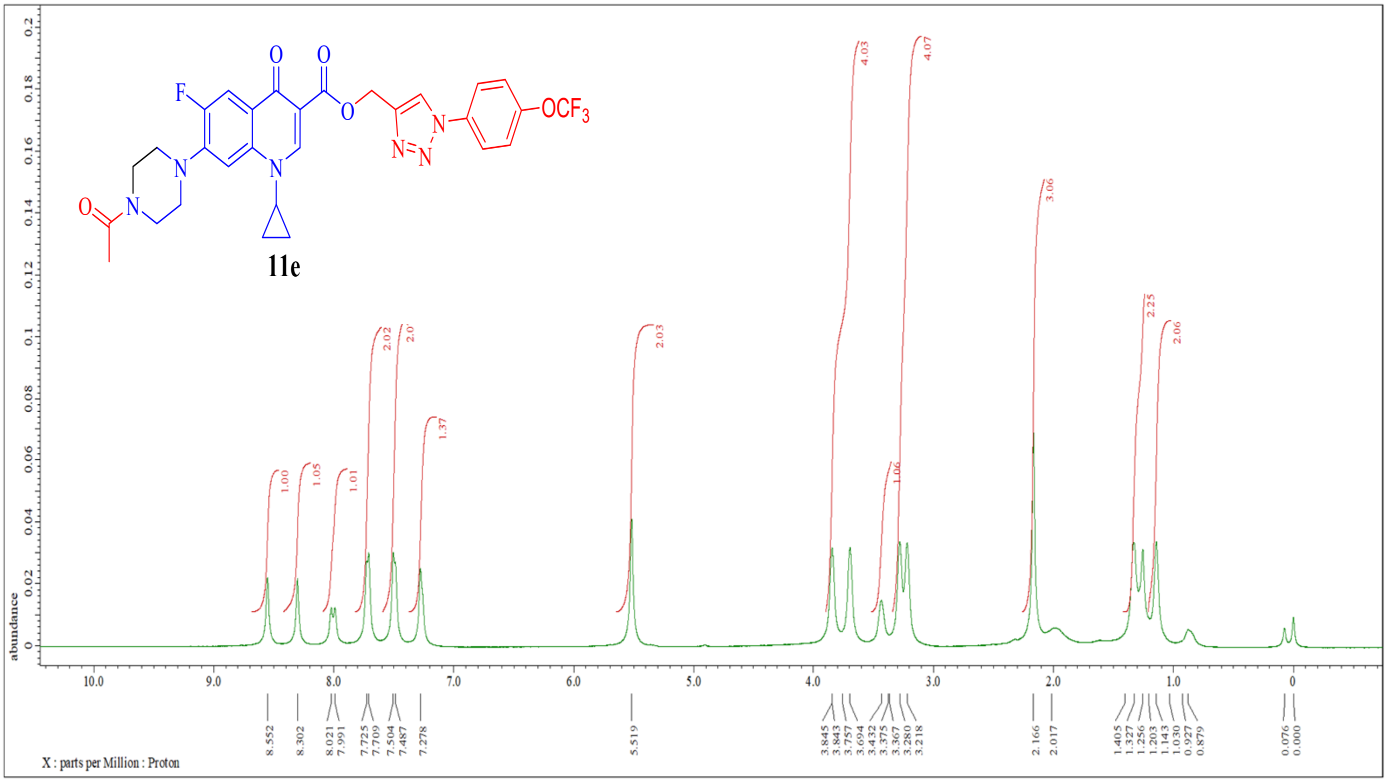

^{13}C-NMR spectrum of compound **11e**

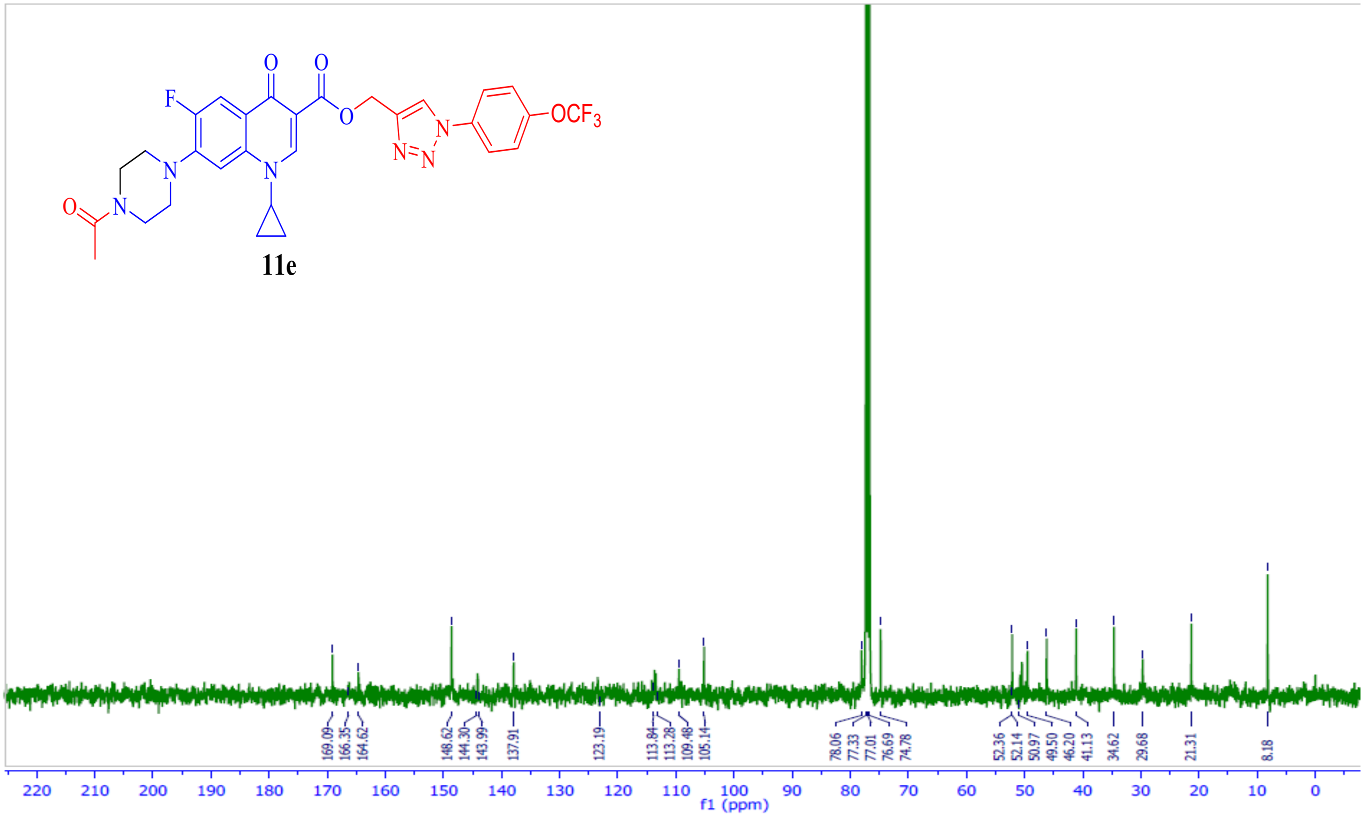

^{19}F-NMR spectrum of compound **11e**

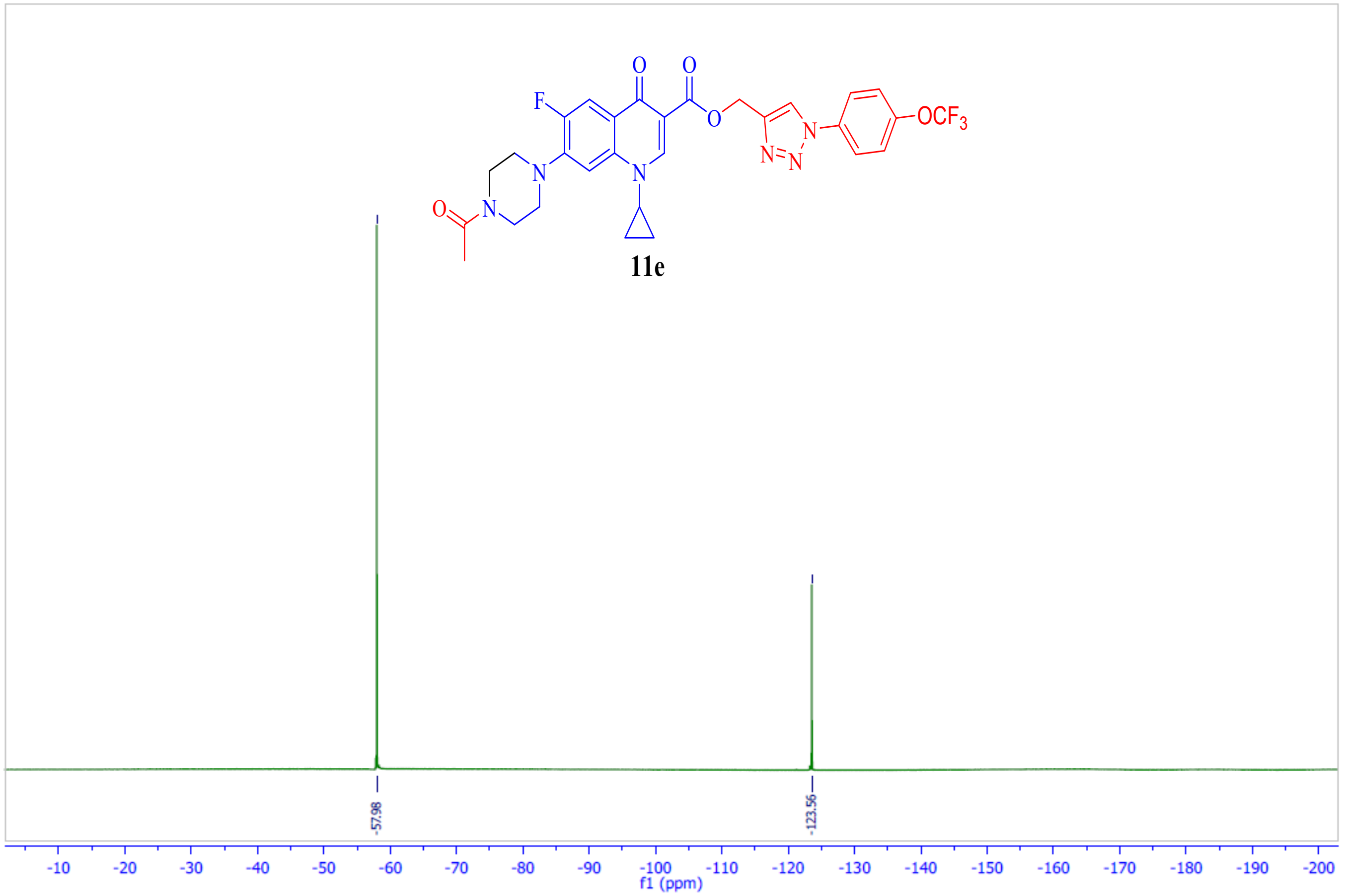

HRMS spectrum of compound **11e**

**Data File**	CPR-23.d	**Sample Name**	CPR-23
**Sample Type**	Sample	**Position**	P1-E5
**Instrument Name**	Instrument 1	**User Name**	
**Acq Method**	MS Scan.m	**Acquired Time**	01-06-2023 16:34:43
**IRM Calibration Status**	Success	**DA Method**	Default.m
**Comment**			

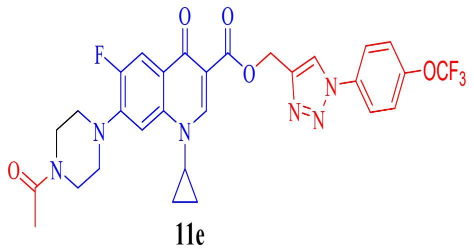

11e

**Sample Group**		**Info.**	3
**Acquisition SW Version**	6200 series TOF/6500 series Q-TOF B.05.01 (B5125)		

Compound Table

Compound Label	RT	Mass	Abund	Formula	Tgt Mass	Diff (ppm)	MFG Formula	DB Formula
Cpd 1: C29 H26 F4 N6 O5	0.096	614.1921	6709	C29 H26 F4 N6 O5	614.1901	3.35	C29 H26 F4 N6 O5	C29 H26 F4 N6 O5

Compound Label	*m/z*	RT	Algorithm	Mass
Cpd 1: C29 H26 F4 N6 O5	637.1807	0.096	Find By Formula	614.1921

MS Zoomed Spectrum

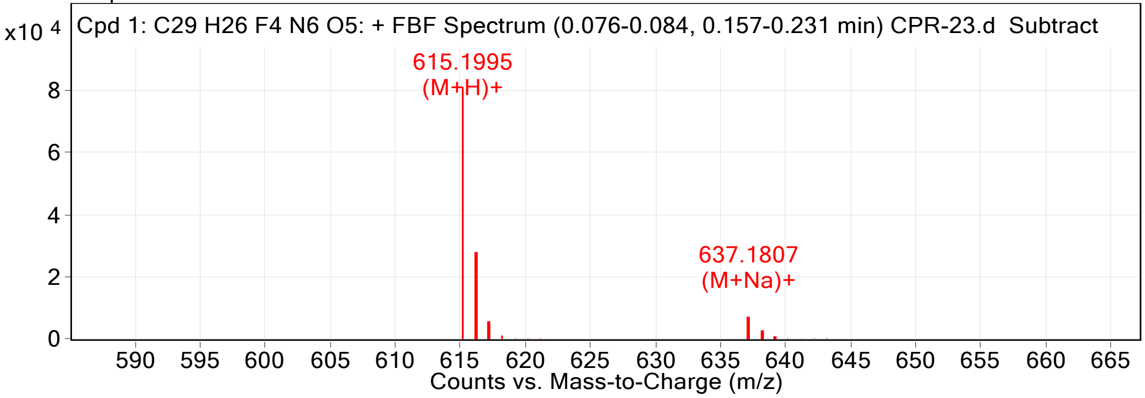

MS Spectrum Peak List

m/z	z	Abund	Formula	Ion
615.1995	1	81111.67	C29H27F4N6O5	(M+H)+
616.2025	1	27492.97	C29H27F4N6O5	(M+H)+
617.2047	1	5063.75	C29H27F4N6O5	(M+H)+
618.2073	1	652.4	C29H27F4N6O5	(M+H)+
637.1807	1	6708.84	C29H26F4N6NaO5	(M+Na)+
638.1834	1	2130.26	C29H26F4N6NaO5	(M+Na)+
639.1859	1	376.75	C29H26F4N6NaO5	(M+Na)+

--- End Of Report ---

^{1}H-NMR spectrum of compound **11f**

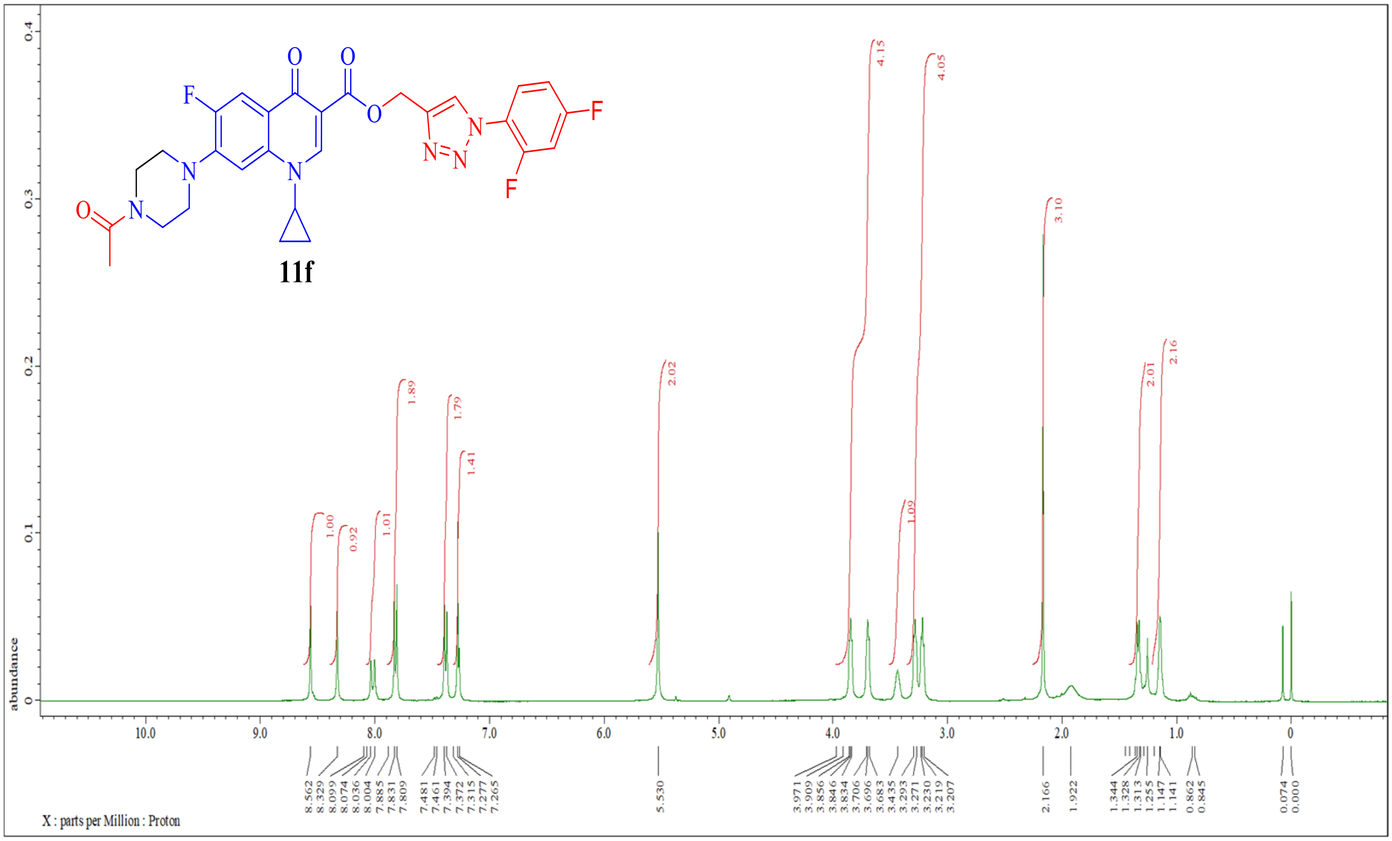

^{13}C-NMR spectrum of compound **11f**

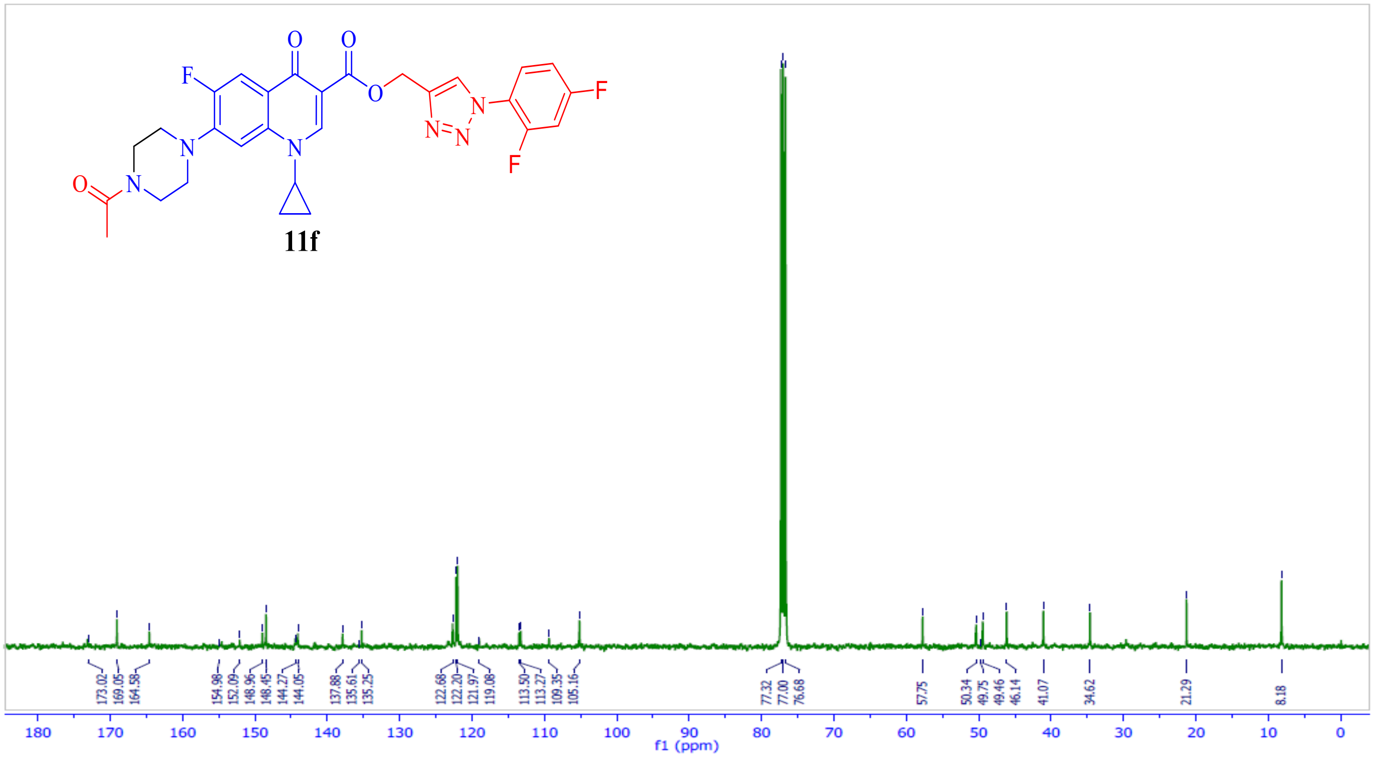

^{19}F-NMR spectrum of compound **11f**

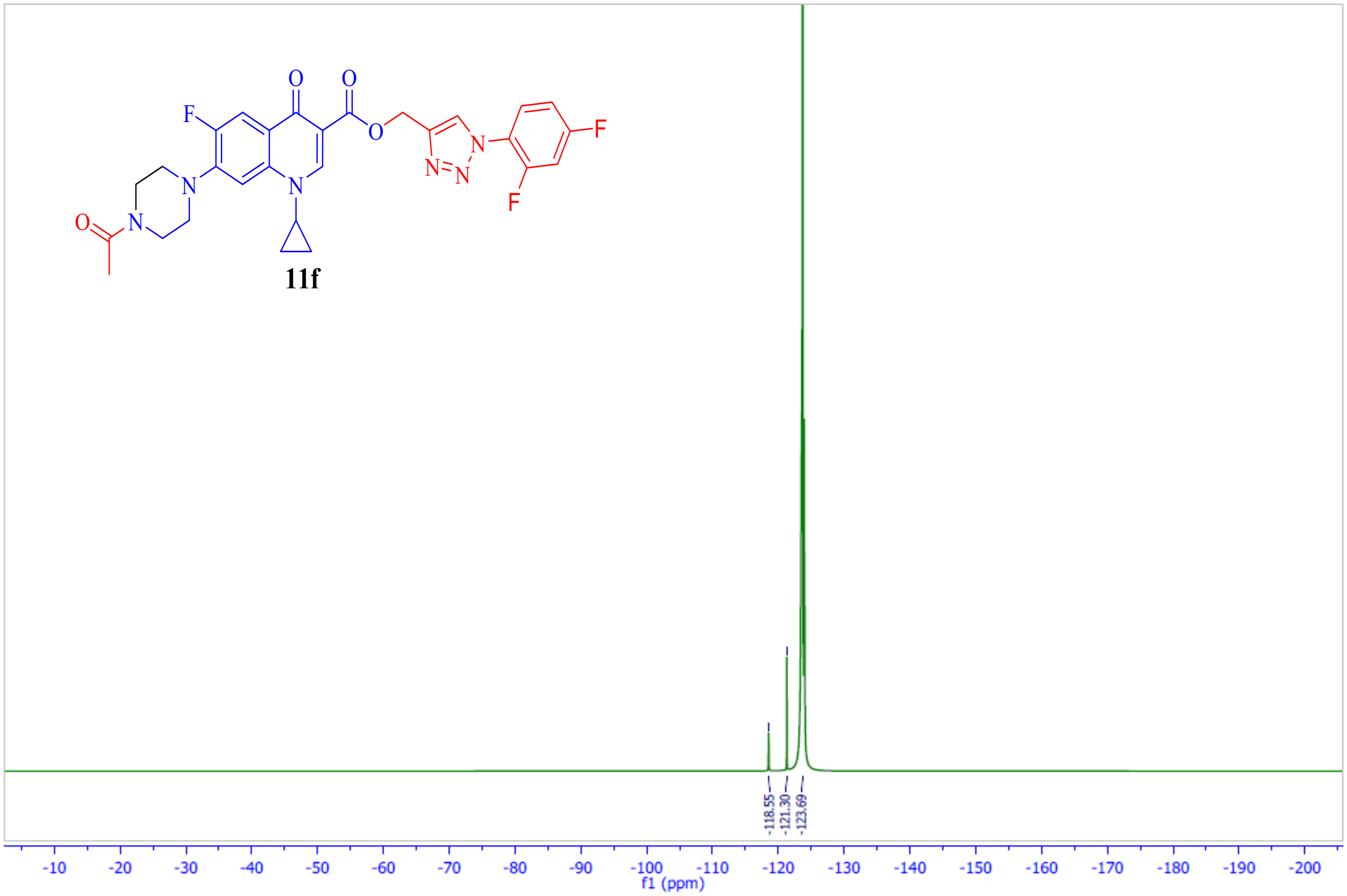

HRMS spectrum of compound **11f**

**Data File**	CPR-24.d	**Sample Name**	CPR-24
**Sample Type**	Sample	**Position**	P1-E6
**Instrument Name**	Instrument 1	**User Name**	
**Acq Method**	MS Scan.m	**Acquired Time**	01-06-2023 16:37:37
**IRM Calibration Status**	Success	**DA Method**	Default.m
**Comment**			

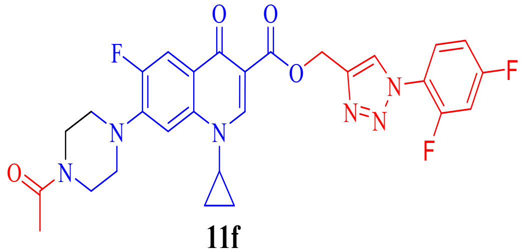

11f

**Sample Group**		**Info.**	3
**Acquisition SW Version**	6200 series TOF/6500 series Q-TOF B.05.01 (B5125)		

Compound Table

Compound Label	RT	Mass	Abund	Formula	Tgt Mass	Diff (ppm)	MFG Formula	DB Formula
Cpd 1: C28 H25 F3 N6 O4	0.099	566.1902	11498	C28 H25 F3 N6 O4	566.1889	2.25	C28 H25 F3 N6 O4	C28 H25 F3 N6 O4

Compound Label	*m/z*	RT	Algorithm	Mass
Cpd 1: C28 H25 F3 N6 O4	567.1976	0.099	Find By Formula	566.1902

MS Zoomed Spectrum

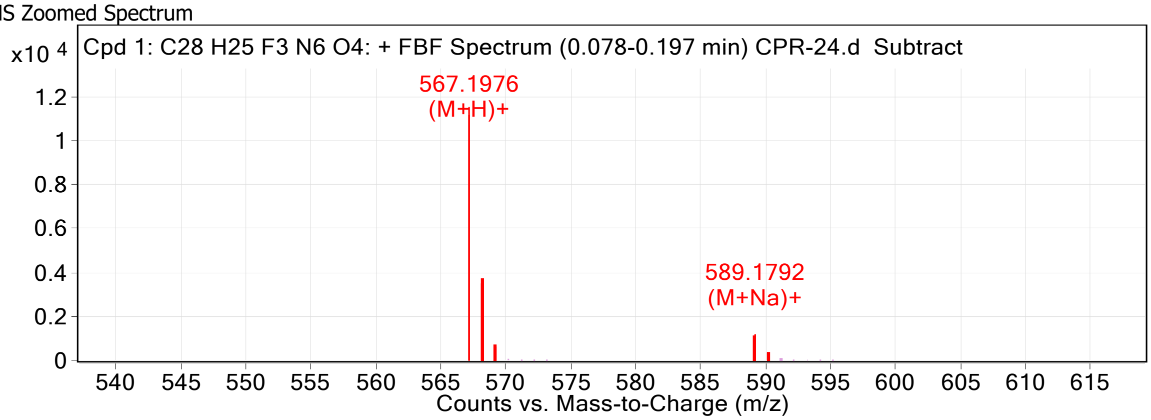

MS Spectrum Peak List

m/z	z	Abund	Formula	Ion
567.1976	1	11498.28	C28H26F3N6O4	(M+H)+
568.2003	1	3477.83	C28H26F3N6O4	(M+H)+
569.2023	1	589.49	C28H26F3N6O4	(M+H)+
589.1792	1	1143.53	C28H25F3N6NaO4	(M+Na)+
590.1821	1	340.76	C28H25F3N6NaO4	(M+Na)+

--- End Of Report ---

^{1}H-NMR spectrum of compound **12a**

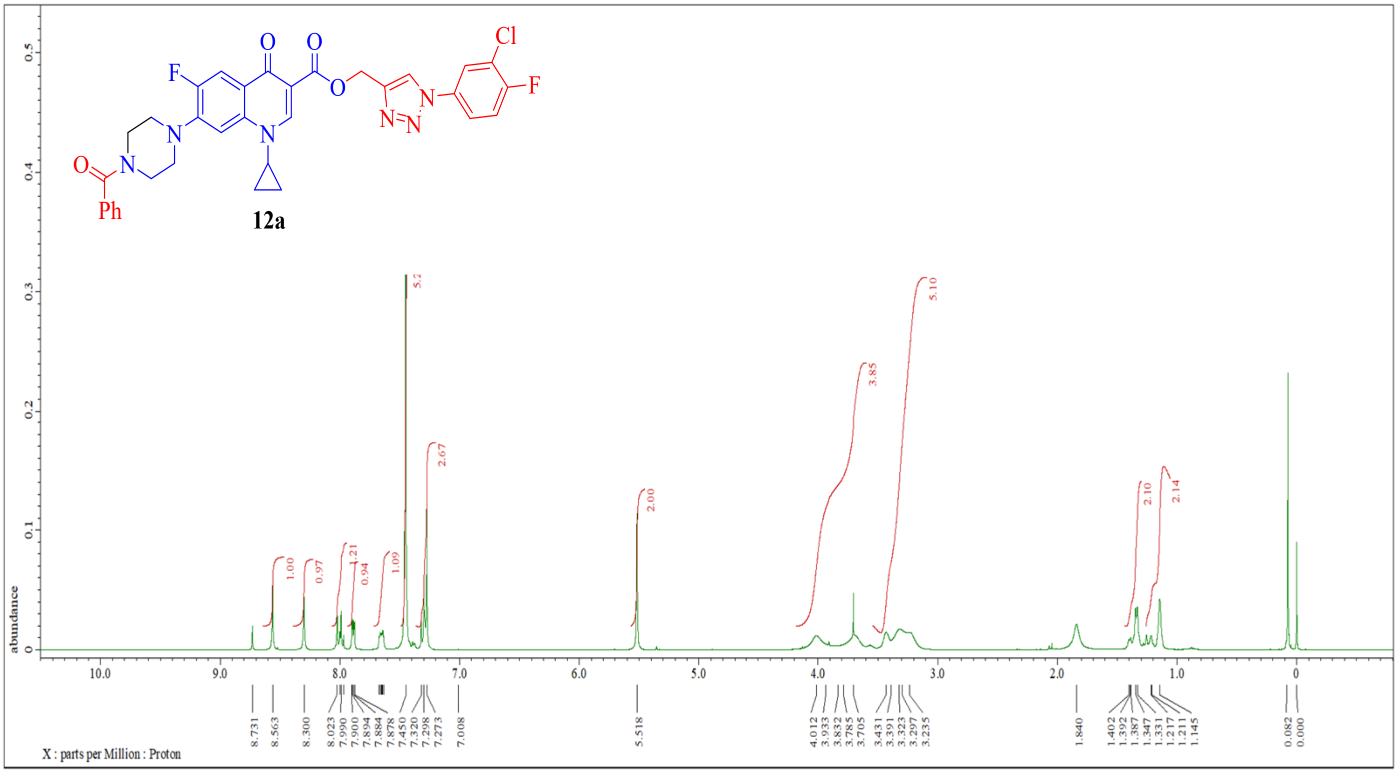

^{13}C-NMR spectrum of compound **12a**

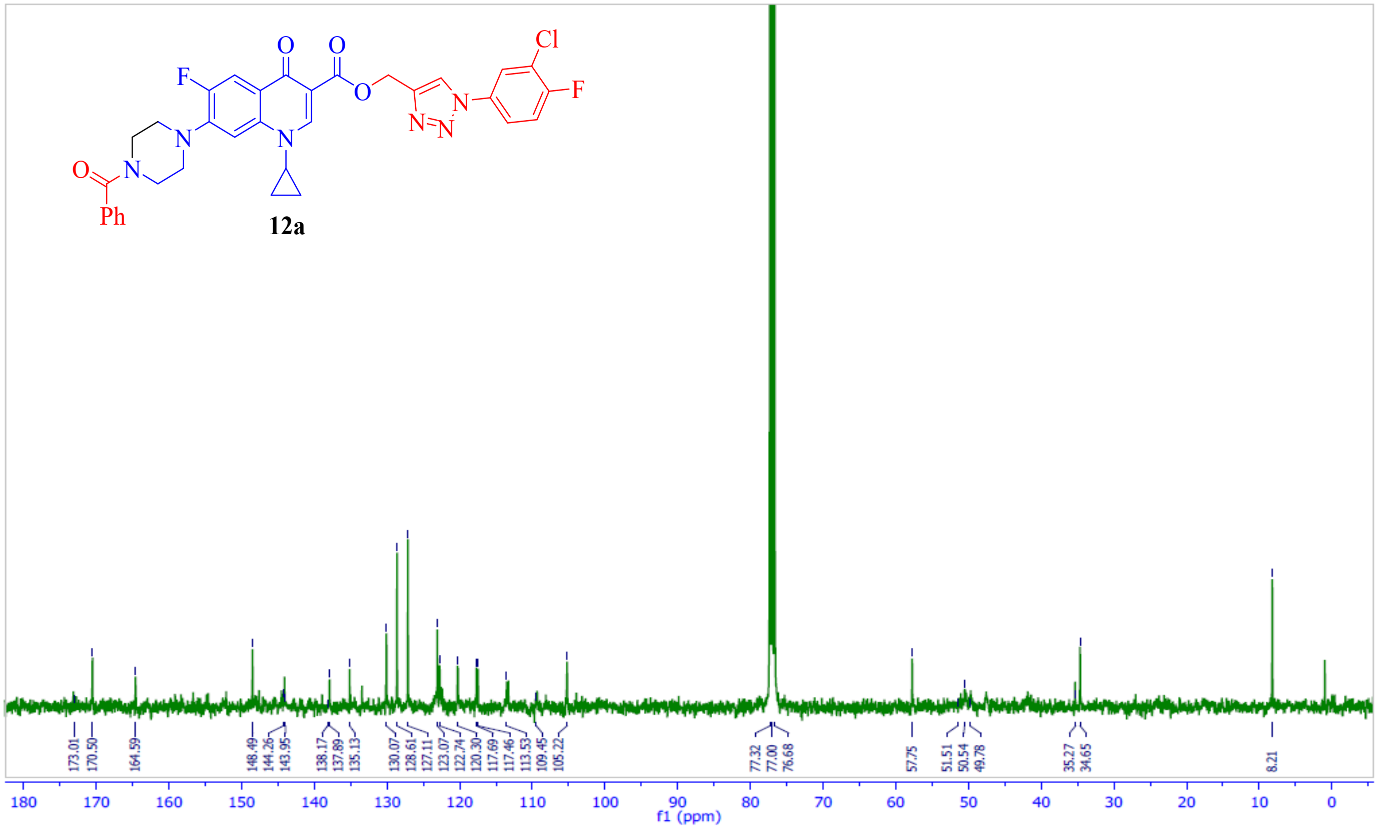

^{19}F-NMR spectrum of compound **12a**

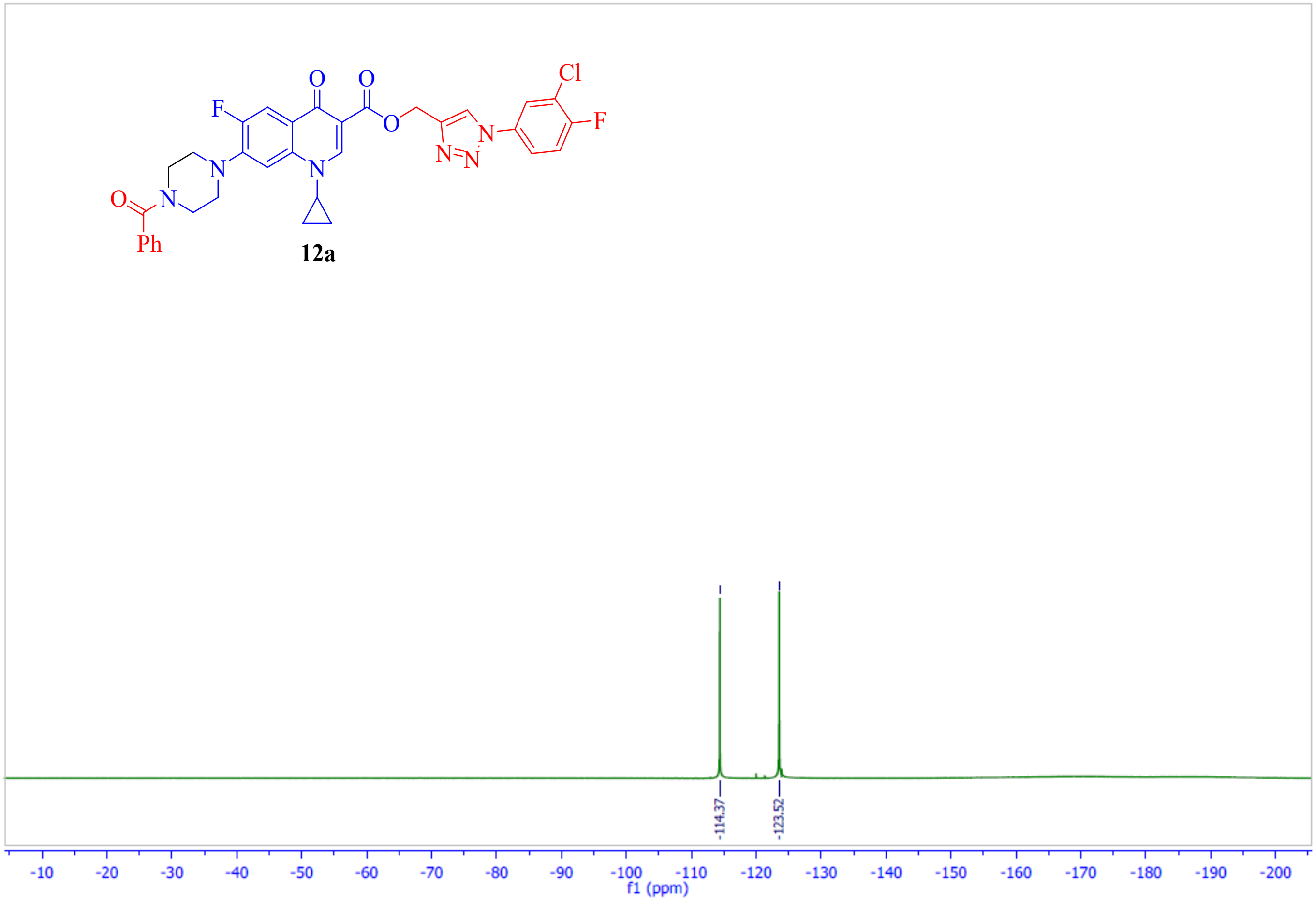

HRMS spectrum of compound **12a**

**Data File**	CPR-27.d	**Sample Name**	CPR-27
**Sample Type**	Sample	**Position**	P1-C2
**Instrument Name**	Instrument 1	**User Name**	
**Acq Method**	MS Scan_02.m	**Acquired Time**	07-06-2023 15:40:00
**IRM Calibration Status**	Success	**DA Method**	Default.m
**Comment**			

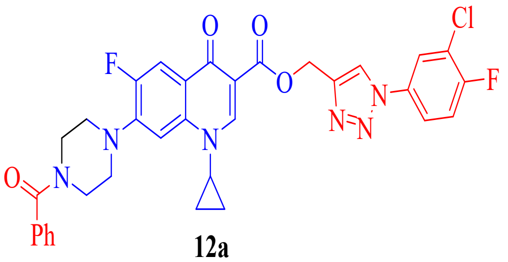

12a

**Sample Group**		**Info.**	3
**Acquisition SW Version**	6200 series TOF/6500 series Q-TOF B.05.01 (B5125)		

Compound Table

Compound Label	RT	Mass	Abund	Formula	Tgt Mass	Diff (ppm)	MFG Formula	DB Formula
Cpd 1: C33 H27 Cl F2 N6 O4	0.101	644.1756	37038	C33 H27 Cl F2 N6 O4	644.175	0.93	C33 H27 Cl F2 N6 O4	C33 H27 Cl F2 N6 O4

Compound Label	*m/z*	RT	Algorithm	Mass
Cpd 1: C33 H27 Cl F2 N6 O4	645.183	0.101	Find By Formula	644.1756

MS Zoomed Spectrum

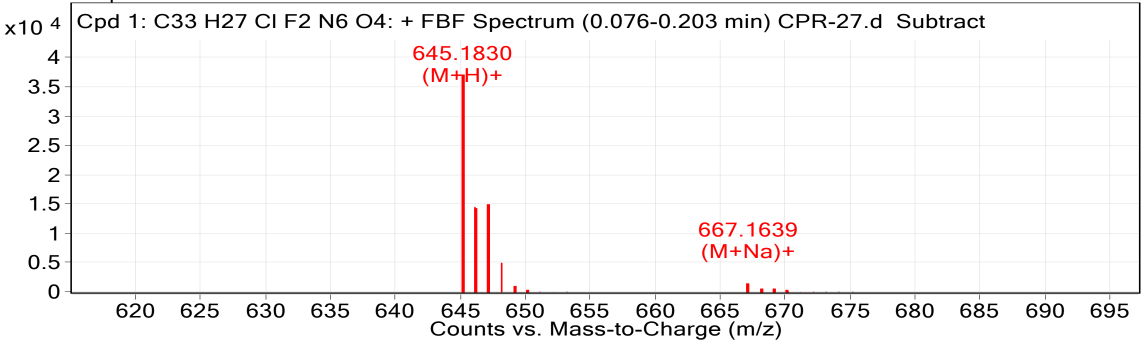

MS Spectrum Peak List

m/z	z	Abund	Formula	Ion
645.183	1	37037.52	C33H28ClF2N6O4	(M+H)+
646.186	1	14411.12	C33H28ClF2N6O4	(M+H)+
647.1819	1	14854.86	C33H28ClF2N6O4	(M+H)+
648.1833	1	4773.1	C33H28ClF2N6O4	(M+H)+
649.1853	1	888.66	C33H28ClF2N6O4	(M+H)+
650.1878	1	54.62	C33H28ClF2N6O4	(M+H)+
667.1639	1	1342.82	C33H27ClF2N6NaO4	(M+Na)+
668.1665	1	485.27	C33H27ClF2N6NaO4	(M+Na)+
669.1624	1	509.15	C33H27ClF2N6NaO4	(M+Na)+
670.1652	1	107.49	C33H27ClF2N6NaO4	(M+Na)+

--- End Of Report ---

^{1}H-NMR spectrum of compound **12b**

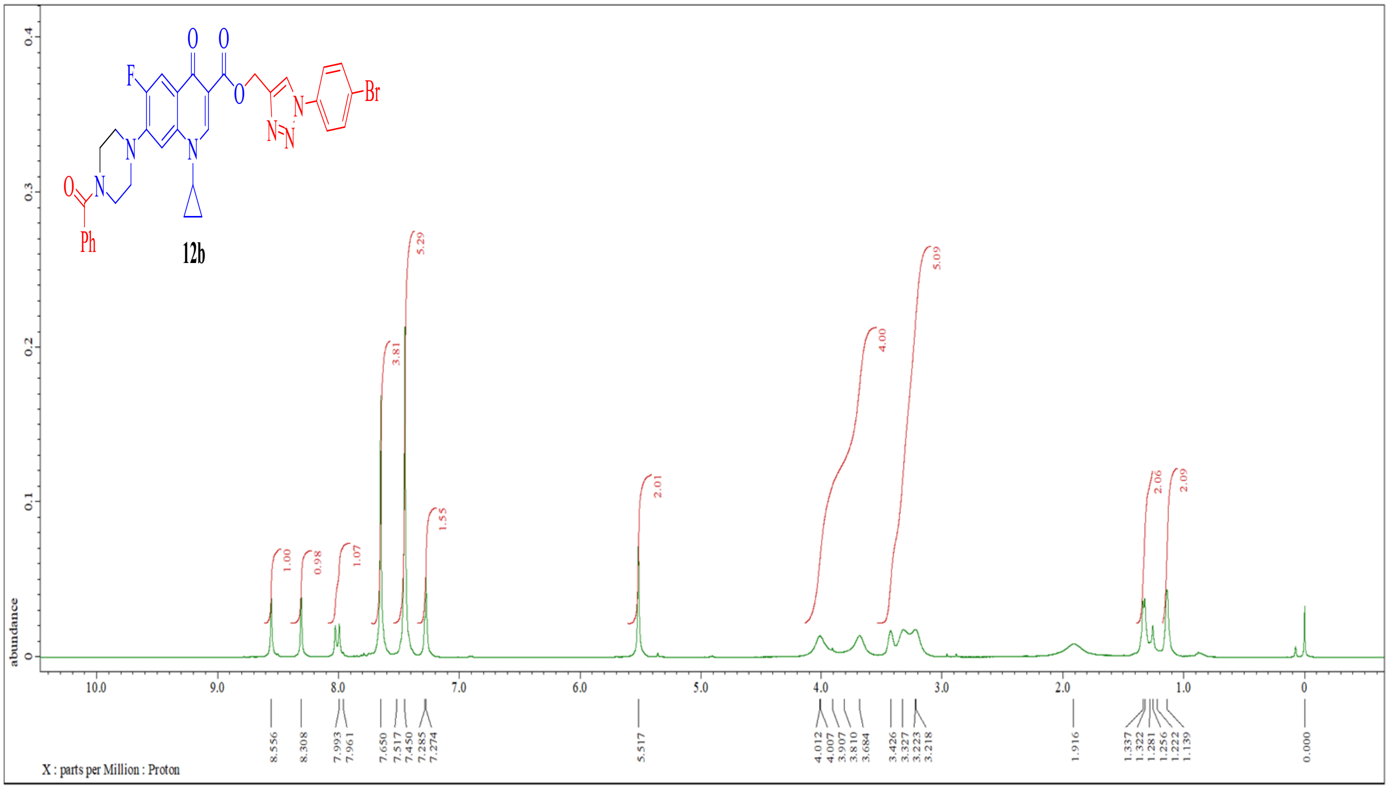

^{13}C-NMR spectrum of compound **12b**

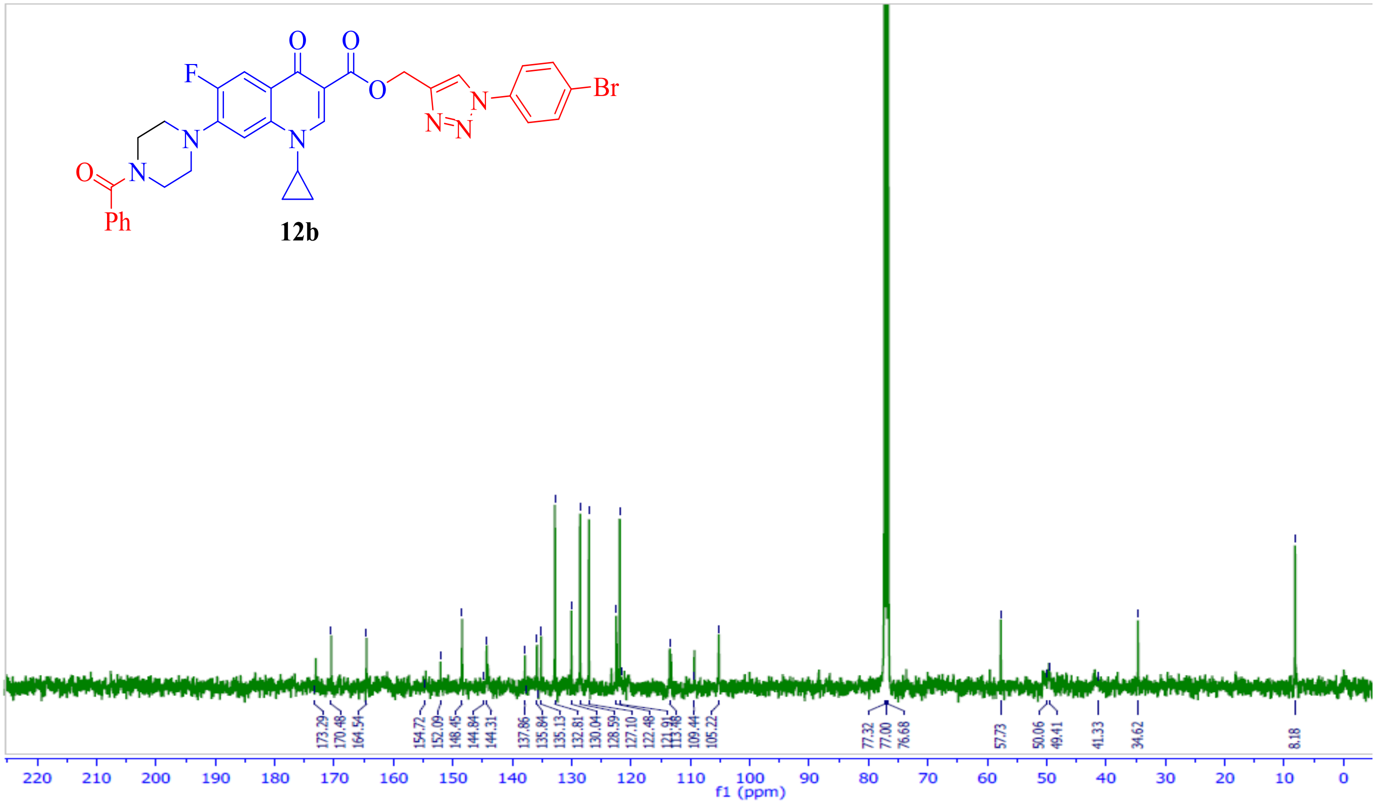

^{1}H-NMR spectrum of compound **12c**

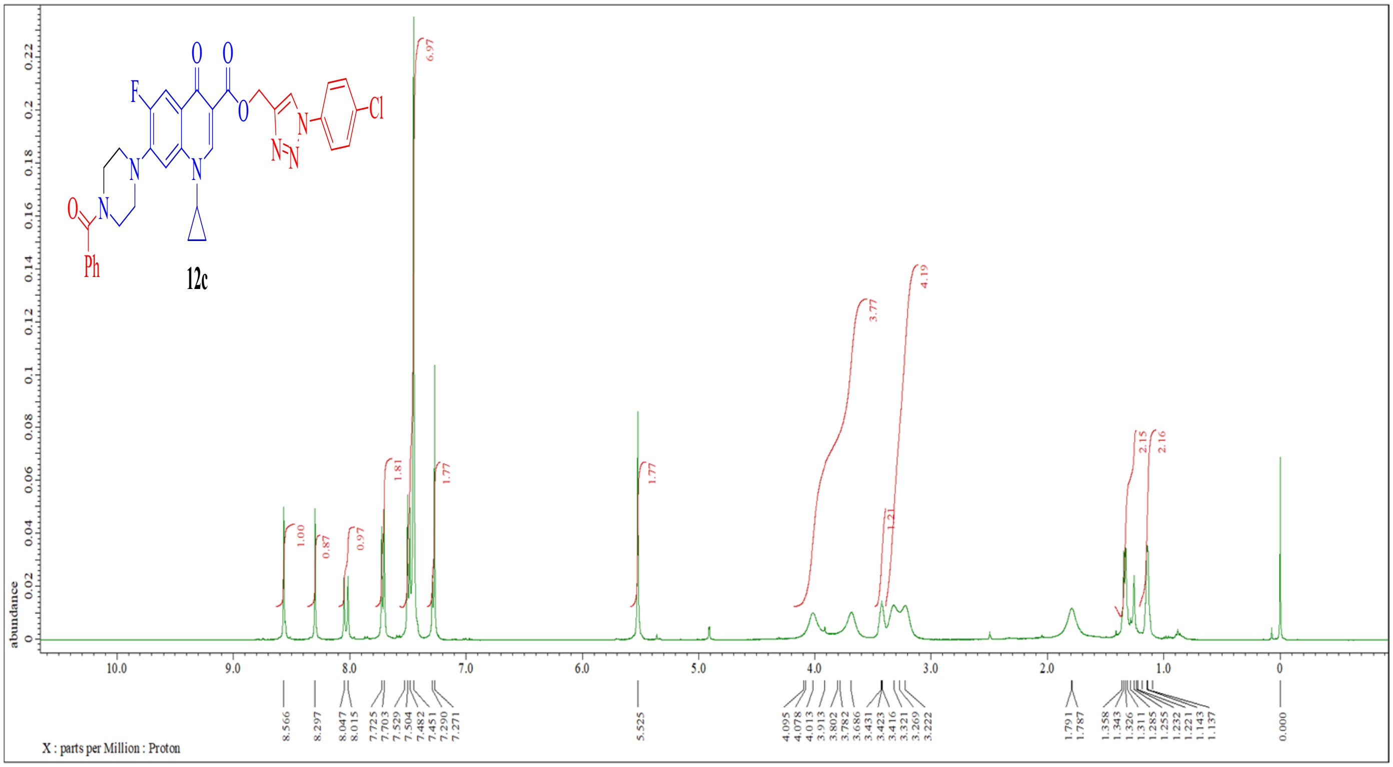

^{13}C-NMR spectrum of compound **12c**

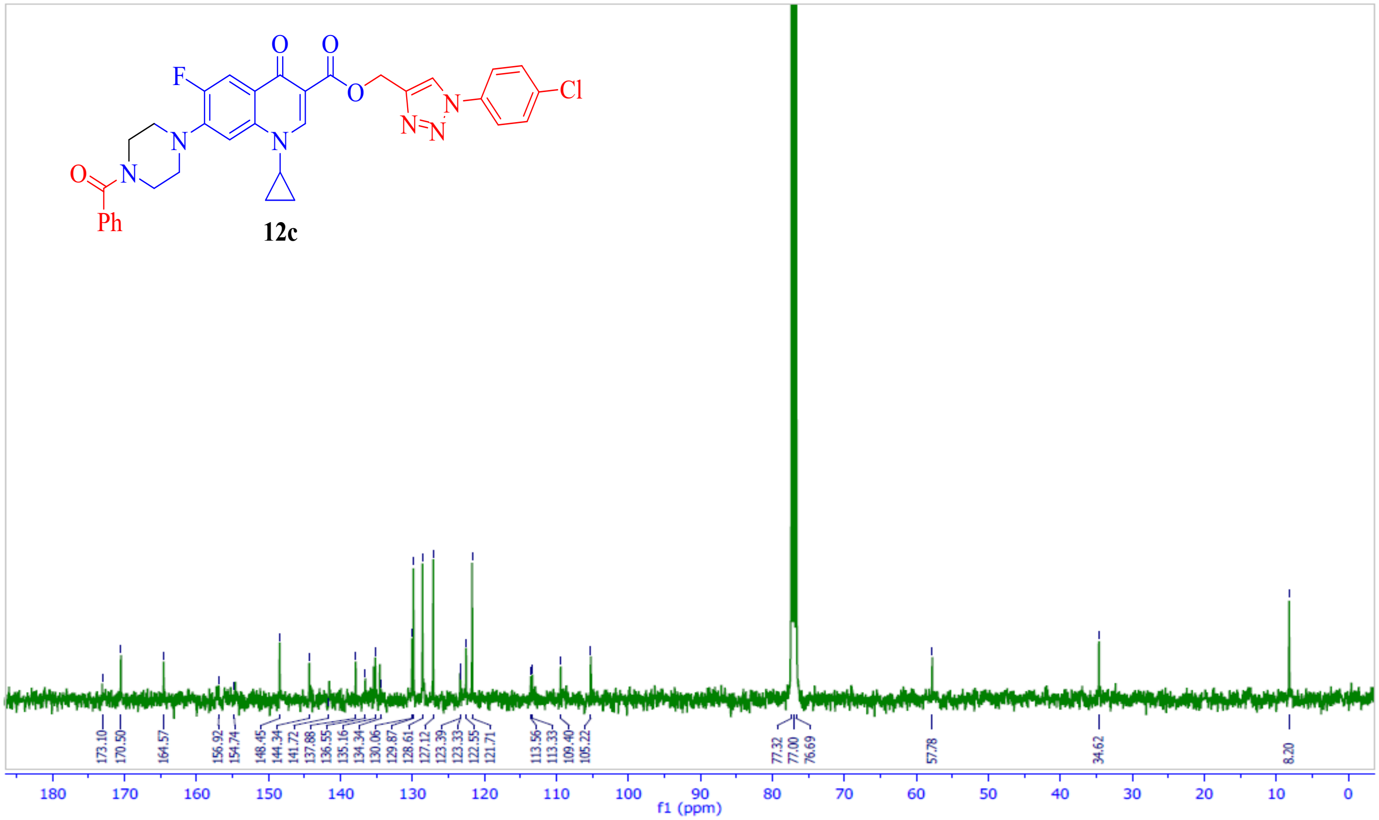

HRMS spectrum of compound **12c**

**Data File**	CPR-29.d	**Sample Name**	CPR-29
**Sample Type**	Sample	**Position**	P1-C4
**Instrument Name**	Instrument 1	**User Name**	
**Acq Method**	MS Scan_02.m	**Acquired Time**	07-06-2023 15:45:31
**IRM Calibration Status**	Success	**DA Method**	Default.m
**Comment**			

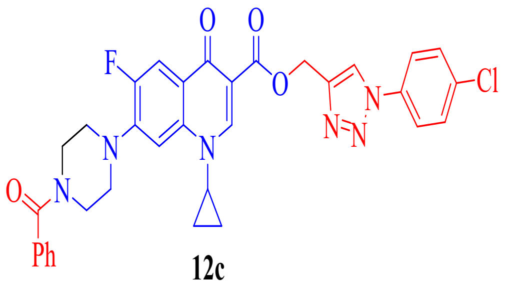

12c

**Sample Group**		**Info.**	3
**Acquisition SW Version**	6200 series TOF/6500 series Q-TOF B.05.01 (B5125)		

Compound Table

Compound Label	RT	Mass	Abund	Formula	Tgt Mass	Diff (ppm)	MFG Formula	DB Formula
Cpd 1: C33 H28 Cl F N6 O4	0.096	626.1855	57778	C33 H28 Cl F N6 O4	626.1845	1.6	C33 H28 Cl F N6 O4	C33 H28 Cl F N6 O4

Compound Label	*m/z*	RT	Algorithm	Mass
Cpd 1: C33 H28 Cl F N6 O4	627.1926	0.096	Find By Formula	626.1855

MS Zoomed Spectrum

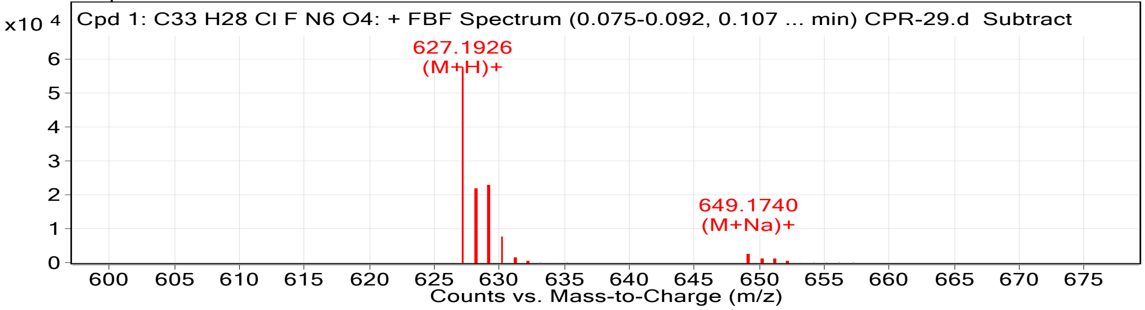

MS Spectrum Peak List

m/z	z	Abund	Formula	Ion
627.1926	1	57778.17	C33H29ClFN6O4	(M+H)+
628.1961	1	21749.25	C33H29ClFN6O4	(M+H)+
629.1918	1	22451.2	C33H29ClFN6O4	(M+H)+
630.1934	1	7549.82	C33H29ClFN6O4	(M+H)+
631.1955	1	1414.02	C33H29ClFN6O4	(M+H)+
632.1977	1	156.17	C33H29ClFN6O4	(M+H)+
649.174	1	2505.56	C33H28ClFN6NaO4	(M+Na)+
650.1767	1	919.03	C33H28ClFN6NaO4	(M+Na)+
651.1725	1	919.59	C33H28ClFN6NaO4	(M+Na)+
652.1748	1	248.73	C33H28ClFN6NaO4	(M+Na)+

--- End Of Report ---

^{1}H-NMR spectrum of compound **12d**

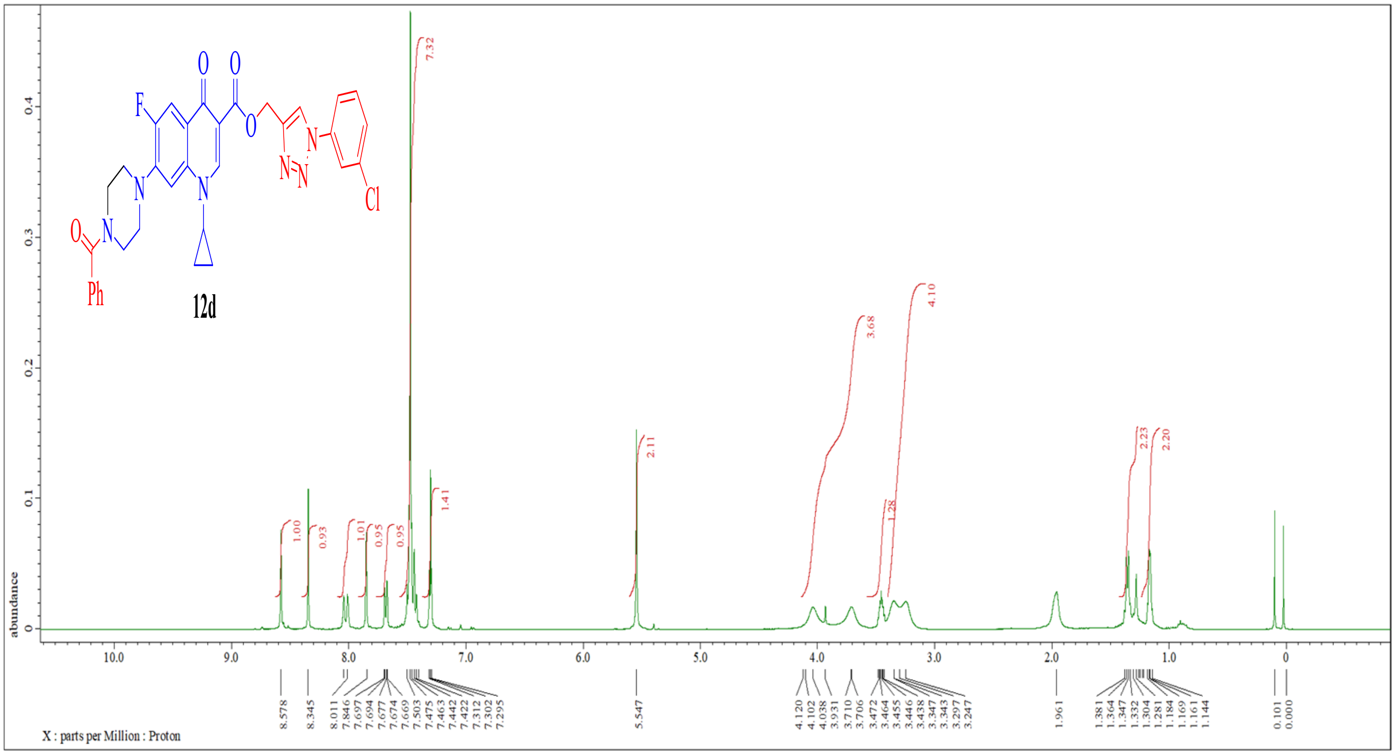

^{13}C-NMR spectrum of compound **12d**

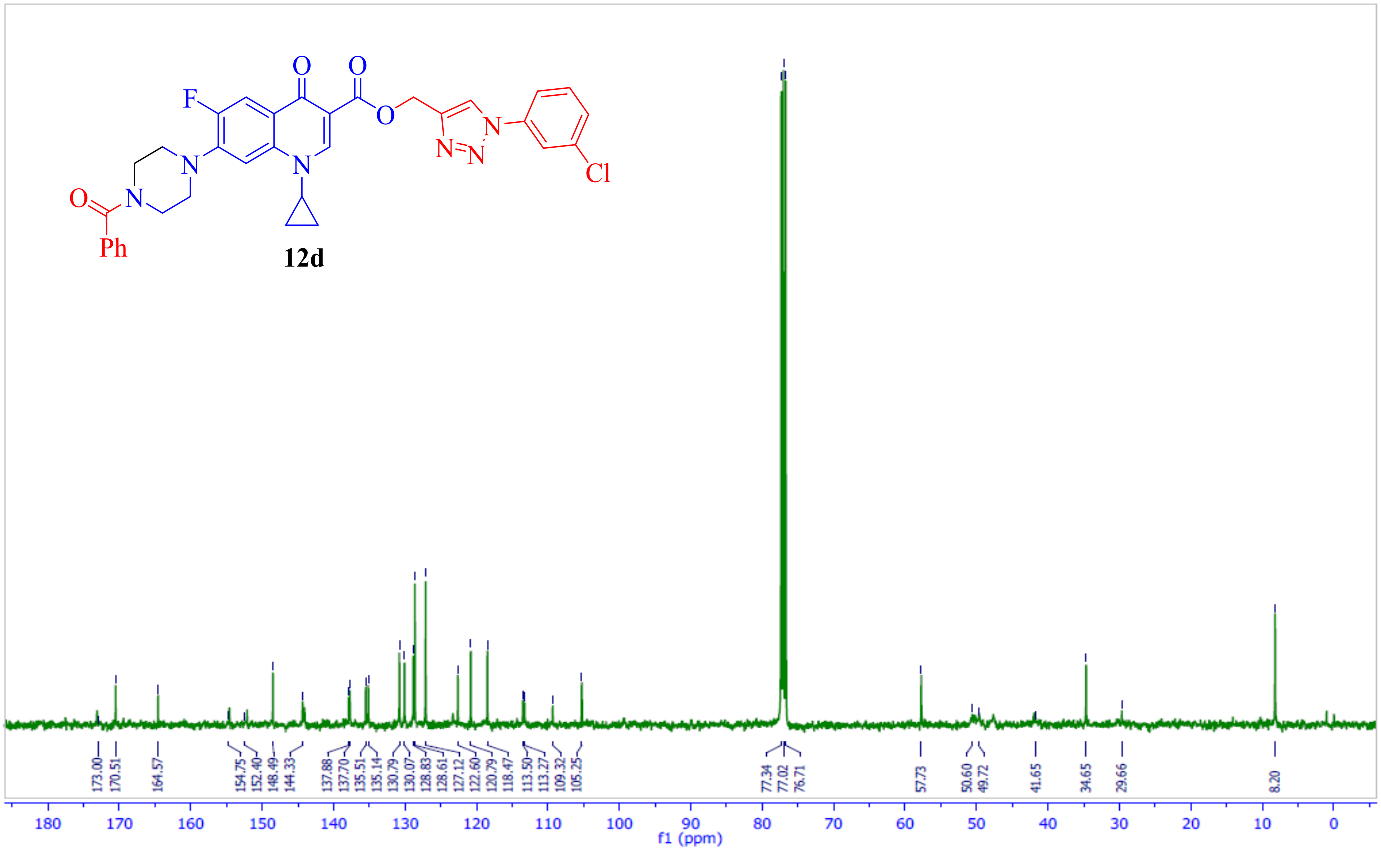

HRMS spectrum of compound **12d**

**Data File**	CPR-30.d	**Sample Name**	CPR-30
**Sample Type**	Sample	**Position**	P1-B8
**Instrument Name**	Instrument 1	**User Name**	
**Acq Method**	MS Scan_02.m	**Acquired Time**	08-06-2023 13:25:21
**IRM Calibration Status**	Success	**DA Method**	Default.m
**Comment**			

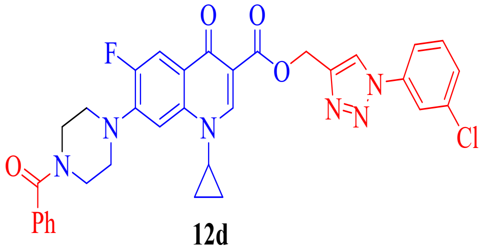

12d

**Sample Group**		**Info.**	3
**Acquisition SW Version**	6200 series TOF/6500 series Q-TOF B.05.01 (B5125)		

Compound Table

Compound Label	RT	Mass	Abund	Formula	Tgt Mass	Diff (ppm)	MFG Formula	DB Formula
Cpd 1: C33 H28 Cl F N6 O4	0.095	626.1862	8348	C33 H28 Cl F N6 O4	626.1845	2.76	C33 H28 Cl F N6 O4	C33 H28 Cl F N6 O4

Compound Label	*m/z*	RT	Algorithm	Mass
Cpd 1: C33 H28 Cl F N6 O4	649.1753	0.095	Find By Formula	626.1862

MS Zoomed Spectrum

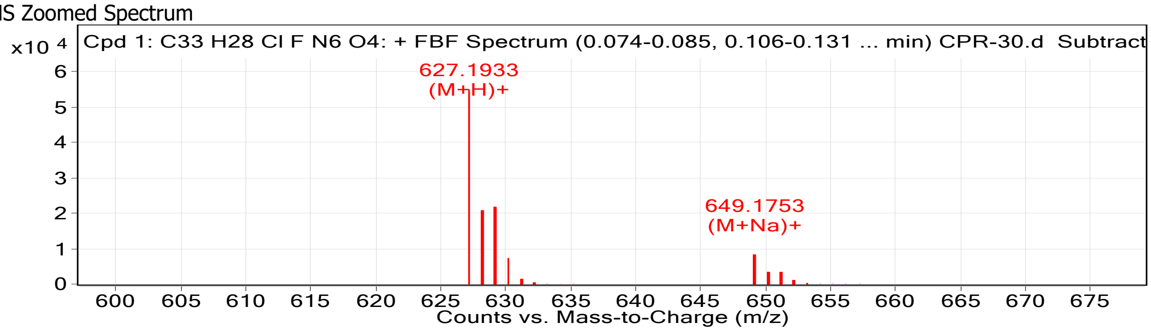

MS Spectrum Peak List

m/z	z	Abund	Formula	Ion
627.1933	1	54838.88	C33H29ClFN6O4	(M+H)+
628.1966	1	20629.42	C33H29ClFN6O4	(M+H)+
629.1926	1	21348.88	C33H29ClFN6O4	(M+H)+
630.1942	1	7257.27	C33H29ClFN6O4	(M+H)+
631.1963	1	1349.39	C33H29ClFN6O4	(M+H)+
632.1986	1	155.5	C33H29ClFN6O4	(M+H)+
649.1753	1	8347.58	C33H28ClFN6NaO4	(M+Na)+
650.1781	1	3174.49	C33H28ClFN6NaO4	(M+Na)+
651.1741	1	3246.1	C33H28ClFN6NaO4	(M+Na)+
652.1759	1	1016.01	C33H28ClFN6NaO4	(M+Na)+
653.1795	1	154.22	C33H28ClFN6NaO4	(M+Na)+

--- End Of Report ---

^{1}H-NMR spectrum of compound **12e**

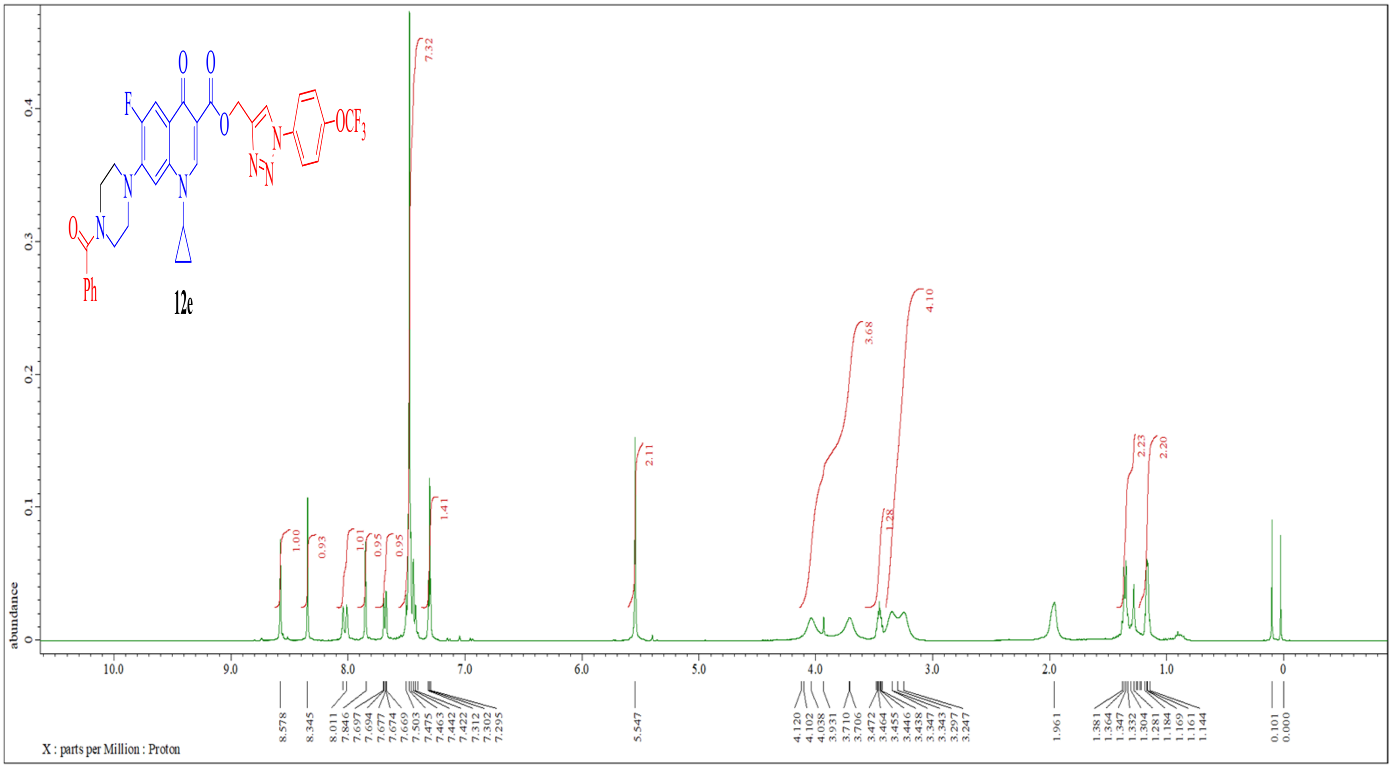

^{13}C-NMR spectrum of compound **12e**

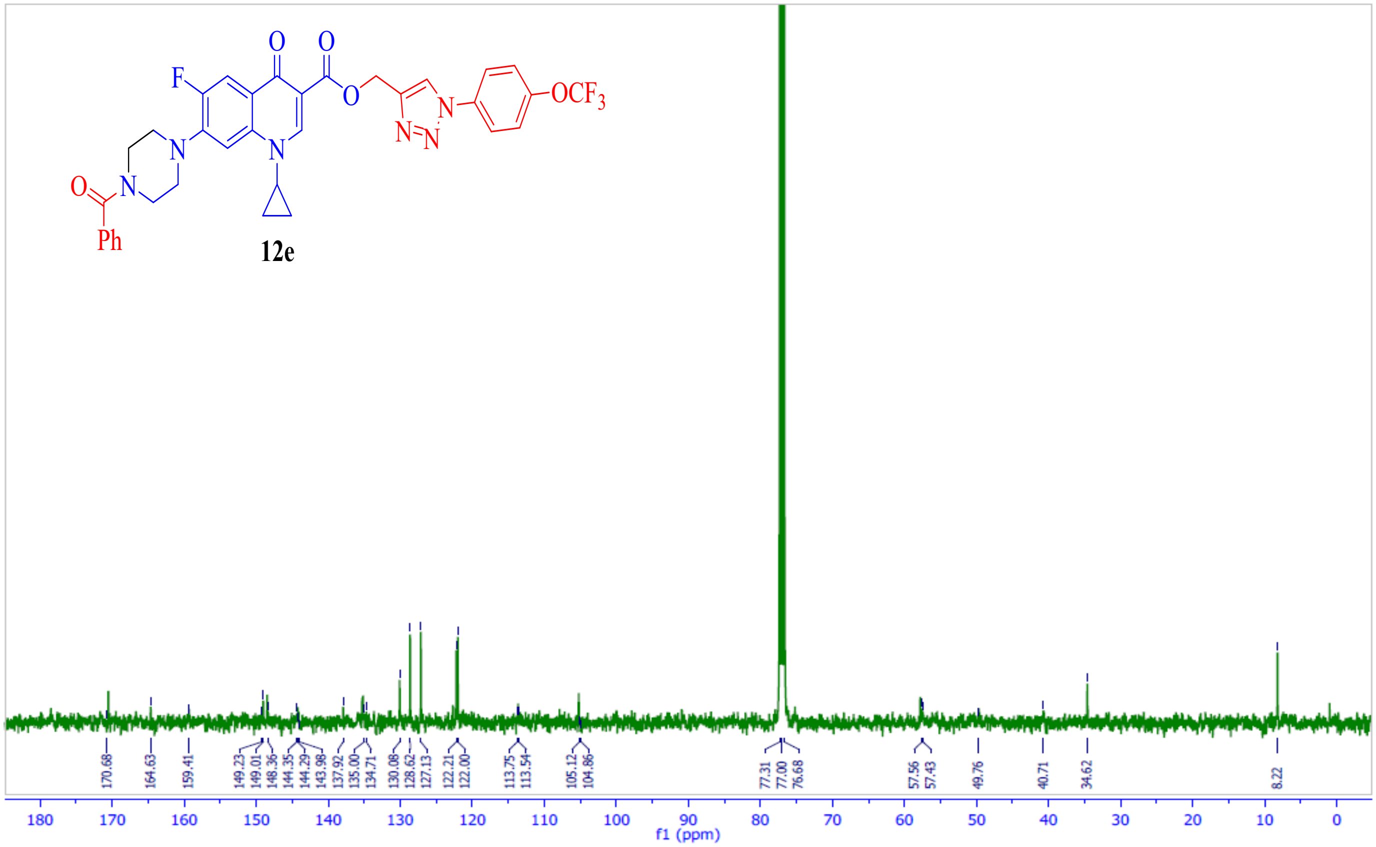

^{19}F-NMR spectrum of compound **12e**

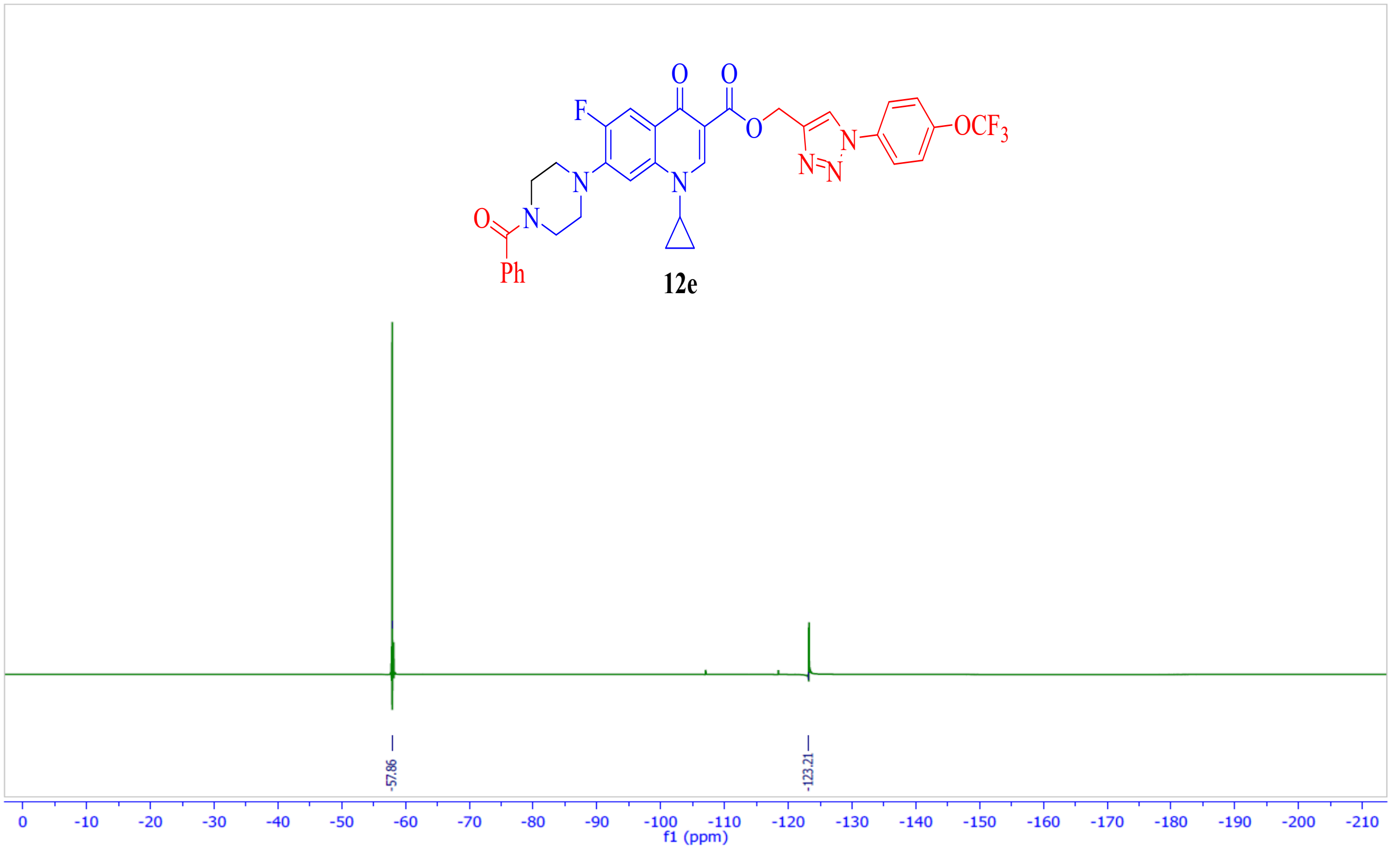

IR spectrum of compound **12e**

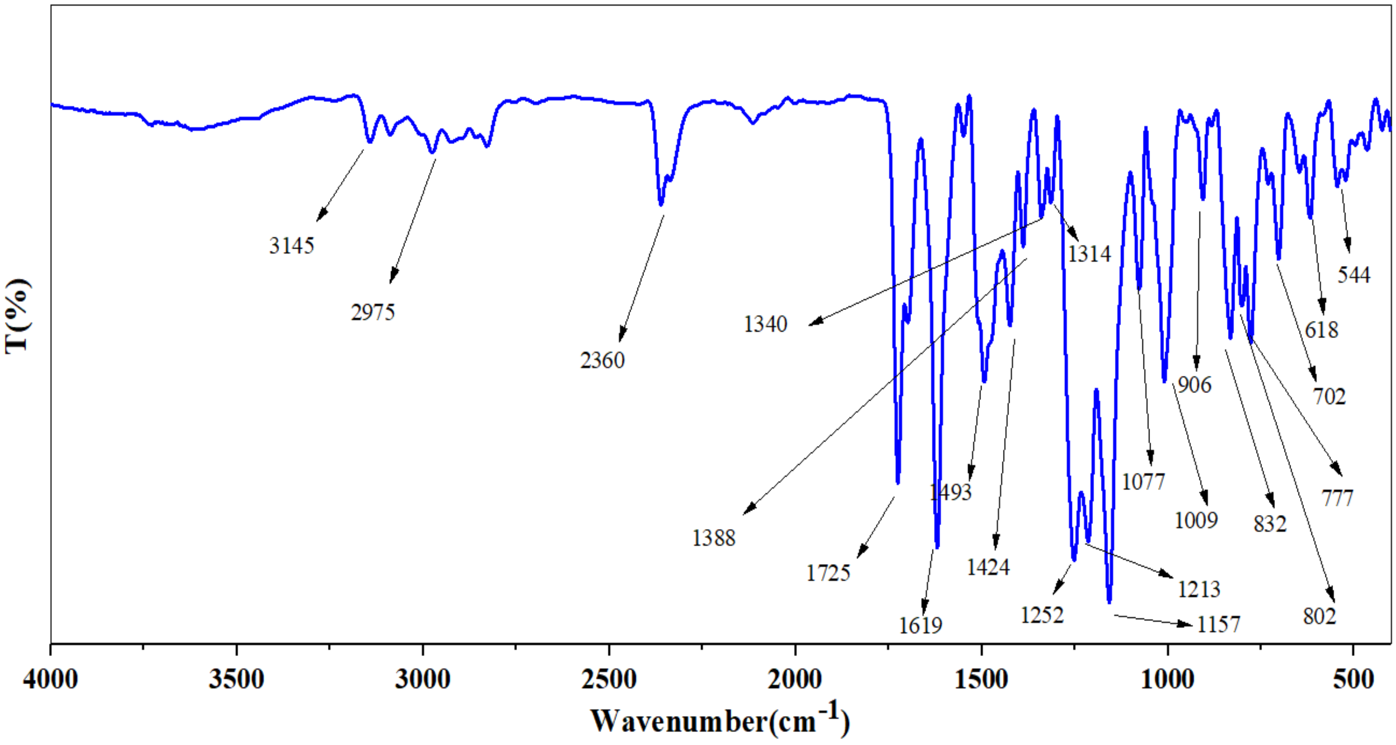

HRMS spectrum of compound **12e**

**Data File**	CPR-31.d	**Sample Name**	CPR-31
**Sample Type**	Sample	**Position**	P1-B9
**Instrument Name**	Instrument 1	**User Name**	
**Acq Method**	MS Scan_02.m	**Acquired Time**	08-06-2023 13:28:50
**IRM Calibration Status**	Success	**DA Method**	Default.m
**Comment**			

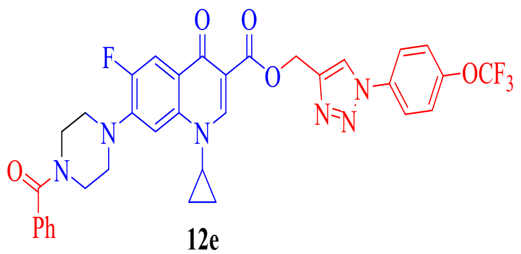

12e

**Sample Group**		**Info.**	3
**Acquisition SW Version**	6200 series TOF/6500 series Q-TOF B.05.01 (B5125)		

Compound Table

Compound Label	RT	Mass	Abund	Formula	Tgt Mass	Diff (ppm)	MFG Formula	DB Formula
Cpd 1: C34 H28 F4 N6 O5	0.1	676.2078	2543	C34 H28 F4 N6 O5	676.2057	3.11	C34 H28 F4 N6 O5	C34 H28 F4 N6 O5

Compound Label	*m/z*	RT	Algorithm	Mass
Cpd 1: C34 H28 F4 N6 O5	699.1963	0.1	Find By Formula	676.2078

MS Zoomed Spectrum

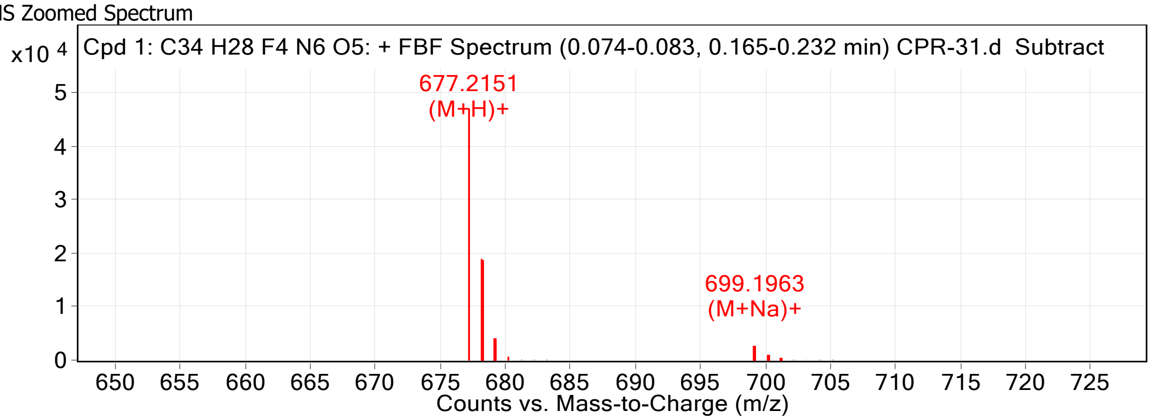

MS Spectrum Peak List

m/z	z	Abund	Formula	Ion
677.2151	1	47100.44	C34H29F4N6O5	(M+H)+
678.2183	1	18830.41	C34H29F4N6O5	(M+H)+
679.2207	1	3979.11	C34H29F4N6O5	(M+H)+
680.2232	1	596.24	C34H29F4N6O5	(M+H)+
699.1963	1	2543.26	C34H28F4N6NaO5	(M+Na)+
700.1992	1	978.64	C34H28F4N6NaO5	(M+Na)+
701.2016	1	141.93	C34H28F4N6NaO5	(M+Na)+

--- End Of Report ---

^{1}H-NMR spectrum of compound **12f**

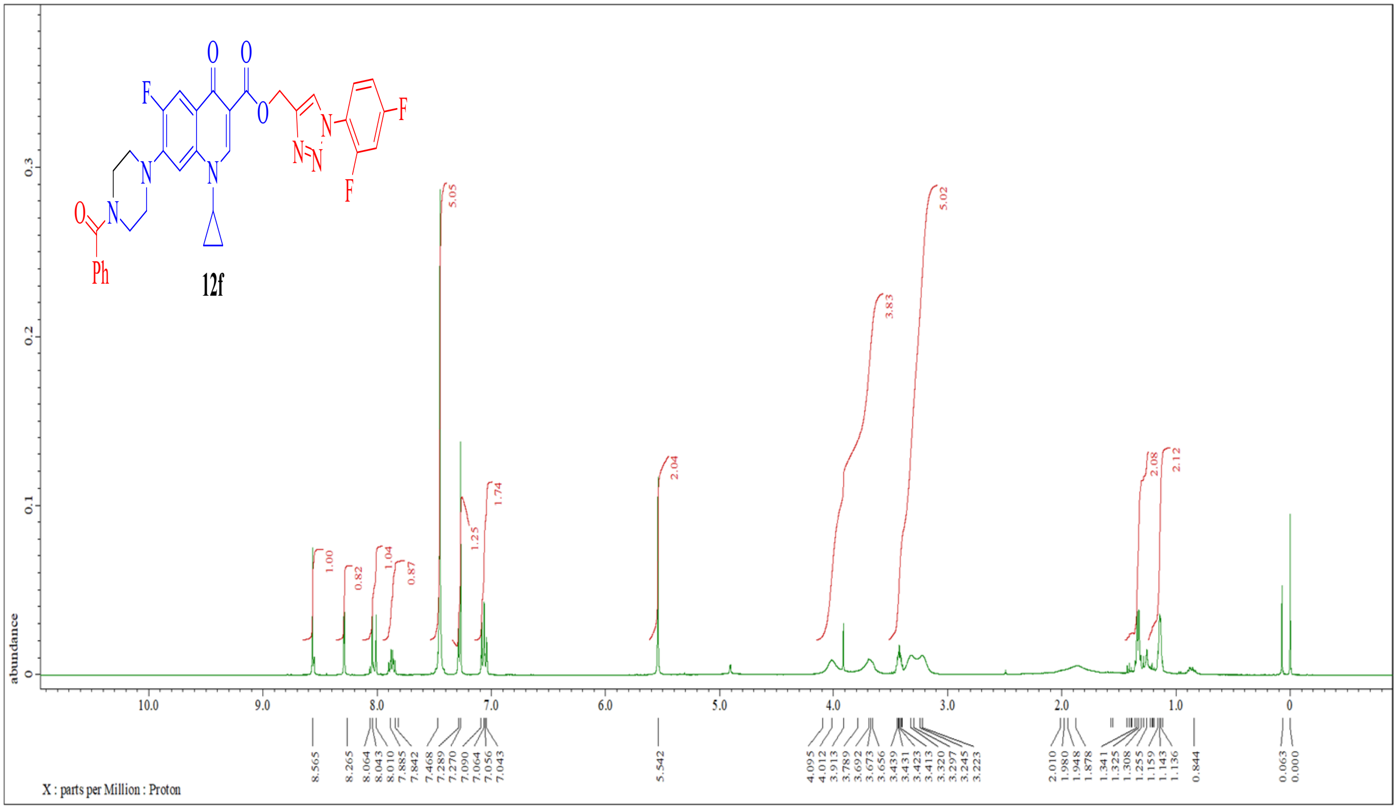

^{13}C-NMR spectrum of compound **12f**

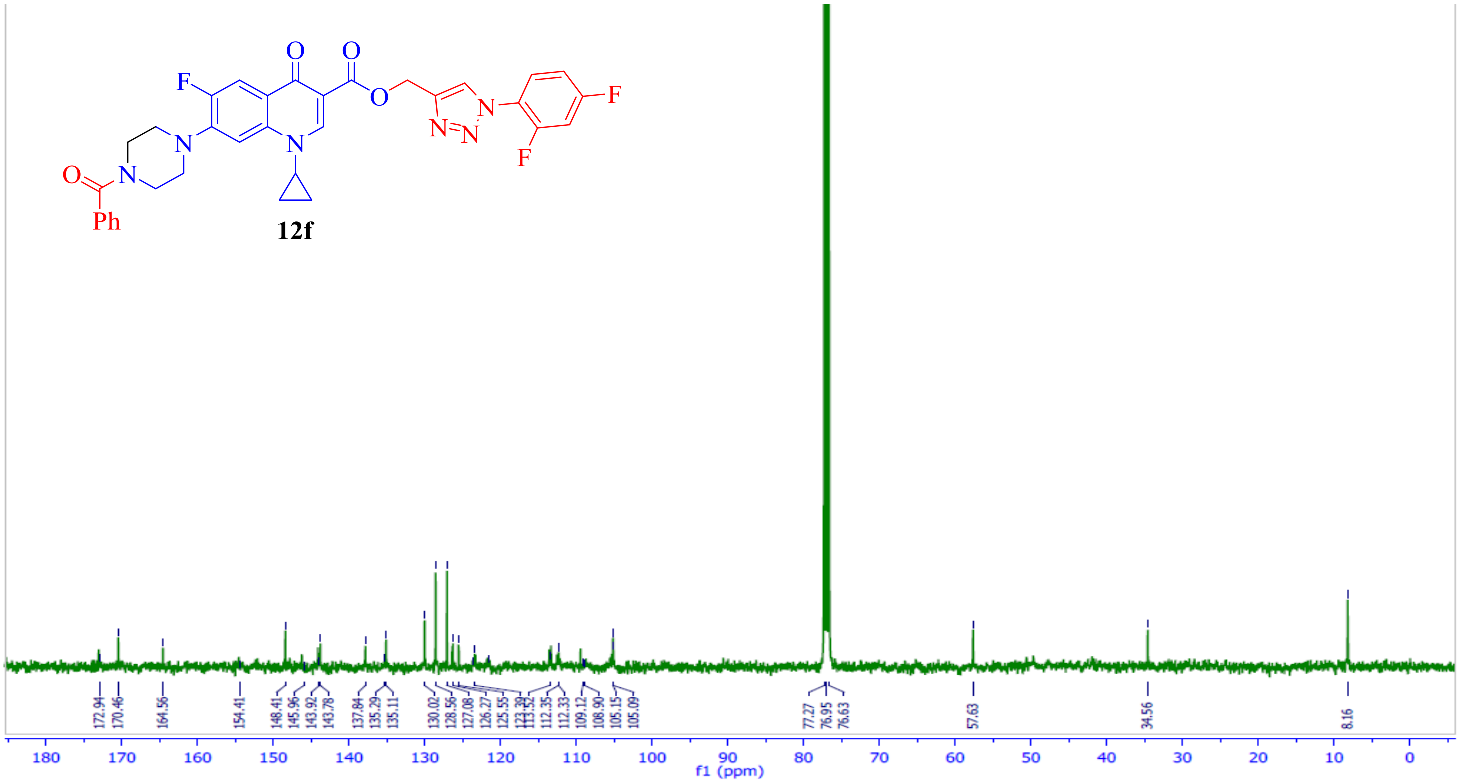

^{19}F-NMR spectrum of compound **12f**

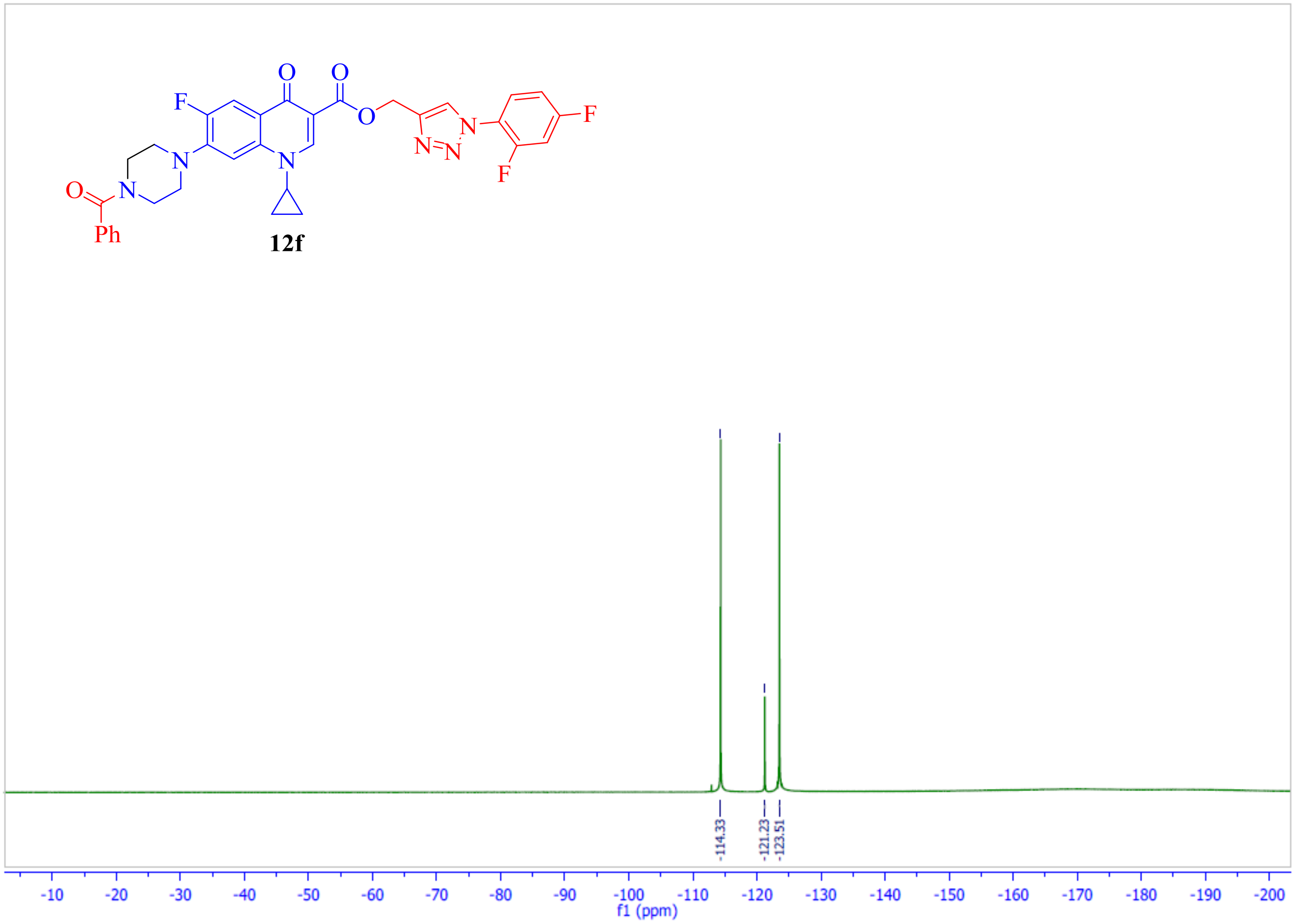

HRMS spectrum of compound **12f**

**Data File**	CPR-32.d	**Sample Name**	CPR-32
**Sample Type**	Sample	**Position**	P1-C1
**Instrument Name**	Instrument 1	**User Name**	
**Acq Method**	MS Scan_02.m	**Acquired Time**	08-06-2023 13:30:51
**IRM Calibration Status**	Success	**DA Method**	Default.m
**Comment**			

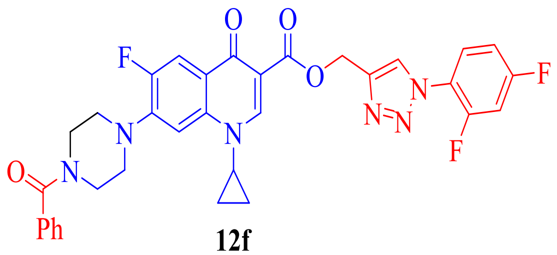

12f

**Sample Group**		**Info.**	3
**Acquisition SW Version**	6200 series TOF/6500 series Q-TOF B.05.01 (B5125)		

Compound Table

Compound Label	RT	Mass	Abund	Formula	Tgt Mass	Diff (ppm)	MFG Formula	DB Formula
Cpd 1: C33 H27 F3 N6 O4	0.097	628.2067	4866	C33 H27 F3 N6 O4	628.2046	3.32	C33 H27 F3 N6 O4	C33 H27 F3 N6 O4

Compound Label	*m/z*	RT	Algorithm	Mass
Cpd 1: C33 H27 F3 N6 O4	651.1955	0.097	Find By Formula	628.2067

MS Zoomed Spectrum

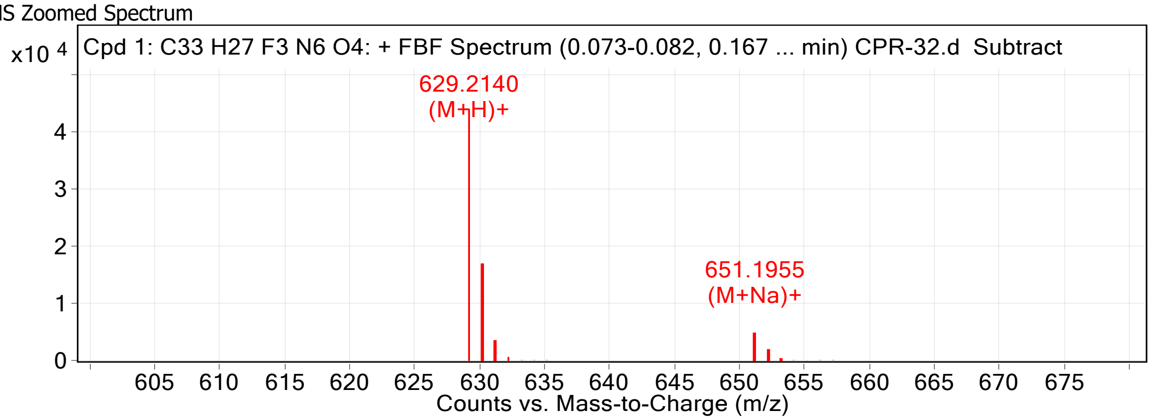

MS Spectrum Peak List

m/z	z	Abund	Formula	Ion
629.214	1	44055.39	C33H28F3N6O4	(M+H)+
630.2172	1	16910.61	C33H28F3N6O4	(M+H)+
631.2194	1	3342.13	C33H28F3N6O4	(M+H)+
632.2217	1	458.13	C33H28F3N6O4	(M+H)+
651.1955	1	4865.95	C33H27F3N6NaO4	(M+Na)+
652.1982	1	1723.25	C33H27F3N6NaO4	(M+Na)+
653.2012	1	289.33	C33H27F3N6NaO4	(M+Na)+

--- End Of Report ---
